# Supplementary material for: A chemical–genetic interaction map of small molecules using high‐throughput imaging in cancer cells
Source: Mol Syst Biol. 2015 Dec 28;11(12):846. doi: 10.15252/msb.20156400 (PMC4704494; doi:10.15252/msb.20156400)
Supplement: Supplementary file 1 — Appendix [file MSB-11-846-s001.pdf]

Appendix.

A chemical-genetic interaction map of small molecules using high-throughput imaging in cancer cells

Breinig et al.

Table of content:

Appendix Figure S1: Quality control.

Appendix Figure S2: Phenotypic features.

Appendix Figure S3: Quantitative analysis of chemical-genetic interactions across multiple phenotypic features.

Appendix Figure S4: Significant multiparametric chemical-genetic interactions.

Appendix Figure S5: Strong main effects of gene KO and associations of isogenic cell lines.

Appendix Figure S6: Synthetic lethal pharmacogenetic interactions.

Appendix Figure S7: Bendamustine and disulfiram interaction spectrum.

Appendix Figure S8: Clustering of chemical-genetic interactions.

Appendix Figure S9: Association between clustering of compounds as shown in Fig 5A and respective interaction spectra for drugs within clusters.

Appendix Figure S10: Integration of phenotypic profiling and chemical-genetic interaction mapping improves resolution.

Appendix Figure S11: Image-based chemical-genetic interaction analyses outperforms other methods.

Appendix Figure S12: The EGFR inhibitor tyrphostin AG555 impairs proteasome function.

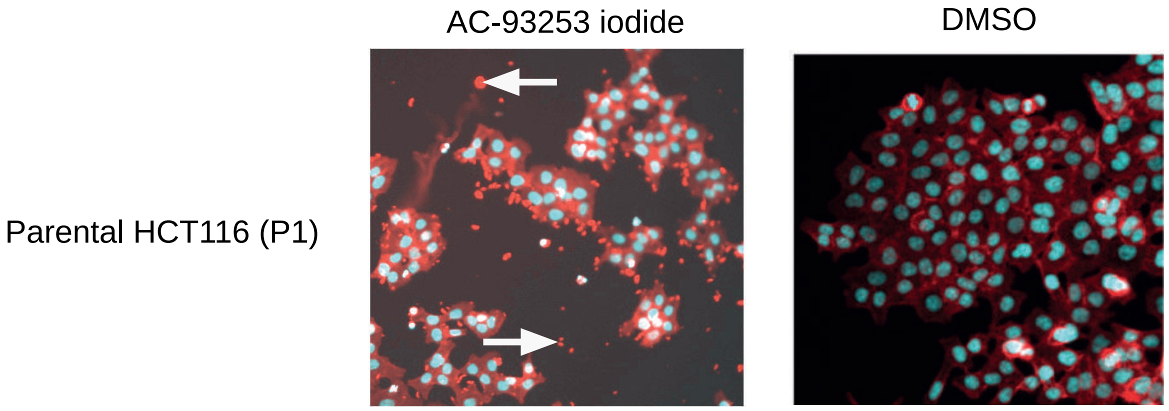

Appendix Figure S1. Quality control.
Images showing experimental artefact, such as autofluorescence of AC-93253 iodide, were removed from the analysis.
Here, a high fluorescence background and the occurence of fluorescent speckles (arrows) can be seen in the TRITC channel used to detect actin via Phalloidin-TRITC staining.
Image of DMSO treated cells is included as a reference, showing low fluorescence background and specific actin staining.

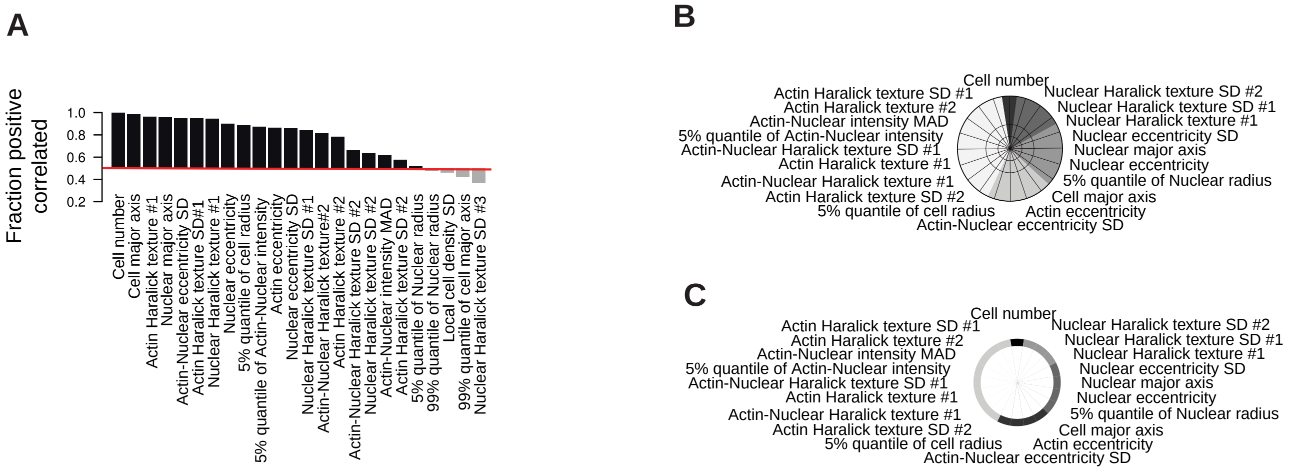

Appendix Figure S2. Phenotypic features.

A Feature names to Figure 1C. Linear decomposition selected a final set of 20 phenotypic features.

B Feature names to Figure 1D. Phenotypic features are grouped in 5 phenotypic categories and span phenoprints.

C Feature names to Figure 2 and 4. Phenotypic interactions for the selected 20 features are grouped in 5 phenotypic categories.

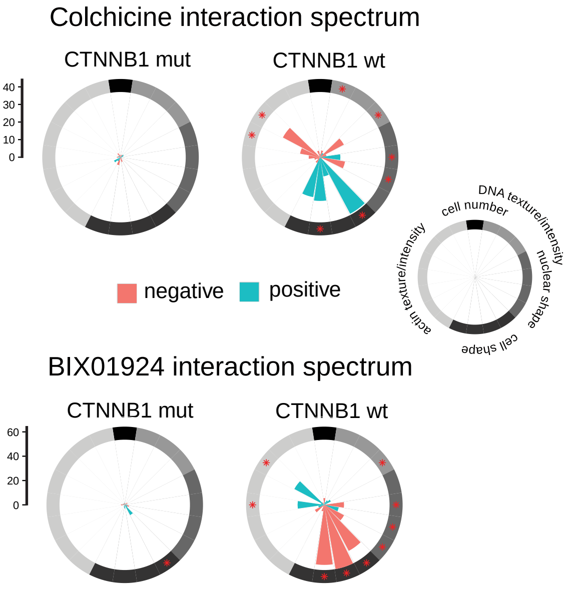

Appendix Figure S3. Quantitative analysis of chemical-genetic interactions across multiple phenotypic features.
Chemical-genetic interactions were calculated for all 20 phenotypic features as described.
Colchicine and BIX01294 show multiple interactions in CTNNB1 wt cells.
Phenotypic chemo-genomic interactions are shown unscaled.
Interactions are further categorized in positive and negative interactions according to the sign of interaction terms (see Material and Methods for details). * adjusted p-value < 0.01.

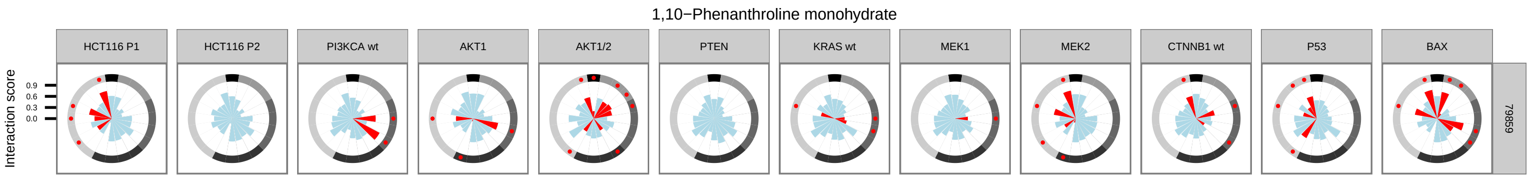
1,10−Phenanthroline monohydrate
Interaction score
0.9
0.6
0.3
0.0
HCT116 P1
HCT116 P2
PI3KCA wt
AKT1
AKT1/2
PTEN
KRAS wt
MEK1
MEK2
CTNNB1 wt
P53
BAX
79859

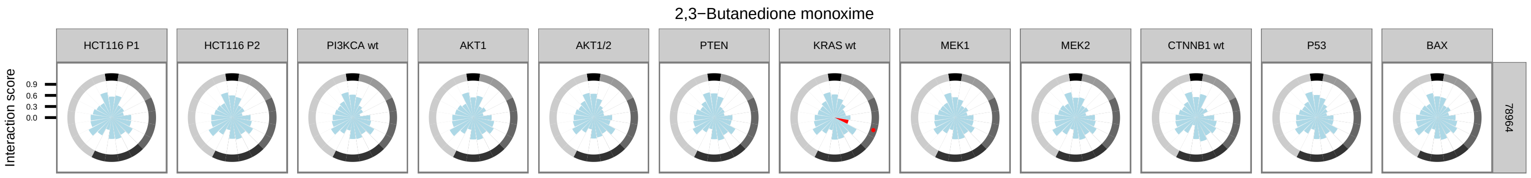
2,3−Butanedione monoxime
Interaction score
0.9
0.6
0.3
0.0
HCT116 P1
HCT116 P2
PI3KCA wt
AKT1
AKT1/2
PTEN
KRAS wt
MEK1
MEK2
CTNNB1 wt
P53
BAX
78964

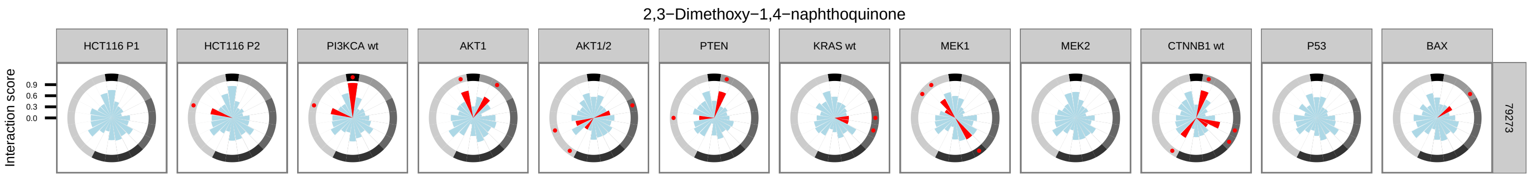
2,3−Dimethoxy−1,4−naphthoquinone
Interaction score
0.9
0.6
0.3
0.0
HCT116 P1
HCT116 P2
PI3KCA wt
AKT1
AKT1/2
PTEN
KRAS wt
MEK1
MEK2
CTNNB1 wt
P53
BAX
79273

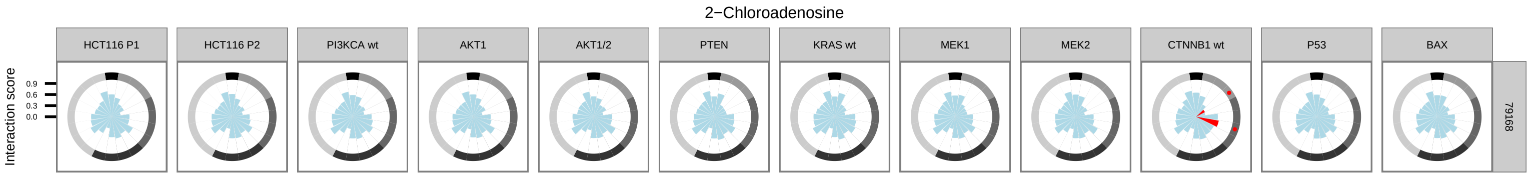
2−Chloroadenosine
Interaction score
0.9
0.6
0.3
0.0
HCT116 P1
HCT116 P2
PI3KCA wt
AKT1
AKT1/2
PTEN
KRAS wt
MEK1
MEK2
CTNNB1 wt
P53
BAX
79168

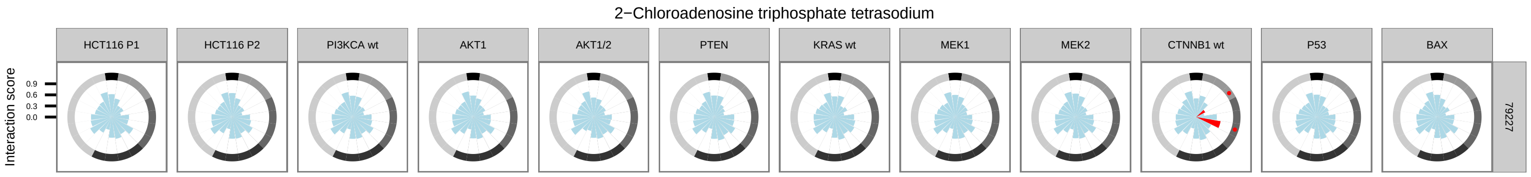
2−Chloroadenosine triphosphate tetrasodium
Interaction score
0.9
0.6
0.3
0.0
HCT116 P1
HCT116 P2
PI3KCA wt
AKT1
AKT1/2
PTEN
KRAS wt
MEK1
MEK2
CTNNB1 wt
P53
BAX
79227

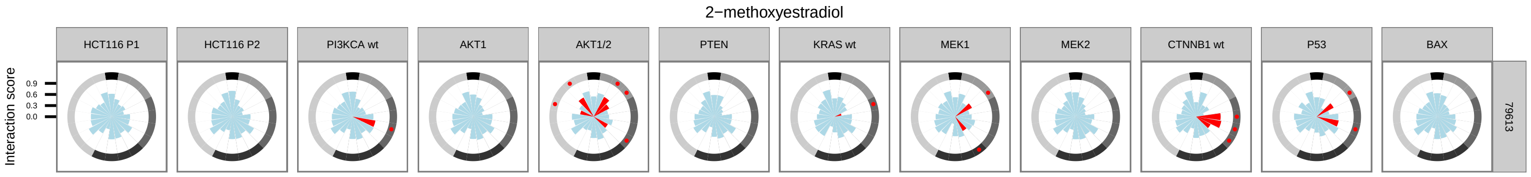
2−methoxyestradiol
Interaction score
0.9
0.6
0.3
0.0
HCT116 P1
HCT116 P2
PI3KCA wt
AKT1
AKT1/2
PTEN
KRAS wt
MEK1
MEK2
CTNNB1 wt
P53
BAX
79613

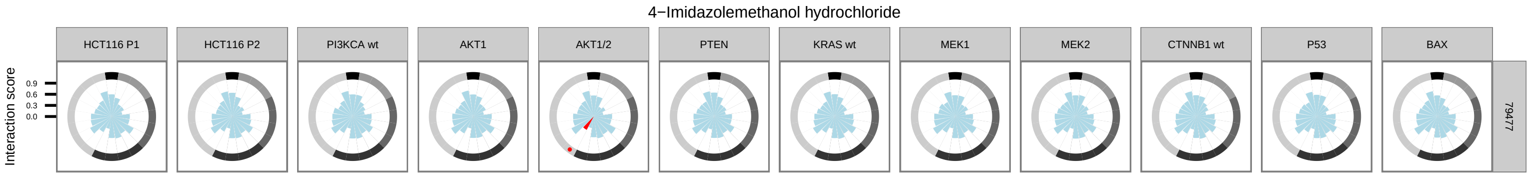
4−Imidazolemethanol hydrochloride
Interaction score
0.9
0.6
0.3
0.0
HCT116 P1
HCT116 P2
PI3KCA wt
AKT1
AKT1/2
PTEN
KRAS wt
MEK1
MEK2
CTNNB1 wt
P53
BAX
79477

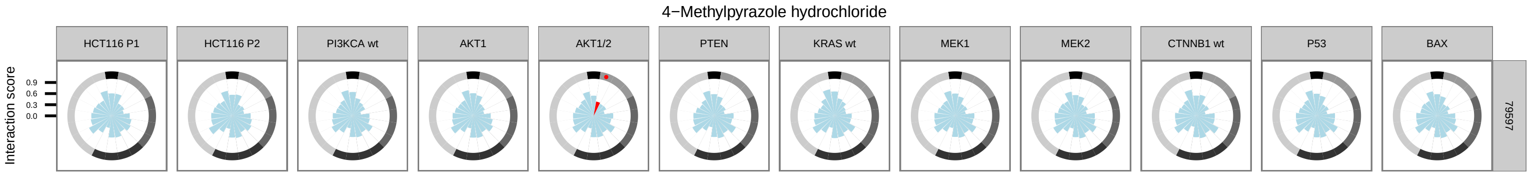
4−Methylpyrazole hydrochloride
Interaction score
0.9
0.6
0.3
0.0
HCT116 P1
HCT116 P2
PI3KCA wt
AKT1
AKT1/2
PTEN
KRAS wt
MEK1
MEK2
CTNNB1 wt
P53
BAX
79597

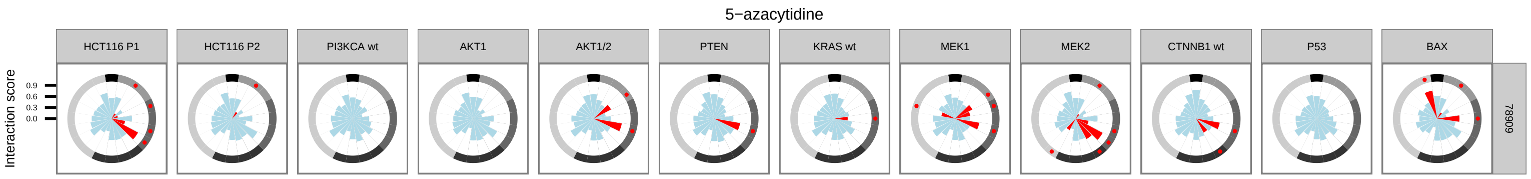
5−azacytidine
HCT116 P1
HCT116 P2
PI3KCA wt
AKT1
AKT1/2
PTEN
KRAS wt
MEK1
MEK2
CTNNB1 wt
P53
BAX
Interaction score
0.9
0.6
0.3
0.0
78909

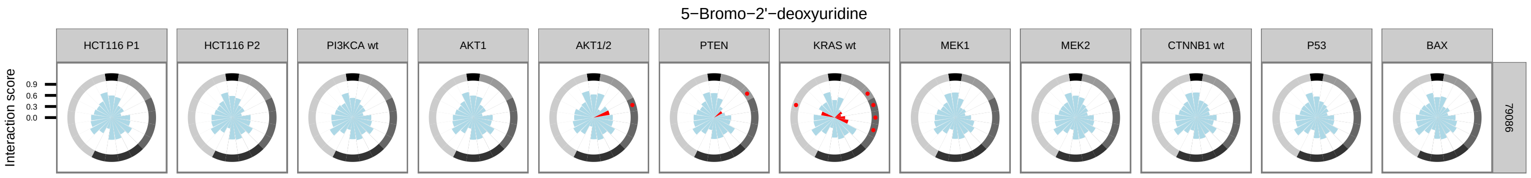
5−Bromo−2'−deoxyuridine
HCT116 P1
HCT116 P2
PI3KCA wt
AKT1
AKT1/2
PTEN
KRAS wt
MEK1
MEK2
CTNNB1 wt
P53
BAX
Interaction score
0.9
0.6
0.3
0.0
79086

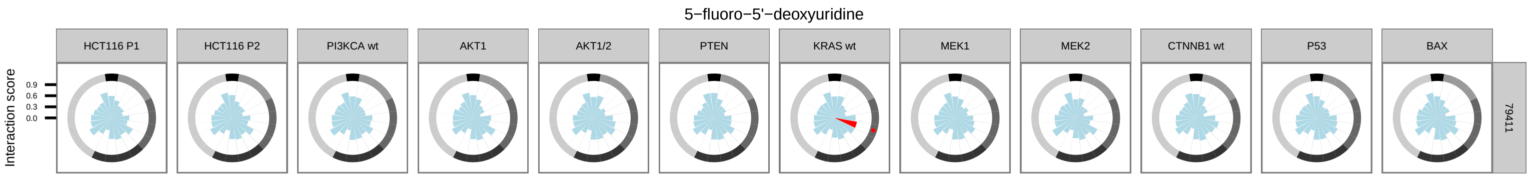
5−fluoro−5'−deoxyuridine
HCT116 P1
HCT116 P2
PI3KCA wt
AKT1
AKT1/2
PTEN
KRAS wt
MEK1
MEK2
CTNNB1 wt
P53
BAX
Interaction score
0.9
0.6
0.3
0.0
79411

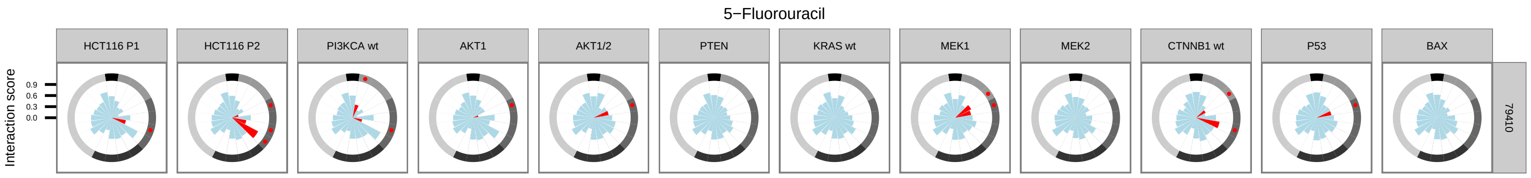
5−Fluorouracil
HCT116 P1
HCT116 P2
PI3KCA wt
AKT1
AKT1/2
PTEN
KRAS wt
MEK1
MEK2
CTNNB1 wt
P53
BAX
Interaction score
0.9
0.6
0.3
0.0
79410

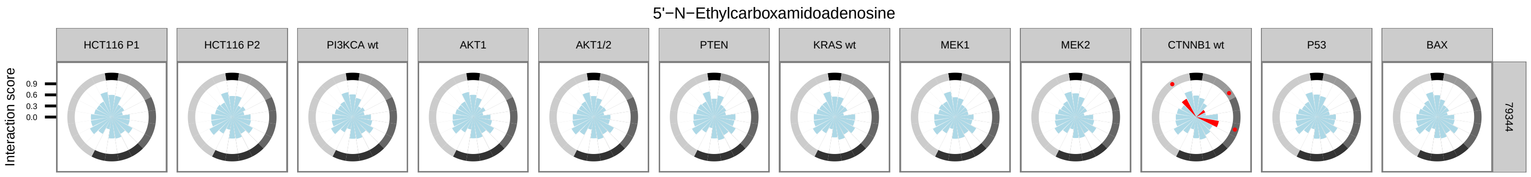
5'−N−Ethylcarboxamidoadenosine
HCT116 P1
HCT116 P2
PI3KCA wt
AKT1
AKT1/2
PTEN
KRAS wt
MEK1
MEK2
CTNNB1 wt
P53
BAX
Interaction score
0.9
0.6
0.3
0.0
79344

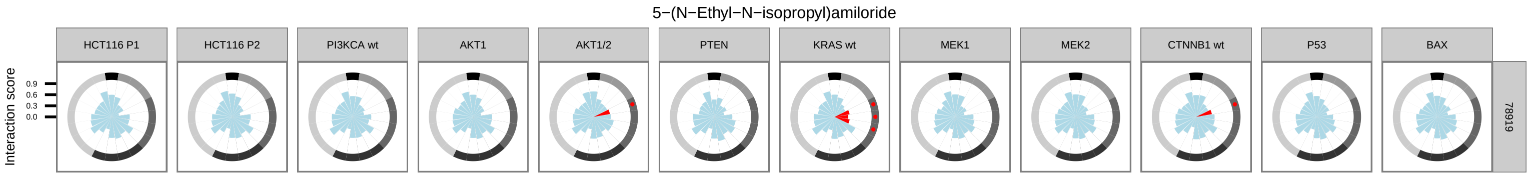
5−(N−Ethyl−N−isopropyl)amiloride
HCT116 P1
HCT116 P2
PI3KCA wt
AKT1
AKT1/2
PTEN
KRAS wt
MEK1
MEK2
CTNNB1 wt
P53
BAX
Interaction score
0.9
0.6
0.3
0.0
78919

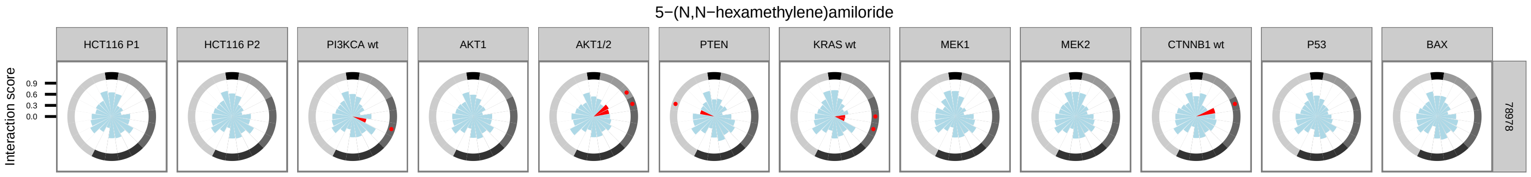
5−(N,N−hexamethylene)amiloride
HCT116 P1
HCT116 P2
PI3KCA wt
AKT1
AKT1/2
PTEN
KRAS wt
MEK1
MEK2
CTNNB1 wt
P53
BAX
Interaction score
0.9
0.6
0.3
0.0
78978

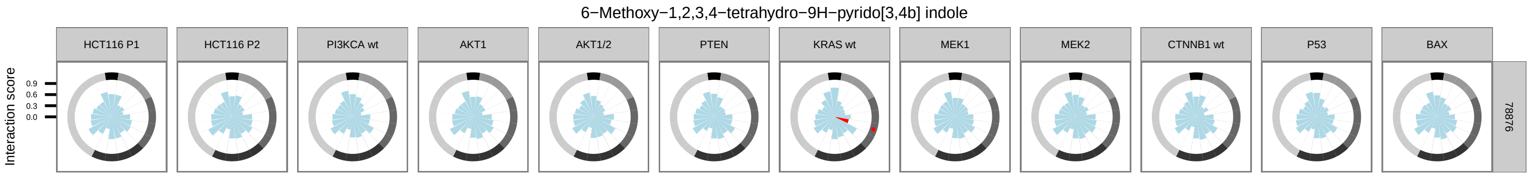
6−Methoxy−1,2,3,4−tetrahydro−9H−pyrido[3,4b] indole
HCT116 P1
HCT116 P2
PI3KCA wt
AKT1
AKT1/2
PTEN
KRAS wt
MEK1
MEK2
CTNNB1 wt
P53
BAX
Interaction score
0.9
0.6
0.3
0.0
78876

7−Cyclopentyl−5−(4−phenoxy)phenyl−7H−pyrrolo[2,3−d]pyrimidin−4−ylamine

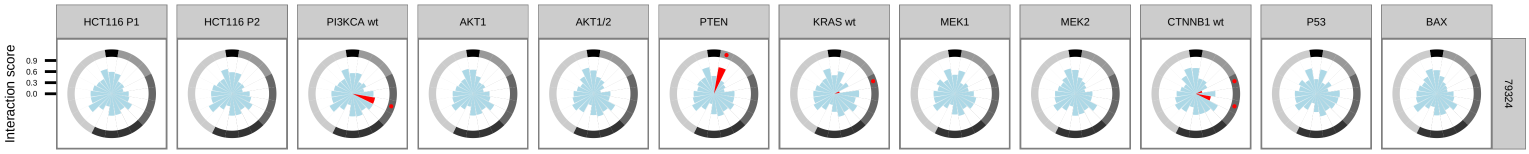

A−134974 dihydrochloride hydrate

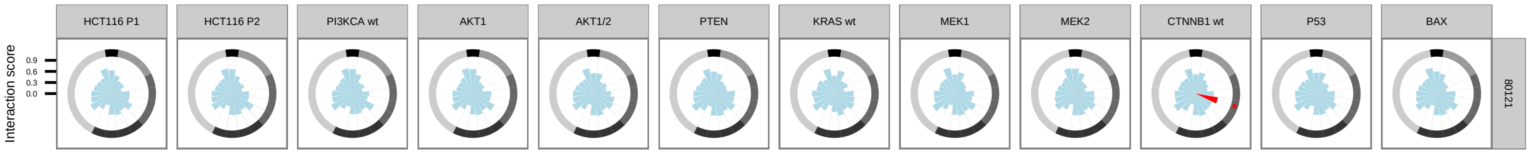

A−77636 hydrochloride

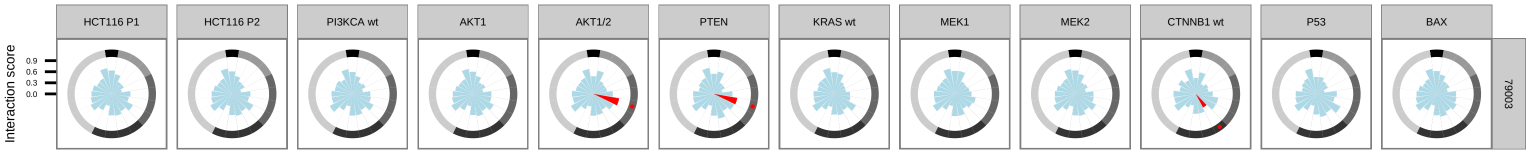

ABT−702 dihydrochloride

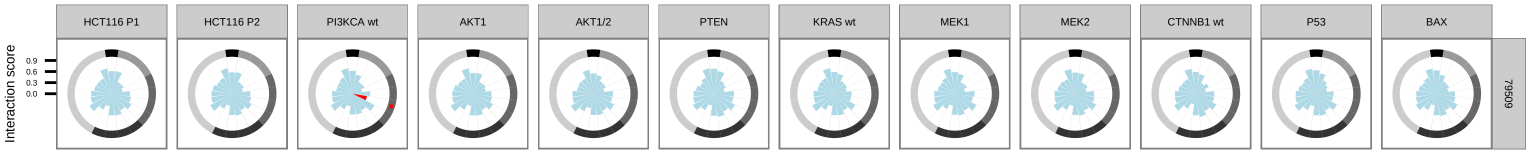

Actinonin

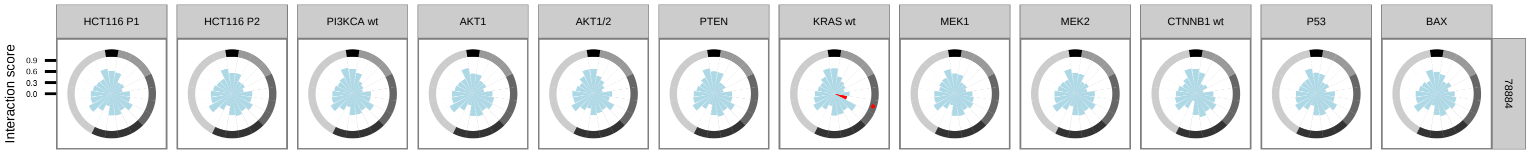

Aminopterin

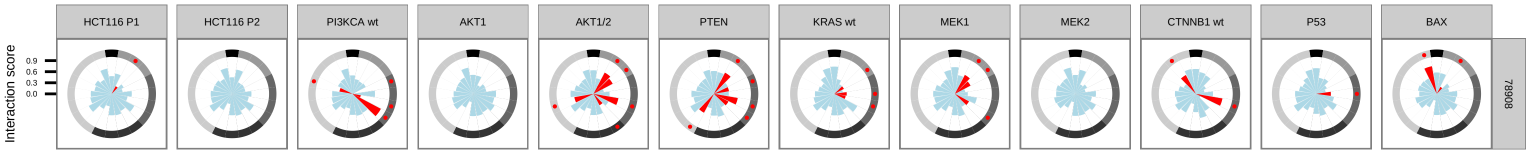

Ammonium pyrrolidinedithiocarbamate

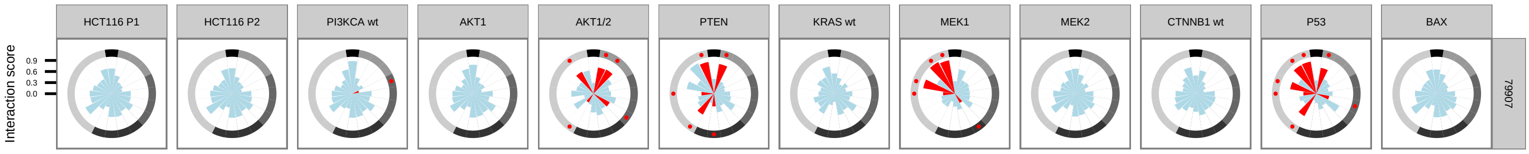

Amoxapine

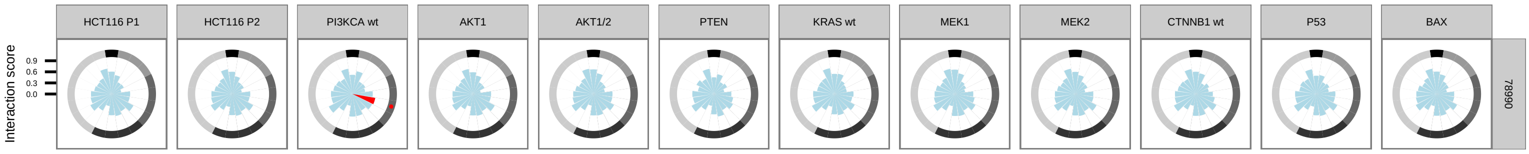

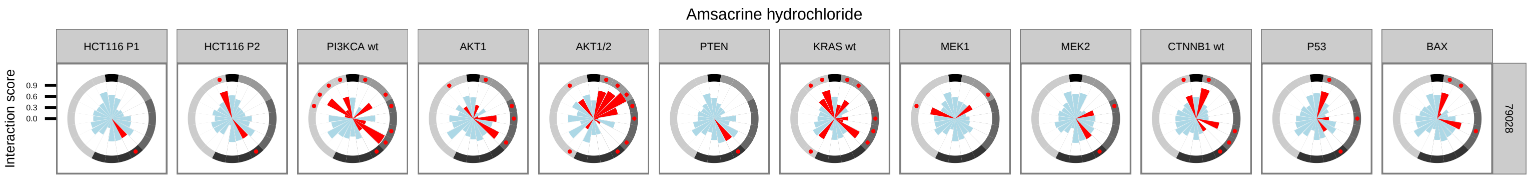
Amsacrine hydrochloride
Interaction score
0.9
0.6
0.3
0.0
HCT116 P1
HCT116 P2
PI3KCA wt
AKT1
AKT1/2
PTEN
KRAS wt
MEK1
MEK2
CTNNB1 wt
P53
BAX
79028

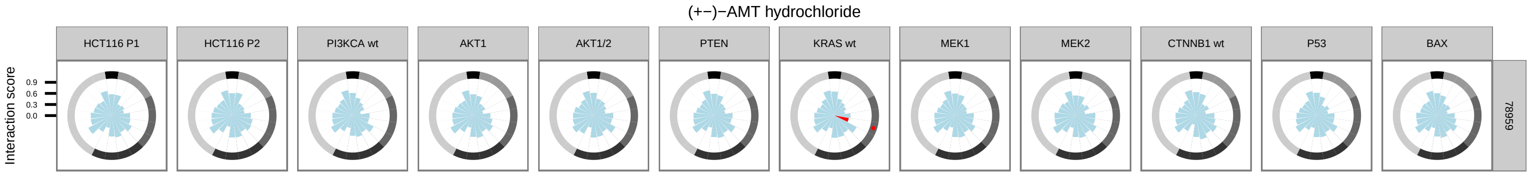
(+−)−AMT hydrochloride
Interaction score
0.9
0.6
0.3
0.0
HCT116 P1
HCT116 P2
PI3KCA wt
AKT1
AKT1/2
PTEN
KRAS wt
MEK1
MEK2
CTNNB1 wt
P53
BAX
78959

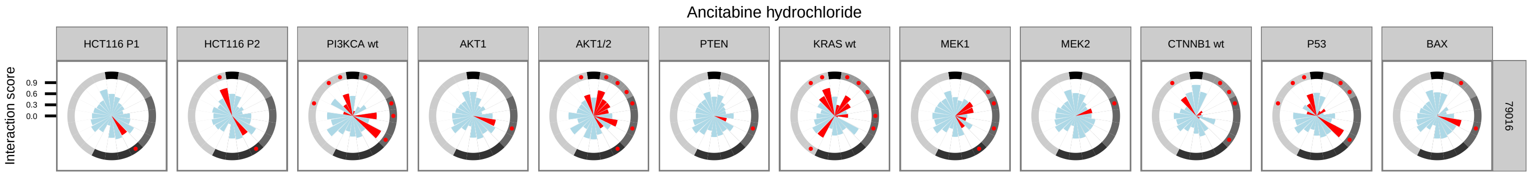
Ancitabine hydrochloride
Interaction score
0.9
0.6
0.3
0.0
HCT116 P1
HCT116 P2
PI3KCA wt
AKT1
AKT1/2
PTEN
KRAS wt
MEK1
MEK2
CTNNB1 wt
P53
BAX
79016

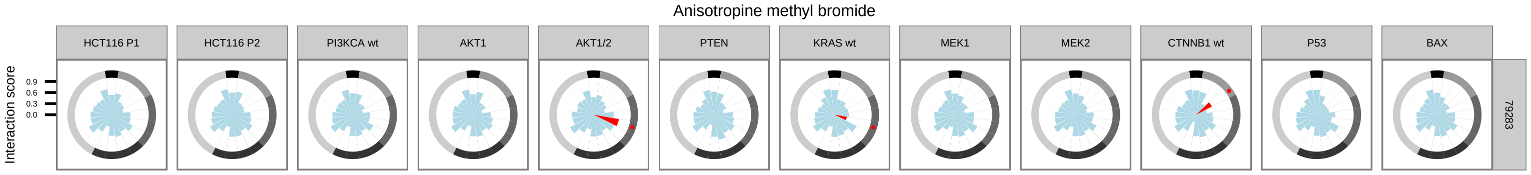
Anisotropine methyl bromide
Interaction score
0.9
0.6
0.3
0.0
HCT116 P1
HCT116 P2
PI3KCA wt
AKT1
AKT1/2
PTEN
KRAS wt
MEK1
MEK2
CTNNB1 wt
P53
BAX
79283

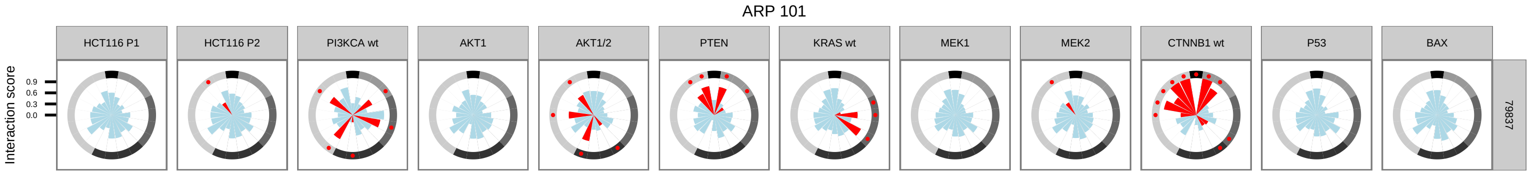
ARP 101
Interaction score
0.9
0.6
0.3
0.0
HCT116 P1
HCT116 P2
PI3KCA wt
AKT1
AKT1/2
PTEN
KRAS wt
MEK1
MEK2
CTNNB1 wt
P53
BAX
79837

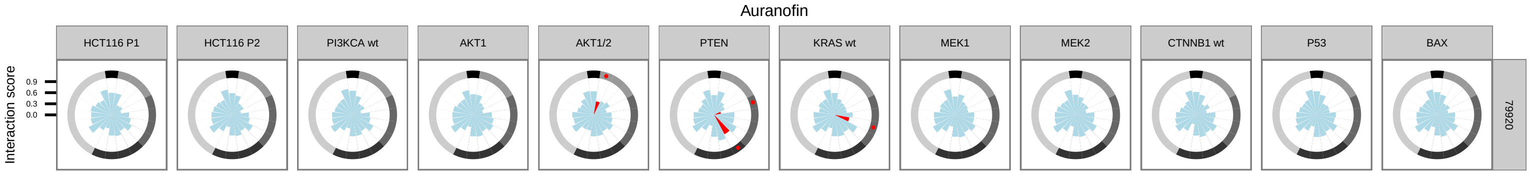
Auranofin
Interaction score
0.9
0.6
0.3
0.0
HCT116 P1
HCT116 P2
PI3KCA wt
AKT1
AKT1/2
PTEN
KRAS wt
MEK1
MEK2
CTNNB1 wt
P53
BAX
79920

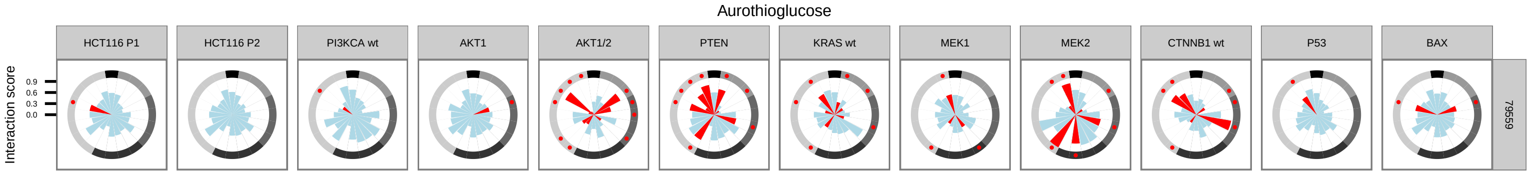
Aurothioglucose
Interaction score
0.9
0.6
0.3
0.0
HCT116 P1
HCT116 P2
PI3KCA wt
AKT1
AKT1/2
PTEN
KRAS wt
MEK1
MEK2
CTNNB1 wt
P53
BAX
79559

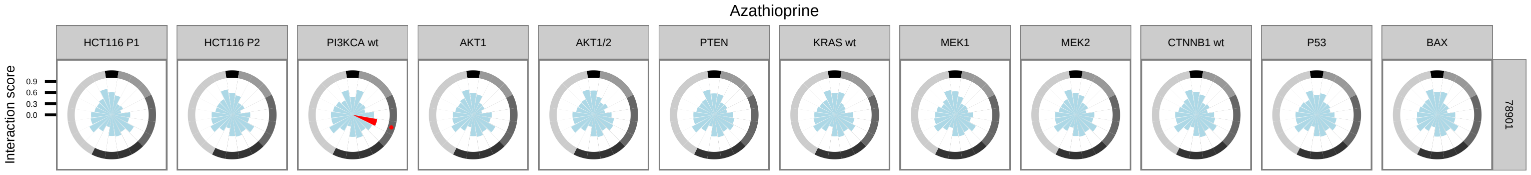
Azathioprine
Interaction score
0.9
0.6
0.3
0.0
HCT116 P1
HCT116 P2
PI3KCA wt
AKT1
AKT1/2
PTEN
KRAS wt
MEK1
MEK2
CTNNB1 wt
P53
BAX
78901

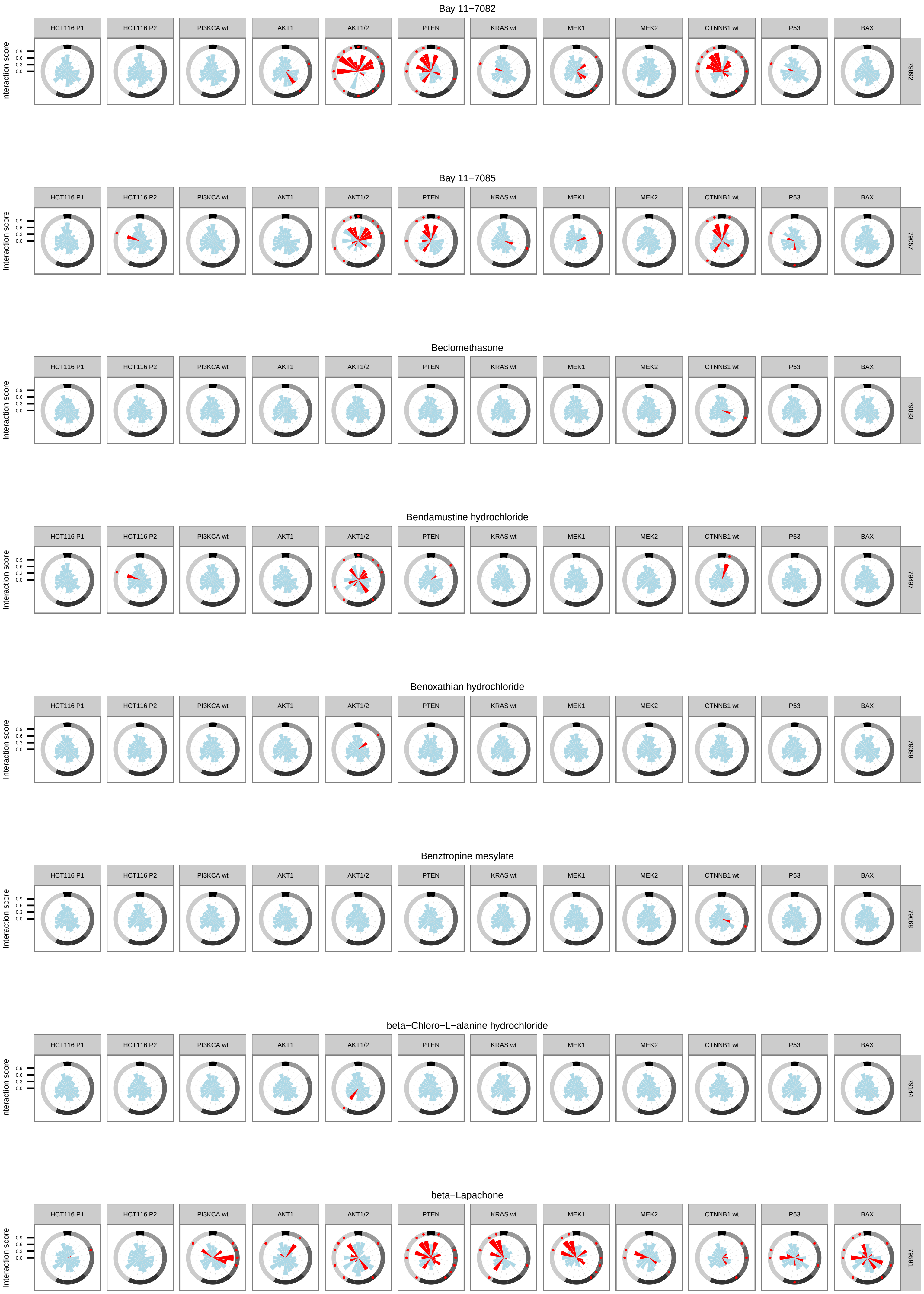
Bay 11−7082
HCT116 P1
HCT116 P2
PI3KCA wt
AKT1
AKT1/2
PTEN
KRAS wt
MEK1
MEK2
CTNNB1 wt
P53
BAX
Interaction score
0.9
0.6
0.3
0.0
79892
Bay 11−7085
79057
Beclomethasone
79033
Bendamustine hydrochloride
79497
Benoxathian hydrochloride
79099
Benztropine mesylate
79068
beta−Chloro−L−alanine hydrochloride
79144
beta−Lapachone
79591

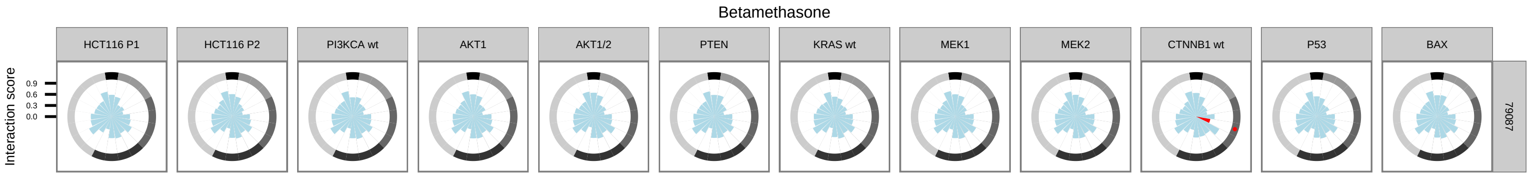
Betamethasone
Interaction score
0.9
0.6
0.3
0.0
HCT116 P1
HCT116 P2
PI3KCA wt
AKT1
AKT1/2
PTEN
KRAS wt
MEK1
MEK2
CTNNB1 wt
P53
BAX
79087

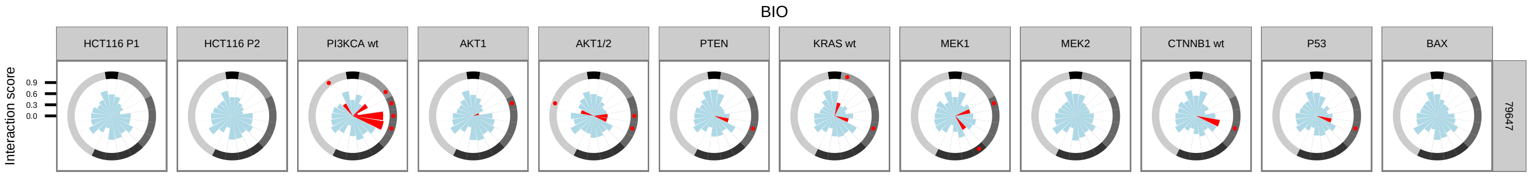
BIO
Interaction score
0.9
0.6
0.3
0.0
HCT116 P1
HCT116 P2
PI3KCA wt
AKT1
AKT1/2
PTEN
KRAS wt
MEK1
MEK2
CTNNB1 wt
P53
BAX
79647

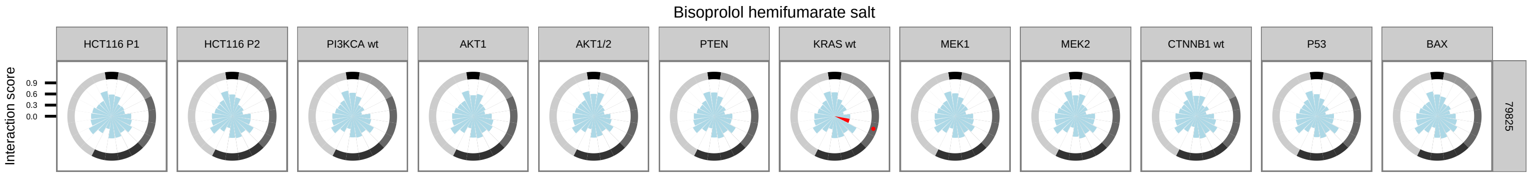
Bisoprolol hemifumarate salt
Interaction score
0.9
0.6
0.3
0.0
HCT116 P1
HCT116 P2
PI3KCA wt
AKT1
AKT1/2
PTEN
KRAS wt
MEK1
MEK2
CTNNB1 wt
P53
BAX
79825

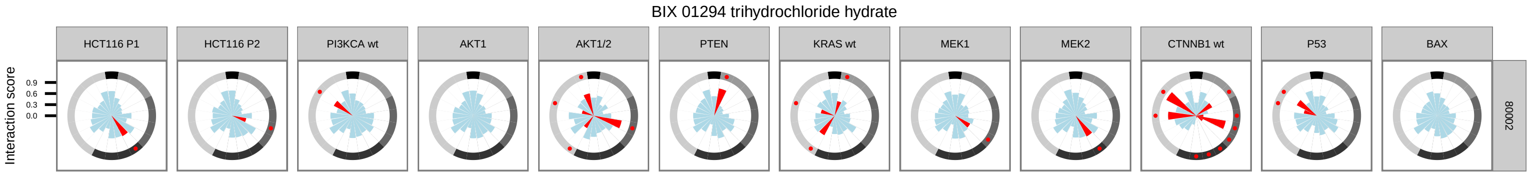
BIX 01294 trihydrochloride hydrate
Interaction score
0.9
0.6
0.3
0.0
HCT116 P1
HCT116 P2
PI3KCA wt
AKT1
AKT1/2
PTEN
KRAS wt
MEK1
MEK2
CTNNB1 wt
P53
BAX
80002

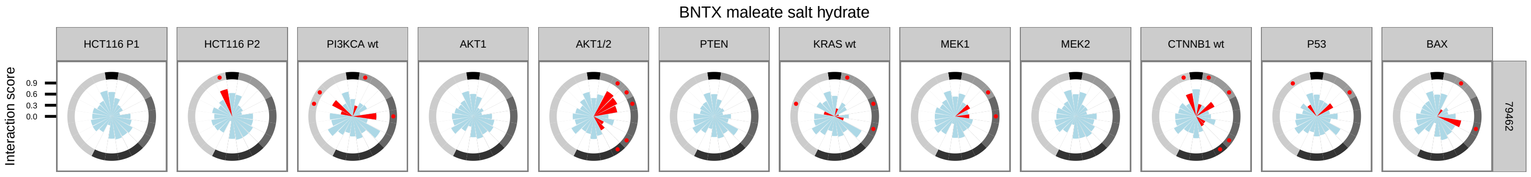
BNTX maleate salt hydrate
Interaction score
0.9
0.6
0.3
0.0
HCT116 P1
HCT116 P2
PI3KCA wt
AKT1
AKT1/2
PTEN
KRAS wt
MEK1
MEK2
CTNNB1 wt
P53
BAX
79462

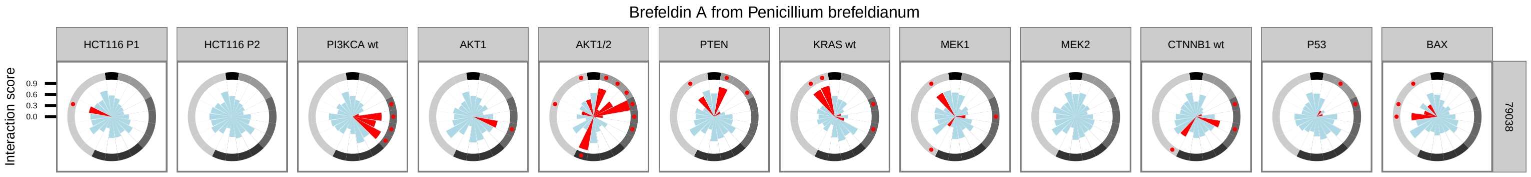
Brefeldin A from Penicillium brefeldianum
Interaction score
0.9
0.6
0.3
0.0
HCT116 P1
HCT116 P2
PI3KCA wt
AKT1
AKT1/2
PTEN
KRAS wt
MEK1
MEK2
CTNNB1 wt
P53
BAX
79038

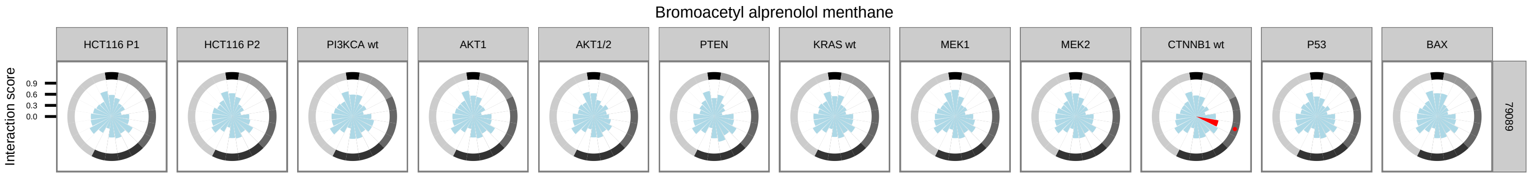
Bromoacetyl alprenolol menthane
Interaction score
0.9
0.6
0.3
0.0
HCT116 P1
HCT116 P2
PI3KCA wt
AKT1
AKT1/2
PTEN
KRAS wt
MEK1
MEK2
CTNNB1 wt
P53
BAX
79089

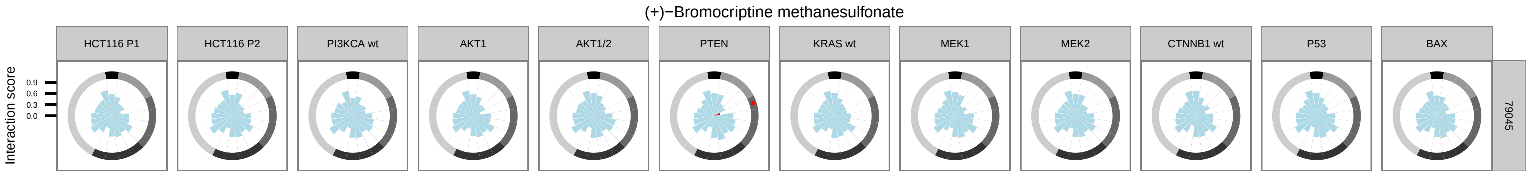
(+)−Bromocriptine methanesulfonate
Interaction score
0.9
0.6
0.3
0.0
HCT116 P1
HCT116 P2
PI3KCA wt
AKT1
AKT1/2
PTEN
KRAS wt
MEK1
MEK2
CTNNB1 wt
P53
BAX
79045

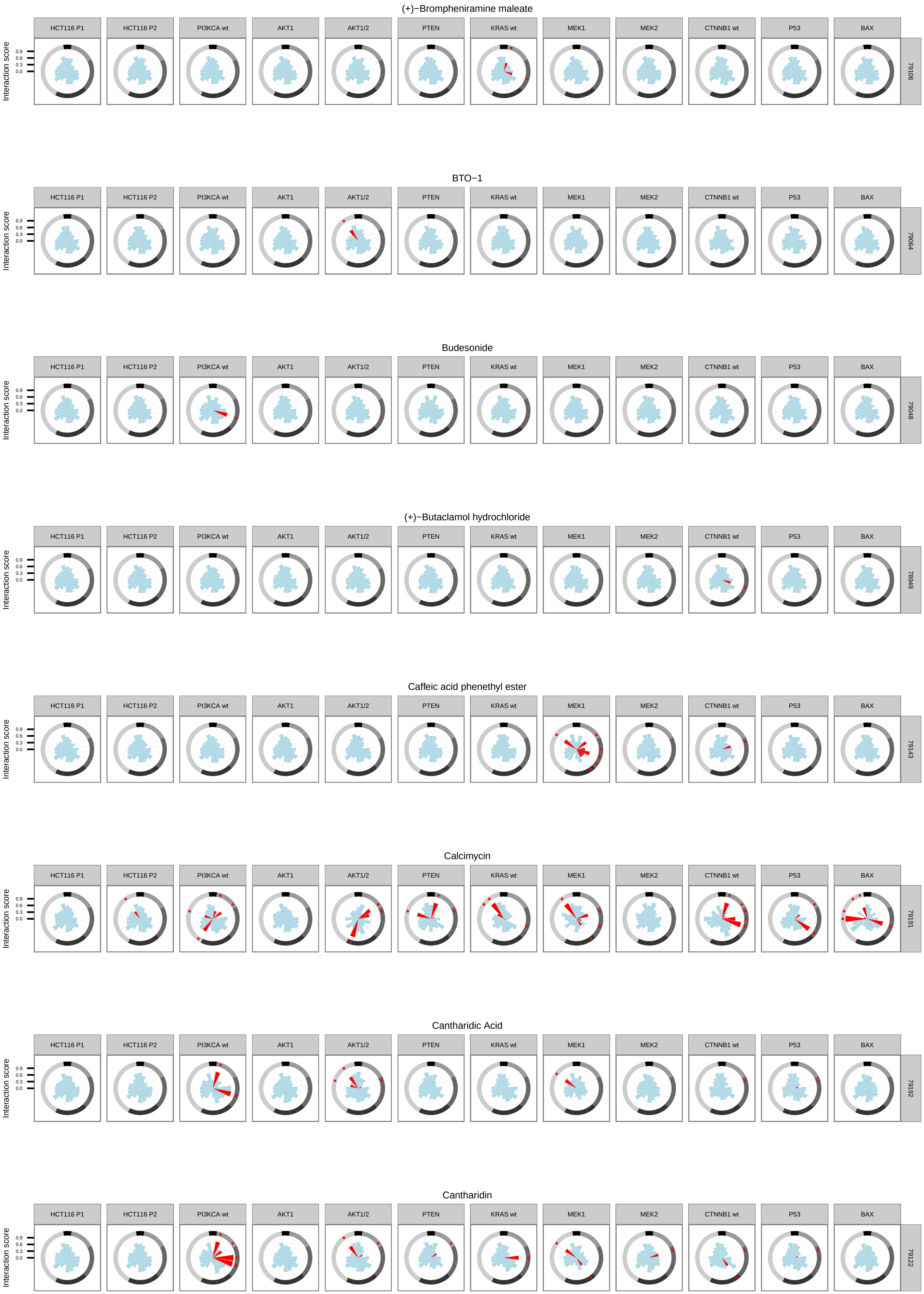

(+)−Brompheniramine maleate
BTO−1
Budesonide
(+)−Butaclamol hydrochloride
Caffeic acid phenethyl ester
Calcimycin
Cantharidic Acid
Cantharidin
HCT116 P1
HCT116 P2
PI3KCA wt
AKT1
AKT1/2
PTEN
KRAS wt
MEK1
MEK2
CTNNB1 wt
P53
BAX
Interaction score
0.9
0.6
0.3
0.0
79106
79064
79048
78849
79143
79191
79192
79122

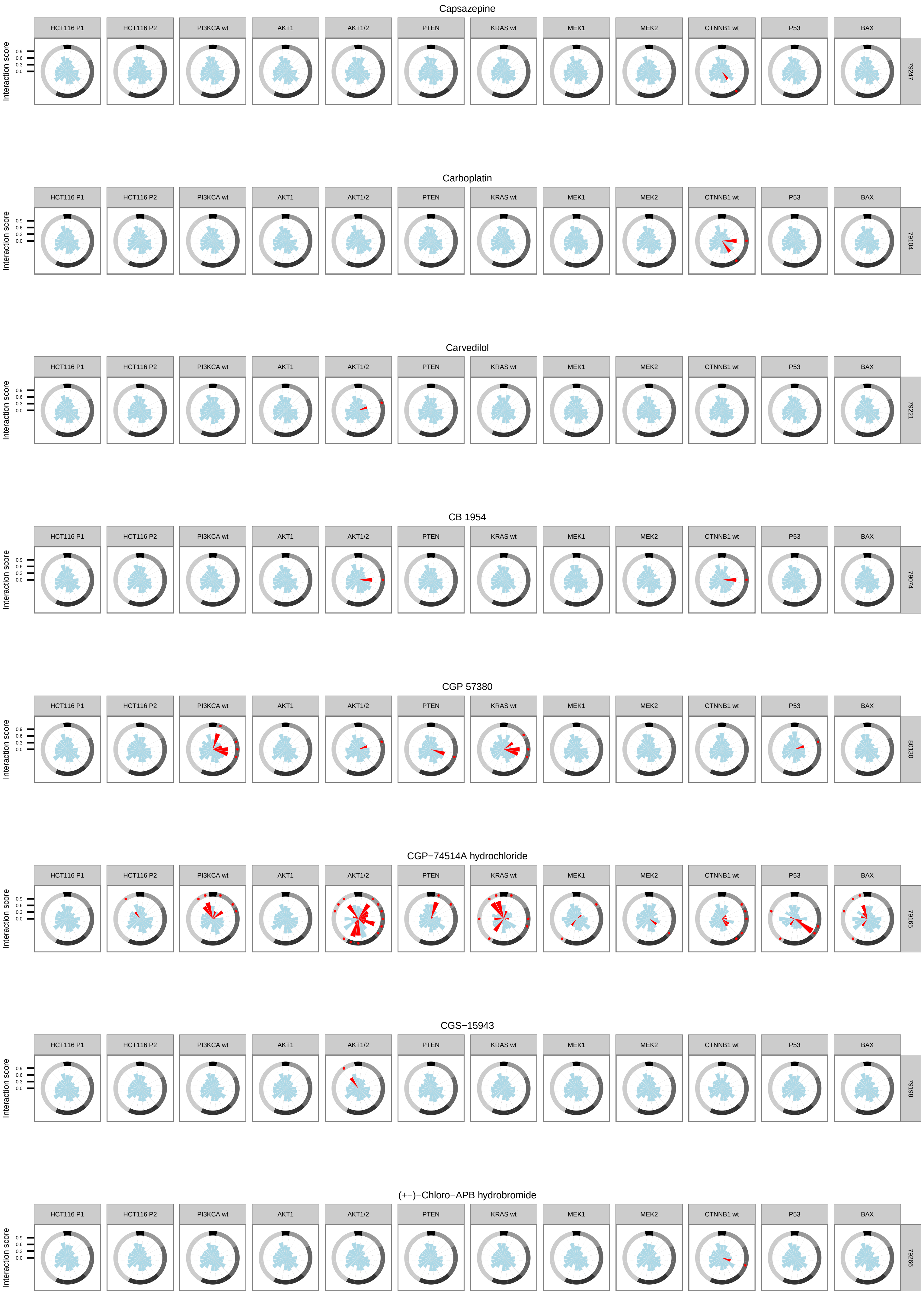
Capsazepine
HCT116 P1
HCT116 P2
PI3KCA wt
AKT1
AKT1/2
PTEN
KRAS wt
MEK1
MEK2
CTNNB1 wt
P53
BAX
79247
Interaction score
0.9
0.6
0.3
0.0
Carboplatin
HCT116 P1
HCT116 P2
PI3KCA wt
AKT1
AKT1/2
PTEN
KRAS wt
MEK1
MEK2
CTNNB1 wt
P53
BAX
79104
Interaction score
0.9
0.6
0.3
0.0
Carvedilol
HCT116 P1
HCT116 P2
PI3KCA wt
AKT1
AKT1/2
PTEN
KRAS wt
MEK1
MEK2
CTNNB1 wt
P53
BAX
79221
Interaction score
0.9
0.6
0.3
0.0
CB 1954
HCT116 P1
HCT116 P2
PI3KCA wt
AKT1
AKT1/2
PTEN
KRAS wt
MEK1
MEK2
CTNNB1 wt
P53
BAX
79074
Interaction score
0.9
0.6
0.3
0.0
CGP 57380
HCT116 P1
HCT116 P2
PI3KCA wt
AKT1
AKT1/2
PTEN
KRAS wt
MEK1
MEK2
CTNNB1 wt
P53
BAX
80130
Interaction score
0.9
0.6
0.3
0.0
CGP−74514A hydrochloride
HCT116 P1
HCT116 P2
PI3KCA wt
AKT1
AKT1/2
PTEN
KRAS wt
MEK1
MEK2
CTNNB1 wt
P53
BAX
79165
Interaction score
0.9
0.6
0.3
0.0
CGS−15943
HCT116 P1
HCT116 P2
PI3KCA wt
AKT1
AKT1/2
PTEN
KRAS wt
MEK1
MEK2
CTNNB1 wt
P53
BAX
79198
Interaction score
0.9
0.6
0.3
0.0
(+−)−Chloro−APB hydrobromide
HCT116 P1
HCT116 P2
PI3KCA wt
AKT1
AKT1/2
PTEN
KRAS wt
MEK1
MEK2
CTNNB1 wt
P53
BAX
79266
Interaction score
0.9
0.6
0.3
0.0

CHM−1 hydrate

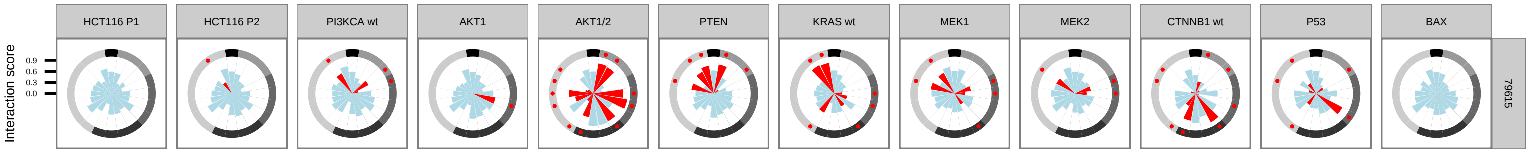

cis−Azetidine−2,4−dicarboxylic acid

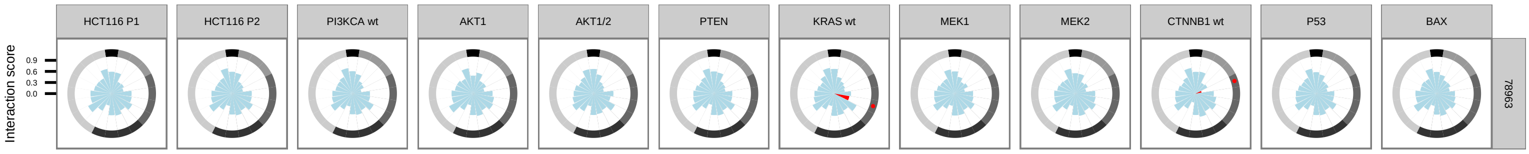

CK2 Inhibitor 2

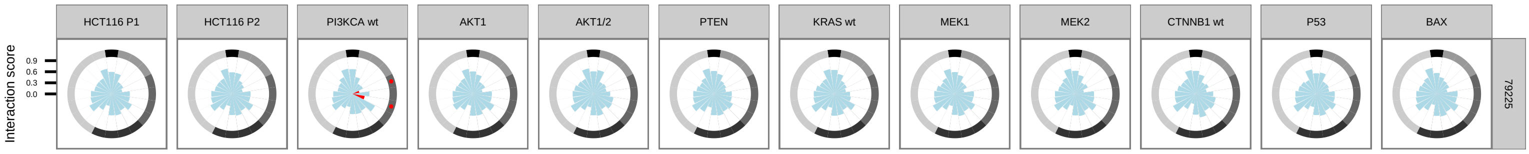

Clemastine fumarate

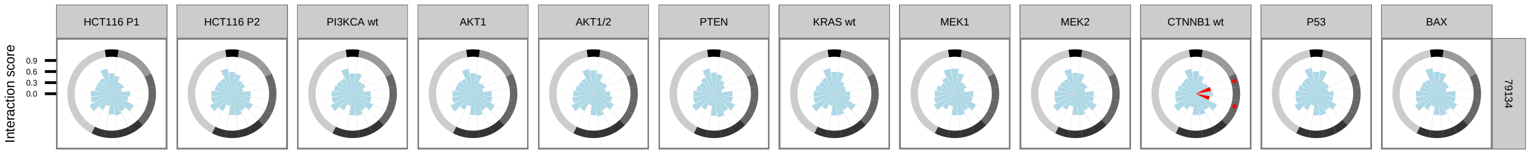

Colchicine

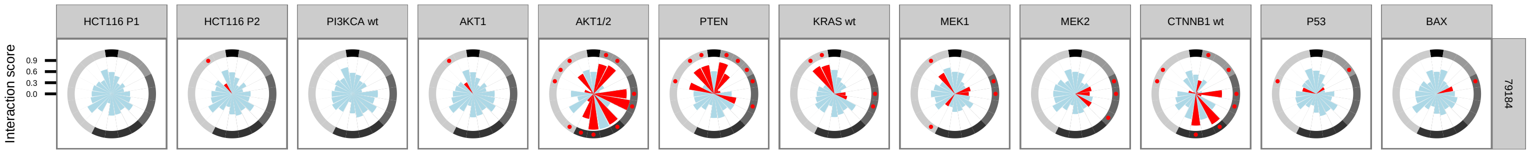

Cyclosporin A

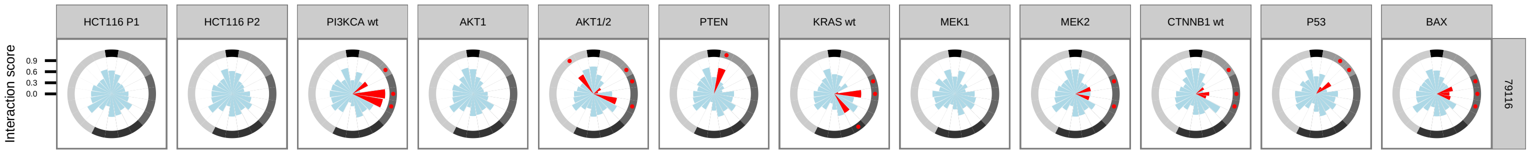

Cytosine−1−beta−D−arabinofuranoside hydrochloride

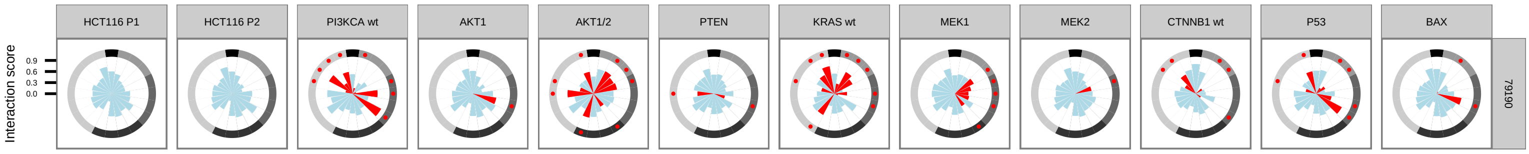

DAPH

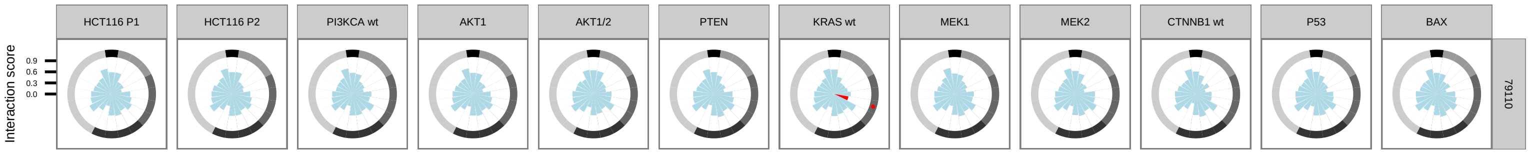

Debrisoquin sulfate

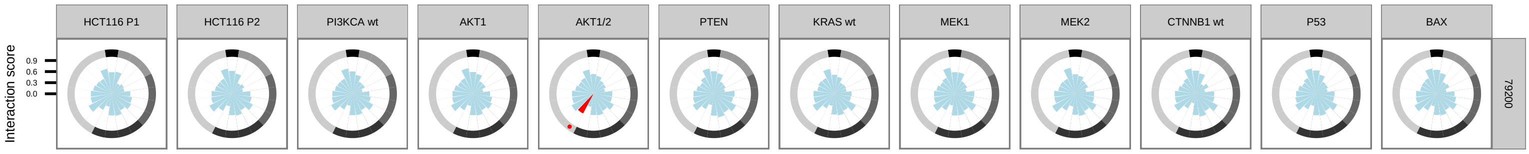

Dequalinium chloride hydrate

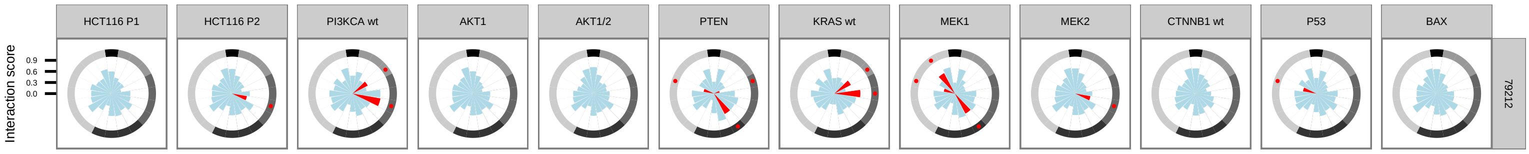

Dihydroouabain

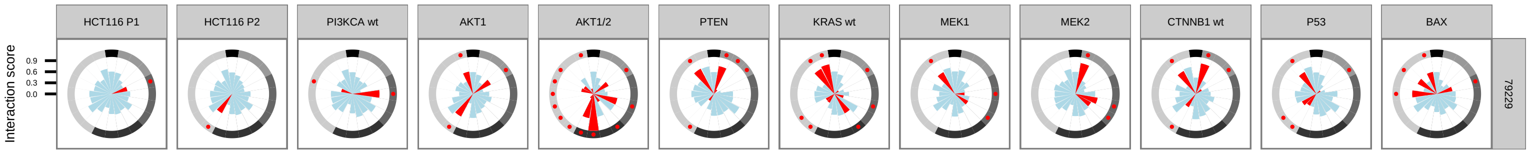

Diphenyleneiodonium chloride

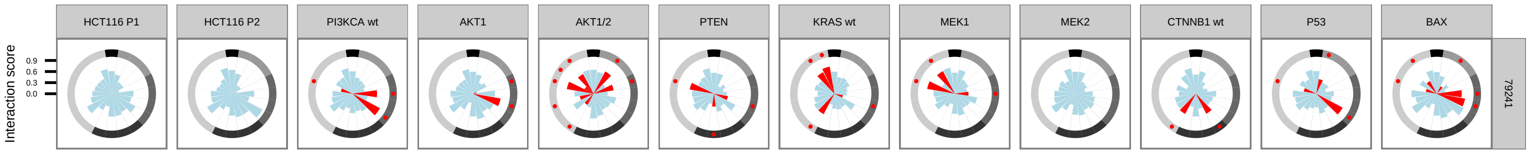

DL−alpha−Methyl−p−tyrosine

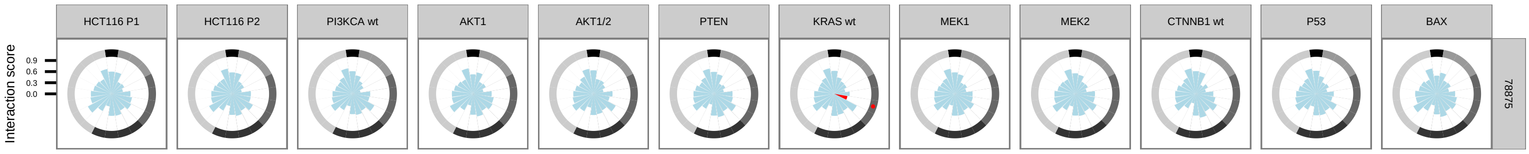

DL−Stearoylcarnitine chloride

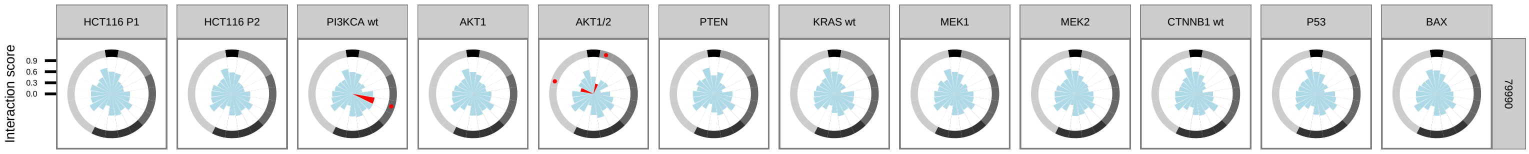

Domperidone

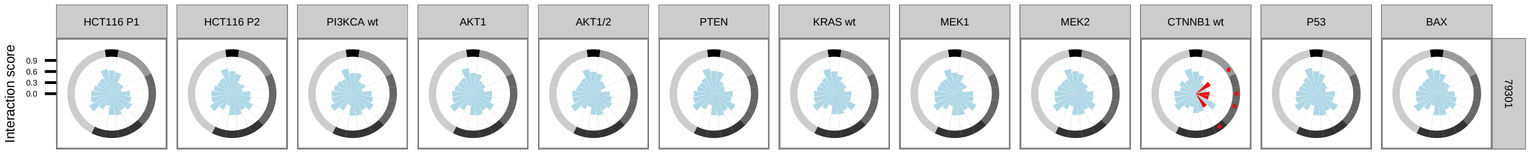

Droperidol

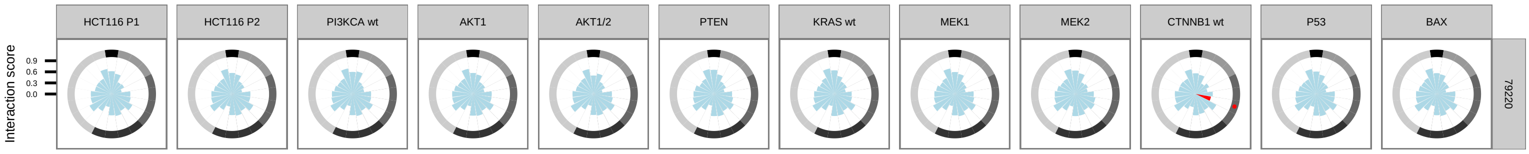

(E)−5−(2−Bromovinyl)−2'−deoxyuridine

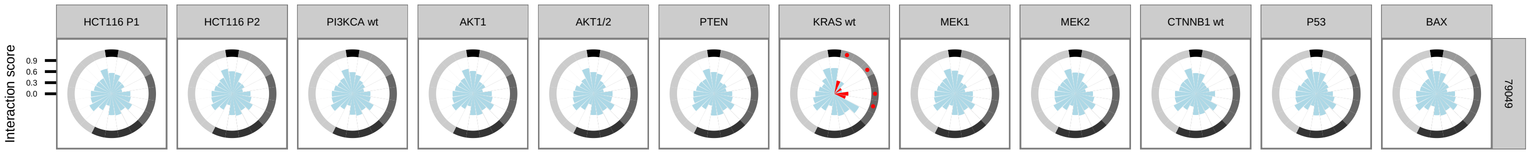

EGTA

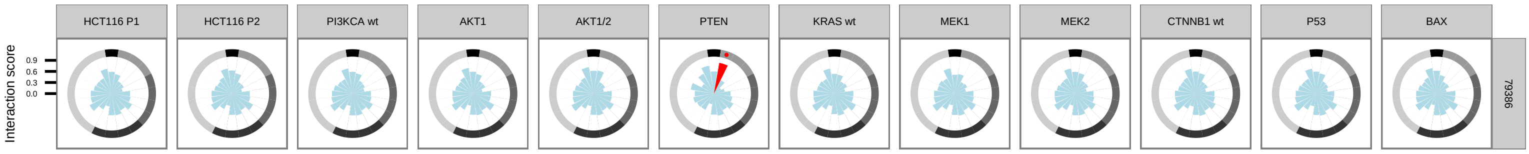

Ellipticine

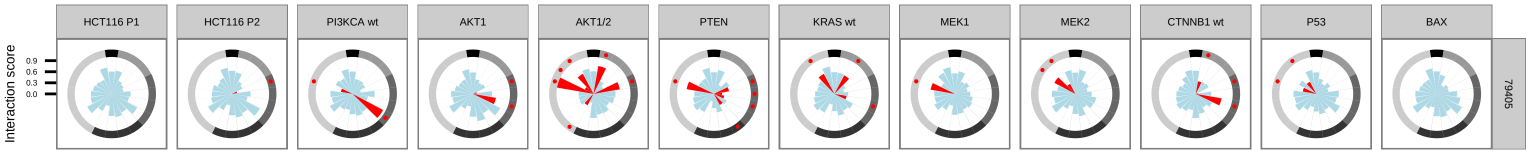

Emetine dihydrochloride hydrate

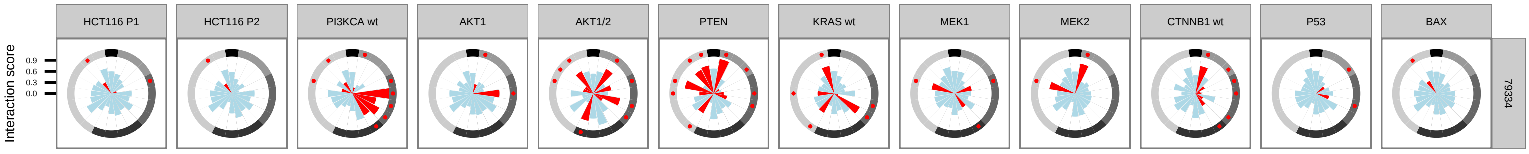

Enalaprilat dihydrate

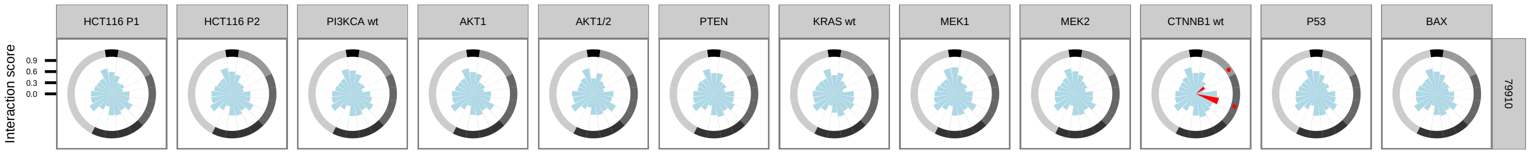

ET−18−OCH3

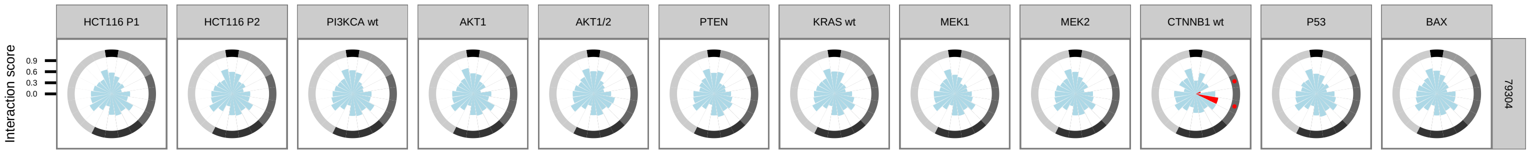

Ethopropazine hydrochloride

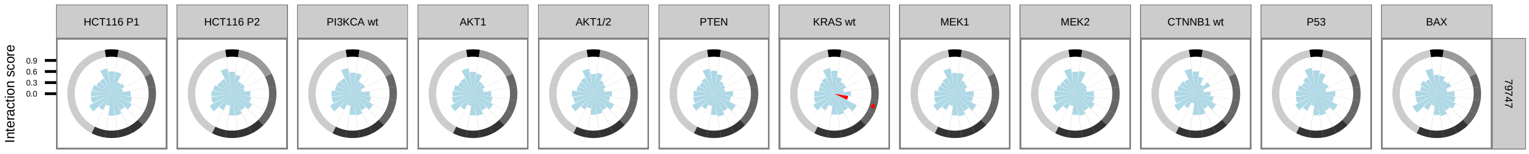

Etoposide

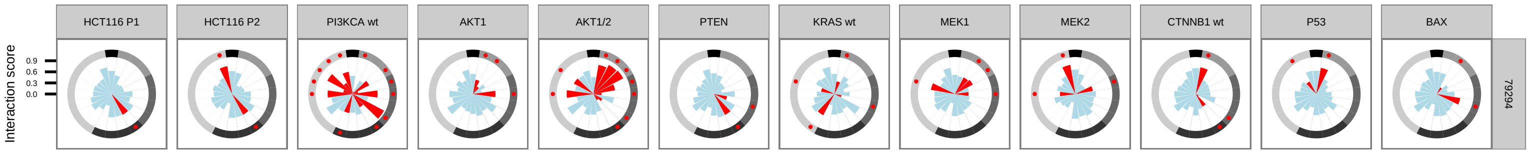

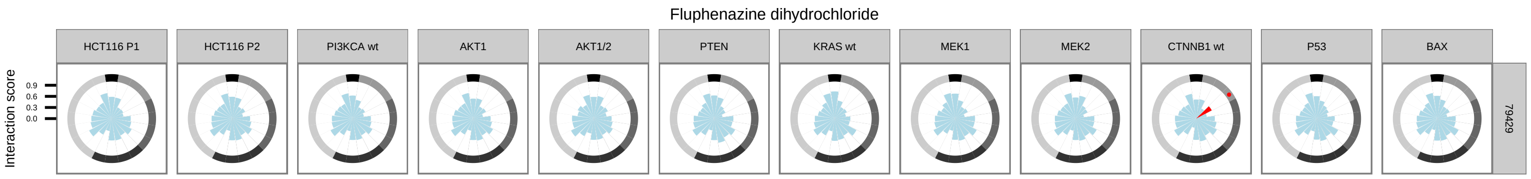
Fluphenazine dihydrochloride
HCT116 P1
HCT116 P2
PI3KCA wt
AKT1
AKT1/2
PTEN
KRAS wt
MEK1
MEK2
CTNNB1 wt
P53
BAX
Interaction score
0.9
0.6
0.3
0.0
79429

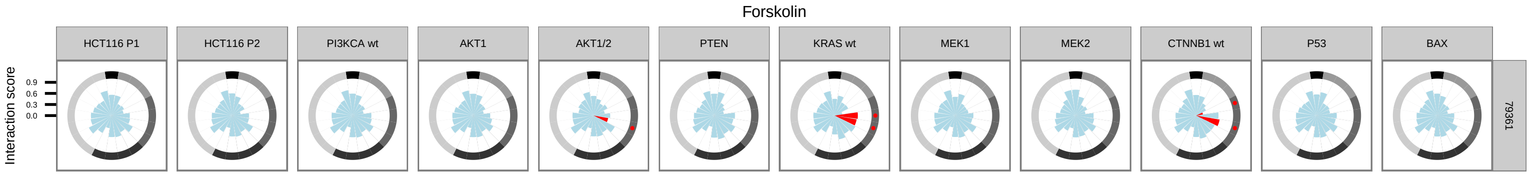
Forskolin
HCT116 P1
HCT116 P2
PI3KCA wt
AKT1
AKT1/2
PTEN
KRAS wt
MEK1
MEK2
CTNNB1 wt
P53
BAX
Interaction score
0.9
0.6
0.3
0.0
79361

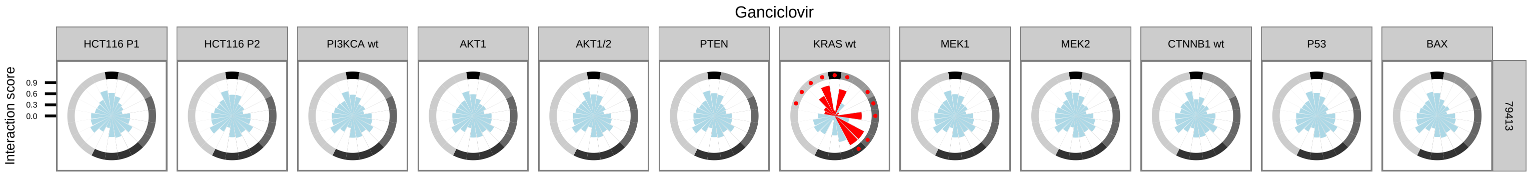
Ganciclovir
HCT116 P1
HCT116 P2
PI3KCA wt
AKT1
AKT1/2
PTEN
KRAS wt
MEK1
MEK2
CTNNB1 wt
P53
BAX
Interaction score
0.9
0.6
0.3
0.0
79413

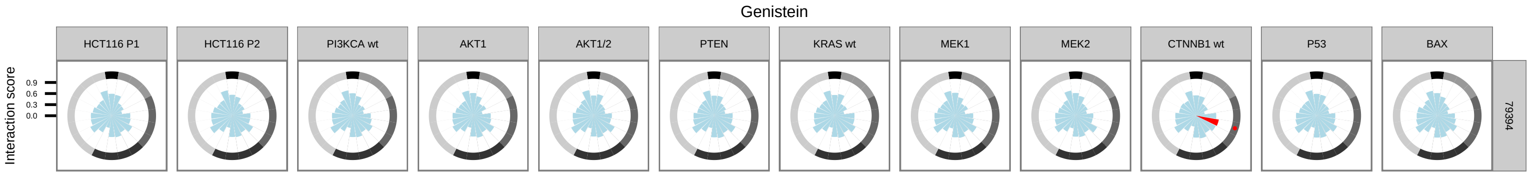
Genistein
HCT116 P1
HCT116 P2
PI3KCA wt
AKT1
AKT1/2
PTEN
KRAS wt
MEK1
MEK2
CTNNB1 wt
P53
BAX
Interaction score
0.9
0.6
0.3
0.0
79394

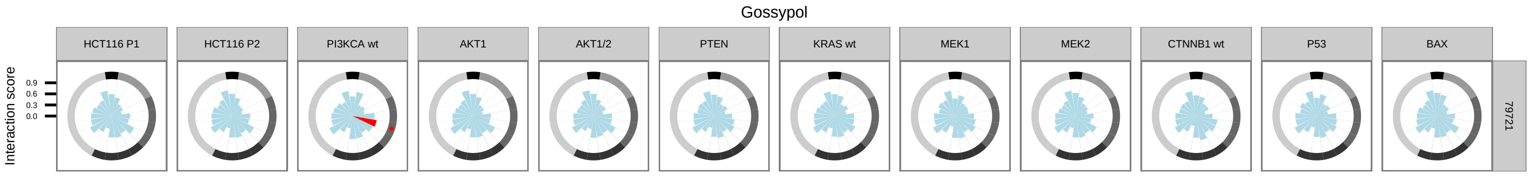
Gossypol
HCT116 P1
HCT116 P2
PI3KCA wt
AKT1
AKT1/2
PTEN
KRAS wt
MEK1
MEK2
CTNNB1 wt
P53
BAX
Interaction score
0.9
0.6
0.3
0.0
79721

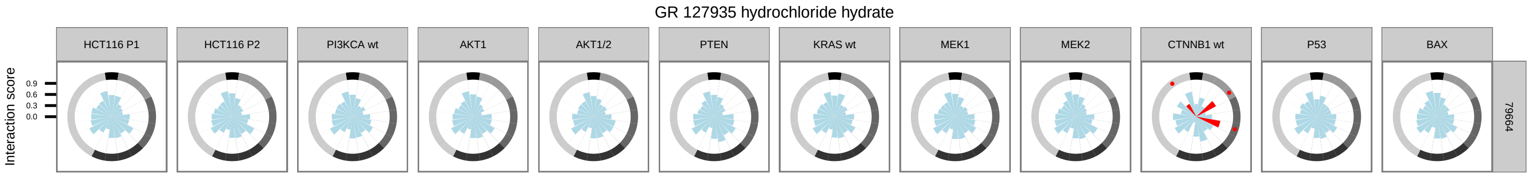
GR 127935 hydrochloride hydrate
HCT116 P1
HCT116 P2
PI3KCA wt
AKT1
AKT1/2
PTEN
KRAS wt
MEK1
MEK2
CTNNB1 wt
P53
BAX
Interaction score
0.9
0.6
0.3
0.0
79664

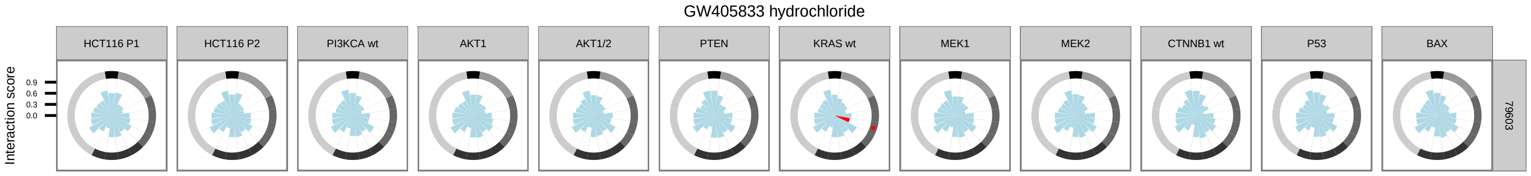
GW405833 hydrochloride
HCT116 P1
HCT116 P2
PI3KCA wt
AKT1
AKT1/2
PTEN
KRAS wt
MEK1
MEK2
CTNNB1 wt
P53
BAX
Interaction score
0.9
0.6
0.3
0.0
79603

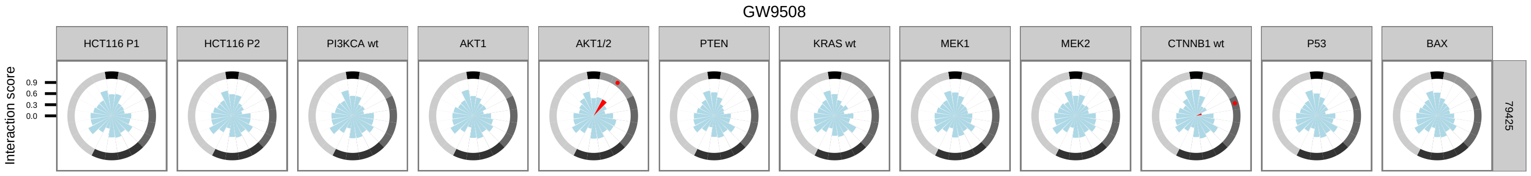
GW9508
HCT116 P1
HCT116 P2
PI3KCA wt
AKT1
AKT1/2
PTEN
KRAS wt
MEK1
MEK2
CTNNB1 wt
P53
BAX
Interaction score
0.9
0.6
0.3
0.0
79425

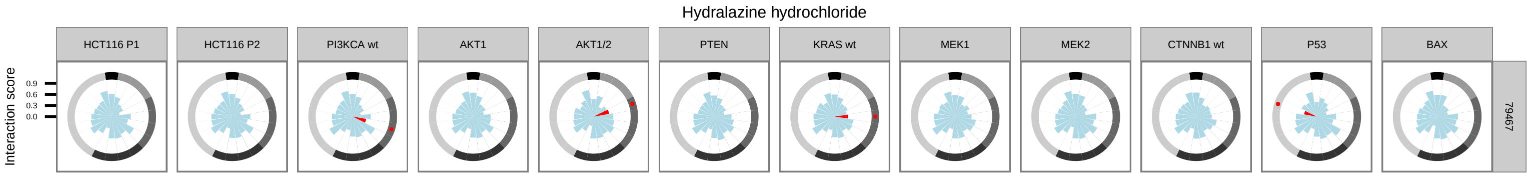
Hydralazine hydrochloride
HCT116 P1
HCT116 P2
PI3KCA wt
AKT1
AKT1/2
PTEN
KRAS wt
MEK1
MEK2
CTNNB1 wt
P53
BAX
79467
Interaction score
0.9
0.6
0.3
0.0

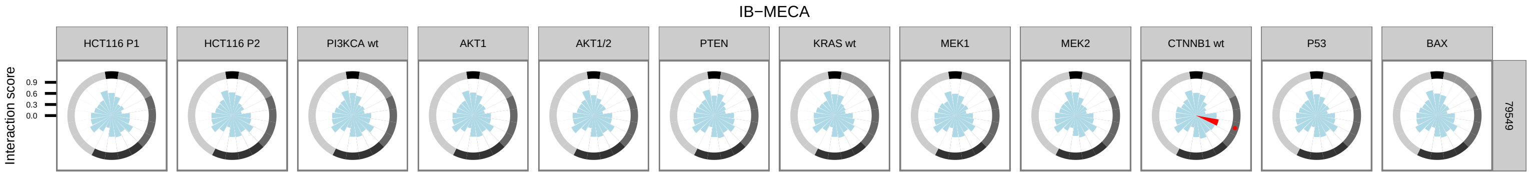
IB−MECA
HCT116 P1
HCT116 P2
PI3KCA wt
AKT1
AKT1/2
PTEN
KRAS wt
MEK1
MEK2
CTNNB1 wt
P53
BAX
79549
Interaction score
0.9
0.6
0.3
0.0

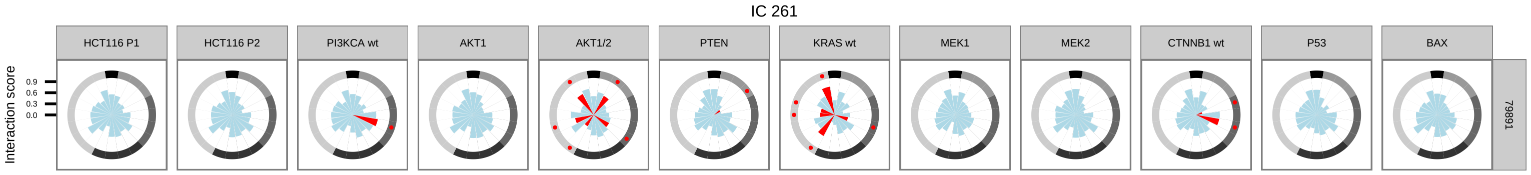
IC 261
HCT116 P1
HCT116 P2
PI3KCA wt
AKT1
AKT1/2
PTEN
KRAS wt
MEK1
MEK2
CTNNB1 wt
P53
BAX
79891
Interaction score
0.9
0.6
0.3
0.0

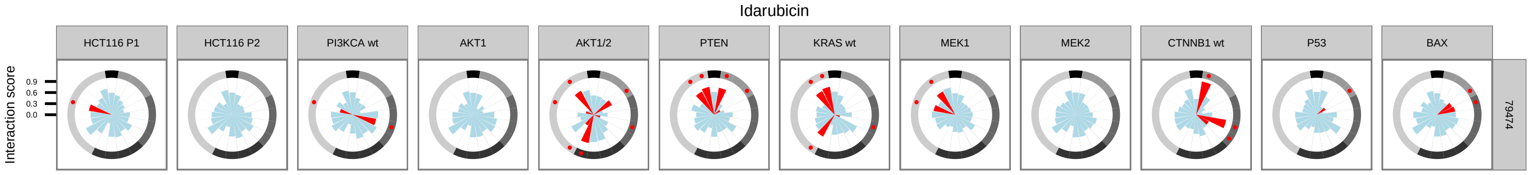
Idarubicin
HCT116 P1
HCT116 P2
PI3KCA wt
AKT1
AKT1/2
PTEN
KRAS wt
MEK1
MEK2
CTNNB1 wt
P53
BAX
79474
Interaction score
0.9
0.6
0.3
0.0

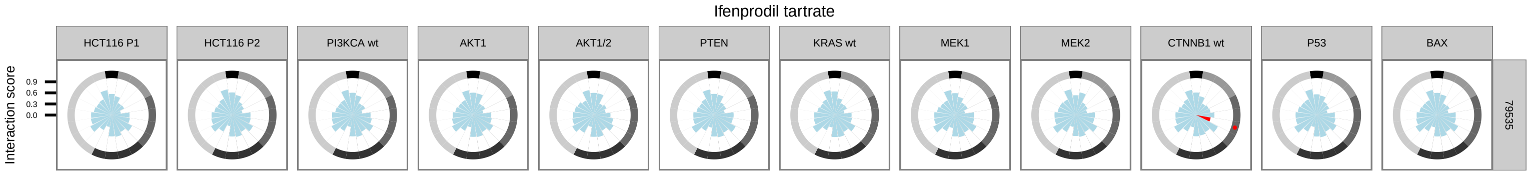
Ifenprodil tartrate
HCT116 P1
HCT116 P2
PI3KCA wt
AKT1
AKT1/2
PTEN
KRAS wt
MEK1
MEK2
CTNNB1 wt
P53
BAX
79535
Interaction score
0.9
0.6
0.3
0.0

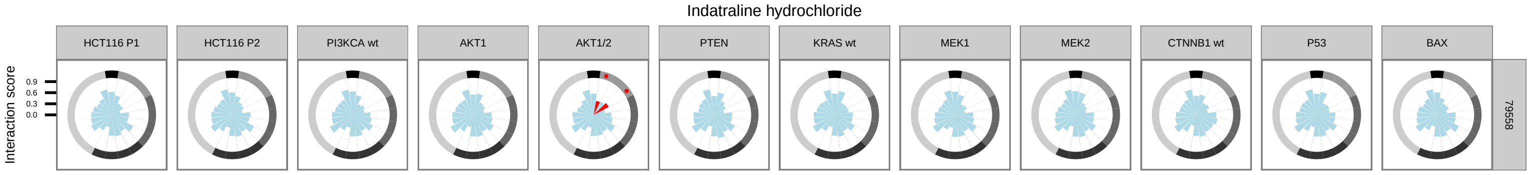
Indatraline hydrochloride
HCT116 P1
HCT116 P2
PI3KCA wt
AKT1
AKT1/2
PTEN
KRAS wt
MEK1
MEK2
CTNNB1 wt
P53
BAX
79558
Interaction score
0.9
0.6
0.3
0.0

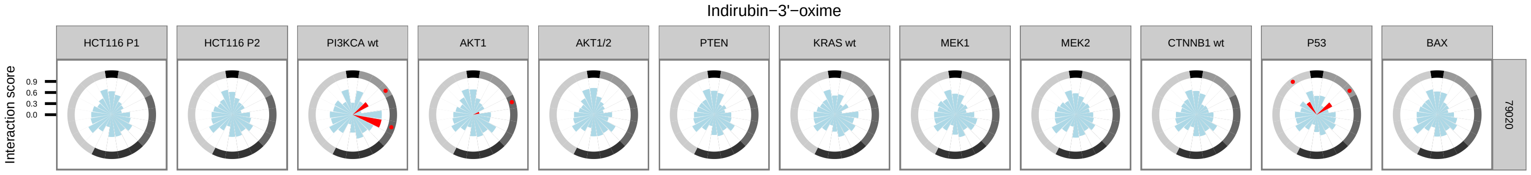
Indirubin−3'−oxime
HCT116 P1
HCT116 P2
PI3KCA wt
AKT1
AKT1/2
PTEN
KRAS wt
MEK1
MEK2
CTNNB1 wt
P53
BAX
79020
Interaction score
0.9
0.6
0.3
0.0

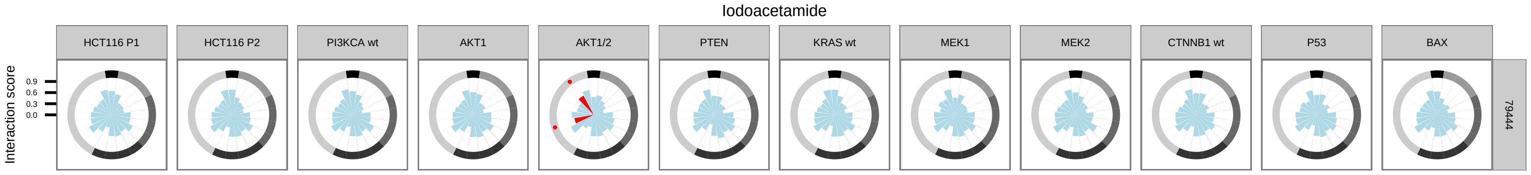
Iodoacetamide
HCT116 P1
HCT116 P2
PI3KCA wt
AKT1
AKT1/2
PTEN
KRAS wt
MEK1
MEK2
CTNNB1 wt
P53
BAX
79444
Interaction score
0.9
0.6
0.3
0.0

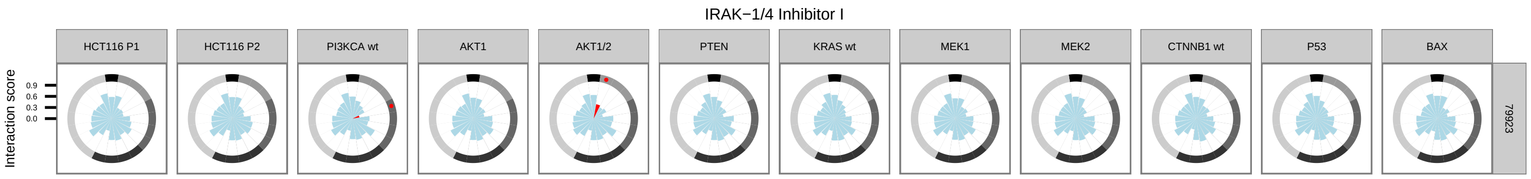
IRAK−1/4 Inhibitor I
HCT116 P1
HCT116 P2
PI3KCA wt
AKT1
AKT1/2
PTEN
KRAS wt
MEK1
MEK2
CTNNB1 wt
P53
BAX
Interaction score
0.9
0.6
0.3
0.0
79923

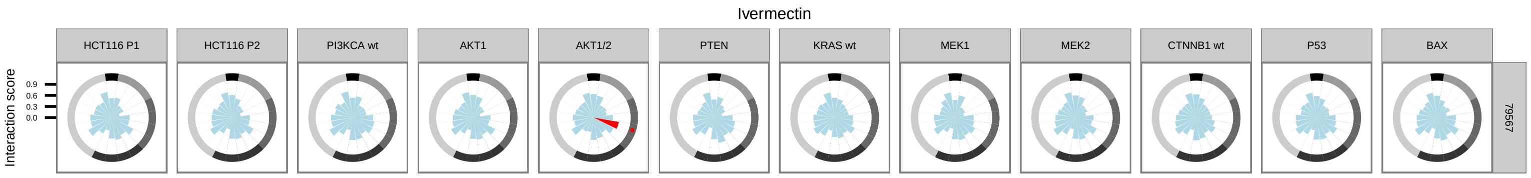
Ivermectin
HCT116 P1
HCT116 P2
PI3KCA wt
AKT1
AKT1/2
PTEN
KRAS wt
MEK1
MEK2
CTNNB1 wt
P53
BAX
Interaction score
0.9
0.6
0.3
0.0
79567

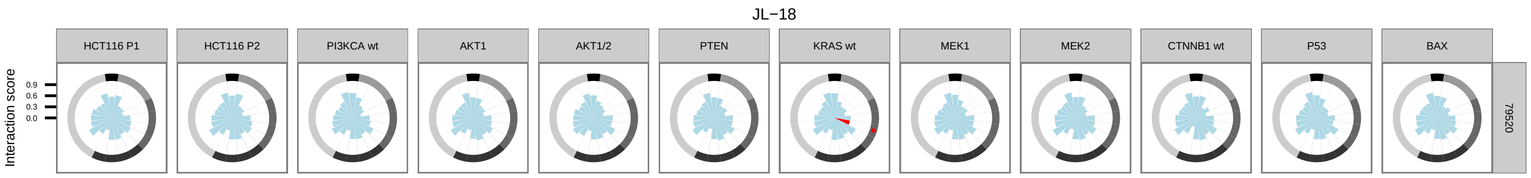
JL−18
HCT116 P1
HCT116 P2
PI3KCA wt
AKT1
AKT1/2
PTEN
KRAS wt
MEK1
MEK2
CTNNB1 wt
P53
BAX
Interaction score
0.9
0.6
0.3
0.0
79520

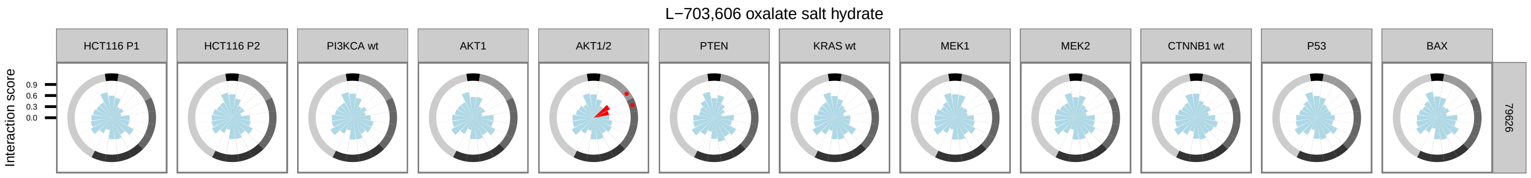
L−703,606 oxalate salt hydrate
HCT116 P1
HCT116 P2
PI3KCA wt
AKT1
AKT1/2
PTEN
KRAS wt
MEK1
MEK2
CTNNB1 wt
P53
BAX
Interaction score
0.9
0.6
0.3
0.0
79626

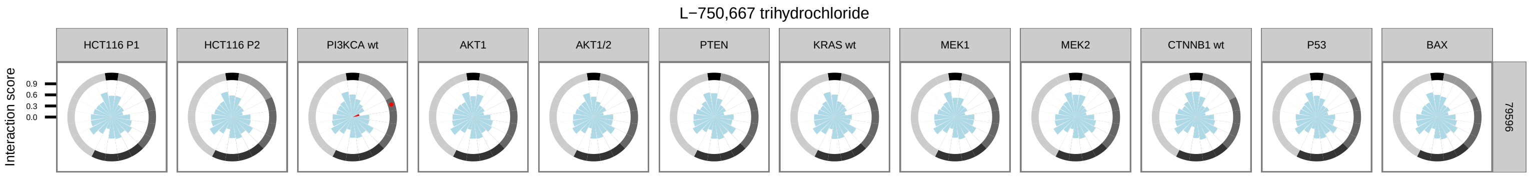
L−750,667 trihydrochloride
HCT116 P1
HCT116 P2
PI3KCA wt
AKT1
AKT1/2
PTEN
KRAS wt
MEK1
MEK2
CTNNB1 wt
P53
BAX
Interaction score
0.9
0.6
0.3
0.0
79596

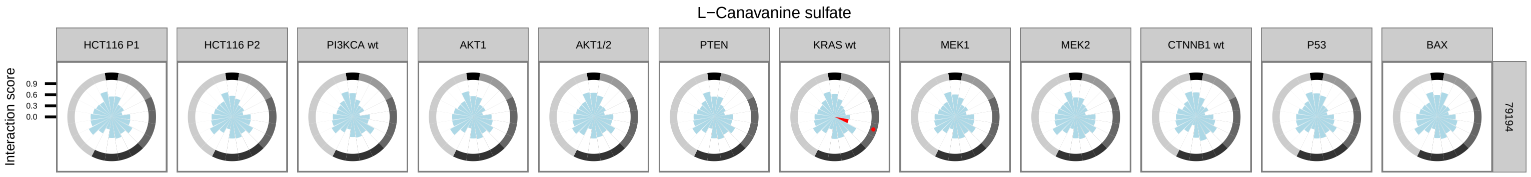
L−Canavanine sulfate
HCT116 P1
HCT116 P2
PI3KCA wt
AKT1
AKT1/2
PTEN
KRAS wt
MEK1
MEK2
CTNNB1 wt
P53
BAX
Interaction score
0.9
0.6
0.3
0.0
79194

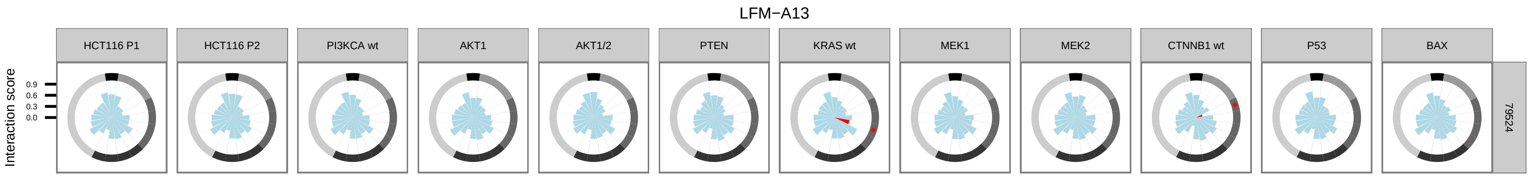
LFM−A13
HCT116 P1
HCT116 P2
PI3KCA wt
AKT1
AKT1/2
PTEN
KRAS wt
MEK1
MEK2
CTNNB1 wt
P53
BAX
Interaction score
0.9
0.6
0.3
0.0
79524

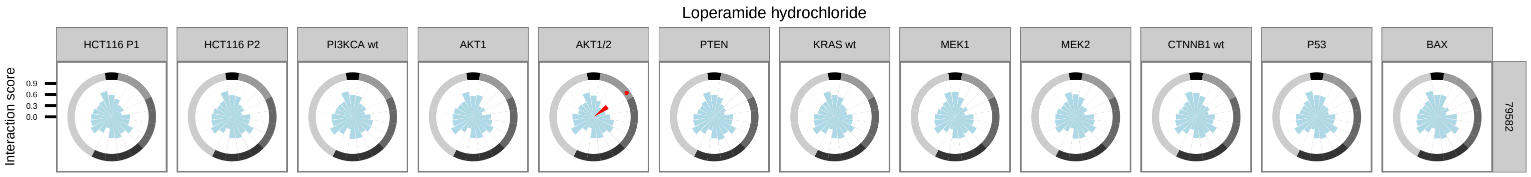
Loperamide hydrochloride
HCT116 P1
HCT116 P2
PI3KCA wt
AKT1
AKT1/2
PTEN
KRAS wt
MEK1
MEK2
CTNNB1 wt
P53
BAX
Interaction score
0.9
0.6
0.3
0.0
79582

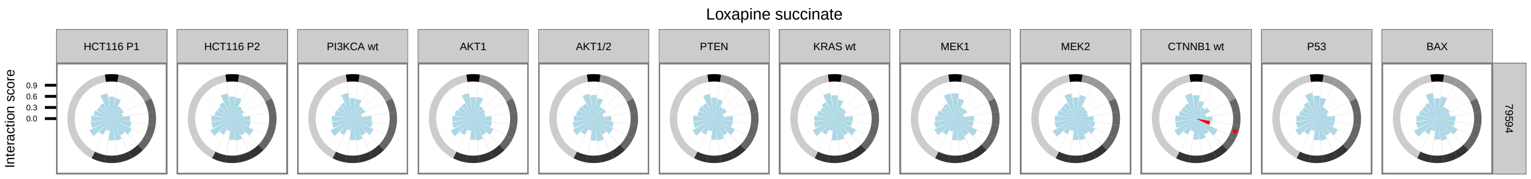
Loxapine succinate
HCT116 P1
HCT116 P2
PI3KCA wt
AKT1
AKT1/2
PTEN
KRAS wt
MEK1
MEK2
CTNNB1 wt
P53
BAX
Interaction score
0.9
0.6
0.3
0.0
79594

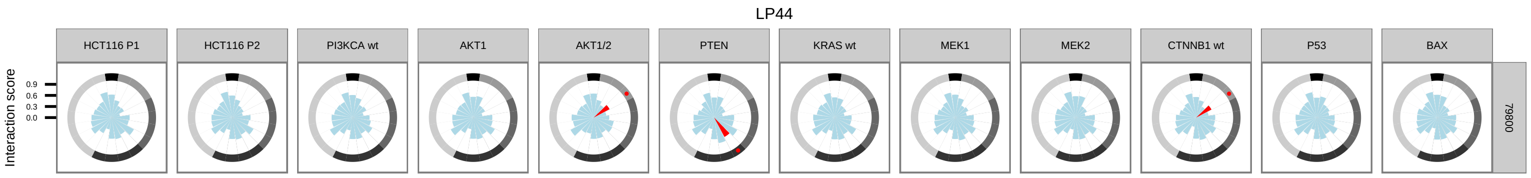
LP44
HCT116 P1
HCT116 P2
PI3KCA wt
AKT1
AKT1/2
PTEN
KRAS wt
MEK1
MEK2
CTNNB1 wt
P53
BAX
Interaction score
0.9
0.6
0.3
0.0
79800

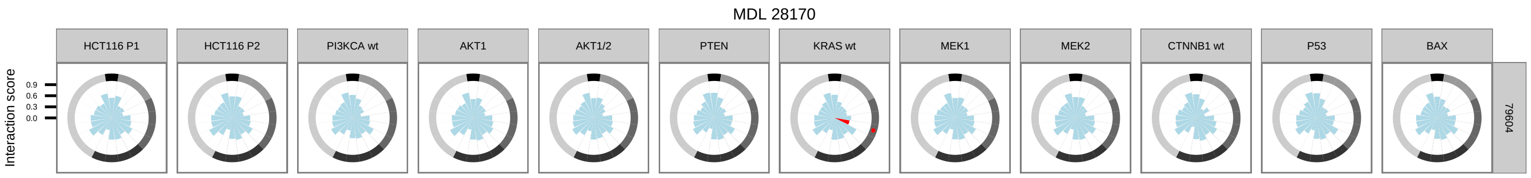
MDL 28170
HCT116 P1
HCT116 P2
PI3KCA wt
AKT1
AKT1/2
PTEN
KRAS wt
MEK1
MEK2
CTNNB1 wt
P53
BAX
Interaction score
0.9
0.6
0.3
0.0
79604

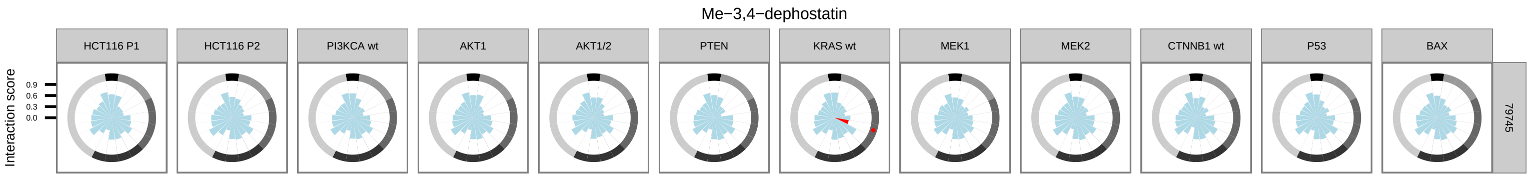
Me−3,4−dephostatin
HCT116 P1
HCT116 P2
PI3KCA wt
AKT1
AKT1/2
PTEN
KRAS wt
MEK1
MEK2
CTNNB1 wt
P53
BAX
Interaction score
0.9
0.6
0.3
0.0
79745

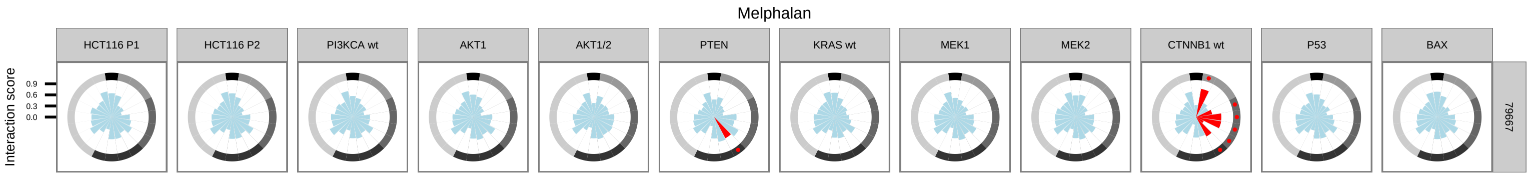
Melphalan
HCT116 P1
HCT116 P2
PI3KCA wt
AKT1
AKT1/2
PTEN
KRAS wt
MEK1
MEK2
CTNNB1 wt
P53
BAX
Interaction score
0.9
0.6
0.3
0.0
79667

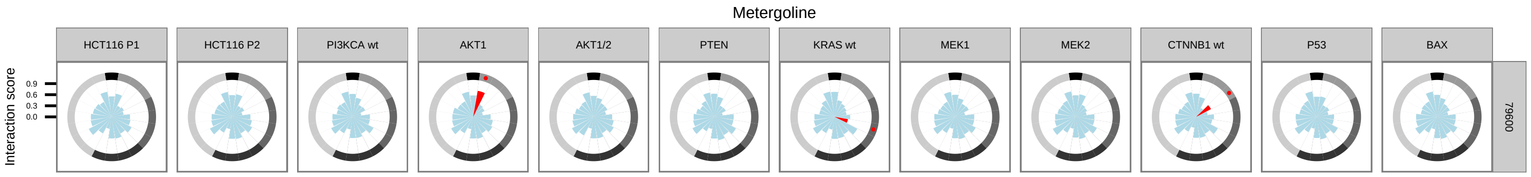
Metergoline
HCT116 P1
HCT116 P2
PI3KCA wt
AKT1
AKT1/2
PTEN
KRAS wt
MEK1
MEK2
CTNNB1 wt
P53
BAX
Interaction score
0.9
0.6
0.3
0.0
79600

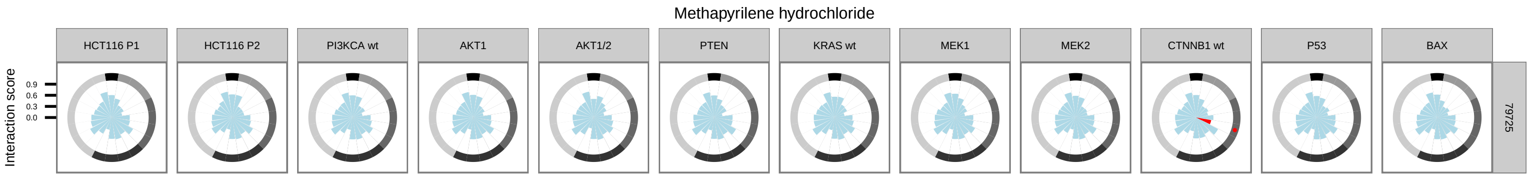
Methapyrilene hydrochloride
HCT116 P1
HCT116 P2
PI3KCA wt
AKT1
AKT1/2
PTEN
KRAS wt
MEK1
MEK2
CTNNB1 wt
P53
BAX
Interaction score
0.9
0.6
0.3
0.0
79725

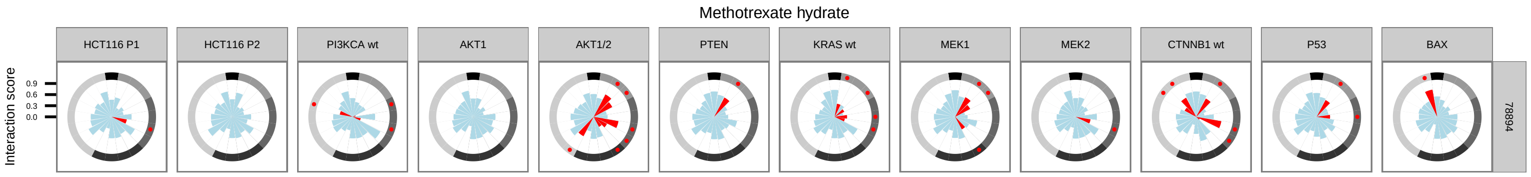
Methotrexate hydrate
HCT116 P1
HCT116 P2
PI3KCA wt
AKT1
AKT1/2
PTEN
KRAS wt
MEK1
MEK2
CTNNB1 wt
P53
BAX
Interaction score
0.9
0.6
0.3
0.0
78894

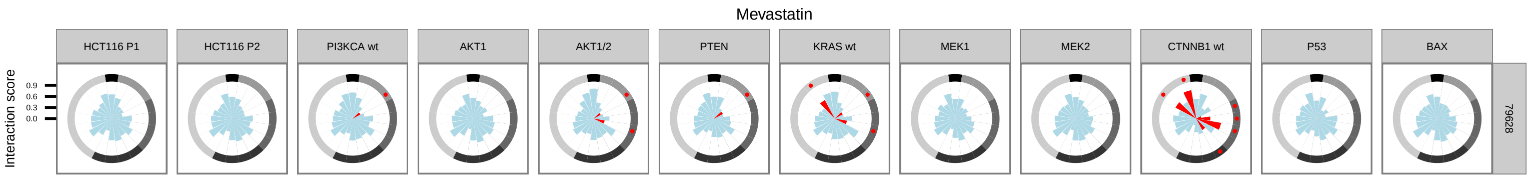
Mevastatin
HCT116 P1
HCT116 P2
PI3KCA wt
AKT1
AKT1/2
PTEN
KRAS wt
MEK1
MEK2
CTNNB1 wt
P53
BAX
Interaction score
0.9
0.6
0.3
0.0
79628

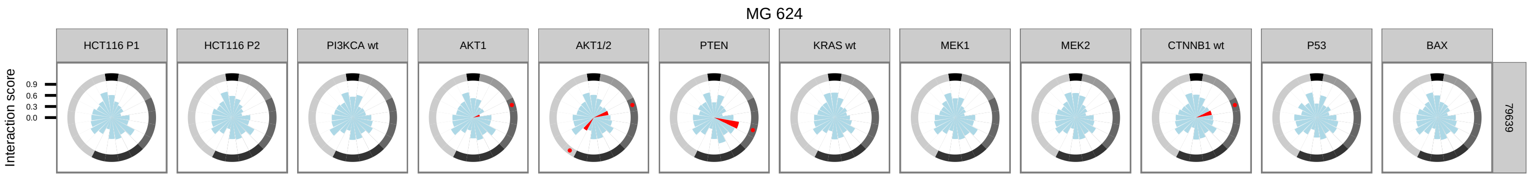
MG 624
HCT116 P1
HCT116 P2
PI3KCA wt
AKT1
AKT1/2
PTEN
KRAS wt
MEK1
MEK2
CTNNB1 wt
P53
BAX
Interaction score
0.9
0.6
0.3
0.0
79639

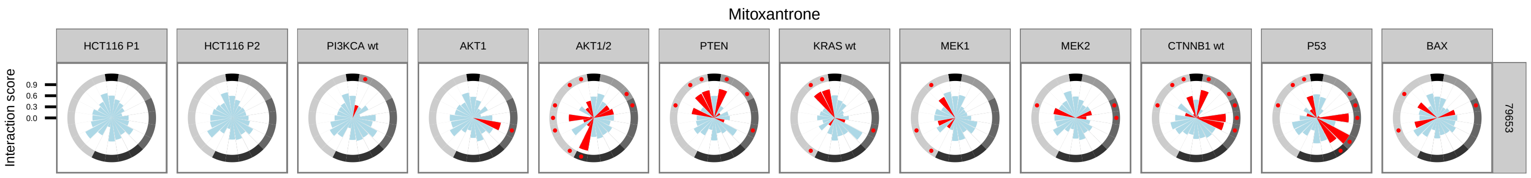
Mitoxantrone
HCT116 P1
HCT116 P2
PI3KCA wt
AKT1
AKT1/2
PTEN
KRAS wt
MEK1
MEK2
CTNNB1 wt
P53
BAX
Interaction score
0.9
0.6
0.3
0.0
79653

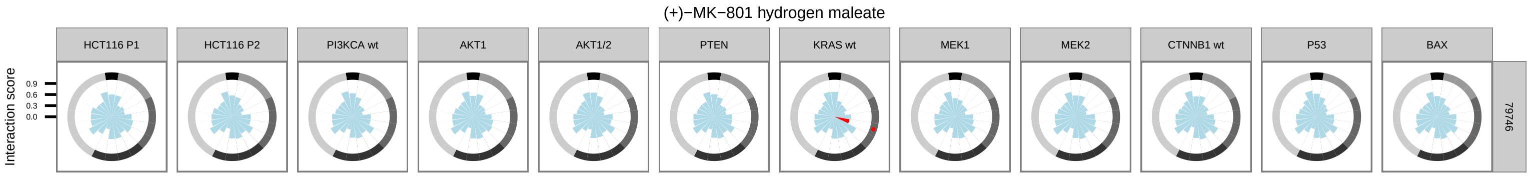
(+)−MK−801 hydrogen maleate
HCT116 P1
HCT116 P2
PI3KCA wt
AKT1
AKT1/2
PTEN
KRAS wt
MEK1
MEK2
CTNNB1 wt
P53
BAX
Interaction score
0.9
0.6
0.3
0.0
79746

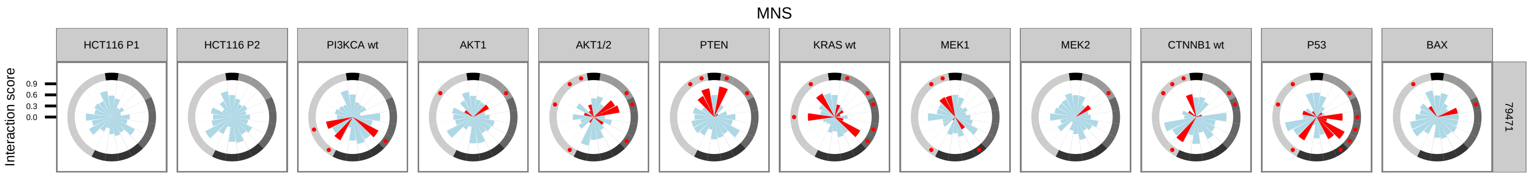
MNS
HCT116 P1
HCT116 P2
PI3KCA wt
AKT1
AKT1/2
PTEN
KRAS wt
MEK1
MEK2
CTNNB1 wt
P53
BAX
Interaction score
0.9
0.6
0.3
0.0
79471

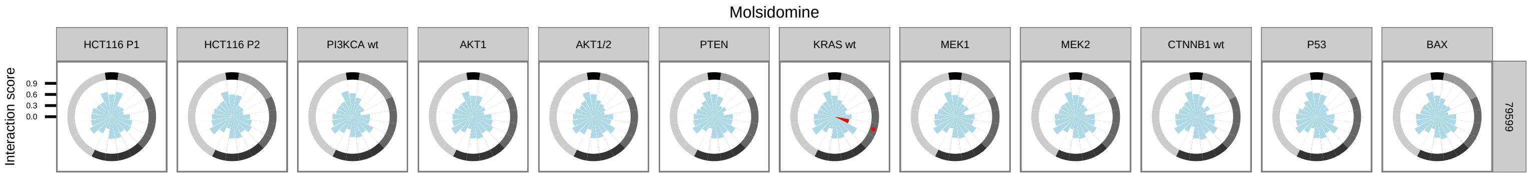
Molsidomine
HCT116 P1
HCT116 P2
PI3KCA wt
AKT1
AKT1/2
PTEN
KRAS wt
MEK1
MEK2
CTNNB1 wt
P53
BAX
Interaction score
0.9
0.6
0.3
0.0
79599

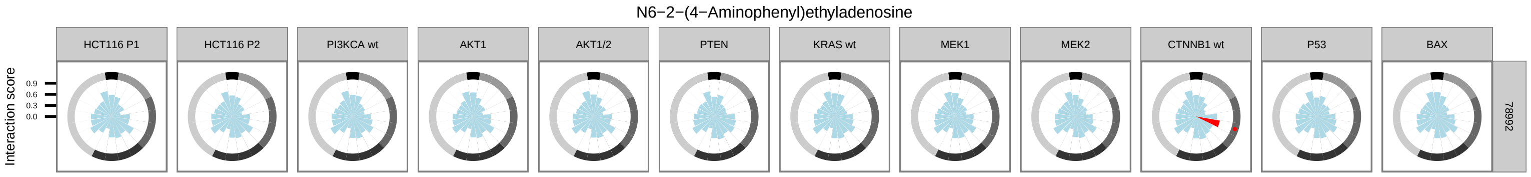
N6−2−(4−Aminophenyl)ethyladenosine
HCT116 P1
HCT116 P2
PI3KCA wt
AKT1
AKT1/2
PTEN
KRAS wt
MEK1
MEK2
CTNNB1 wt
P53
BAX
Interaction score
0.9
0.6
0.3
0.0
78992

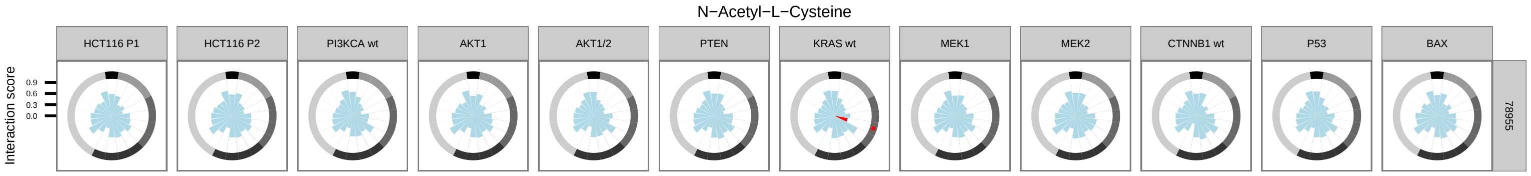
N−Acetyl−L−Cysteine
HCT116 P1
HCT116 P2
PI3KCA wt
AKT1
AKT1/2
PTEN
KRAS wt
MEK1
MEK2
CTNNB1 wt
P53
BAX
Interaction score
0.9
0.6
0.3
0.0
78955

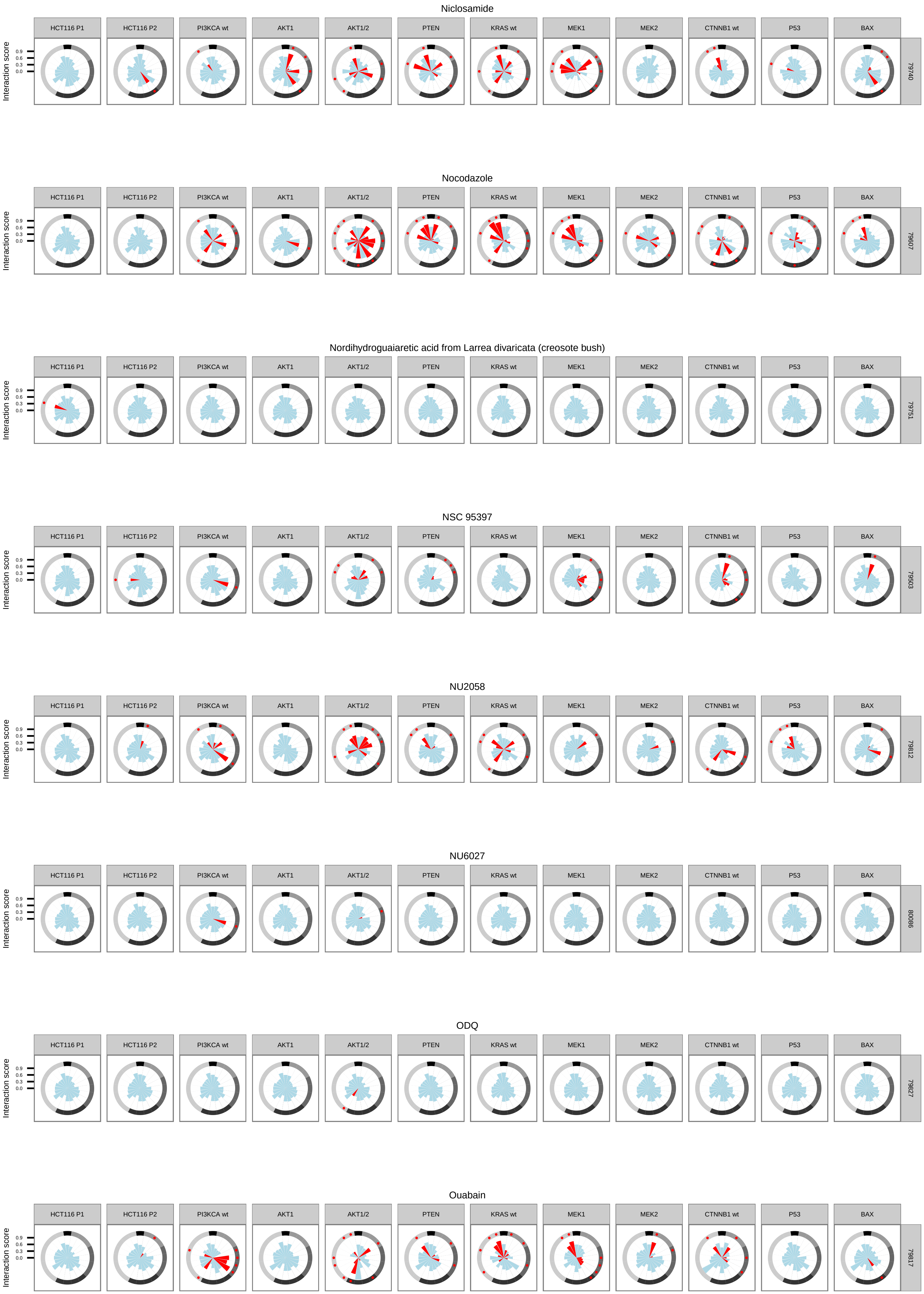
Niclosamide
HCT116 P1
HCT116 P2
PI3KCA wt
AKT1
AKT1/2
PTEN
KRAS wt
MEK1
MEK2
CTNNB1 wt
P53
BAX
Interaction score
0.9
0.6
0.3
0.0
79740
Nocodazole
79607
Nordihydroguaiaretic acid from Larrea divaricata (creosote bush)
79751
NSC 95397
79503
NU2058
79812
NU6027
80086
ODQ
79827
Ouabain
79817

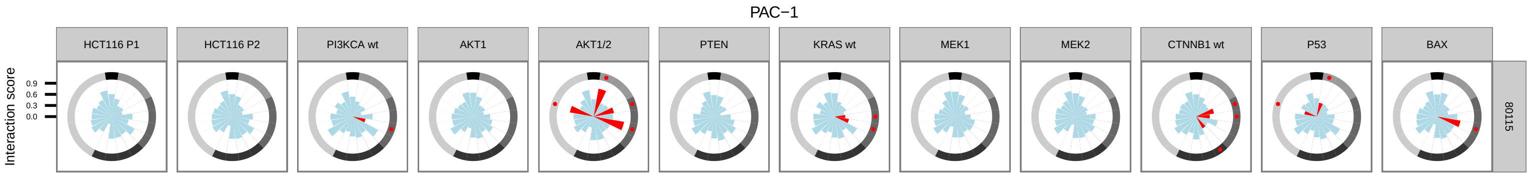
PAC−1
HCT116 P1
HCT116 P2
PI3KCA wt
AKT1
AKT1/2
PTEN
KRAS wt
MEK1
MEK2
CTNNB1 wt
P53
BAX
Interaction score
0.9
0.6
0.3
0.0
80115

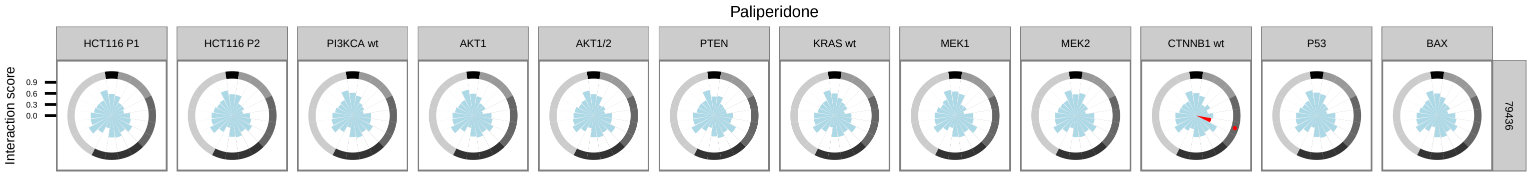
Paliperidone
HCT116 P1
HCT116 P2
PI3KCA wt
AKT1
AKT1/2
PTEN
KRAS wt
MEK1
MEK2
CTNNB1 wt
P53
BAX
Interaction score
0.9
0.6
0.3
0.0
79436

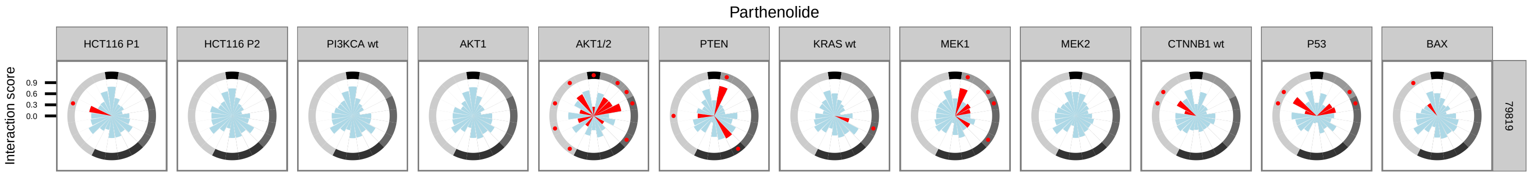
Parthenolide
HCT116 P1
HCT116 P2
PI3KCA wt
AKT1
AKT1/2
PTEN
KRAS wt
MEK1
MEK2
CTNNB1 wt
P53
BAX
Interaction score
0.9
0.6
0.3
0.0
79819

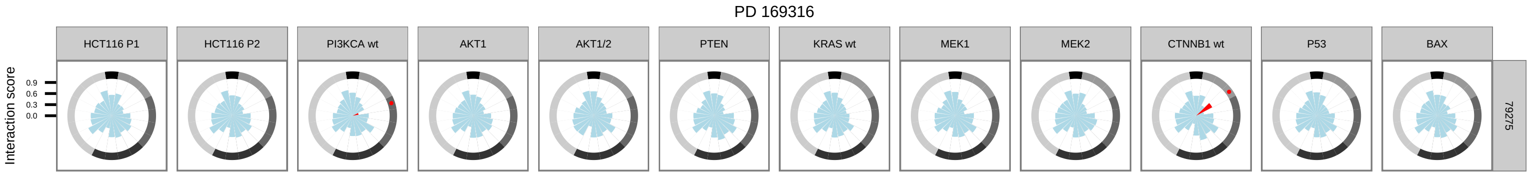
PD 169316
HCT116 P1
HCT116 P2
PI3KCA wt
AKT1
AKT1/2
PTEN
KRAS wt
MEK1
MEK2
CTNNB1 wt
P53
BAX
Interaction score
0.9
0.6
0.3
0.0
79275

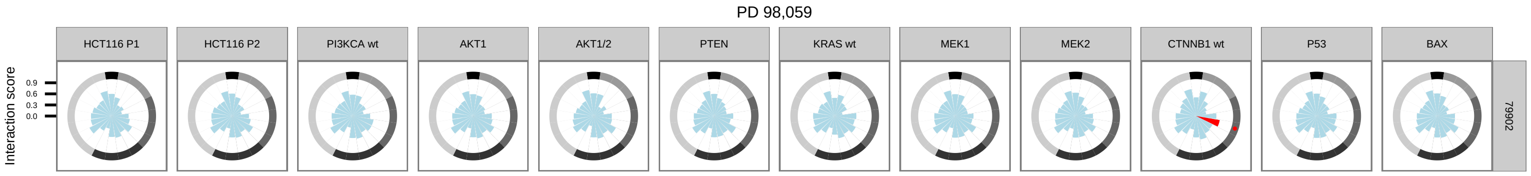
PD 98,059
HCT116 P1
HCT116 P2
PI3KCA wt
AKT1
AKT1/2
PTEN
KRAS wt
MEK1
MEK2
CTNNB1 wt
P53
BAX
Interaction score
0.9
0.6
0.3
0.0
79902

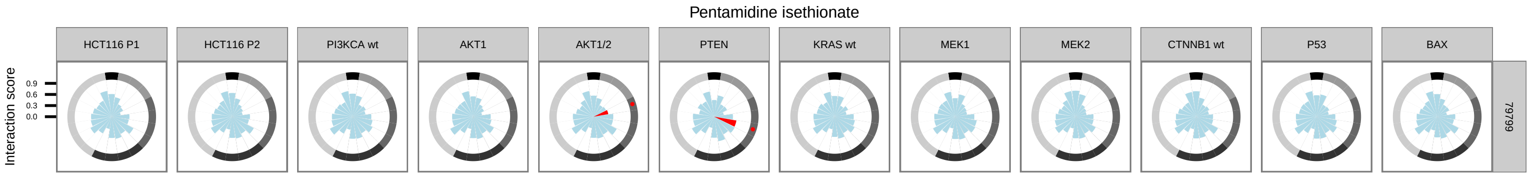
Pentamidine isethionate
HCT116 P1
HCT116 P2
PI3KCA wt
AKT1
AKT1/2
PTEN
KRAS wt
MEK1
MEK2
CTNNB1 wt
P53
BAX
Interaction score
0.9
0.6
0.3
0.0
79799

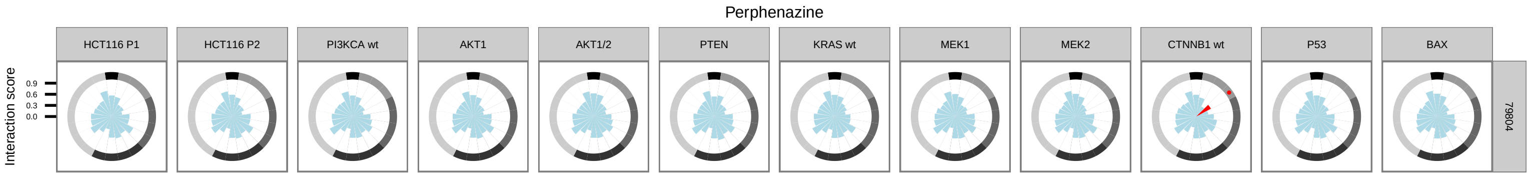
Perphenazine
HCT116 P1
HCT116 P2
PI3KCA wt
AKT1
AKT1/2
PTEN
KRAS wt
MEK1
MEK2
CTNNB1 wt
P53
BAX
Interaction score
0.9
0.6
0.3
0.0
79804

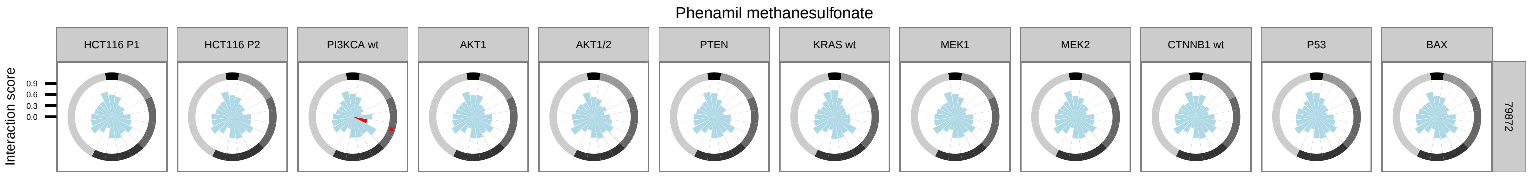
Phenamil methanesulfonate
HCT116 P1
HCT116 P2
PI3KCA wt
AKT1
AKT1/2
PTEN
KRAS wt
MEK1
MEK2
CTNNB1 wt
P53
BAX
Interaction score
0.9
0.6
0.3
0.0
79872

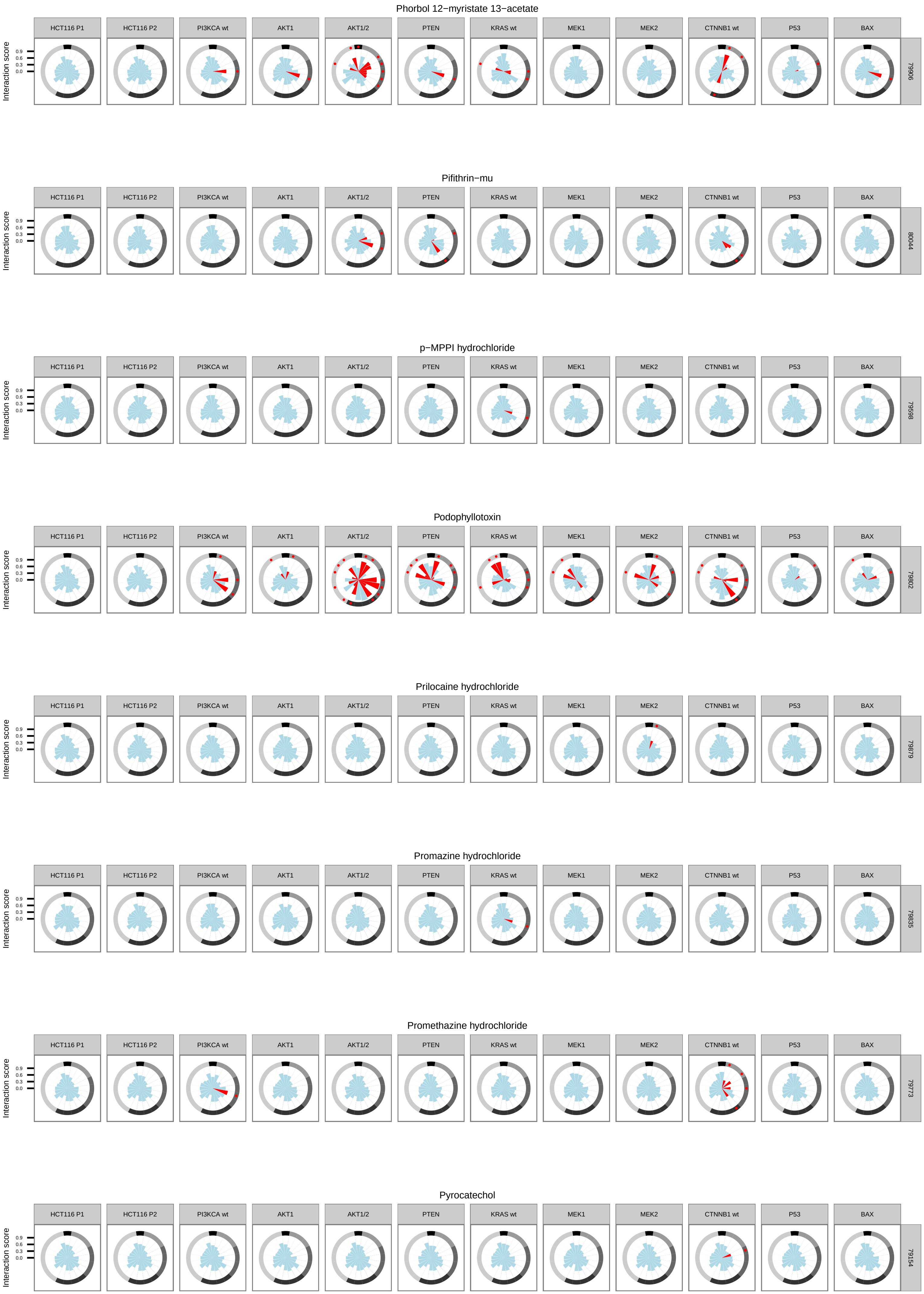

Phorbol 12−myristate 13−acetate
HCT116 P1
HCT116 P2
PI3KCA wt
AKT1
AKT1/2
PTEN
KRAS wt
MEK1
MEK2
CTNNB1 wt
P53
BAX
Interaction score
0.9
0.6
0.3
0.0
79906
Pifithrin−mu
80044
p−MPPI hydrochloride
79598
Podophyllotoxin
79802
Prilocaine hydrochloride
79879
Promazine hydrochloride
79835
Promethazine hydrochloride
79773
Pyrocatechol
79154

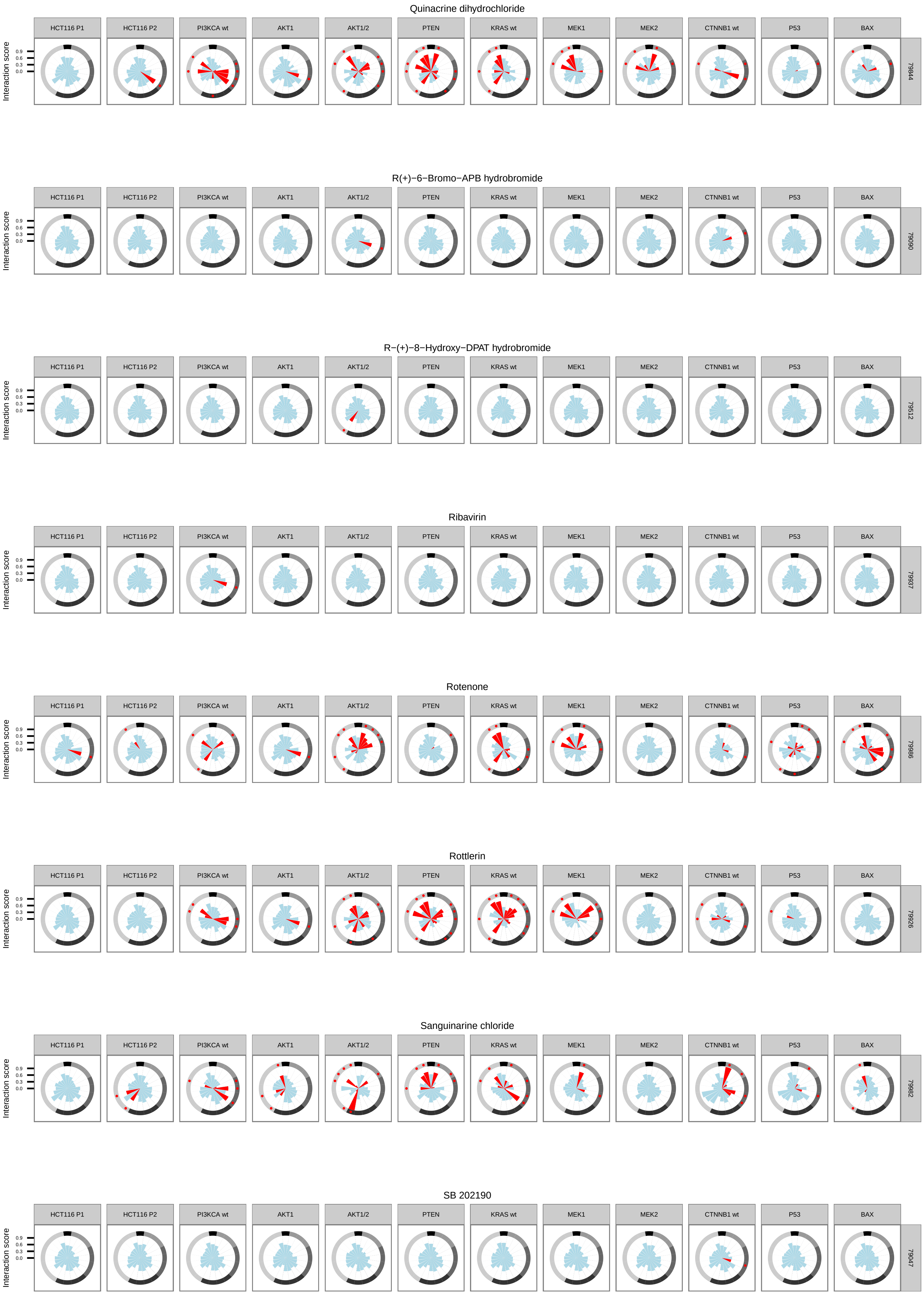
Quinacrine dihydrochloride
R(+)−6−Bromo−APB hydrobromide
R−(+)−8−Hydroxy−DPAT hydrobromide
Ribavirin
Rotenone
Rottlerin
Sanguinarine chloride
SB 202190
HCT116 P1
HCT116 P2
PI3KCA wt
AKT1
AKT1/2
PTEN
KRAS wt
MEK1
MEK2
CTNNB1 wt
P53
BAX
Interaction score
0.9
0.6
0.3
0.0
79844
79090
79512
79937
79986
79926
79982
79047

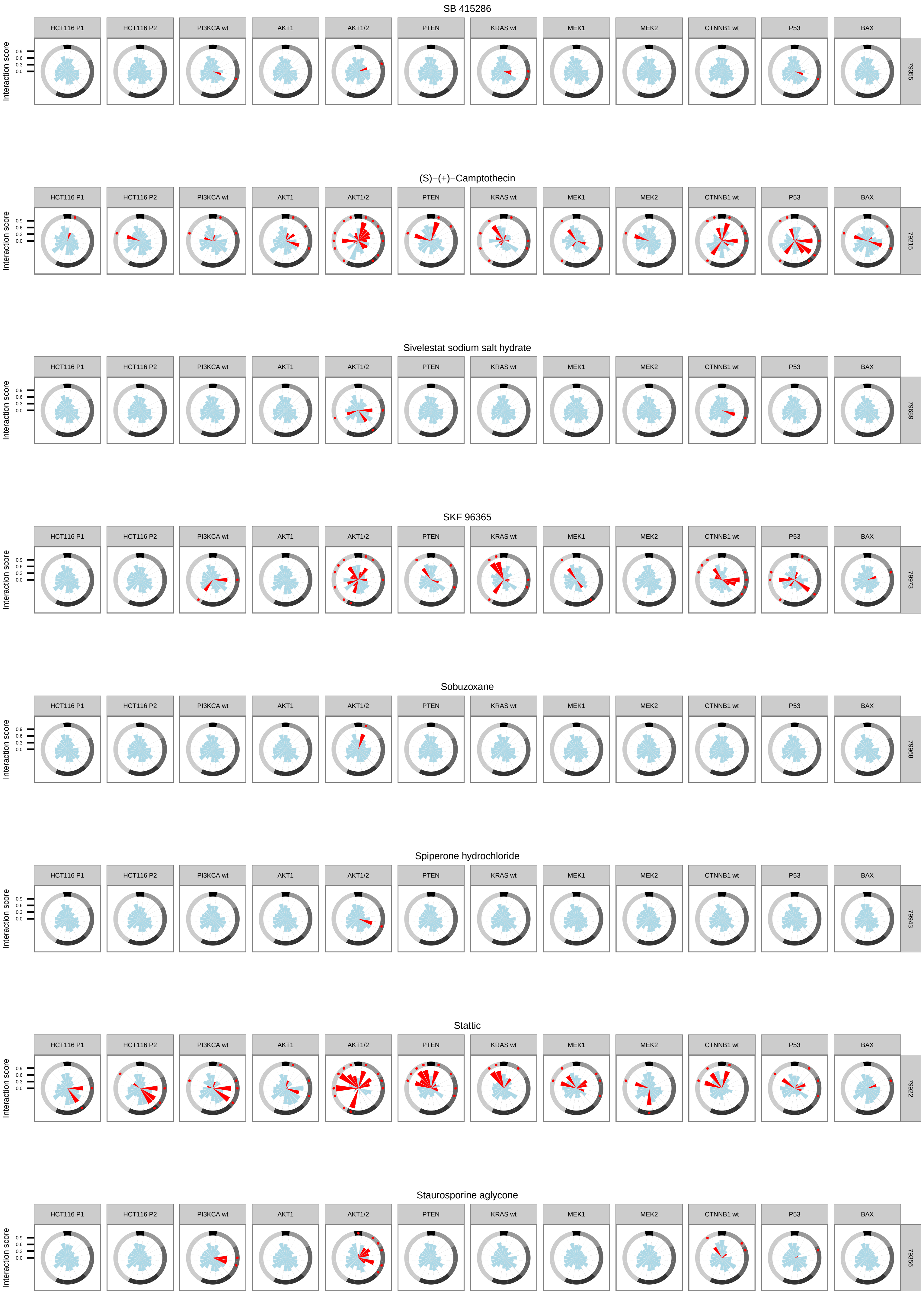
SB 415286
(S)−(+)−Camptothecin
Sivelestat sodium salt hydrate
SKF 96365
Sobuzoxane
Spiperone hydrochloride
Stattic
Staurosporine aglycone
HCT116 P1
HCT116 P2
PI3KCA wt
AKT1
AKT1/2
PTEN
KRAS wt
MEK1
MEK2
CTNNB1 wt
P53
BAX
Interaction score
0.9
0.6
0.3
0.0
79355
79215
79689
79973
79968
79943
79922
79956

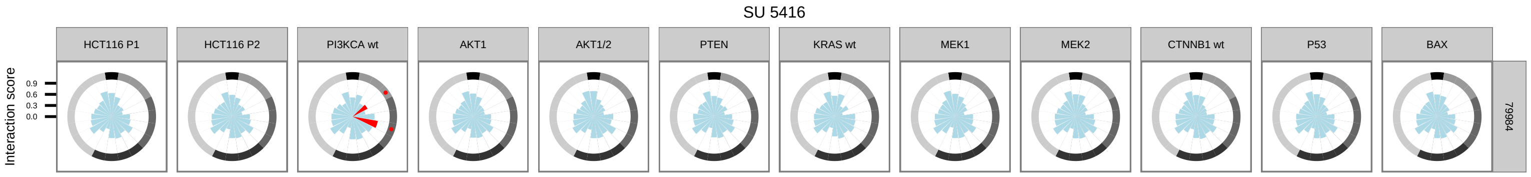
SU 5416
HCT116 P1
HCT116 P2
PI3KCA wt
AKT1
AKT1/2
PTEN
KRAS wt
MEK1
MEK2
CTNNB1 wt
P53
BAX
Interaction score
0.9
0.6
0.3
0.0
79984

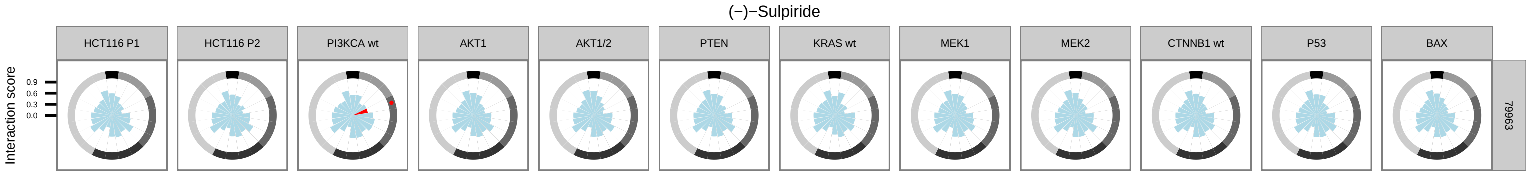
(−)−Sulpiride
HCT116 P1
HCT116 P2
PI3KCA wt
AKT1
AKT1/2
PTEN
KRAS wt
MEK1
MEK2
CTNNB1 wt
P53
BAX
Interaction score
0.9
0.6
0.3
0.0
79963

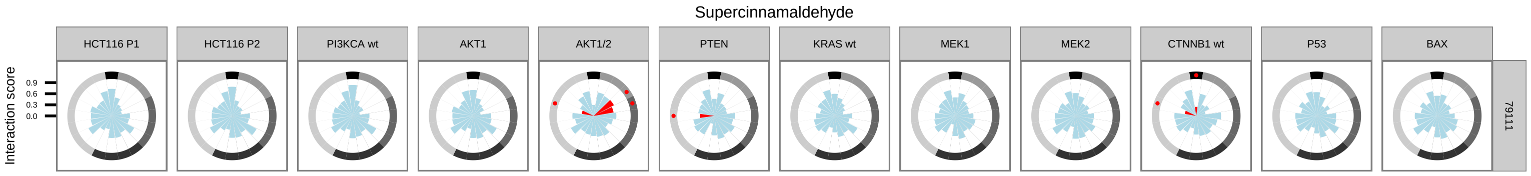
Supercinnamaldehyde
HCT116 P1
HCT116 P2
PI3KCA wt
AKT1
AKT1/2
PTEN
KRAS wt
MEK1
MEK2
CTNNB1 wt
P53
BAX
Interaction score
0.9
0.6
0.3
0.0
79111

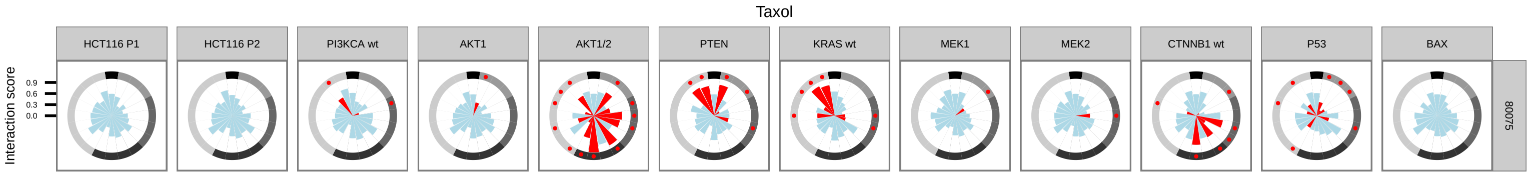
Taxol
HCT116 P1
HCT116 P2
PI3KCA wt
AKT1
AKT1/2
PTEN
KRAS wt
MEK1
MEK2
CTNNB1 wt
P53
BAX
Interaction score
0.9
0.6
0.3
0.0
80075

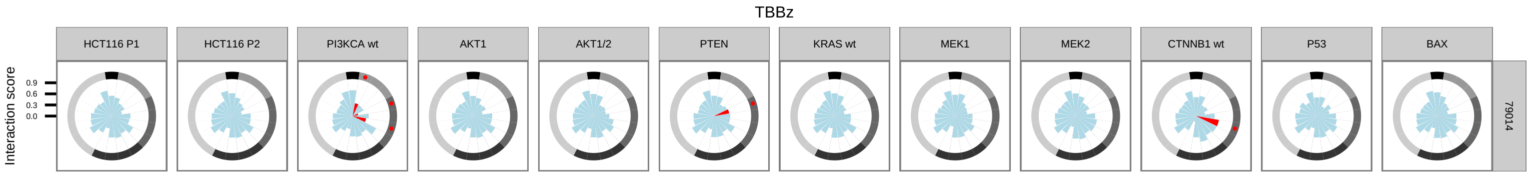
TBBz
HCT116 P1
HCT116 P2
PI3KCA wt
AKT1
AKT1/2
PTEN
KRAS wt
MEK1
MEK2
CTNNB1 wt
P53
BAX
Interaction score
0.9
0.6
0.3
0.0
79014

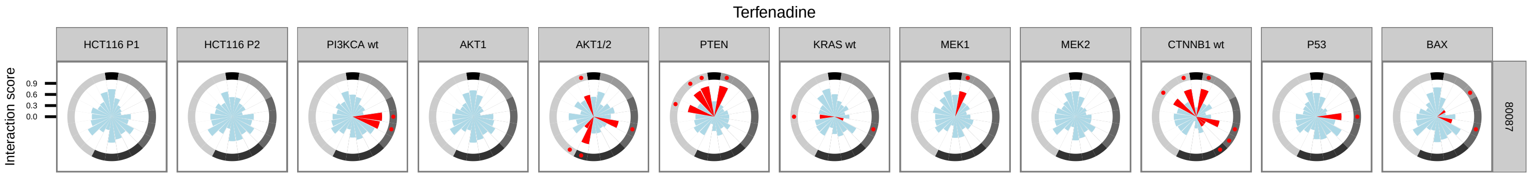
Terfenadine
HCT116 P1
HCT116 P2
PI3KCA wt
AKT1
AKT1/2
PTEN
KRAS wt
MEK1
MEK2
CTNNB1 wt
P53
BAX
Interaction score
0.9
0.6
0.3
0.0
80087

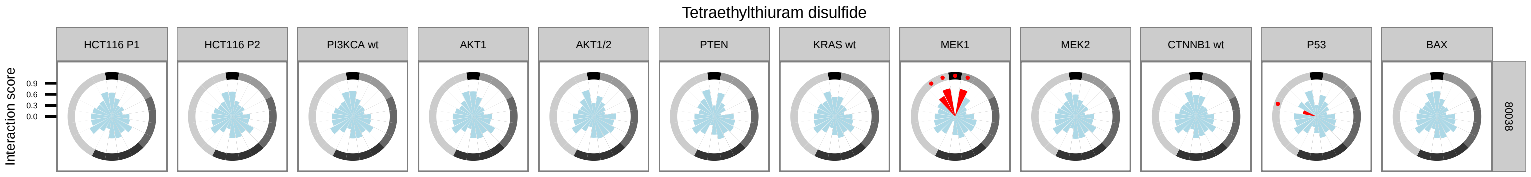
Tetraethylthiuram disulfide
HCT116 P1
HCT116 P2
PI3KCA wt
AKT1
AKT1/2
PTEN
KRAS wt
MEK1
MEK2
CTNNB1 wt
P53
BAX
Interaction score
0.9
0.6
0.3
0.0
80038

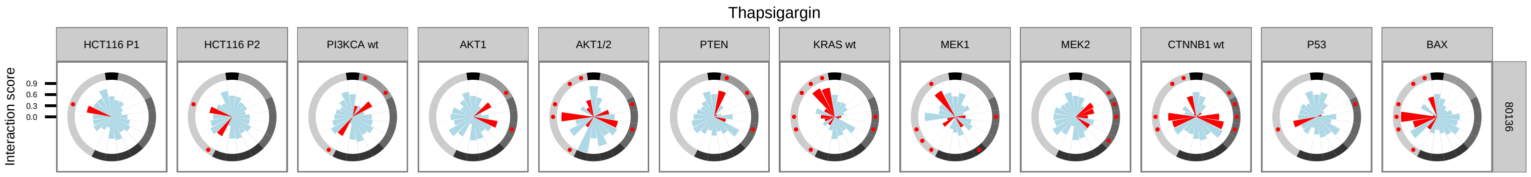
Thapsigargin
HCT116 P1
HCT116 P2
PI3KCA wt
AKT1
AKT1/2
PTEN
KRAS wt
MEK1
MEK2
CTNNB1 wt
P53
BAX
Interaction score
0.9
0.6
0.3
0.0
80136

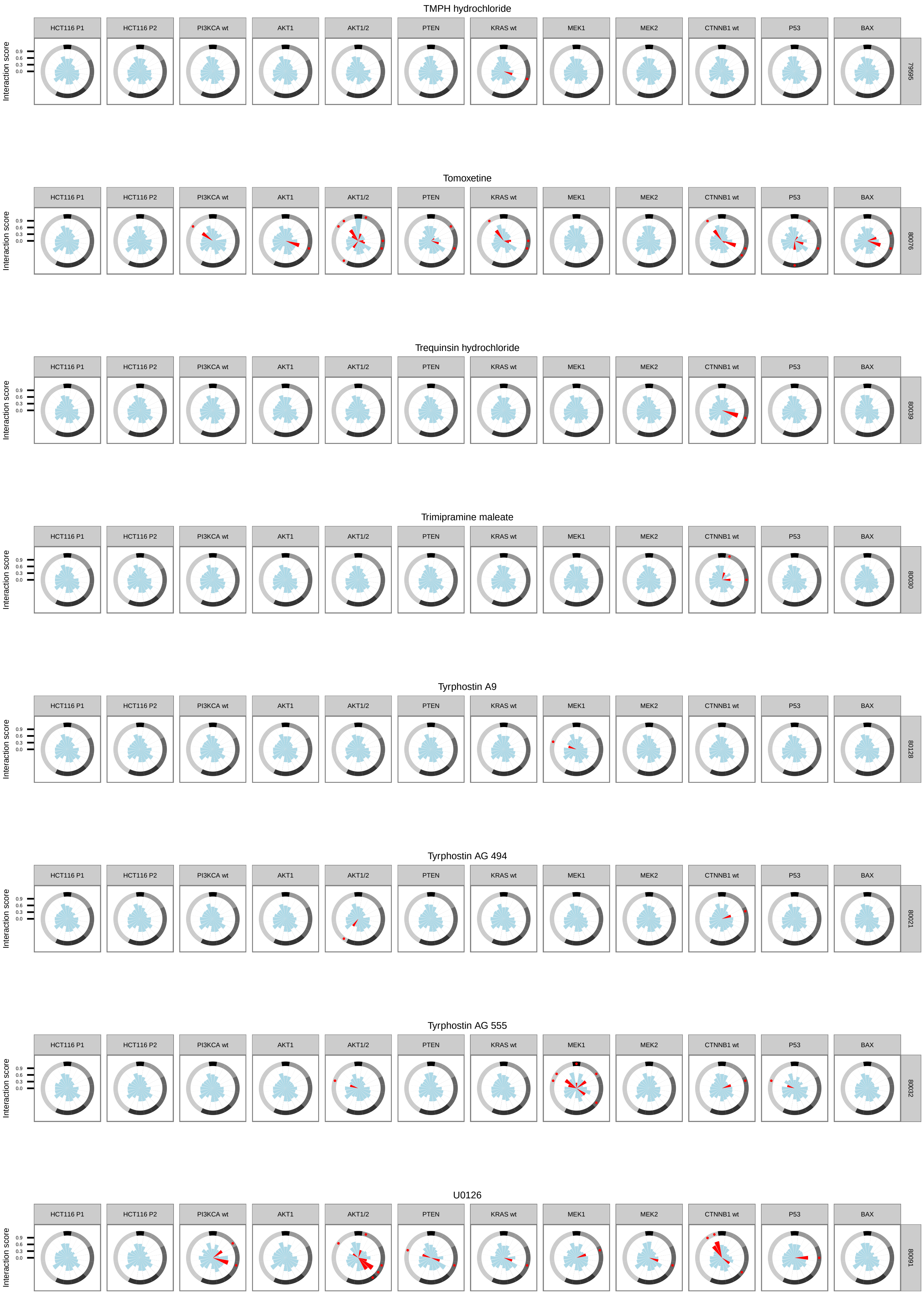

TMPH hydrochloride
HCT116 P1
HCT116 P2
PI3KCA wt
AKT1
AKT1/2
PTEN
KRAS wt
MEK1
MEK2
CTNNB1 wt
P53
BAX
Interaction score
0.9
0.6
0.3
0.0
79595
Tomoxetine
80076
Trequinsin hydrochloride
80039
Trimipramine maleate
80030
Tyrphostin A9
80128
Tyrphostin AG 494
80021
Tyrphostin AG 555
80032
U0126
80091

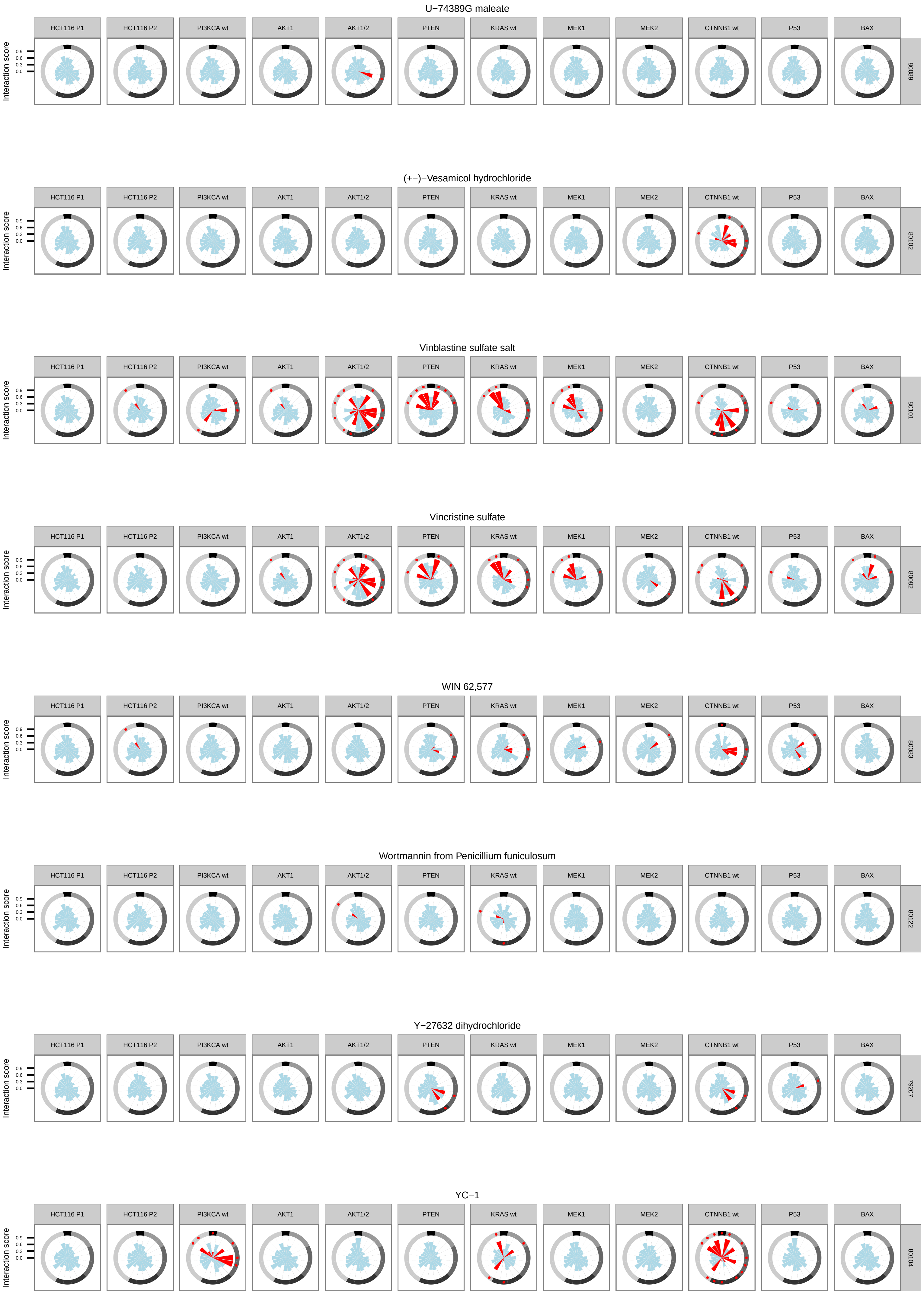

U−74389G maleate
HCT116 P1
HCT116 P2
PI3KCA wt
AKT1
AKT1/2
PTEN
KRAS wt
MEK1
MEK2
CTNNB1 wt
P53
BAX
Interaction score
0.9
0.6
0.3
0.0
80089
(+−)−Vesamicol hydrochloride
HCT116 P1
HCT116 P2
PI3KCA wt
AKT1
AKT1/2
PTEN
KRAS wt
MEK1
MEK2
CTNNB1 wt
P53
BAX
Interaction score
0.9
0.6
0.3
0.0
80102
Vinblastine sulfate salt
HCT116 P1
HCT116 P2
PI3KCA wt
AKT1
AKT1/2
PTEN
KRAS wt
MEK1
MEK2
CTNNB1 wt
P53
BAX
Interaction score
0.9
0.6
0.3
0.0
80101
Vincristine sulfate
HCT116 P1
HCT116 P2
PI3KCA wt
AKT1
AKT1/2
PTEN
KRAS wt
MEK1
MEK2
CTNNB1 wt
P53
BAX
Interaction score
0.9
0.6
0.3
0.0
80082
WIN 62,577
HCT116 P1
HCT116 P2
PI3KCA wt
AKT1
AKT1/2
PTEN
KRAS wt
MEK1
MEK2
CTNNB1 wt
P53
BAX
Interaction score
0.9
0.6
0.3
0.0
80083
Wortmannin from Penicillium funiculosum
HCT116 P1
HCT116 P2
PI3KCA wt
AKT1
AKT1/2
PTEN
KRAS wt
MEK1
MEK2
CTNNB1 wt
P53
BAX
Interaction score
0.9
0.6
0.3
0.0
80122
Y−27632 dihydrochloride
HCT116 P1
HCT116 P2
PI3KCA wt
AKT1
AKT1/2
PTEN
KRAS wt
MEK1
MEK2
CTNNB1 wt
P53
BAX
Interaction score
0.9
0.6
0.3
0.0
79207
YC−1
HCT116 P1
HCT116 P2
PI3KCA wt
AKT1
AKT1/2
PTEN
KRAS wt
MEK1
MEK2
CTNNB1 wt
P53
BAX
Interaction score
0.9
0.6
0.3
0.0
80104

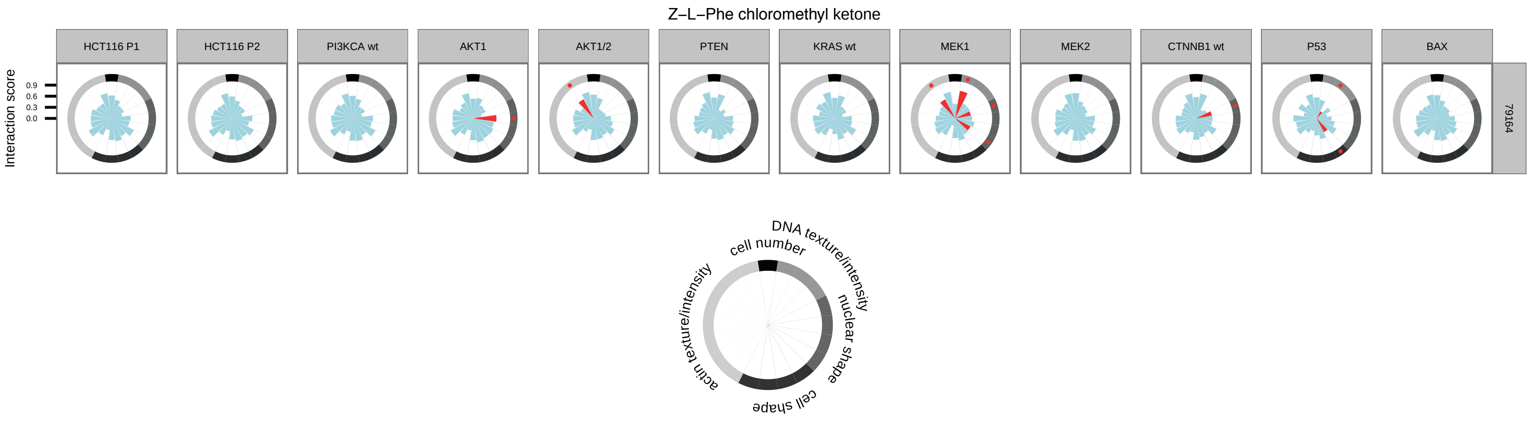

Appendix Figure S4. Significant multiparametric chemical-genetic interactions.

Chemical-genetic interactions were calculated for all 20 phenotypic features as described. A total of 193 compounds revealed significant interactions and their respective interaction spectra are plotted alphabetically.
Interactions are scaled from 0-1. * FDR < 0.01, highlighted in red.

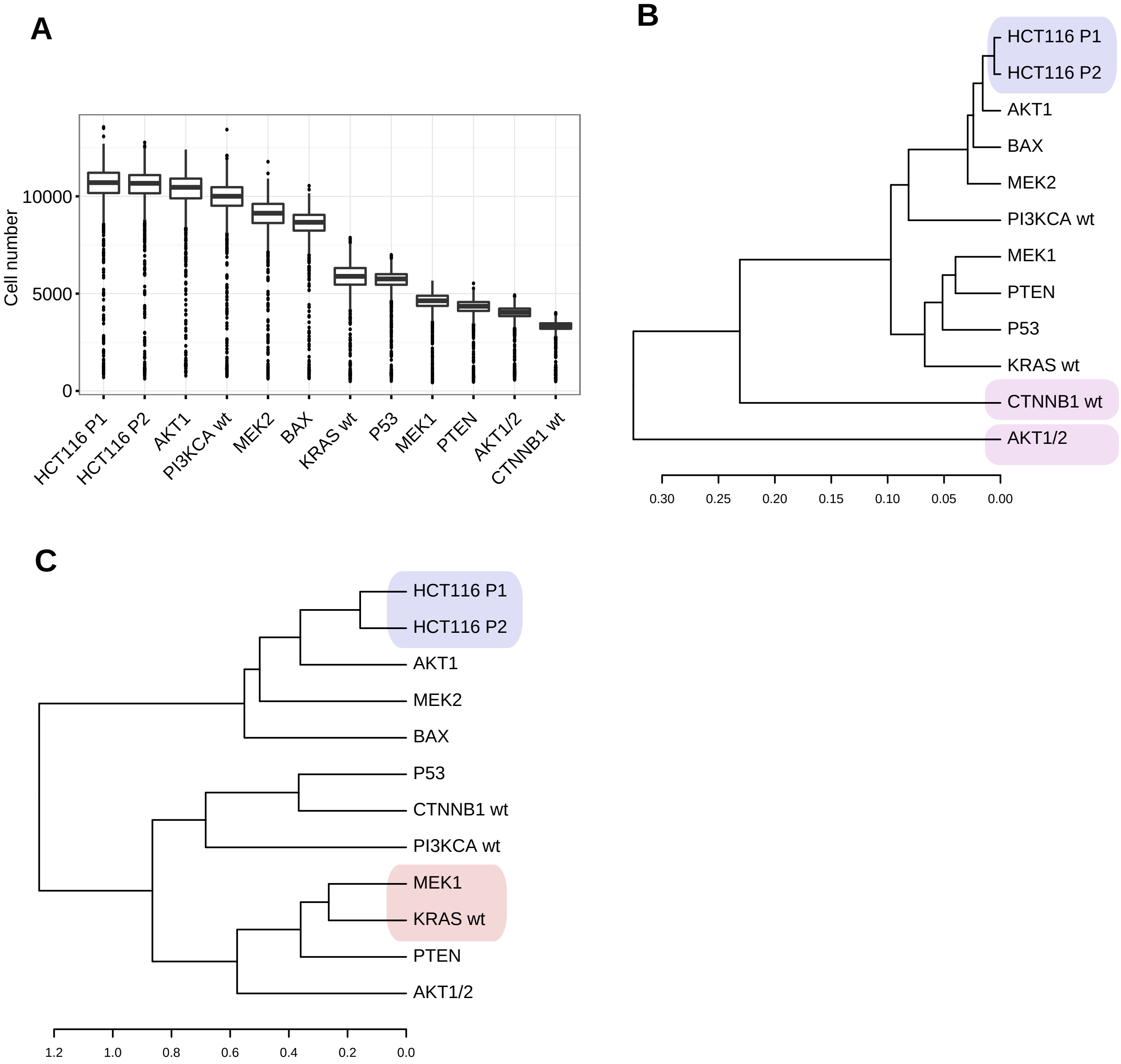

Appendix Figure S5. Strong main effects of gene KO and associations of isogenic cell lines.
A Cell number for each isogenic cell line tested after 48h growth under control conditions (DMSO treatment). Particular isogenic cell lines display a pronounced phenotypic effect, i.e. impaired proliferation as compared to parental HCT116 cells.
B Unsupervised clustering of isogenic cell lines based on their 20-feature phenotype signature reveals that the parental HCT116 cell lines from two different sources cluster tightly together. CTNNB1 wt cells and AKT1/2 KO cells cluster apart from the other cell lines, indicating that the KO of respective genes induces strong phenotypic main effects.
C Unsupervised clustering of the phenotypic chemical-genetic interaction profiles for all drugs on all 20 phenotypic features reveals that the parental HCT116 cell lines from two different sources cluster tightly together. KRAS wt cluster together with MEK1 KO and not with MEK2 KO cells, indicating the key role of MEK1 in KRAS signaling.

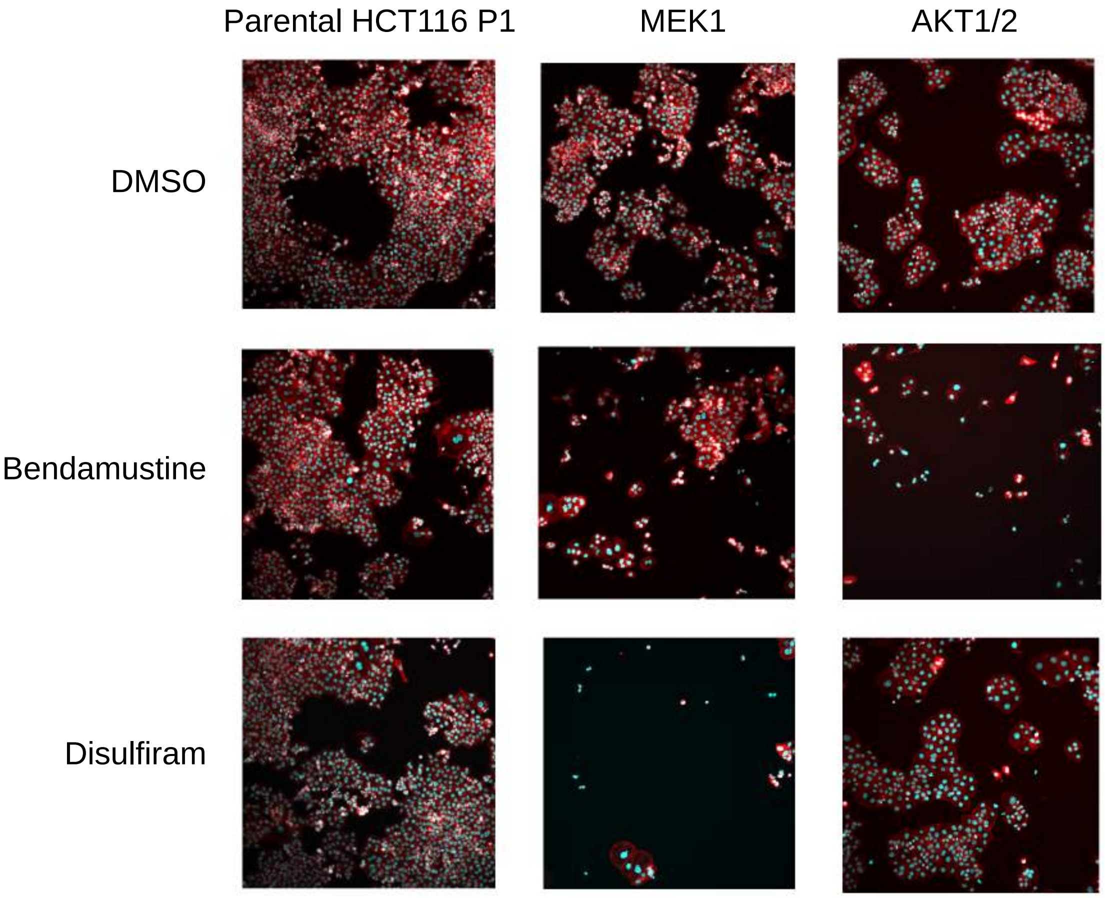

Appendix Figure S6. Synthetic lethal pharmacogenetic interactions. Bendamustine specifically impaired the growth of AKT1/2 KO cells. Disulfiram specifically impaired the growth of MEK1 KO cells. Actin = red, DNA = cyan.

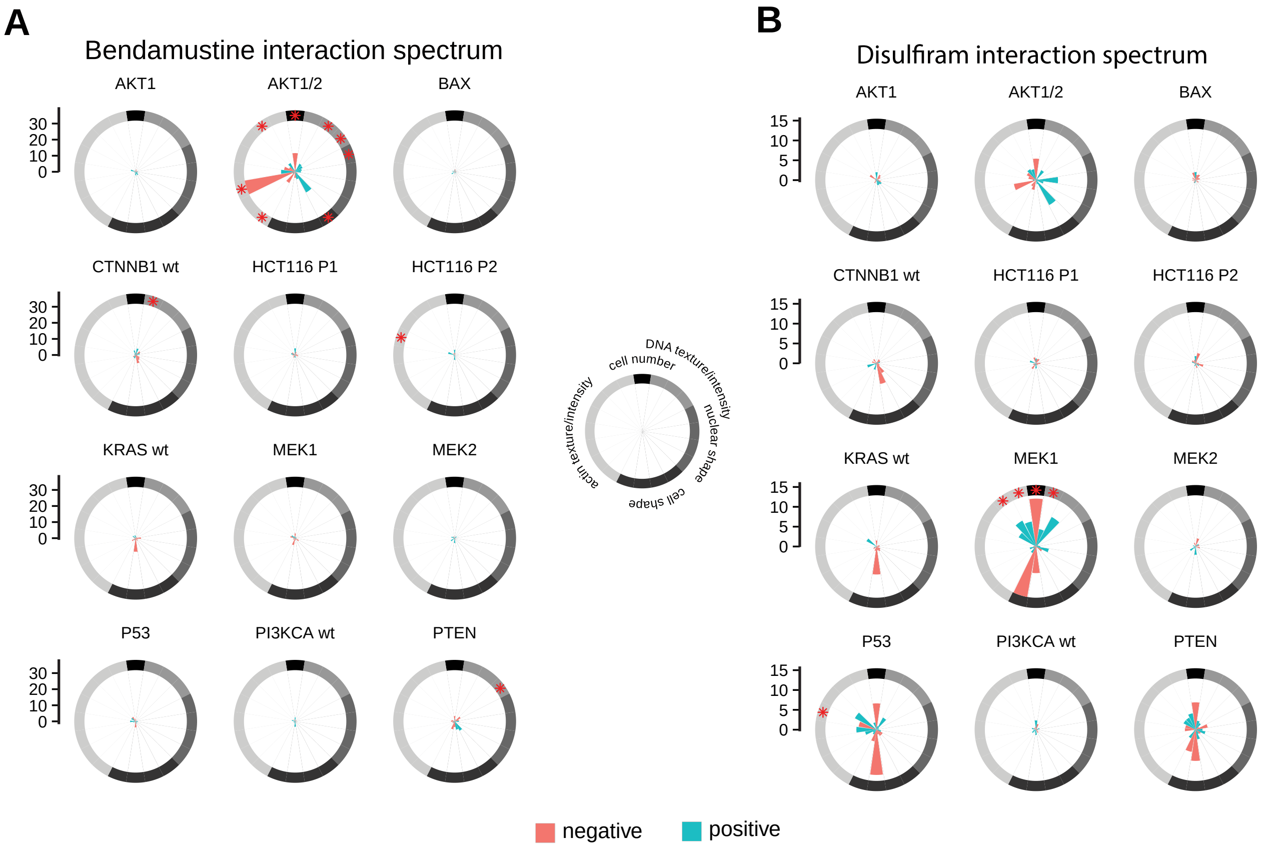

Appendix Figure S7. Bendamustine and disulfiram interaction spectrum.

A Bendamustine interaction spectrum. Bendamustine revealed chemical-genetic interactions across multiple phenotypic features including cell number in AKT1/2 double KO cells (highlighted in red).

B Disulfiram interaction spectrum. Disulfiram revealed chemical-genetic interactions across multiple phenotypic features, including cell number in MEK1 KO cells (highlighted in red). Phenotypic chemo-genomic interactions are shown unscaled which reveals that interactions are rare. Interactions are further categorized in positive and negative interactions according to the sign of interaction terms. * adjusted p-value < 0.01.

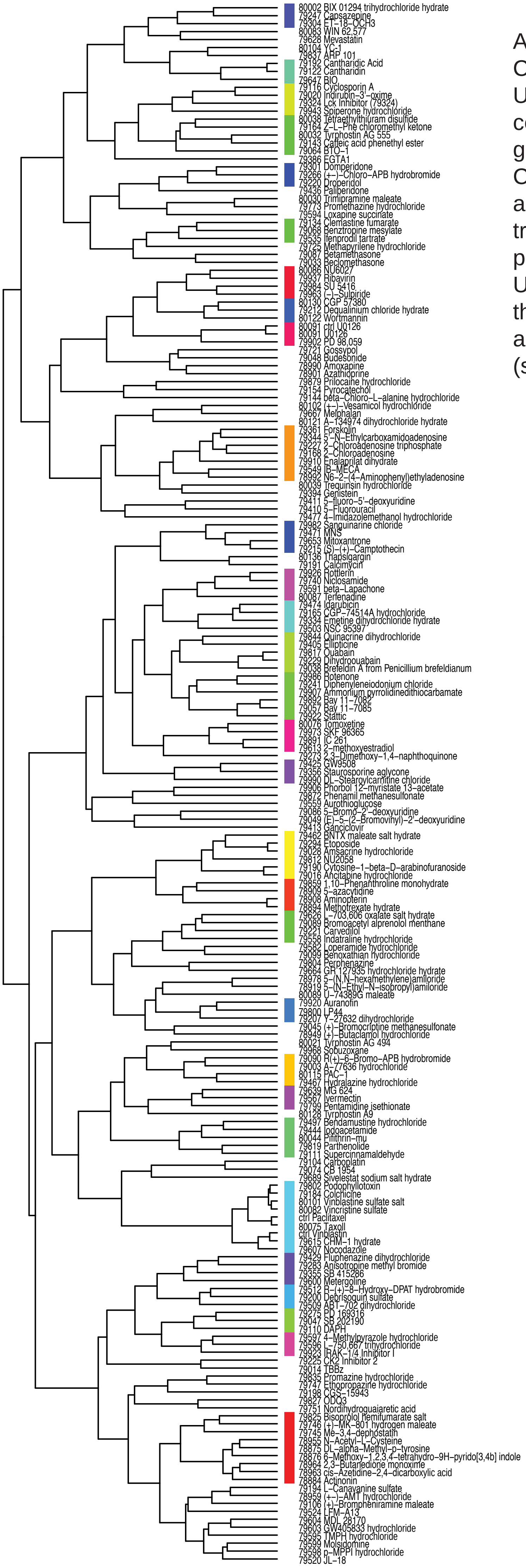

Appendix Figure S8.
Clustering of chemical-genetic interactions.
Unsupervised clustering based on the correlation of interaction profiles of all genetic backgrounds for 20 phenotypic features. Color coding visualizes automated cluster analysis using a 0.6 height cutoff for the cluster tree and the inclusion of >2 and <10 drugs per cluster.
Unique drug identifiers for compounds in the LOPAC library as supplied by Sigma as well as compound names are depicted (see also Table EV1).

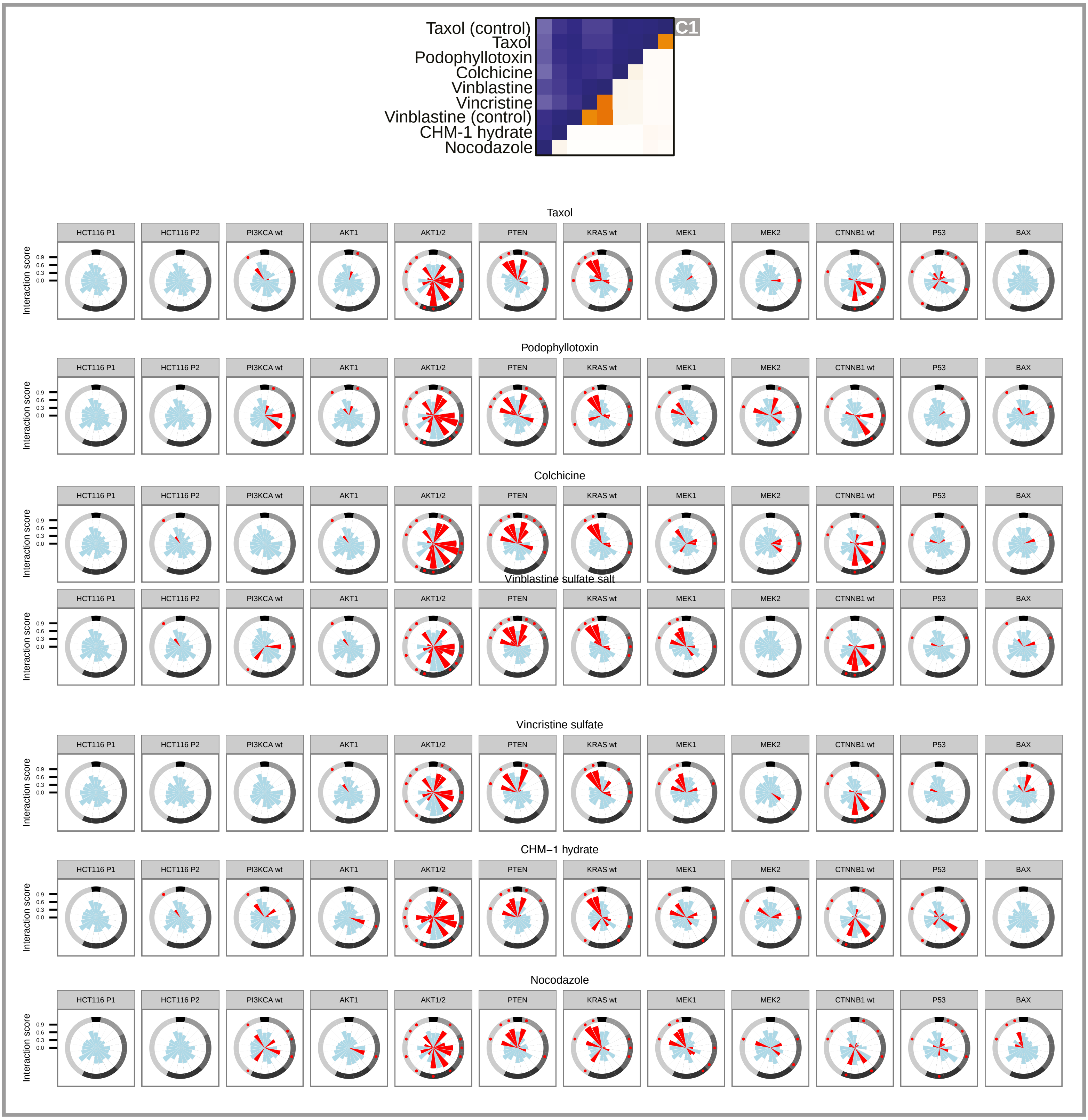

Taxol (control)
Taxol
Podophyllotoxin
Colchicine
Vinblastine
Vincristine
Vinblastine (control)
CHM-1 hydrate
Nocodazole
C1
Taxol
Podophyllotoxin
Colchicine
Vinblastine sulfate salt
Vincristine sulfate
CHM−1 hydrate
Nocodazole
HCT116 P1
HCT116 P2
PI3KCA wt
AKT1
AKT1/2
PTEN
KRAS wt
MEK1
MEK2
CTNNB1 wt
P53
BAX
Interaction score
0.9
0.6
0.3
0.0

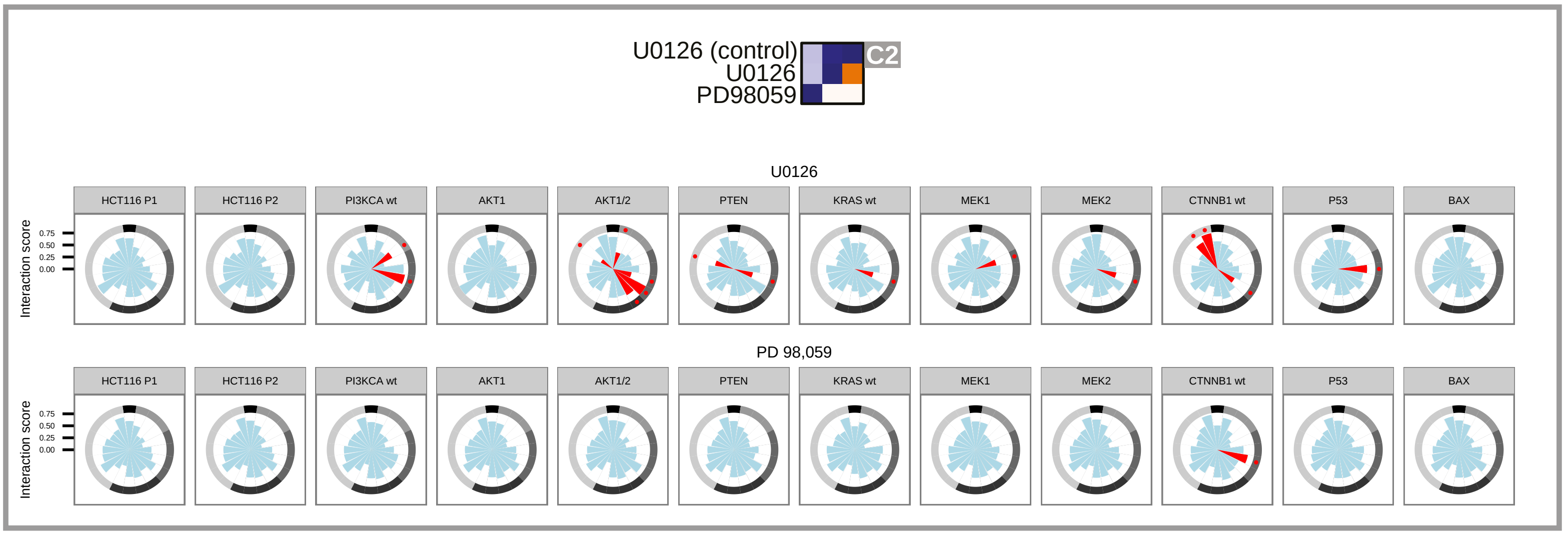

U0126 (control)
U0126
PD98059
C2
U0126
PD 98,059
HCT116 P1
HCT116 P2
PI3KCA wt
AKT1
AKT1/2
PTEN
KRAS wt
MEK1
MEK2
CTNNB1 wt
P53
BAX
Interaction score
0.75
0.50
0.25
0.00

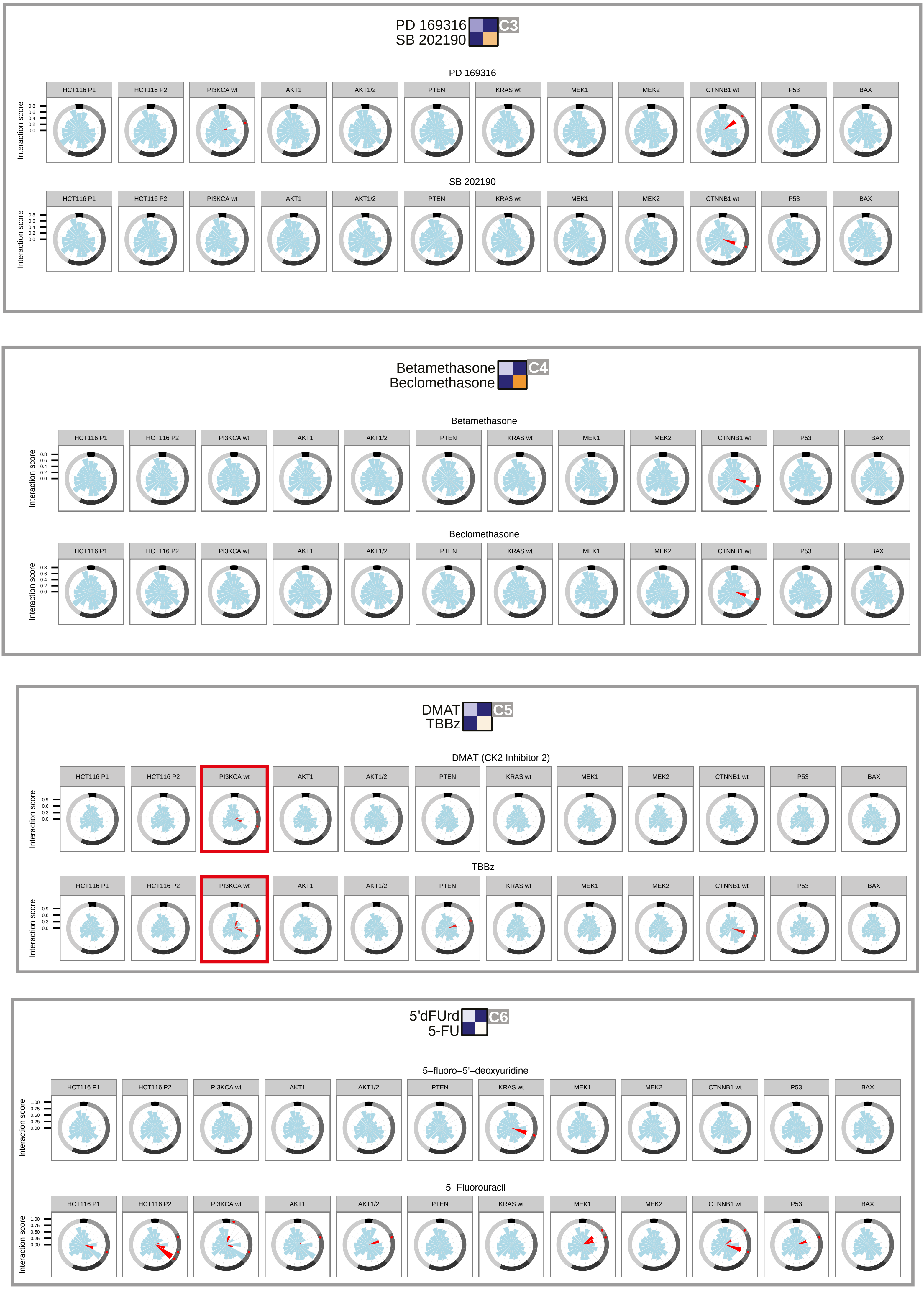

PD 169316
SB 202190
C3
Interaction score
0.8
0.6
0.4
0.2
0.0
HCT116 P1
HCT116 P2
PI3KCA wt
AKT1
AKT1/2
PTEN
KRAS wt
MEK1
MEK2
CTNNB1 wt
P53
BAX
Betamethasone
Beclomethasone
C4
DMAT
TBBz
C5
DMAT (CK2 Inhibitor 2)
0.9
0.6
0.3
0.0
5'dFUrd
5-FU
C6
5–fluoro–5'–deoxyuridine
5–Fluorouracil
1.00
0.75
0.50
0.25
0.00

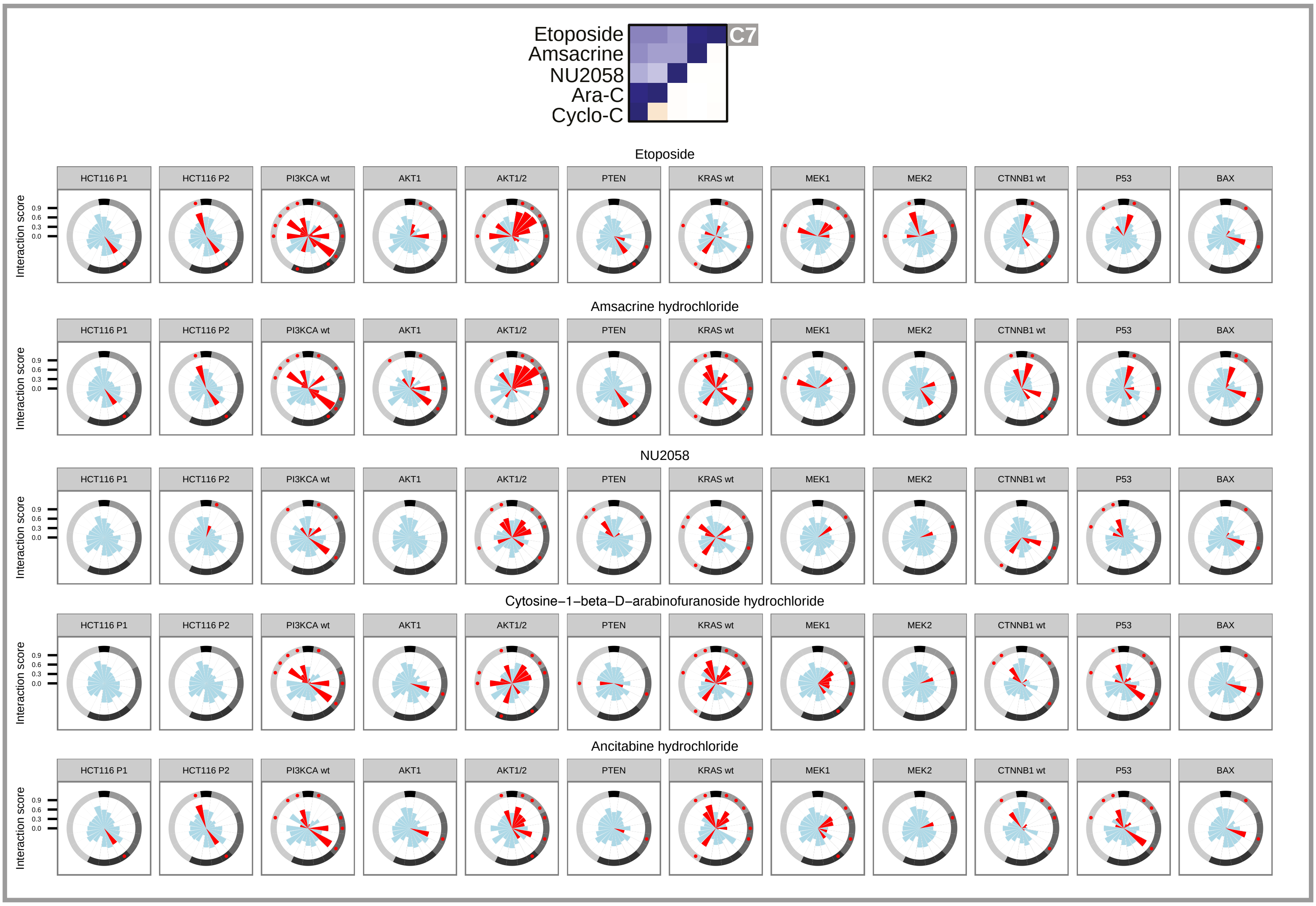
Etoposide
Amsacrine
NU2058
Ara-C
Cyclo-C
C7
Etoposide
Amsacrine hydrochloride
NU2058
Cytosine–1–beta–D–arabinofuranoside hydrochloride
Ancitabine hydrochloride
HCT116 P1
HCT116 P2
PI3KCA wt
AKT1
AKT1/2
PTEN
KRAS wt
MEK1
MEK2
CTNNB1 wt
P53
BAX
Interaction score
0.9
0.6
0.3
0.0

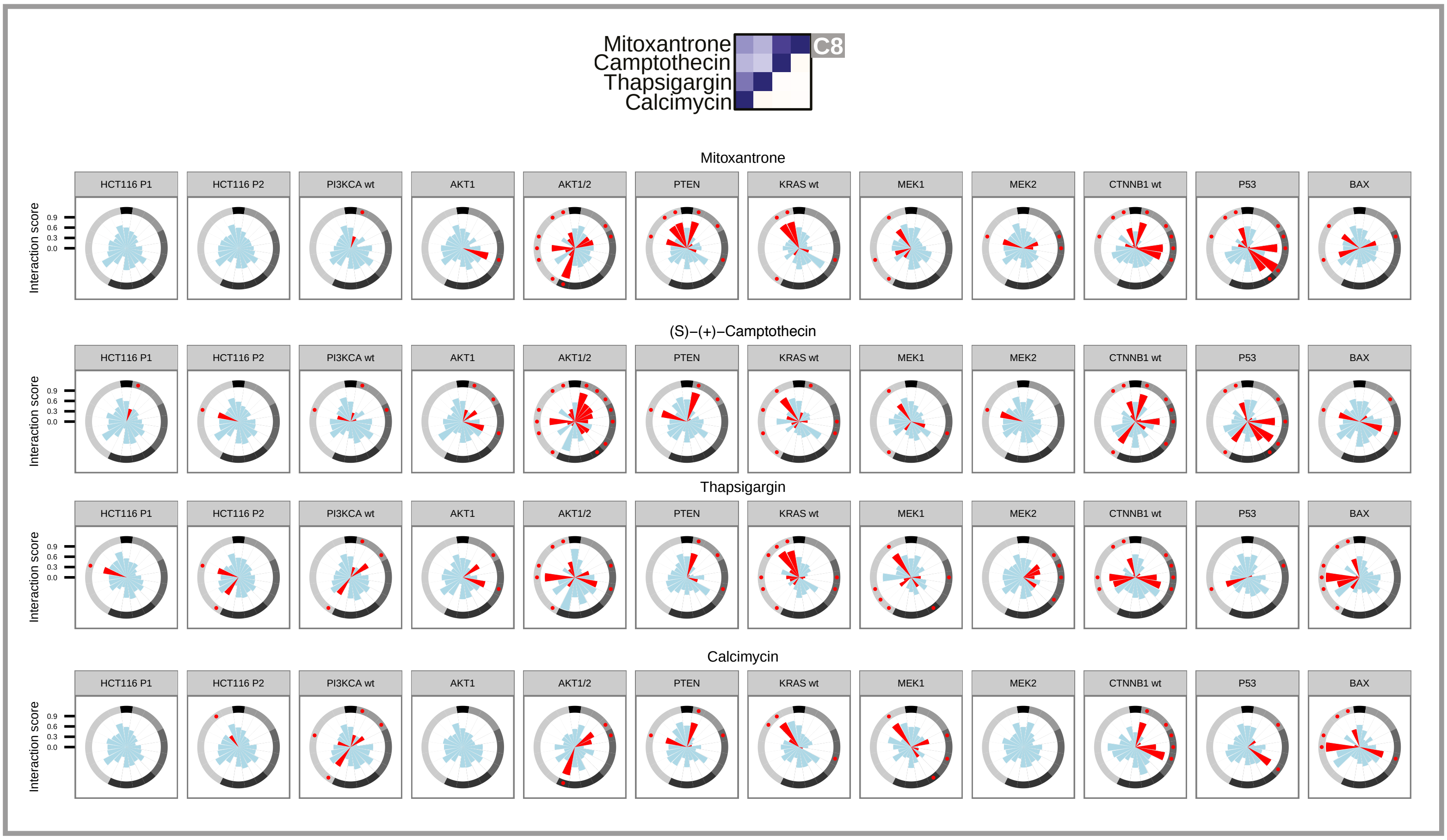
Mitoxantrone
Camptothecin
Thapsigargin
Calcimycin
C8
Mitoxantrone
(S)–(+)–Camptothecin
Thapsigargin
Calcimycin
HCT116 P1
HCT116 P2
PI3KCA wt
AKT1
AKT1/2
PTEN
KRAS wt
MEK1
MEK2
CTNNB1 wt
P53
BAX
Interaction score
0.9
0.6
0.3
0.0

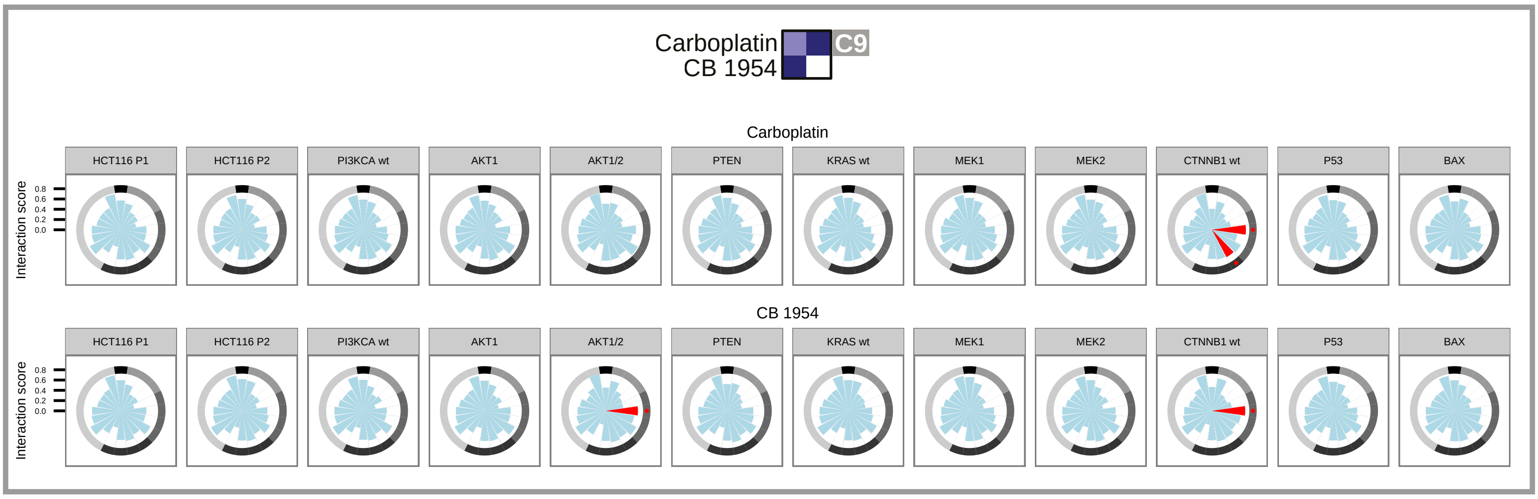

Carboplatin
CB 1954
C9
Carboplatin
CB 1954
HCT116 P1
HCT116 P2
PI3KCA wt
AKT1
AKT1/2
PTEN
KRAS wt
MEK1
MEK2
CTNNB1 wt
P53
BAX
Interaction score
0.8
0.6
0.4
0.2
0.0

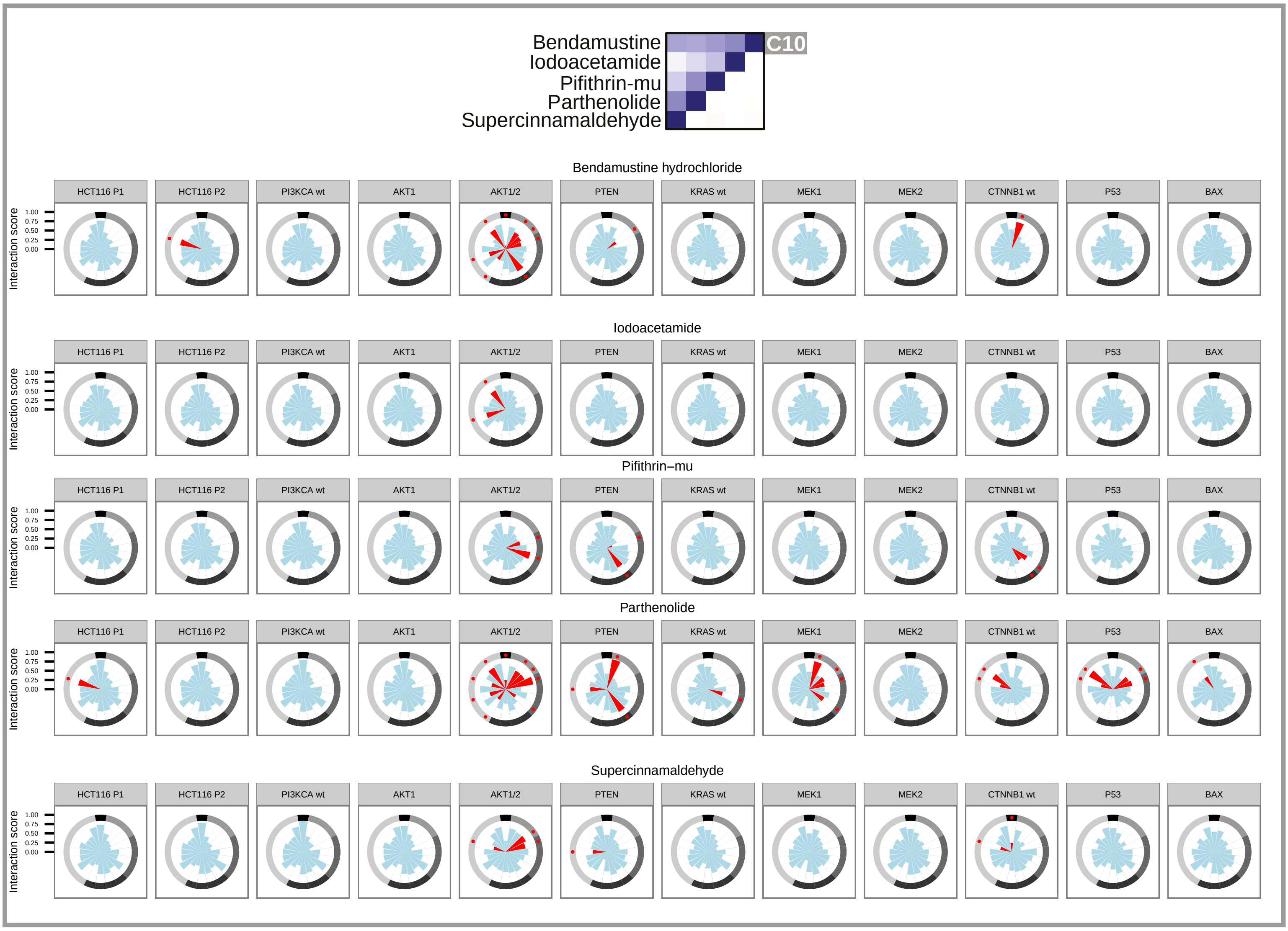

Bendamustine
Iodoacetamide
Pifithrin-mu
Parthenolide
Supercinnamaldehyde
C10
Bendamustine hydrochloride
Iodoacetamide
Pifithrin–mu
Parthenolide
Supercinnamaldehyde
HCT116 P1
HCT116 P2
PI3KCA wt
AKT1
AKT1/2
PTEN
KRAS wt
MEK1
MEK2
CTNNB1 wt
P53
BAX
Interaction score
1.00
0.75
0.50
0.25
0.00

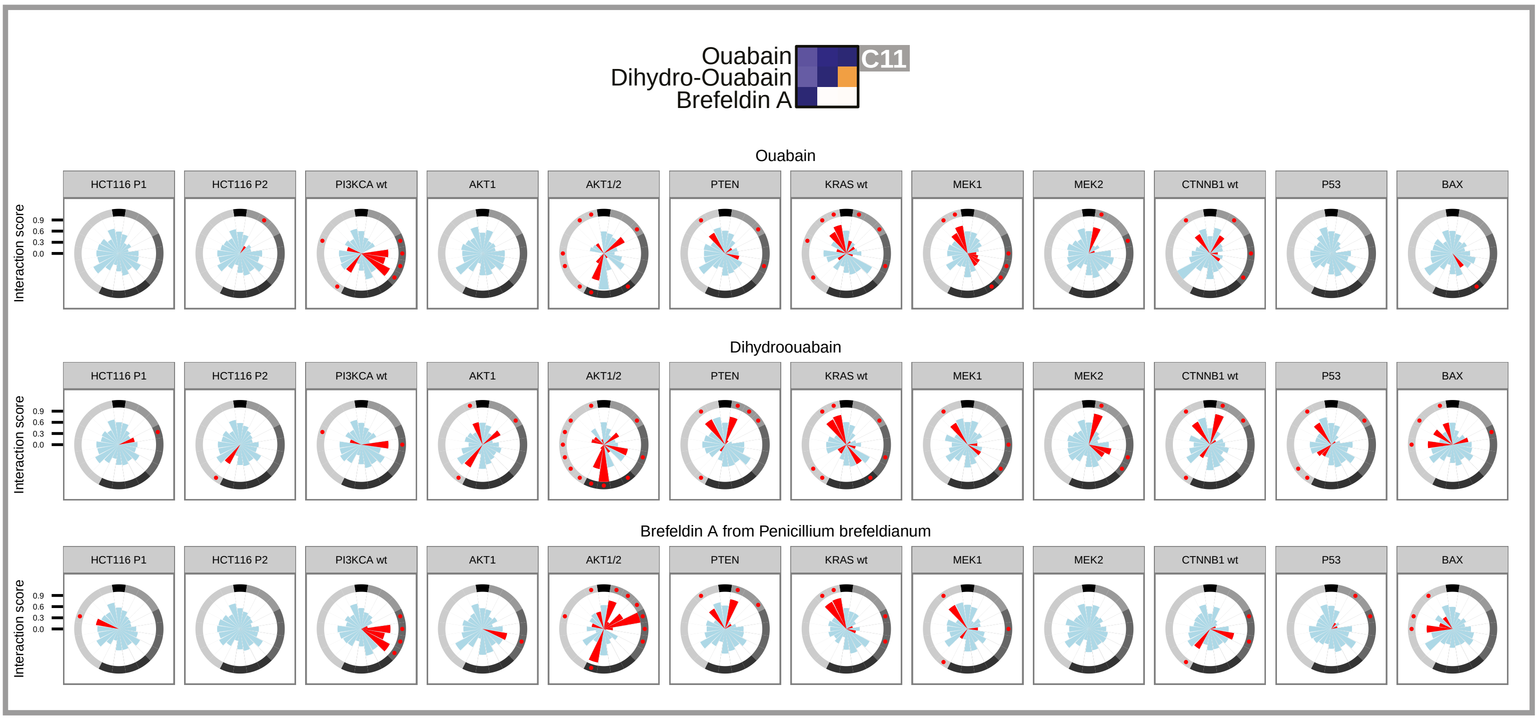
Ouabain
Dihydro-Ouabain
Brefeldin A
C11
Ouabain
Dihydroouabain
Brefeldin A from Penicillium brefeldianum
HCT116 P1 HCT116 P2 PI3KCA wt AKT1 AKT1/2 PTEN KRAS wt MEK1 MEK2 CTNNB1 wt P53 BAX
Interaction score
0.9
0.6
0.3
0.0

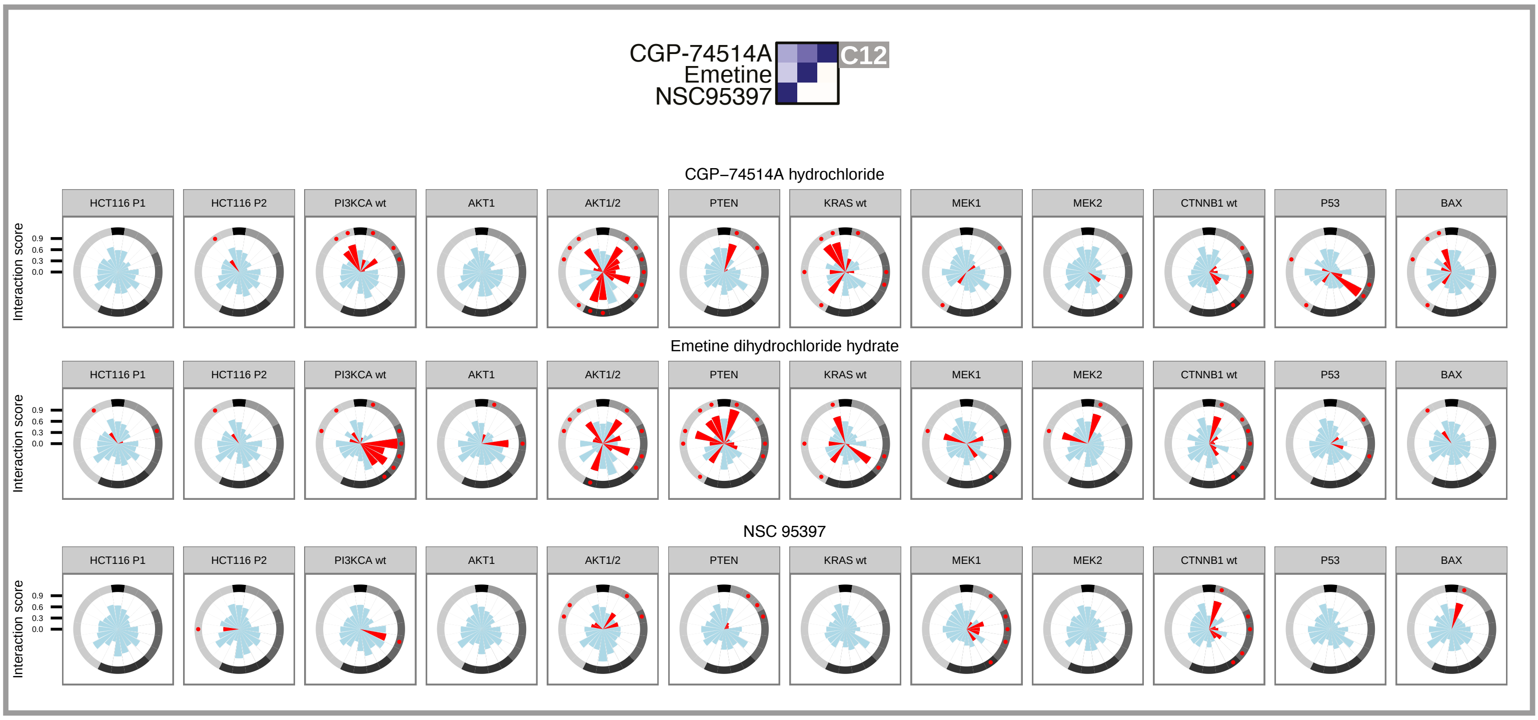
CGP-74514A
Emetine
NSC95397
C12
CGP−74514A hydrochloride
Emetine dihydrochloride hydrate
NSC 95397
HCT116 P1 HCT116 P2 PI3KCA wt AKT1 AKT1/2 PTEN KRAS wt MEK1 MEK2 CTNNB1 wt P53 BAX
Interaction score
0.9
0.6
0.3
0.0

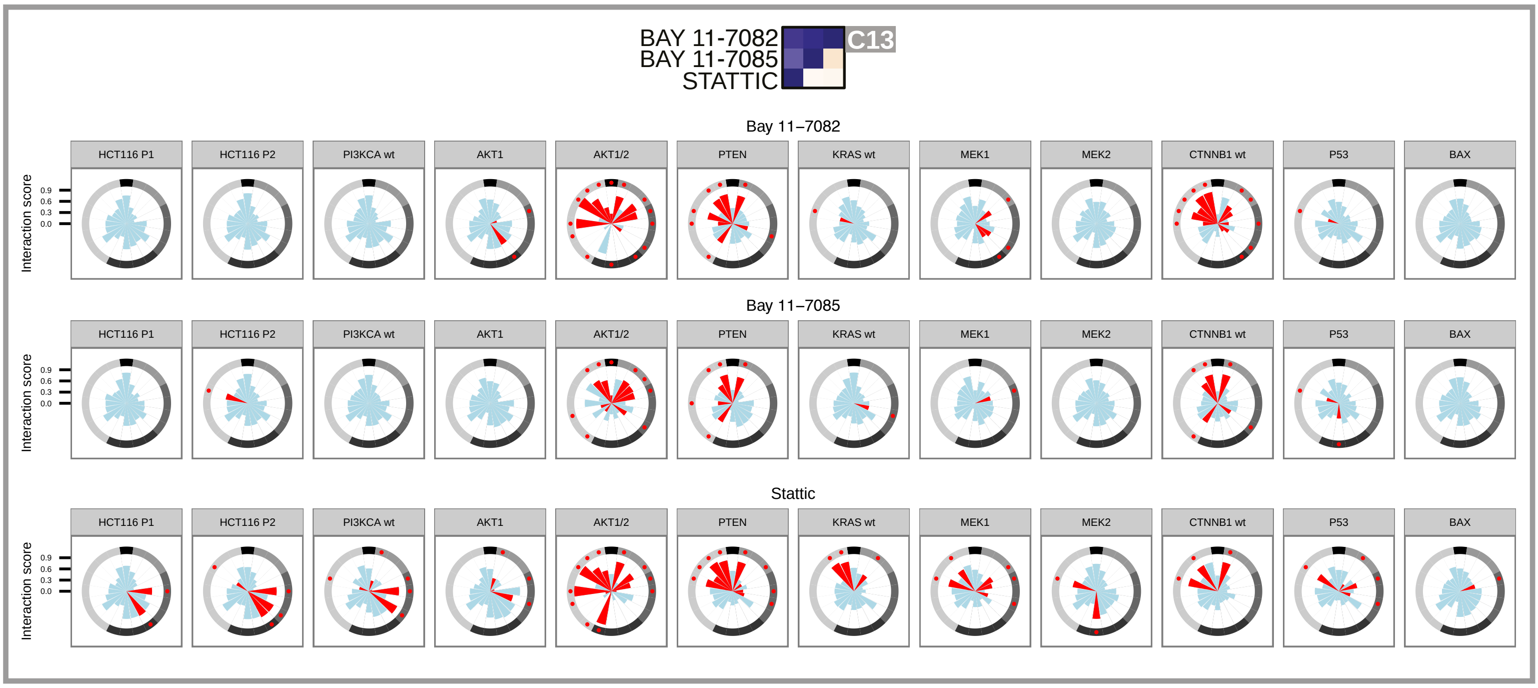
BAY 11-7082
BAY 11-7085
STATTIC
C13
Bay 11−7082
Bay 11−7085
Stattic
HCT116 P1 HCT116 P2 PI3KCA wt AKT1 AKT1/2 PTEN KRAS wt MEK1 MEK2 CTNNB1 wt P53 BAX
Interaction score
0.9
0.6
0.3
0.0

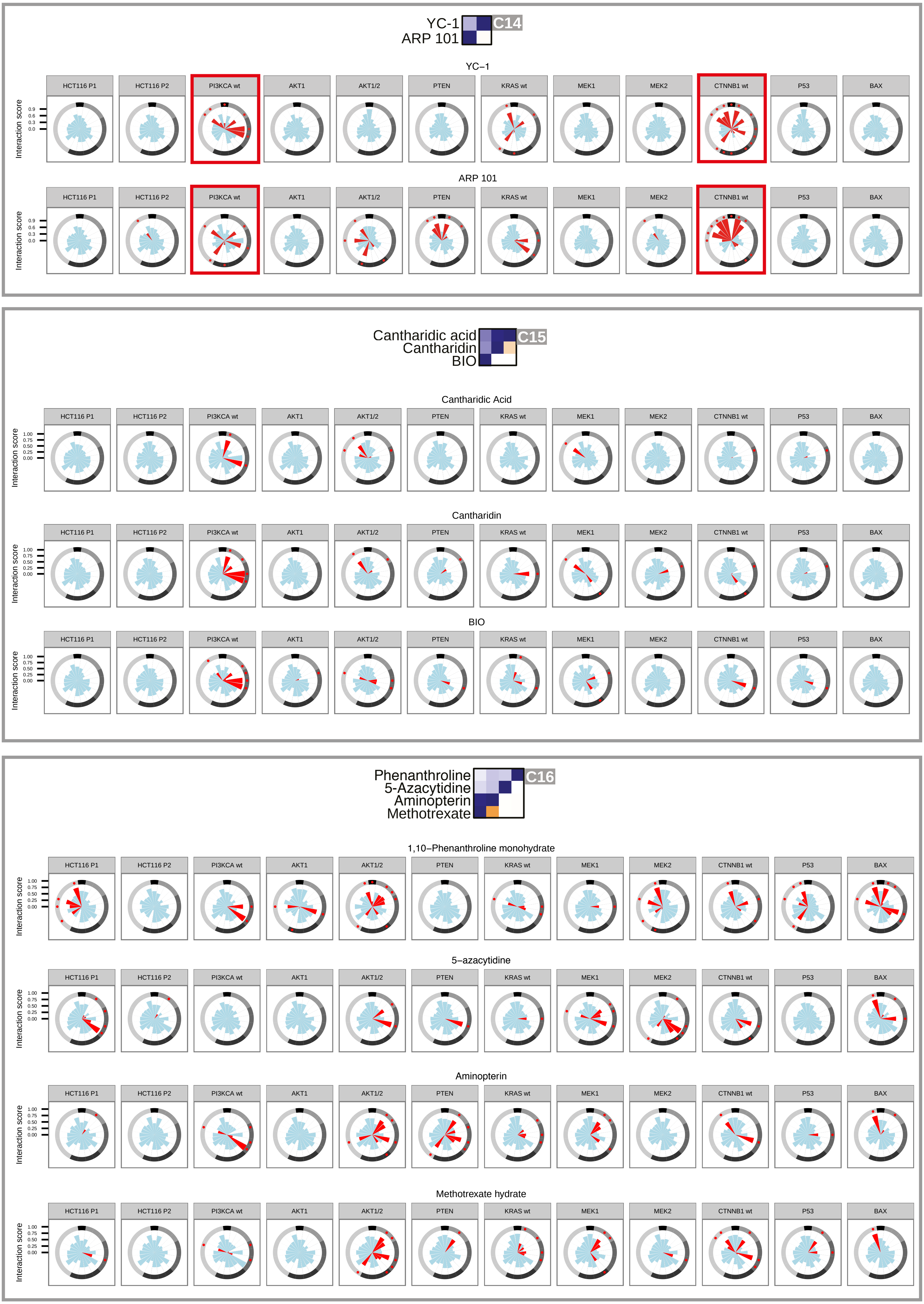
YC-1
ARP 101
C14
YC−1
ARP 101
HCT116 P1
HCT116 P2
PI3KCA wt
AKT1
AKT1/2
PTEN
KRAS wt
MEK1
MEK2
CTNNB1 wt
P53
BAX
Interaction score
0.9
0.6
0.3
0.0
Cantharidic acid
Cantharidin
BIO
C15
Cantharidic Acid
Cantharidin
BIO
1.00
0.75
0.50
0.25
0.00
Phenanthroline
5-Azacytidine
Aminopterin
Methotrexate
C16
1,10−Phenanthroline monohydrate
5−azacytidine
Aminopterin
Methotrexate hydrate

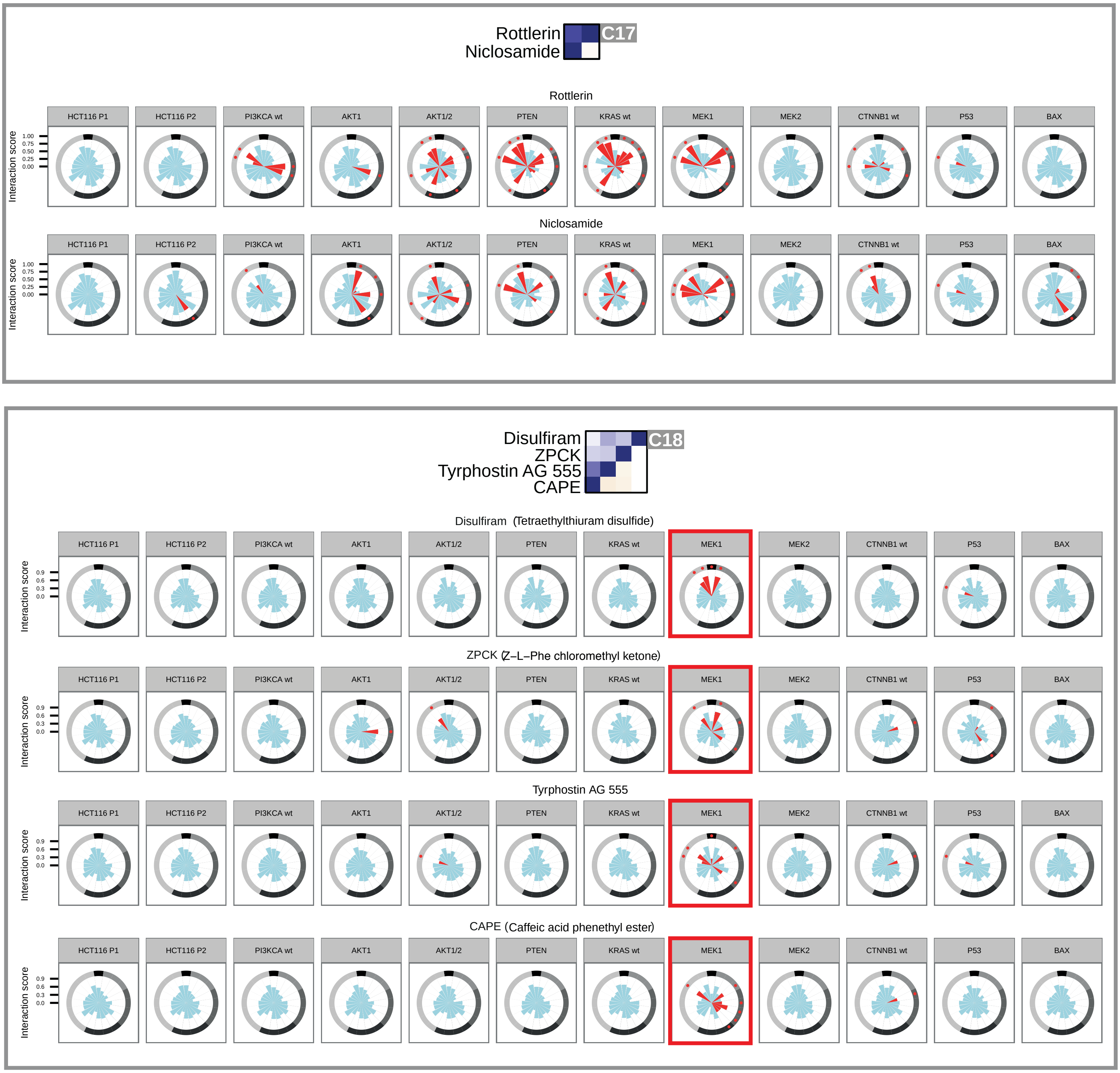

Appendix Figure S9. Association between clustering of compounds as shown in Fig 5A and respective interaction spectra for drugs within clusters.

While in some instances the similarity of drug profiles was a result of coordinated subtle covariation across multiple cell lines and phenotypes, in other cases it was driven by the similarity of distinctive phenotypes of individual cell lines.

For example, the CK2 inhibitors DMAT and TBBz (cluster C5) affected nuclear shape features specifically in cells with only the wildtype copy of PI3KCA (HCT116 PI3KCA wt +/ mt -).

The clustering of the compounds ARP101 and YC-1 in C15 was driven by their distinct phenotypes in cells with only the wildtype copies of CTNNB1 (HCT116 CTNNB1 wt +/ mt -) and PI3KCA (HCT116 PI3KCA wt +/ mt -).

The clustering of compounds in C18 was driven by distinctive interactions with MEK1 KO cells.

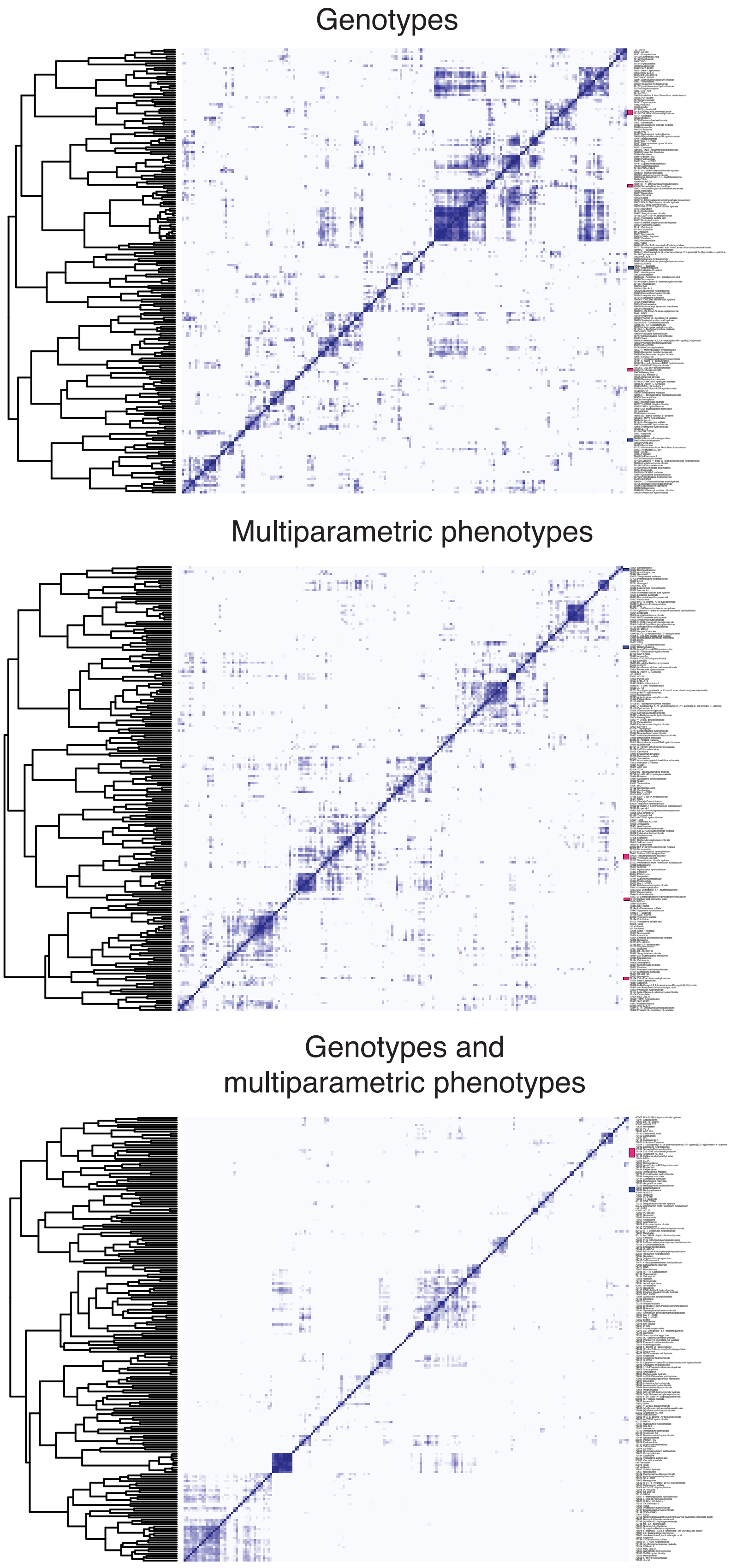

Appendix Figure S10. Integration of phenotypic profiling and chemical-genetic interaction mapping improves resolution.
Unsupervised clustering based on profile correlation using data only on cell number for all twelve genetic backgrounds screened (Genotype), using data from image-based phenotypes of one genetic background (parental HCT116; P2) (Multiparametric phenotypes), or using interaction profiles derived from both genetic backgrounds and image-based phenotypes (Genotypes and multiparametric phenotypes). Each heatmap is ordered based on individual clustering of respective datasets. Blue squares indicate compounds in cluster C4, red squares indicate compounds in cluster C18 (see Fig 5A). In contrast to the low-resolution obtained for genotype or multiparametric phenotype analysis, the combination of phenotypic profiling with chemical-genetic interaction mapping provides distinct high-resolution clusters.

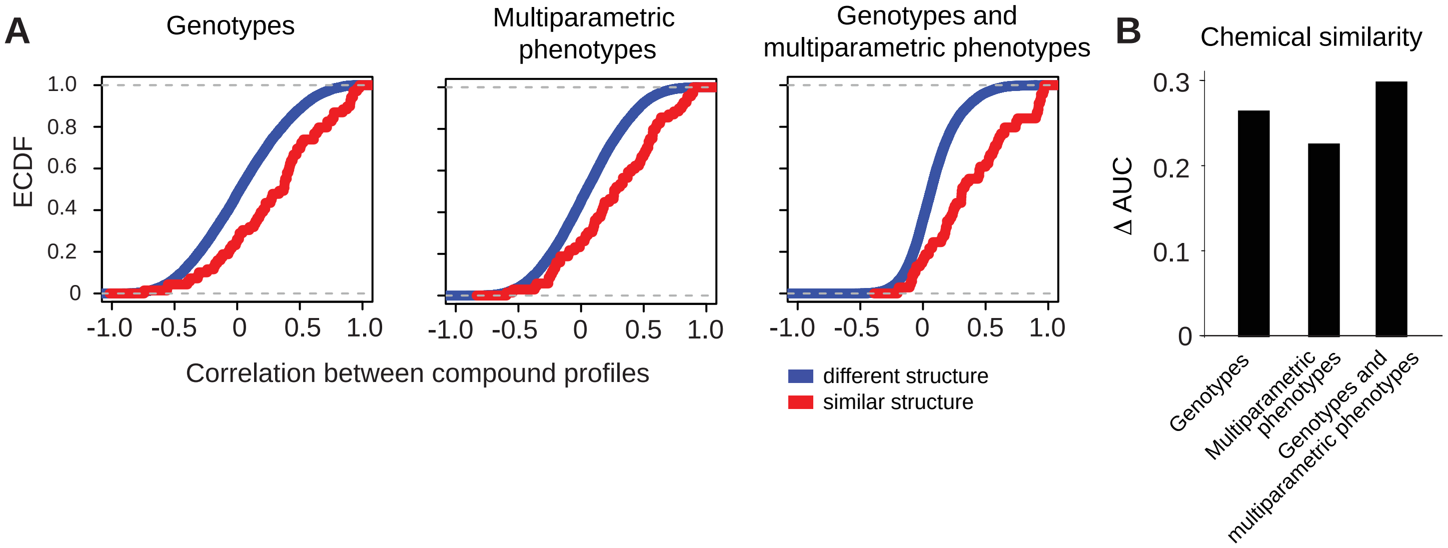

Appendix Figure S11. Image-based chemical-genetic interaction analyses outperforms other methods.
A Compounds that share chemical similarity have a higher correlation of interaction profiles as compared to drugs that do not share chemical similarity, as indicated by the shift of the empirical cumulative density function (ECDF) for shared structures (red curve) compared to non-shared structures (blue curve). Genotype: data only on cell number using 12 genetic backgrounds; Multiparametric phenotypes: data using image-based phenotypes of one genetic background (parental HCT116; P2);
Genotypes and multiparametric phenotypes: interaction profiles derived from both genetic backgrounds and image-based phenotypes.
B Resolution index (Δ AUC) which displays the performance with which each strategy separated drugs that share/do not chemical similarity is shown for aforementioned approaches (see methods for details).

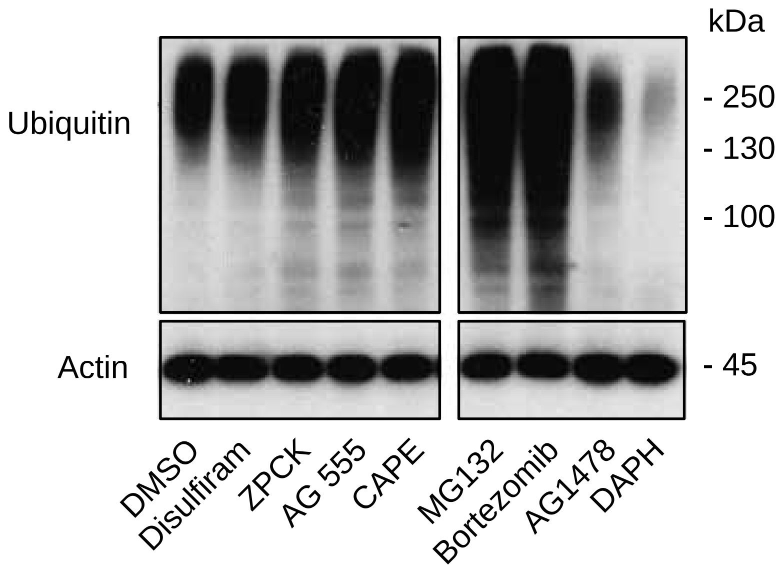

Appendix Figure S12. The EGFR inhibitor tyrphostin AG555 impairs proteasome function. Tyrphostin AG555 increases Ubiquitin abundance. Western blots of total ubiquitin levels after 24 h treatment with compounds at a concentration of 5 µM in HCT116 cells. Tyrphostin AG555 increases the abundance of Ubiquitin, which indicates proteasome inhibition. The proteasome inhibitors MG132 and bortezomib served as positive controls. EGFR inhibitors AG1478 and DAPH did not increase the abundance of ubiquitin. Representative example of 4 independent experiments is shown.
