# Supplementary material for: A chemical–genetic interaction map of small molecules using high‐throughput imaging in cancer cells
Source: Mol Syst Biol. 2015 Dec 28;11(12):846. doi: 10.15252/msb.20156400 (PMC4704494; doi:10.15252/msb.20156400)
Supplement: Supplementary file 2 — Expanded View Figures PDF [file MSB-11-846-s002.pdf]

Expanded View Figures

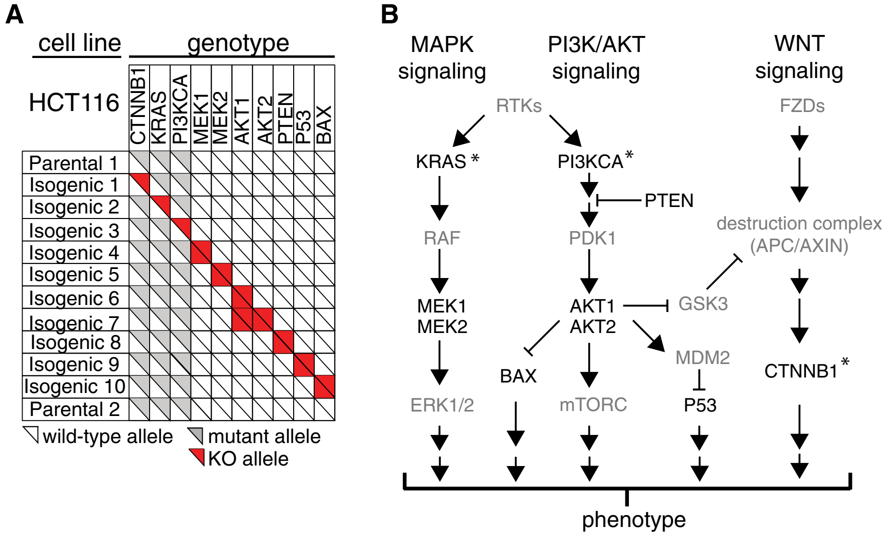

Figure EV1. Overview of genetic backgrounds analyzed in this study.

A A panel of 12 isogenic cell lines derived from HCT116 cells was employed to study pharmacogenomic interactions across complex phenotypes. Genotypes are schematically depicted, including mutant alleles and KO alleles. We included parental HCT116 cells from two different sources.

B The genes that are respectively deleted in the isogenic cell lines are depicted in the context of simplified intracellular signaling pathways. Asterisks indicate genes that are mutated in HCT116 cells.

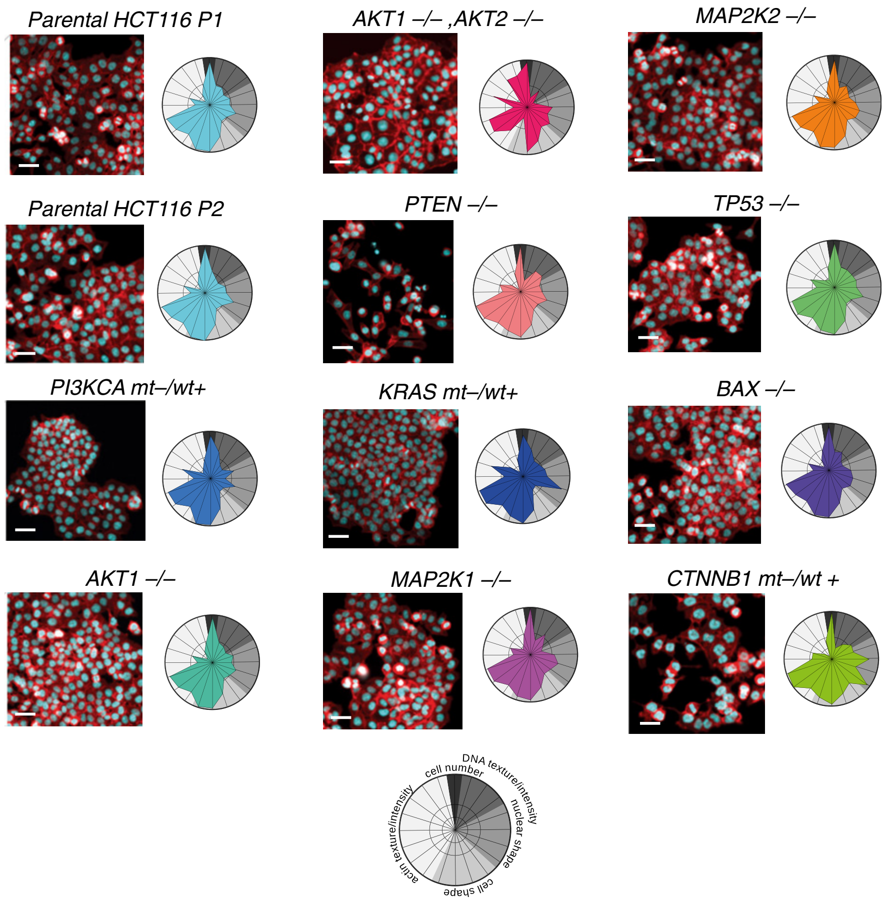

Figure EV2. Phenotypes of the twelve isogenic cell lines employed.

Isogenic KO cell lines show divergent phenotypes; actin, red; DNA, cyan. Phenoprints for the isogenic cell lines are depicted. Scale bars = 20 μm.

© 2015 The Authors

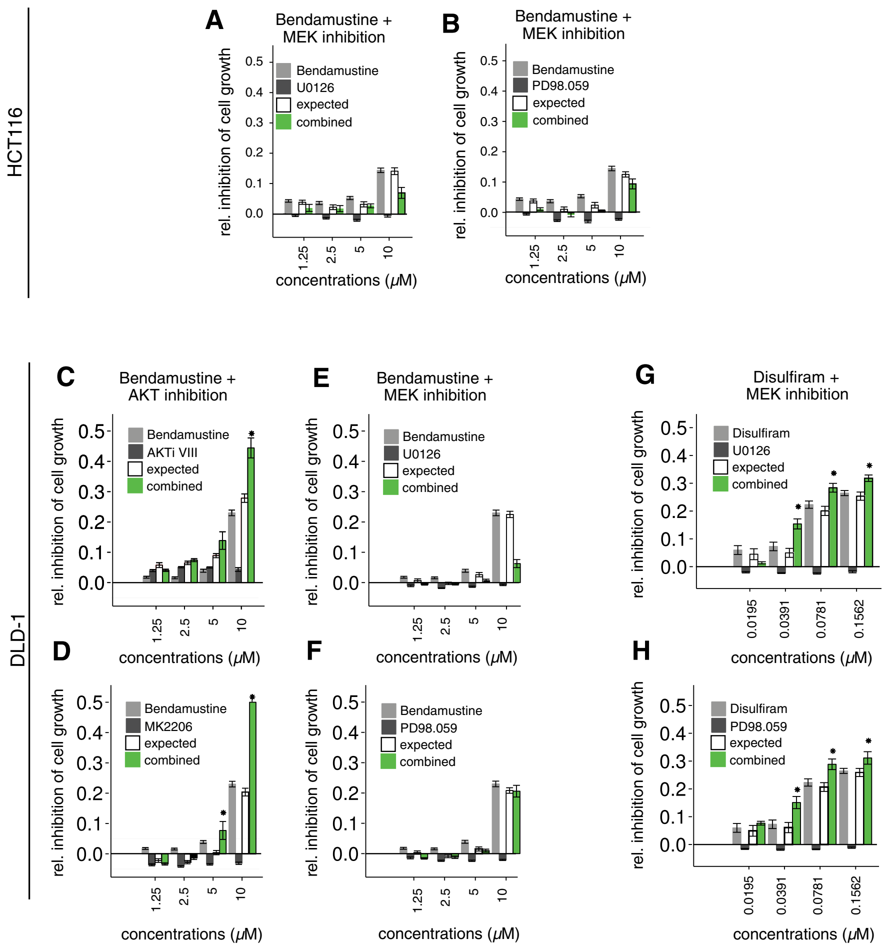

Figure EV3. Predicting effective drug combinations from phenotypic pharmacogenomic interactions.

A, B In contrast to the combination of bendamustine and AKT inhibitors (Fig 4A), the combination of bendamustine and MEK1/2 inhibitors (U0126, PD98.059) is not effective in HCT116 cells.

C, D Efficacy between bendamustine and Akt inhibitors in DLD-1 colon cancer cells. Pharmacological mimicking the *AKT1/2* double KO genetic background revealed that the combination of bendamustine and Akt inhibitors (AKTiVIII as well MK2206) resulted in significantly stronger cell killing compared with either drug alone.

E, F The effect was specific for bendamustine and Akt inhibition since no efficacy was observed for the combination of bendamustine and MEK1/2 inhibitors (U0126, PD98.059).

G, H Efficacy between disulfiram and MEK1/2 inhibitors in DLD-1 colon cancer cells. Pharmacological mimicking the *MEK1* KO genetic background revealed that the combination of disulfiram and MEK1/2 inhibitors (U0126, PD98.059) resulted in significantly stronger cell killing in DLD-1 colon cancer cells compared with either drug alone.

Data information: Error bars, means ± s.e.m. $n \geq 3$ of at least three independent experiments that determined cell viability using the CellTiterGlo assay. BI significance is shown, $^*P < 0.05$.

© 2015 The Authors
