# Supplementary material for: A chemical–genetic interaction map of small molecules using high‐throughput imaging in cancer cells
Source: Mol Syst Biol. 2015 Dec 28;11(12):846. doi: 10.15252/msb.20156400 (PMC4704494; doi:10.15252/msb.20156400)
Supplement: Supplementary file 4 — Table EV2 [file MSB-11-846-s004.docx]

**Table EV2. Detailed description of cluster and respective compounds highlighted in Figure 5A.**

| Cluster | **Compounds** | **Target selectivity/**  **Biological process** | **Associated references** |
| --- | --- | --- | --- |
| C1 | Podophyllotoxin  Colchicine  Vinblastine  Vincristine  Taxol  CHM-1 hydrate  Nocodazol | microtubule | - |
| C2 | U0126  PD98,059 | MEK1/2 | - |
| C3 | PD169316  SB202190 | p38 MAPK | - |
| C4 | Betamethasone  Beclomethasone | steroidal anti-inflammatory  glucocorticoids | - |
| C5 | DMAT  TBBz | CK2 | - |
| C6 | 5’dFUrd  5-FU | DNA/RNA metabolism | (Lum et al., 2004) this study used yeast chemo-genomics and identified that 5-FU preferentially interferes with RNA metabolism |
| C7 | Etoposide  Amsacrine  NU2058  Ara-c  Cyclo-c | Topoisomerase  Topoisomerase  CDK2  DNA metabolism  DNA metabolism | (Iorio et al., 2010) this study used transcriptional profiling and identified a link between CDK2 inhibitors and topoisomerase inhibitors;  (Pourquier et al., 2000) this study identified a link between ara-c and topoisomerase |
| C8 | Mitoxantrone  Camptothecin  Thapsigargin  Calcimycin | Topoisomerase  Topoisomerase  Increased intracellular calcium levels (SERCA inhibitor)  Increased intracellular calcium levels (Ca ionophore) | (Bertrand et al., 1991) this study identified links between calcium levels and topoisomerase inhibitor activity |
| C9 | Carboplatin  CB1954 | DNA alkylating | - |
| C10 | Bendamustine  Iodoacetamine  Pifithrin-µ  Parthenolide  Supercinnamaldehyde | DNA alkylating  Alkylating  P53  IKKbeta, Cysteine modifying  TRPA1, Cysteine modifying | (Leoni et al., 2008) this study showed that bendamustine has a different mode-of-action as compared to standard DNA alkylating agents;  (Strom et al., 2006) this study identified pifithrin-µ as a compound that interferes with p53 signaling;  (Kwok et al., 2001) this study showed that parthenolide interferes with IKKbeta due to modifying cysteine residues;  (Macpherson et al., 2007) this study identified compounds, including supercinnamaldehyde and iodoacetamide, that interfere with TRPA1 due to modifying cysteine residues |
| C11 | Ouabain  Dihydro-Ouabain  Brefeldin | Na/K ATPase  Na/K ATPase  Golgi/ER | (Farr et al., 2009) this study showed Golgi mediated transport of Na/K pump subunits to the plasma membrane |
| C12 | CGP-74514A  Emetine  NSC95297 | CDK1  Translation  Cdc25 | (Boutros et al., 2007) this review summarizes the interplay between CDK1, cdc25, and translational control during the cell cycle |
| C13 | BAY11-7082  BAY11-7085  STATTIC | IKB-α  IKB-α  STAT3 | (Grivennikov and Karin, 2010) this review summarizes the links between NFκB signaling and JAK/STAT signaling |
| C14 | YC-1  ARP101 | guanylyl cyclase activator  MMP2 | (Lubbe et al., 2009) this study showed a link between guanylyl cyclase activity and MMPs |
| C15 | Cantharidic acic  Cantharidin  BIO | PP2A  PP2A  GSK3 > CDKs | (Hernández et al., 2010) this study showed a link between PP2 and GSK3 |
| C16 | Phenanthroline  5-azacytidine  Aminopterin  Methotrexate | Iron chelator  DNA methyltransferase  Folate metabolism/DHFR  Folate metabolism/DHFR | (Oppenheim et al., 2000)(Crider et al., 2012) this study and review summarize links between iron metabolism, folate metabolism and DNA methylation |
| C17 | Rottlerin  Niclosamide | PKCδ  Oxidative phosphorylation | (MacDonald et al., 2006)(Maioli et al., 2012) this study and review highlight that rotterlin does not inhibit PKC and interferes with oxidative phosphorylation;  (Weinbach and Garbus, 1969) this study showed that niclosamide uncouples oxidative phosphorylation |
| C18 | Disulfiram  ZPCK  Tyrphostatin AG555  CAPE | ALDH  Chymotrypsin-A  EGFR  NFκB | (Kisselev et al., 2012)(Grivennikov and Karin, 2010) these reviews address proteasome functions and NFκB signaling |
| Arrow 1 | Ara-c  5-Azacytidine | DNA metabolism  DNA methyltransferase | (Christman, 2002) this review summarizes the DNA methyltransferase activity of 5-azacytidine |
| Arrow 2 | BIO  Indirubin-3-oxim | GSK3 > CDKs  CDKs > GSK3 | (Meijer et al., 2003) this study revealed that indirubin derivatives can inhibit GSK3 as well as CDKs and showed that BIO preferentially inhibits GSK3 whereas indirubin-3-oxime preferentially inhibits CDKs |

**References:**

Bertrand, R., Kerrigan, D., Sarang, M., and Pommier, Y. (1991). Cell death induced by topoisomerase inhibitors. Role of calcium in mammalian cells. Biochem. Pharmacol. *42*, 77–85.

Boutros, R., Lobjois, V., and Ducommun, B. (2007). CDC25 phosphatases in cancer cells: key players? Good targets? Nat. Rev. Cancer *7*, 495–507.

Christman, J.K. (2002). 5-Azacytidine and 5-aza-2’-deoxycytidine as inhibitors of DNA methylation: mechanistic studies and their implications for cancer therapy. Oncogene *21*, 5483–5495.

Crider, K.S., Yang, T.P., Berry, R.J., and Bailey, L.B. (2012). Folate and DNA methylation: a review of molecular mechanisms and the evidence for folate’s role. Adv. Nutr. Bethesda Md *3*, 21–38.

Farr, G.A., Hull, M., Mellman, I., and Caplan, M.J. (2009). Membrane proteins follow multiple pathways to the basolateral cell surface in polarized epithelial cells. J. Cell Biol. *186*, 269–282.

Grivennikov, S.I., and Karin, M. (2010). Dangerous liaisons: STAT3 and NF-kappaB collaboration and crosstalk in cancer. Cytokine Growth Factor Rev. *21*, 11–19.

Hernández, F., Langa, E., Cuadros, R., Avila, J., and Villanueva, N. (2010). Regulation of GSK3 isoforms by phosphatases PP1 and PP2A. Mol. Cell. Biochem. *344*, 211–215.

Iorio, F., Bosotti, R., Scacheri, E., Belcastro, V., Mithbaokar, P., Ferriero, R., Murino, L., Tagliaferri, R., Brunetti-Pierri, N., Isacchi, A., et al. (2010). Discovery of drug mode of action and drug repositioning from transcriptional responses. Proc. Natl. Acad. Sci. U. S. A. *107*, 14621–14626.

Kisselev, A.F., van der Linden, W.A., and Overkleeft, H.S. (2012). Proteasome inhibitors: an expanding army attacking a unique target. Chem. Biol. *19*, 99–115.

Kwok, B.H., Koh, B., Ndubuisi, M.I., Elofsson, M., and Crews, C.M. (2001). The anti-inflammatory natural product parthenolide from the medicinal herb Feverfew directly binds to and inhibits IkappaB kinase. Chem. Biol. *8*, 759–766.

Leoni, L.M., Bailey, B., Reifert, J., Bendall, H.H., Zeller, R.W., Corbeil, J., Elliott, G., and Niemeyer, C.C. (2008). Bendamustine (Treanda) displays a distinct pattern of cytotoxicity and unique mechanistic features compared with other alkylating agents. Clin. Cancer Res. Off. J. Am. Assoc. Cancer Res. *14*, 309–317.

Lubbe, W.J., Zuzga, D.S., Zhou, Z., Fu, W., Pelta-Heller, J., Muschel, R.J., Waldman, S.A., and Pitari, G.M. (2009). Guanylyl cyclase C prevents colon cancer metastasis by regulating tumor epithelial cell matrix metalloproteinase-9. Cancer Res. *69*, 3529–3536.

Lum, P.Y., Armour, C.D., Stepaniants, S.B., Cavet, G., Wolf, M.K., Butler, J.S., Hinshaw, J.C., Garnier, P., Prestwich, G.D., Leonardson, A., et al. (2004). Discovering modes of action for therapeutic compounds using a genome-wide screen of yeast heterozygotes. Cell *116*, 121–137.

MacDonald, M.L., Lamerdin, J., Owens, S., Keon, B.H., Bilter, G.K., Shang, Z., Huang, Z., Yu, H., Dias, J., Minami, T., et al. (2006). Identifying off-target effects and hidden phenotypes of drugs in human cells. Nat. Chem. Biol. *2*, 329–337.

Macpherson, L.J., Dubin, A.E., Evans, M.J., Marr, F., Schultz, P.G., Cravatt, B.F., and Patapoutian, A. (2007). Noxious compounds activate TRPA1 ion channels through covalent modification of cysteines. Nature *445*, 541–545.

Maioli, E., Torricelli, C., and Valacchi, G. (2012). Rottlerin and cancer: novel evidence and mechanisms. ScientificWorldJournal *2012*, 350826.

Meijer, L., Skaltsounis, A.-L., Magiatis, P., Polychronopoulos, P., Knockaert, M., Leost, M., Ryan, X.P., Vonica, C.A., Brivanlou, A., Dajani, R., et al. (2003). GSK-3-selective inhibitors derived from Tyrian purple indirubins. Chem. Biol. *10*, 1255–1266.

Oppenheim, E.W., Nasrallah, I.M., Mastri, M.G., and Stover, P.J. (2000). Mimosine is a cell-specific antagonist of folate metabolism. J. Biol. Chem. *275*, 19268–19274.

Pourquier, P., Takebayashi, Y., Urasaki, Y., Gioffre, C., Kohlhagen, G., and Pommier, Y. (2000). Induction of topoisomerase I cleavage complexes by 1-β-d-arabinofuranosylcytosine (ara-C) in vitro and in ara-C-treated cells. Proc. Natl. Acad. Sci. *97*, 1885–1890.

Strom, E., Sathe, S., Komarov, P.G., Chernova, O.B., Pavlovska, I., Shyshynova, I., Bosykh, D.A., Burdelya, L.G., Macklis, R.M., Skaliter, R., et al. (2006). Small-molecule inhibitor of p53 binding to mitochondria protects mice from gamma radiation. Nat. Chem. Biol. *2*, 474–479.

Weinbach, E.C., and Garbus, J. (1969). Mechanism of action of reagents that uncouple oxidative phosphorylation. Nature *221*, 1016–1018.
