# Supplementary material for: A chemical–genetic interaction map of small molecules using high‐throughput imaging in cancer cells
Source: Mol Syst Biol. 2015 Dec 28;11(12):846. doi: 10.15252/msb.20156400 (PMC4704494; doi:10.15252/msb.20156400)
Supplement: Supplementary file 6 — Code EV1 [file MSB-11-846-s006.pdf]

A chemical-genetic interaction map of small molecules using high-throughput imaging in cancer cells

Marco Breinig, Felix A. Klein, Wolfgang Huber and Michael Boutros

Accepted for publication at Molecular Systems Biology

Felix A. Klein

European Molecular Biology Laboratory (EMBL),
Heidelberg, Germany
felix.klein@embl.de

November 29, 2015

Contents

1 Introduction 2
1.1 Accessing the data . . . 2

2 Image and data processing 3
2.1 Image processing on cluster . . . 3
2.2 Data extraction and conversion . . . 5
2.3 Annotation . . . 5
2.4 Removal/Filtering of empty wells and features . . . 6
2.5 Generalized logarithm transformation . . . 7

3 Feature selection and calculation of interactions 8
3.1 Quality control of features . . . 8
3.2 Feature selection by stability . . . 9
3.3 Calculation of interactions . . . 11

4 Display of phenotypes as 'phenoprints' 13

5 Display of interactions as star plots. 16
5.1 Scaled to the range of 0 to 1 . . . 16
5.2 Using the absolute values of an interaction. . . . 21

6 Clustering of cell lines 26
6.1 Clustering cell lines based on features . . . 26
6.2 Clustering cell lines based on interaction terms . . . 27

7 Compound - cell line interaction network 29
7.1 Extract significant interactions for visualization. . . . 29
7.1.1 Removing controls from the interactions . . . 31
7.1.2 Pleiotropic degree . . . 33
7.1.3 Grouping of features into feature classes. . . . 37
7.1.4 Interaction map export for Cytoscape . . . 38

8 Heat maps of interaction profiles 41

9 Clustering of interaction profiles 43

9.1 Clustering of interaction profiles using the filtered data . . . 43
9.1.1 Reordered dendrogram . . . 43
9.2 Structural similarity of compounds . . . 46
9.2.1 Chemical similarity heat map ordered by interaction profile similarity . . . 47
9.2.2 Combined cluster heat map . . . 48
9.3 Clustering of interaction profiles using the filtered data of a single parental cell line . . . 50
9.4 Clustering of interaction profiles using the filtered cell number as feature. . . . 51

10 Correlation between interaction profiles of shared drug targets **54**
10.1 Using the target selectivity for grouping . . . 54
10.2 Using the chemical similarity for grouping . . . 57

11 Follow-up: Drug combinations **64**
11.1 Quality control . . . 66
11.2 Interactions / Synergies . . . 69
11.2.1 Analysis functions . . . 69
11.2.2 Investigating drug synergies . . . 73
11.2.3 Investigating drug synergies, DLD cell line . . . 76

12 Follow-up: Proteasome inhibition assay **81**
12.0.4 Data processing and quality control . . . 82
12.0.5 Viability normalization . . . 85
12.0.6 Proteasome activity normalization . . . 86
12.0.7 Result summary . . . 87

13 Session Info **90**

1 Introduction

This document is associated with the R package *PGPC*, which contains the input data and the R code for the statistical analysis presented in the following paper:

Systems pharmacology by integrating image-based phenotypic profiling and chemical-genetic interaction mapping
Marco Breinig, Felix A. Klein, Wolfgang Huber, and Michael Boutros
in preparation

The figures of the paper and the intermediate results are reproduced in this vignette. Intermediate results are stored in the folder "result" created in the current working directory.

```
library("PGPC")
if(!file.exists(file.path("result","data")))
  dir.create(file.path("result","data"),recursive=TRUE)
if(!file.exists(file.path("result","Figures")))
  dir.create(file.path("result","Figures"),recursive=TRUE)
```

To reduce the computational effort required, the package contains some analysis results already in precomputed form. However, the corresponding code junks are contained in this document, and they can be executed to recompute all results except the initial image analysis. For the inital image analysis approximately 3 TB of image data were processed. We provide the analysis of one single well to show the steps used in the initial image analysis.

1.1 Accessing the data

The final data object can be loaded by

```
data("interactions", package="PGPC")
```

A description of this object is given in the following Section together with further data objects that are contained within this package. Additional information on the objects can be obtained from the R documentation by invoking the help page.

```
?interactions
```

The package contains the following data objects:

`ftrs`
: *data.frame* with the feature data extracted from the images using imageHTS.

`datamatrixTransformed`
: Feature data represented as a *list* containing *array* `D` and *list* `anno`. `D` is a four-dimensional *array* of the filtered feature data that passed quality control. The dimensions represent:
  1. drug
  2. cell line
  3. replicate
  4. feature

  To select the second feature of the first replicate of the third drug of the fourth cell line use:
  `value=datamatrixfull$D[3,4,1,2]`
  To select all values of the drugs, cell lines and replicates for the first feature use:
  `values=datamatrixfull$D[, , ,1]`
  `anno` is annotation of the dimensions of `D`, represented as a *list* containing a *data.frame* named `drug`, a *data.frame* named `line`, a *vector* named `repl` and a *vector* named `ftr`. Use `$` to access the elements (e. g. `anno$drug`).

`selected`
: *list* of 4 elements containing the results of the feature selection. The elements are, the *vector* `selected` of selected features, the *vector* `correlation` of their correlation, the *vector* `ratioPositive` with the fraction of positive correlations for each iteration and a *list* `correlationAll` which contains the correlations of all features at each iteration step.

`interactions`
: *list* containing the *list* `anno`, *array* `D`, *array* `res`, *list* `effect` and *array* `pVal`.
  `res` is a four-dimensional *array* of the interaction terms and has the same dimensions as `D`. The dimensions represent:
  1. drug
  2. cell line
  3. replicate
  4. feature

  `effect` contains the drug and cell line effect as three-dimensional *array*
  `drug` and `line` with the following dimensions:
  1. drug/cell line
  2. replicate
  3. feature

  `pVal` is an *array* containing the p-values, adjusted p-values and correlation between replicates of interactions. The dimensions represent:
  1. drug
  2. cell line
  3. p-value, adjusted p-value, correlation
  4. feature

2 Image and data processing

2.1 Image processing on cluster

The images were processed using the R packages *imageHTS* and *EBImage* [1] adapting previous strategies [2, 3, 4]. The following code shows the processing script that was used to segment the images and extract features. The calculations were parallelized by splitting the wells defined by the unique identifiers `unames` into different processes. The object `ftrs` contains all the extracted features and is included in this package.

In the following code `serverURL` is the path to the folder which contains the example files and images in the local installation of the *PGPC* package. `localPath` is a temporary local working directory for the results. If you want to keep the results beyound the R session change this path to a directory on your machine. If you want to run this

example code approximatly 150 MB free space are required for the 1 example wells. For the whole screen of 36864 wells approximately 5 TB were required. The required annotation and image files are automatically retrieved from the provided serverURL if they are not found in the local directory.

```
localPath = tempdir()
serverURL = system.file("extdata", package = "PGPC")

imageConfFile = file.path("conf", "imageconf.txt")

## circumvent memory problem on 32bit windows by segementing only spot 1.
if(.Platform$OS.type == "windows" & R.Version()$arch == "i386")
    imageConfFile = file.path("conf", "imageconf_windows32.txt")

x = parseImageConf(imageConfFile, localPath=localPath, serverURL=serverURL)

unames = getUnames(x) ## select all wells for processing
unames = c("045-01-C23") ## select single well for demonstration

segmentWells(x, unames, file.path("conf", "segmentationpar.txt"))

PGPC:::extractFeaturesWithParameter(x,
                                    unames,
                                    file.path("conf", "featurepar.txt"))

summarizeWellsExtended(x, unames, file.path("conf", "featurepar.txt"))

## PGPC:::mergeProfiles(x)  only needed if wells were processed in parallel

ftrs = readHTS(x, type="file",
               filename=file.path("data", "profiles.tab"),
               format="tab")
```

Images were processed in parallel on a cluster and the corresponding session info is provided here:

```
R version 3.0.1 (2013-05-16)
Platform: x86_64-unknown-linux-gnu (64-bit)

locale:
 [1] LC_CTYPE=en_US.UTF-8       LC_NUMERIC=C               LC_TIME=en_US.UTF-8
 [4] LC_COLLATE=en_US.UTF-8     LC_MONETARY=en_US.UTF-8    LC_MESSAGES=en_US.UTF-8
 [7] LC_PAPER=C                 LC_NAME=C                  LC_ADDRESS=C
[10] LC_TELEPHONE=C             LC_MEASUREMENT=en_US.UTF-8 LC_IDENTIFICATION=C

attached base packages:
[1] parallel  grid      stats     graphics  grDevices utils     datasets  methods   base

other attached packages:
 [1] PGPC_0.1.1     SearchTrees_0.5.2   ggplot2_0.9.3.1     imageHTS_1.10.0
 [5] cellHTS2_2.24.0      locfit_1.5-9.1       hwriter_1.3          vsn_3.28.0
 [9] genefilter_1.42.0    EBImage_4.2.1        LSD_2.5              ellipse_0.3-8
[13] schoolmath_0.4       colorRamps_2.3       limma_3.16.4         splots_1.26.0
[17] geneplotter_1.38.0   lattice_0.20-15      annotate_1.38.0      AnnotationDbi_1.22.5
[21] Biobase_2.20.0       BiocGenerics_0.6.0   gplots_2.11.0.1      MASS_7.3-26
[25] KernSmooth_2.23-10   caTools_1.14         gdata_2.12.0.2       gtools_2.7.1
[29] RColorBrewer_1.0-5   BiocInstaller_1.10.2

loaded via a namespace (and not attached):
 [1] abind_1.4-0          affy_1.38.1          affyio_1.28.0        bitops_1.0-5
 [5] Category_2.26.0      class_7.3-7          colorspace_1.2-2     DBI_0.2-7
 [9] dichromat_2.0-0      digest_0.6.3         e1071_1.6-1          graph_1.38.0
```

```
[13] GSEABase_1.22.0       gtable_0.1.2          IRanges_1.18.1        jpeg_0.1-4
[17] labeling_0.1          munsell_0.4           plyr_1.8              png_0.1-4
[21] prada_1.36.0          preprocessCore_1.22.0 proto_0.3-10          RBGL_1.36.2
[25] reshape2_1.2.2        robustbase_0.9-7      rrcov_1.3-3           RSQLite_0.11.4
[29] scales_0.2.3          splines_3.0.1         stats4_3.0.1          stringr_0.6.2
[33] survival_2.37-4       tiff_0.1-4            tools_3.0.1           XML_3.96-1.1
[37] xtable_1.7-1
```

2.2 Data extraction and conversion

The `ftrs` object is included in this vignette and the following code junks can be executed to generate the results described in the following.

The extracted feature data are represented in *data.frame* where each row corresponds to a well in the screen defined by the unique identifier `uname` in the first column. All remaining columns represent the extracted features. There are 36864 wells (12 cell lines * 2 replicates * 4 plates * 384 wells).

```
data("ftrs", package="PGPC")
dim(ftrs)
ftrnames = colnames(ftrs[,-1])
```

For further processing the data are transformed into an array.

```
makeArray <- function(df, nLines=12, nWells=384, nPlates=4, nRep=2){
  ## df = list of features ordered by unames
  if(! nLines*nWells*nPlates*nRep == nrow(df))
    stop("Wrong dimensions!")

  ## order by replicate
  replicate <- rep(c(rep(1, nWells), rep(2, nWells)),
                   nrow(df)/(nWells*nRep))
  df = df[order(replicate),]

  ## create matrix
  D = as.matrix(df[,-1])

  ## convert Matrix: x=Gene/wells, y=Celllines, z=replicates
  dim(D) = c(nPlates*nWells,nLines,nRep,ncol(D))
  invisible(D)
}

D = makeArray(ftrs)
dim(D)

## free space and save full data matrix
rm("ftrs")
datamatrixfull = list(D = D, anno=list(ftr=ftrnames))
save(datamatrixfull, file=file.path("result","data","datamatrixfull.rda"),
     compress="xz")
rm("datamatrixfull")
```

2.3 Annotation

The drug annotation was given for the plate format and had to be converted to fit the matrix format. The `Content` is set to `"empty"` if `GeneID` is `"no treatment"`.

```
drug = read.delim(
  system.file("extdata",
```

```
                "conf",
                "Changed_LOPAC3_384_convert96er_GeneID_updated_Selectivity.txt",
    package = "PGPC"),
  stringsAsFactors=FALSE,
  encoding="latin1")

drug$Plate = NULL
drug = unique(drug)
drug$Content[drug$GeneID == "no treatment"] = "empty"
drug$Content[grep("ctrl", drug$GeneID)] = drug$GeneID[grep("ctrl", drug$GeneID)]
drug$Content[grep("neg", drug$GeneID)] = drug$GeneID[grep("neg", drug$GeneID)]
```

The annotation of the cell lines contains the name, mutation and start plate for each cell line.

```
line <- data.frame(name = c('02-006', '02-031',
                     '02-004', '104-009', 'PAR007',
                     '104-001', '104-004', '104-007',
                     '104-008', '02-008', '02-030', 'PAR001'),
                   mutationDetailed =  c('AKT1-/-&AKT2-/-', 'MEK2-/-',
                     'AKT1-/-', 'CTNNB1 mt-/wt+', 'PARENTAL007',
                     'P53-/-', 'PTEN-/-', 'PI3KCA mt-/wt+', 'KRAS mt-/wt+',
                     'BAX-/-', 'MEK1-/-', 'PARENTAL001'),
                   mutation = c('AKT1/2', 'MEK2',
                     'AKT1', 'CTNNB1 wt', 'HCT116 P2',
                     'P53', 'PTEN', 'PI3KCA wt', 'KRAS wt',
                     'BAX', 'MEK1', 'HCT116 P1'),
                   startPlate=seq(1, (4*12), by = 4),
                   stringsAsFactors=FALSE)

anno=list(drug=drug, line=line, repl=1:2, ftr=ftrnames)
save(anno, file=file.path("result","data","anno.rda"), compress="bzip2")
```

2.4 Removal/Filtering of empty wells and features

Empty and flagged wells are removed from further analysis.

```
emptyWell = anno$drug$Content == "empty"

screenlog <- read.delim(system.file("extdata",
                                    "conf",
                                    "2013-01-10_LOPACScreenlog_plate.txt",
                                    package = "PGPC"),
                        stringsAsFactors=FALSE)
allPlates = screenlog$Well[screenlog$Plate=="*"]
flaggedWell = anno$drug$Well %in% allPlates

singlePlate = screenlog[screenlog$Plate!="*",]
singlePlate$Plate = paste("P", singlePlate$Plate, sep="")
for (i in 1:nrow(singlePlate)){
  flaggedWell[singlePlate$Plate[i] == anno$drug$Plate &
                singlePlate$Well[i] == anno$drug$Well] = TRUE
}

D <- D[!(emptyWell | flaggedWell),,,]

anno$drug <- anno$drug[!(emptyWell | flaggedWell),]
rownames(anno$drug) <- 1:nrow(anno$drug)
```

Some of the features contain only a small number of unique values (this is especially the case for quantile-based features). Here we remove features with less than 2000 unique values (of 32928 measured values per feature).

```
levels = apply(D, 4, function(x) length(unique(c(x))))

D = D[,,,!(levels < 2000)]
anno$ftr = anno$ftr[-which(levels < 2000)]

datamatrixWithAnnotation = list(D=D, anno=anno)
save(datamatrixWithAnnotation,
     file=file.path("result","data","datamatrixWithAnnotation.rda"),
     compress="xz")
```

2.5 Generalized logarithm transformation

The data is transformed to a logarithmic scale using a so-called generalized logarithm transformation [5]:

$$f(x;c) = \log\left(\frac{x+\sqrt{x^2+c^2}}{2}\right). \tag{1}$$

This avoids singularities for values equal to or smaller than 0. We use the 5% quantile as parameter c. In the cases where the 5% quantile is zero we use 0.05 times the maximum as parameter c.

```
glog <- function(x, c) {
  log2((x+sqrt(x^2+c^2))/2)
}
```

```
D = datamatrixWithAnnotation$D
quantile <- apply(D, 4, quantile, probs=0.05)

zeroQt <- quantile == 0
quantile[zeroQt] <- 0.05*apply(D[,,,zeroQt], 4, max)

for (i in seq_len(dim(D)[4])) {
  D[,,,i] = glog(c(D[,,,i]), c=quantile[i])
}

datamatrixTransformed = list(D=D, anno=datamatrixWithAnnotation$anno)
save(datamatrixTransformed,
     file=file.path("result","data","datamatrixTransformed.rda"),
     compress="xz")
```

```
if(exists("datamatrixWithAnnotation")) rm(datamatrixWithAnnotation)
if(!exists("datamatrixTransformed")){
  data("datamatrixTransformed", package="PGPC")
} else {
  PGPC:::checkConsistency("datamatrixTransformed")
}
```

The data are now represented in the 4-dimensional array `D` with dimensions

	1372	chemical compounds
×	12	cell line
×	2	biological replicate
×	385	phenotypic features

A precomputed version of the `datamatrixTransformed` is available from the package and can be loaded by `data("datamatrixTransformed", package="PGPC")`.

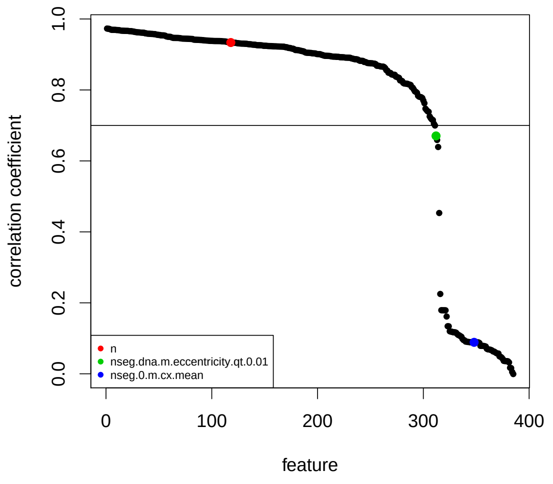

Figure 1: Correlation of features. This figure is the basis for Figure 1B in the paper.

3 Feature selection and calculation of interactions

3.1 Quality control of features

To assess the reproducibility of a feature, we calculate the correlation of each feature from the replicates.

```
getCorrelation <- function(d){
  df <- do.call(rbind,
             lapply(seq_along(d$anno$ftr),
                    function(i) {
                      data.frame(name=d$anno$ftr[i],
                                 cor=cor(c(d$D[,,1,i]),
                                   c(d$D[,,2,i]),
                                   use="pairwise.complete.obs"),
                                 stringsAsFactors=FALSE)
                    }
                    )
                )
  df
}
correlation = getCorrelation(datamatrixTransformed)
correlation = correlation[order(correlation$cor, decreasing=TRUE),]
```

```
selection = c("n", "nseg.dna.m.eccentricity.qt.0.01", "nseg.0.m.cx.mean")

plot(1:nrow(correlation),
     correlation$cor,
     ylab="correlation coefficient",
     xlab="feature",
     pch=20)
abline(h=0.7)
points(which(correlation$name %in% selection),
       correlation$cor[correlation$name %in% selection],
       pch=19,
       col=c(2,3,4))
legend("bottomleft", legend=selection, col=c(2,3,4), pch=19, cex=0.6)
```

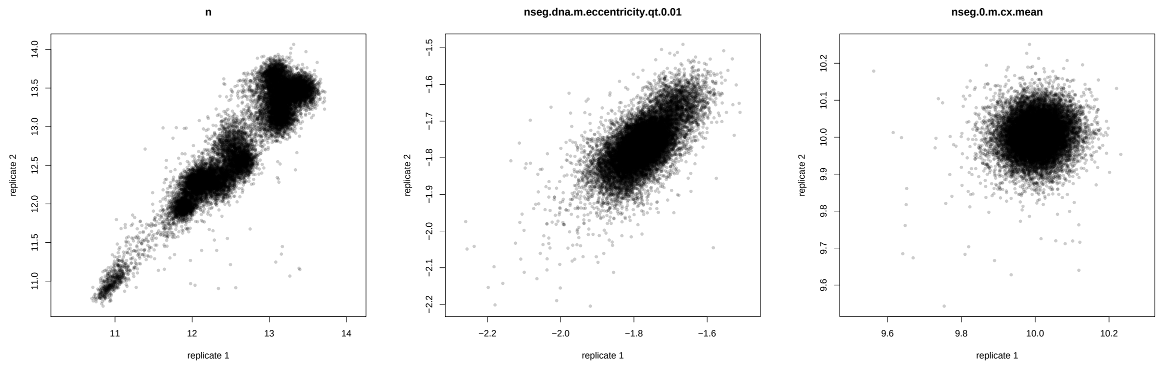

Figure 2: Correlation of feature n, nseg.dna.m.eccentricity.qt.0.01 and nseg.0.m.cx.mean

In Figure 1 feature correlations are plotted in decreasing order. n, nseg.dna.m.eccentricity.qt.0.01, nseg.0.m.cx.mean are highlighted and scatter plots of these features are shown as well in Figure 2.

```
for (i in seq_along(selection)){
  plot(c(datamatrixTransformed$D[,,1,match(selection[i],
                                          datamatrixTransformed$anno$ftr)]),
       c(datamatrixTransformed$D[,,2,match(selection[i],
                                          datamatrixTransformed$anno$ftr)]),
       xlab="replicate 1",
       ylab="replicate 2",
       main = selection[i],
       pch=20,
       col="#00000033",
       asp=1)
}
```

Features with a correlation lower than 0.7 were removed from further analysis.

```
## remove columns with low correlation or few levels
remove = correlation$name[correlation$cor < 0.7]


D = datamatrixTransformed$D[,,, -match(remove, datamatrixTransformed$anno$ftr)]
anno = datamatrixTransformed$anno
anno$ftr = anno$ftr[-match(remove, anno$ftr)]

datamatrixFiltered = list(D=D, anno=anno)
save(datamatrixFiltered,
     file=file.path("result","data","datamatrixFiltered.rda"),
     compress="xz")
rm(datamatrixTransformed)
```

The data are now represented in the 4-dimensional array D with dimensions

$$\begin{array}{rrr} & 1372 & \text{chemical compounds} \\ \times & 12 & \text{cell line} \\ \times & 2 & \text{biological replicate} \\ \times & 310 & \text{phenotypic features} \end{array}$$

3.2 Feature selection by stability

The data contains features that are partially redundant. Therefore we aim to select the features that contain the least redundant information. As first step we center and scale the features, using the median as a measure of location and the median absolute deviation as a measure of spread. The features are selected as described by Laufer et al.

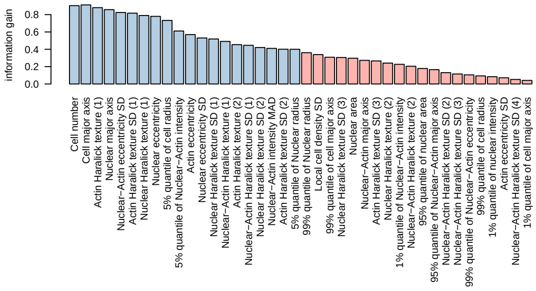

Figure 3: Correlation of selected feature after subtraction of the information containted in the previously selected features.

[4] starting with cell number as first selected feature.

```
D = apply(datamatrixFiltered$D,
          2:4,
          function(x) { (x - median(x,na.rm=TRUE)) / mad(x, na.rm=TRUE) } )
D[!is.finite(D)] = 0
dim(D) = c(prod(dim(D)[1:2]),2,dim(D)[4])

forStabilitySelection = list(D=D,
                             Sample = 1:prod(dim(D)[1:2]),
                             phenotype = datamatrixFiltered$anno$ftr)

preselect=c("n")

selected <- PGPC:::selectByStability(forStabilitySelection,
                                            preselect,
                                            Rdim=40)
save(selected, file=file.path("result","data","selected.rda"), compress="xz")
```

The result `selected` is available from the package and can be loaded by `data("selected", package="PGPC")`.

The result of the feature selection is shown in Figures 3 and 4. The first figure shows the correlations of the fit residuals for the feature selected at each step and the second figure shows the fraction of features for which the fit residuals are positively correlated.

```
if(!exists("selected")){
  data("selected", package="PGPC")
} else {
  PGPC:::checkConsistency("selected")
}

selectedFtrs = selected$selected
correlation = selected$correlation
ratioPositive = selected$ratioPositive

par(mfrow=c(2,1))
barplot(correlation,
        names.arg=PGPC:::hrNames(selectedFtrs),
        las=2,
        col = ifelse(ratioPositive > 0.5,
                     brewer.pal(3,"Pastel1")[2], brewer.pal(3,"Pastel1")[1]),
        ylab = "information gain")
```

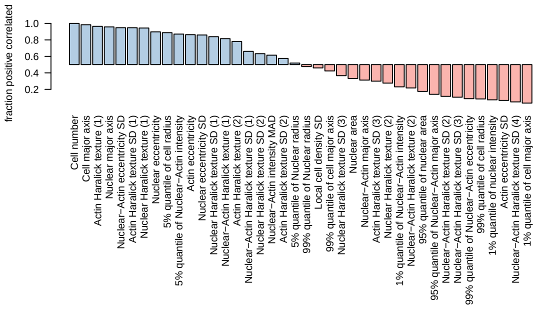

Figure 4: Fraction of positive correlations. This figure is the basis for Figure 1C and Appendix Figure S2A in the paper.

```
par(mfrow=c(2,1))
barplot(ratioPositive-0.5,
        names.arg=PGPC:::hrNames(selectedFtrs),
        las=2,
        col = ifelse(ratioPositive > 0.5,
                     brewer.pal(3,"Pastel1")[2], brewer.pal(3,"Pastel1")[1]),
        offset=0.5,
        ylab = "fraction positive correlated")
#abline(a=0.5,0)

selectedFtrs=selectedFtrs[ratioPositive > 0.5]

D = datamatrixFiltered$D[,,,match(selectedFtrs, datamatrixFiltered$anno$ftr)]
anno = datamatrixFiltered$anno
anno$ftr = anno$ftr[match(selectedFtrs, anno$ftr)]

datamatrixSelected = list(D=D, anno=anno)
save(datamatrixSelected,
     file=file.path("result","data","datamatrixSelected.rda"),
     compress="xz")
```

The feature selection algorithm selected 20 features which are kept for further analysis.

The final data are now represented in the 4-dimensional array D with dimensions

$$\begin{array}{rrr} & 1372 & \text{chemical compounds} \\ \times & 12 & \text{cell line} \\ \times & 2 & \text{biological replicate} \\ \times & 20 & \text{phenotypic features} \end{array}$$

3.3 Calculation of interactions

Te cell number is scaled to the dynamic range between positive (Paclitaxel) and negative controls (DMSO) for each cell line and replicate, to account for the different proliferation rates of the cell lines. For this purpose the median of the control wells is calculated and used for the scaling of cell number.

```
neg = grepl("neg", datamatrixSelected$anno$drug$GeneID)
pos = grepl("Paclitaxel", datamatrixSelected$anno$drug$GeneID)

negMedians = apply(datamatrixSelected$D[neg,,,1], c(2,3), median)
posMedians = apply(datamatrixSelected$D[pos,,,1], c(2,3), median)
```

```
Nnorm = (datamatrixSelected$D[,,,1] - rep(posMedians,
                                          each=dim(datamatrixSelected$D)[1])) /
      (rep(negMedians, each=dim(datamatrixSelected$D)[1]) -
         rep(posMedians, each=dim(datamatrixSelected$D)[1]))

datamatrixSelected$D[,,,1] <- Nnorm


plot(c(datamatrixSelected$D[!(neg | pos),,1,1]),
     c(datamatrixSelected$D[!(neg | pos),,2,1]),
     xlab="replicate 1",
     ylab="replicate 2",
     main = "n",
     pch=20,
     col="grey",
     asp=1)
points(c(datamatrixSelected$D[pos,,1,1]),
     c(datamatrixSelected$D[pos,,2,1]),
     col=2,
     pch=20)
points(c(datamatrixSelected$D[neg,,1,1]),
     c(datamatrixSelected$D[neg,,2,1]),
     col=4,
     pch=20)
```

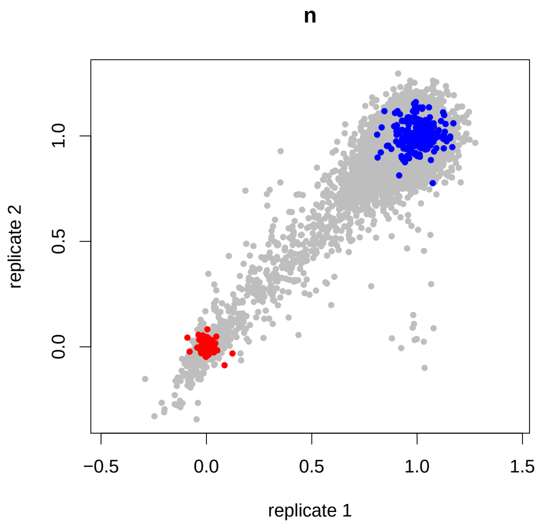

We use an additive model on glog-transformed data to calculate interactions following previous approaches [6, 7, 3, 4]. The fit is implemented using the `medpolish` function, which fits an additive model using Tukey's median polish procedure. The residuals from this fit represent the interactions.

```
interactions = getInteractions(datamatrixSelected,
                               datamatrixSelected$anno$ftr,
                               eps = 1e-4,
                               maxiter = 100)
save(interactions,
     file=file.path("result","data", "interactions.rda"), compress="xz")

## check consistency
PGPC:::checkConsistency("interactions")
```

4 Display of phenotypes as 'phenoprints'

To visualize the phenotypes represented by the extracted features, we display the features as radar plots called 'phenoprints'. For this representation, each feature is scaled by the median absolut deviation observed and then linearly transformed to the interval 0 to 1.

The phenoprints for several drugs are shown for the two parental cell lines and the CTNNB1 wt, cell line. Also the phenoprint for one DMSO control well is shown for all cell lines.

```
if(!exists("interactions")) data(interactions, package="PGPC")
D = interactions$D
D2 = D
dim(D2) = c(prod(dim(D2)[1:2]),dim(D2)[3],dim(D2)[4])

SD = apply(D2, 3, function(m) apply(m, 2, mad, na.rm=TRUE))
MSD = apply(SD, 2, function(x) { median(x,na.rm=TRUE) } )

pAdjusted = interactions$pVal[,,,2]

bin <- function(x) (x-min(x)) / (max(x)-min(x))

## normalize by mean SD
D = apply(D, c(1, 2, 4), mean)
for (i in 1:dim(D)[3]) {
  D[,,i] = bin(D[,,i] / MSD[i])
}

dimnames(D) = list(template = paste(interactions$anno$drug$GeneID),
                   query = interactions$anno$line$mutation,
                   phenotype = PGPC::hrNames(interactions$anno$ftr))
dimnames(pAdjusted) = dimnames(D)


drugPheno <- data.frame(name = c("DMSO", "PD98", "Vinblastin",
                                 "Vincristine", "Ouabain", "Rottlerin",
                                 "Etoposide", "Amsacrine", "Colchicine",
                                 "BIX"),
                        GeneID = c("neg ctr DMSO", "79902", "80101",
                                   "80082", "79817", "79926",
                                   "79294", "79028", "79184",
                                   "80002"),
                        stringsAsFactors=FALSE)
drugPheno$annotationName =
  interactions$anno$drug$Name[match(drugPheno$GeneID,
                                    interactions$anno$drug$GeneID)]

drugPositions <- match(drugPheno$GeneID, dimnames(D)$template)
# define order of ftrs in plot:
#feature clustering
#4 nseg.0.m.majoraxis.mean       --> nuclear shape
#12 nseg.dna.m.eccentricity.sd   --> nuclear shape

#7 nseg.dna.h.var.s2.mean        --> nuclear texture
#13 nseg.dna.h.idm.s1.sd         --> nuclear texture
#17 nseg.dna.h.cor.s2.sd         --> nuclear texture

#1 n                             --> cell number

#6 cseg.act.h.f12.s2.sd          --> cellular texture
```

```
#15 cseg.act.h.asm.s2.mean       --> cellular texture
#18 cseg.dnaact.b.mad.mean       --> cellular texture
#16 cseg.dnaact.h.den.s2.sd      --> cellular texture
#10 cseg.dnaact.b.mean.qt.0.05   --> cellular texture
#3 cseg.act.h.cor.s1.mean        --> cellular texture
#19 cseg.act.h.idm.s2.sd         --> cellular texture
#14 cseg.dnaact.h.f13.s1.mean    --> cellular texture

#9 cseg.0.s.radius.min.qt.0.05   --> cellular shape
#5 cseg.dnaact.m.eccentricity.sd --> cellular shape
#11 cseg.act.m.eccentricity.mean --> cellular shape
#2 cseg.act.m.majoraxis.mean     --> cellular shape

#20 nseg.0.s.radius.max.qt.0.05  --> nuclear shape
#8 nseg.0.m.eccentricity.mean    --> nuclear shape

orderFtr <- c(4,12,7,13,17,1,6,15,18,16,10,3,14,19,9,5,11,2,20,8)
## select cell lines
par001 <- match("HCT116 P1", dimnames(D)$query)
Dselect <- D[drugPositions,par001,]
Dselect <- Dselect[, orderFtr]

stars(Dselect,
      len = 0.8,
      key.loc = c(8, 2),
      labels=drugPheno$name,
      col.stars=rainbow(dim(Dselect)[1]),
      main = "Phenotypes of HCT116 P1",
      draw.segments = FALSE,
      scale=FALSE)

par007 <- match("HCT116 P2", dimnames(D)$query)

Dselect <- D[drugPositions,par007,]
Dselect <- Dselect[, orderFtr]

stars(Dselect,
      len = 0.8,
      key.loc = c(8, 2),
      labels=drugPheno$name,
      col.stars=rainbow(dim(Dselect)[1]),
      main = "Phenotypes of HCT116 P2",
      draw.segments = FALSE,
      scale=FALSE)

mtCtnb1KO <- match("CTNNB1 wt", dimnames(D)$query)
Dselect <- D[drugPositions,mtCtnb1KO,]
Dselect <- Dselect[, orderFtr]

stars(Dselect,
      len = 0.8,
      key.loc = c(8, 2),
      labels=drugPheno$name,
      col.stars= rainbow(dim(Dselect)[1]),
      main = "Phenotypes of CTNNB1 wt",
      draw.segments = FALSE,
      scale=FALSE)
```

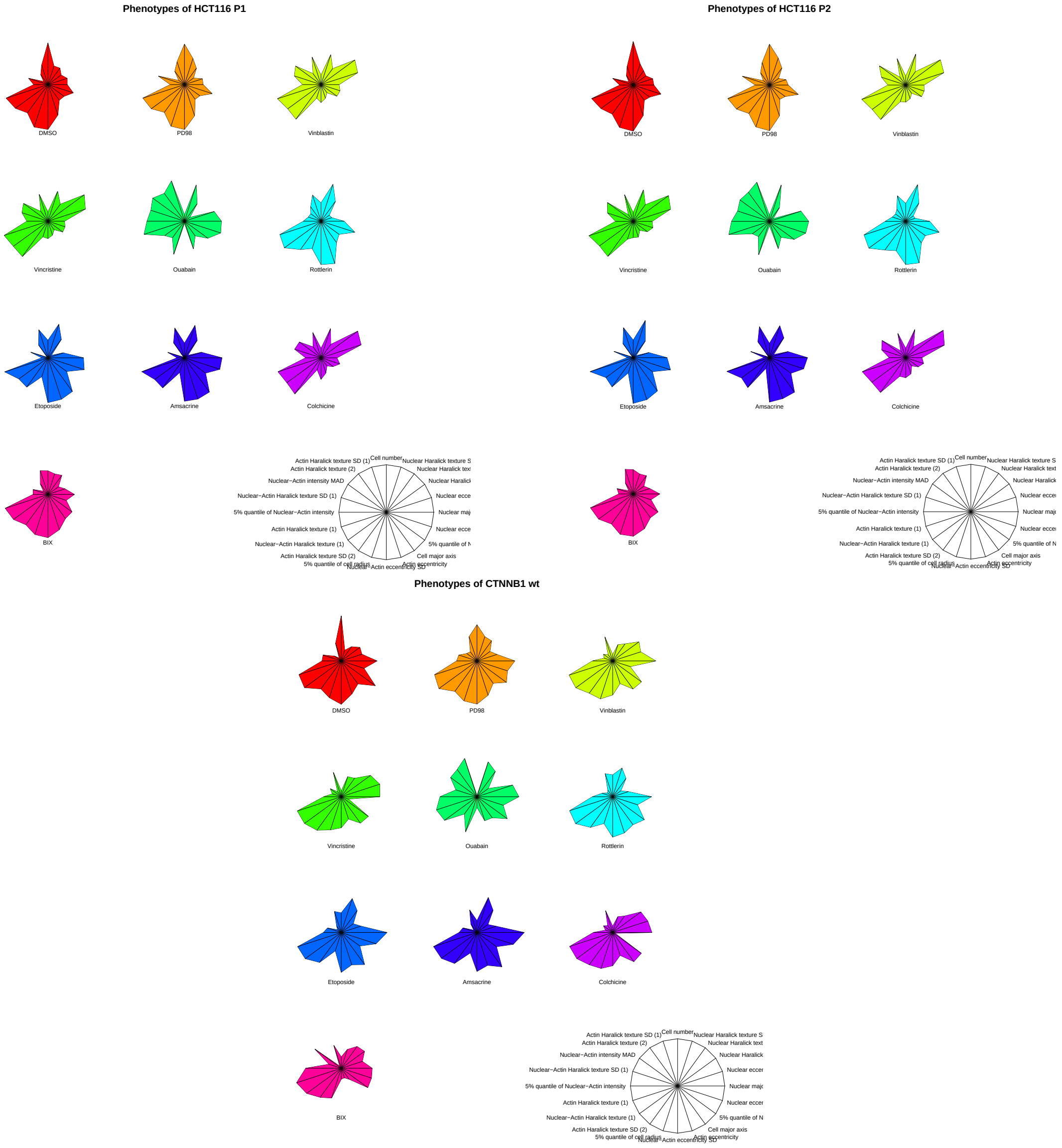

Figure 5: Phenoprints of the two parental cell lines and the CTNNB1 WT background. This figure is the basis for Figure 1E-K, Figure 2A and Appendix Figure S2B in the paper.

```
## neg controls for all cell lines
drugPositions <- match("neg ctr DMSO", dimnames(D)$template)

Dselect <- D[drugPositions,,orderFtr]
stars(Dselect,
      len = 0.8,
      key.loc = NULL,
      labels=dimnames(D)$query,
      col.stars= rainbow(dim(Dselect)[1]),
      main = "Phenotypes of DMSO control for all cell lines",
      draw.segments = FALSE,
      scale=FALSE)
```

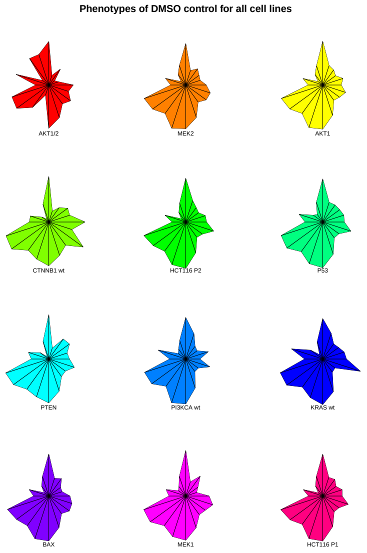

Figure 6: Phenoprints of the all cell lines for a DMSO control. This figure is the basis for Expanded View Figure EV2 in the paper.

5 Display of interactions as star plots.

Similar to the phenotypes, the interaction profiles are displayed as star plots for the different features.

5.1 Scaled to the range of 0 to 1

The interactions are scaled by the median absolute deviation and linearly transformed to the interval 0 to 1 for this display. A ring scale is used to represent the features.

```
#### plot interactions for 1 drug as radar plot of all celllines
#### including ftrs as segments...
if(!exists("interactions")) data(interactions, package="PGPC")
int = interactions$res
dim(int) = c(prod(dim(int)[1:2]),dim(int)[3],dim(int)[4])
dim(int)

## [1] 16464     2    20

SD = apply(int, 3, function(m) apply(m, 2, mad, na.rm=TRUE))
MSD = apply(SD, 2, function(x) { median(x,na.rm=TRUE) } )

pAdjusted = interactions$pVal[,,,2]

bin <- function(x) (x-min(x)) / (max(x)-min(x))

int = apply(interactions$res, c(1, 2, 4), mean)
for (i in 1:dim(int)[3]) {
  int[,,i] = int[,,i] / MSD[i]
  int[,,i] = bin(int[,,i])
}

dimnames(int) = list(template = paste(interactions$anno$drug$GeneID),
                     query = interactions$anno$line$mutation,
                     phenotype = interactions$anno$ftr)
dimnames(pAdjusted) = dimnames(int)
```

```
drugPheno <- data.frame(name = c("Bendamustine", "Disulfiram",
                                  "Colchicine", "BIX01294"),
                        GeneID = c("79497", "80038", "79184", "80002"),
                        stringsAsFactors=FALSE)
drugPheno$annotationName =
  interactions$anno$drug$Name[match(drugPheno$GeneID,
                                    interactions$anno$drug$GeneID)]

##order of featurs for plot
ftrLevels=c("n",
            "nseg.dna.h.cor.s2.sd",
            "nseg.dna.h.idm.s1.sd",
            "nseg.dna.h.var.s2.mean",
            "nseg.dna.m.eccentricity.sd",
            "nseg.0.m.majoraxis.mean",
            "nseg.0.m.eccentricity.mean",
            "nseg.0.s.radius.max.qt.0.05",
            "cseg.act.m.majoraxis.mean" ,
            "cseg.act.m.eccentricity.mean",
            "cseg.dnaact.m.eccentricity.sd",
            "cseg.0.s.radius.min.qt.0.05",
            "cseg.act.h.idm.s2.sd",
            "cseg.dnaact.h.f13.s1.mean",
            "cseg.act.h.cor.s1.mean",
            "cseg.dnaact.b.mean.qt.0.05",
            "cseg.dnaact.h.den.s2.sd",
            "cseg.dnaact.b.mad.mean",
            "cseg.act.h.asm.s2.mean",
            "cseg.act.h.f12.s2.sd" )

## define background colors for phenogroups
backgroundColors = c("black",
                     rep("grey60", 3),
                     rep("grey40", 4),
                     rep("grey20", 4),
                     rep("grey80", 8))

## order of mutations for plot
mutationOrder = c("HCT116 P1", "HCT116 P2", "PI3KCA wt",
                  "AKT1", "AKT1/2", "PTEN", "KRAS wt",
                  "MEK1", "MEK2", "CTNNB1 wt", "P53", "BAX")



### plot radar for each drug showing all cell lines
for(i in seq_len(nrow(drugPheno))){

  drugPosition <- match(drugPheno$GeneID[i], dimnames(int)$template)

  Dselect <- int[drugPosition,,]
  pAdjustedSelect <- pAdjusted[drugPosition,,]

  featureDf <- data.frame(Dselect)
  featureDf$line <- rownames(featureDf)
  featureDf = melt(featureDf, id.vars="line", variable.name="feature")

  pAdjustedDf <- data.frame(pAdjustedSelect)
  pAdjustedDf$line <- rownames(pAdjustedDf)
```

```
pAdjustedDf <- melt(pAdjustedDf,
                    id.vars="line",
                    variable.name="feature",
                    value.name="pAdjusted")


featureDf <- merge(featureDf, pAdjustedDf, sort=FALSE)

pAdjustedThresh = 0.01

## order features and cell lines
featureDf$feature = factor(featureDf$feature,
                           levels=ftrLevels,
                           ordered=TRUE)
featureDf$line <- factor(featureDf$line, levels=mutationOrder)

theme_new = theme_bw()
#theme_new£text£size = 3
#theme_new£axis.text£size = rel(0.2)
theme_new$axis.text.x = element_blank()

maxInt = max(featureDf$value)

colors = colorRampPalette(c("black", "grey90"))(length(unique(featureDf$line)))
if(i==1) colors[grep("AKT1/2", featureDf$line)[1]] = "red"
if(i==2) colors[grep("MEK1", featureDf$line)[1]] = "red"

if(i > 2) {
  colors = c("red", "black")
  featureDf <- subset(featureDf, line %in% c("HCT116 P1", "CTNNB1 wt"))
  featureDf$line = gsub("HCT116 P1", "CTNNB1 mut", featureDf$line)
}

## remove grid
theme_new$panel.grid.major$colour="white"

if(i < 3){
  barColor = "lightblue"
}
if(i==3){
  barColor = "darkslateblue"
}
if(i==4){
  barColor =  "mediumvioletred"
}

starplot <- ggplot(featureDf) +
  facet_wrap(~line, ncol=3) +
  geom_bar(aes(feature, maxInt*1.2),
           fill=backgroundColors,
           stat="identity",
           width = 1)  +
  geom_bar(aes(feature, maxInt),
           fill="white",
           stat="identity",
           width = 1)  +
  geom_bar(aes(feature, value),
           fill=barColor,
```

```
            stat="identity") +
    coord_polar(start=-pi/nlevels(featureDf$feature)) +
    ylim(c(0,maxInt*1.2)) +
    geom_bar(aes(feature, value),
             data = featureDf[featureDf$pAdjusted < pAdjustedThresh,],
             fill="red",
             stat="identity")  +
    geom_point(aes(feature, maxInt*1.1),
               data = featureDf[featureDf$pAdjusted < pAdjustedThresh,],
               pch=8,
               col=2)  +
    theme_new + labs(title = paste0("Interactions of ", drugPheno$name[i]))
  print(starplot)

}
```

Here we plot the scaled profiles for all compounds with significant interactions. This was provided as Appendix Figure 6 and the code is not executed for the generation of this vignette.

```
#### plot interactions for 1 drug as radar plot of all celllines
#### including ftrs as segments...

featureDf <- do.call(rbind,
                     lapply(seq_len(dim(int)[1]),
                            function(i){
                              tmp=data.frame(int[i,,])
                              tmp$GeneID = dimnames(int)[[1]][i]
                              tmp$line <- rownames(tmp)
                              tmp
                            }))
featureDf = melt(featureDf, id.vars=c("GeneID", "line"),
                 variable.name="feature")

pAdjustedDf <- do.call(rbind,
                     lapply(seq_len(dim(pAdjusted)[1]),
                            function(i){
                              tmp=data.frame(pAdjusted[i,,])
                              tmp$GeneID = dimnames(pAdjusted)[[1]][i]
                              tmp$line <- rownames(tmp)
                              tmp
                            }))
pAdjustedDf <- melt(pAdjustedDf,
                    id.vars=c("GeneID", "line"),
                    variable.name="feature",
                    value.name="pAdjusted")

featureDf <- merge(featureDf, pAdjustedDf, sort=FALSE)

## remove controls
featureDf <- subset(featureDf, !grepl("ctr", GeneID))
featureDf$Name <- with(interactions$anno$drug,
                       Name[match(featureDf$GeneID, GeneID)])


pAdjustedThresh = 0.01
## just keep drugs with interactions
featureDf <- subset(featureDf,
                    GeneID %in% unique(GeneID[pAdjusted < pAdjustedThresh]))
```

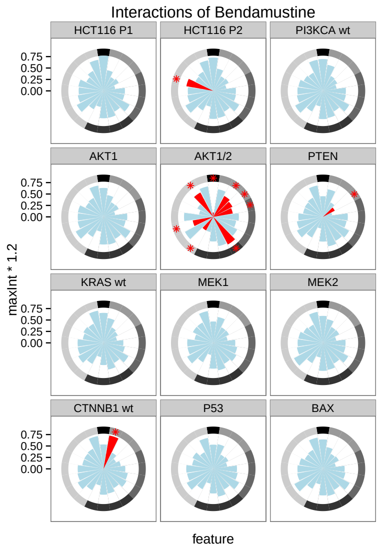

(a) Interaction profile of Bendamustine. This figure is the basis for Figure 4A in the paper.

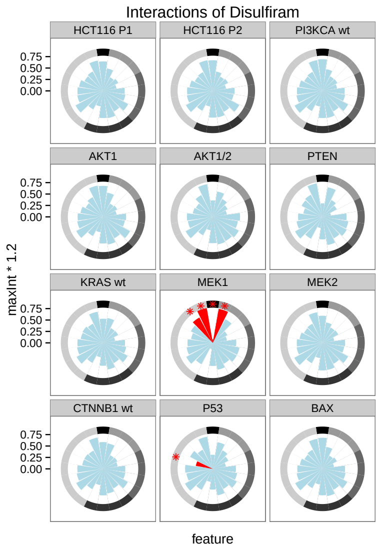

(b) Interaction profile of Disulfiram. This figure is the basis for Figure 4C in the paper.

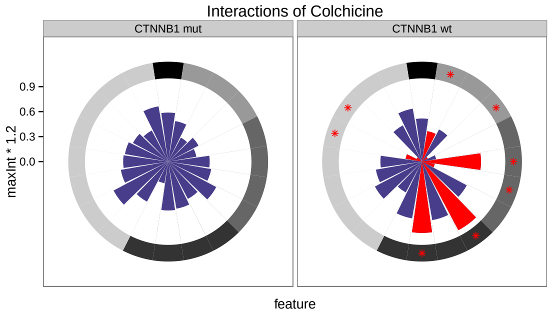

(c) Interaction profile of Colchicine in the parental cell line and the CTNNB1 WT background. This figure is the basis for Figure 2B in the paper.

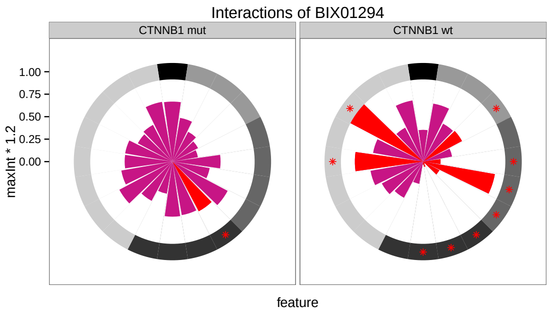

(d) Interaction profile of BIX01294 in the parental cell line and the CTNNB1 WT background. This figure is the basis for Figure 2B in the paper.

Figure 7: Interaction profiles

```
## order features and cell lines
featureDf$feature = factor(featureDf$feature,
                           levels=ftrLevels,
                           ordered=TRUE)
featureDf$line <- factor(featureDf$line, levels=mutationOrder)

maxInt = max(featureDf$value)

colors = colorRampPalette(c("black", "grey90"))(length(unique(featureDf$line)))


theme_new = theme_bw(base_size=5) +
```

```
  theme(panel.grid.major = element_blank(),
        panel.grid.minor = element_blank(),
        axis.text.y = element_text(size=3))
#theme_new£text£size = 3
#theme_new£axis.text£size = rel(0.2)
theme_new$axis.text.x = element_blank()


barColor = "lightblue"

allplots <- lapply(unique(featureDf$GeneID[order(featureDf$Name)]),
                   function(id){
  subset=subset(featureDf, GeneID %in% id)
  starplot <- ggplot(subset) +
    facet_grid(GeneID ~ line) +
    geom_bar(aes(feature, maxInt*1.2),
             fill=backgroundColors,
             stat="identity",
             width = 1)  +
    geom_bar(aes(feature, maxInt),
             fill="white",
             stat="identity",
             width = 1)  +
    geom_bar(aes(feature, value),
             fill=barColor,
             stat="identity") +
    coord_polar(start=-pi/nlevels(featureDf$feature)) +
    ylim(c(0,maxInt*1.2)) +
    geom_bar(aes(feature, value),
             data = subset[subset$pAdjusted < pAdjustedThresh,],
             fill="red",
             stat="identity")  +
    geom_point(aes(feature, maxInt*1.1),
               data = subset[subset$pAdjusted < pAdjustedThresh,],
               pch=8,
               col=2,
               size=0.3) +
    labs(y="Interaction score", x="", title=unique(subset$Name))   +
    theme_new
  invisible(starplot)
})

library(gridExtra)
ggsave(file.path("result", "all_plots.pdf"),
       do.call(marrangeGrob,
               c(allplots, list(nrow=8, ncol=1, top=NULL))),
       width=8.27, height=11.7)
```

5.2 Using the absolute values of an interaction.

The interactions are scaled by the median absolute deviation. The absolut values are plotted after scaling and the direciton of the interaction, defined by the sign of the interaction term, is colorcoded. A ring scale is used to represent the features.

```
#### plot interactions for 1 drug as radar plot of all celllines
#### including ftrs as segments...
```

```
int = apply(interactions$res, c(1, 2, 4), mean)
for (i in 1:dim(int)[3]) {
  int[,,i] = int[,,i] / MSD[i]
}

## use abs value and replace values larger than 10 by 10
direction <- sign(int)

int = abs(int)

dimnames(int) = list(template = paste(interactions$anno$drug$GeneID),
                     query = interactions$anno$line$mutation,
                     phenotype = interactions$anno$ftr)
dimnames(direction) = dimnames(int)


### plot radar for each drug showing all cell lines
for(i in seq_len(nrow(drugPheno))){

  drugPosition <- match(drugPheno$GeneID[i], dimnames(int)$template)

  Dselect <- int[drugPosition,,]
  pAdjustedSelect <- pAdjusted[drugPosition,,]
  directionSelect <- direction[drugPosition,,]

  featureDf <- data.frame(Dselect)
  featureDf$line <- rownames(featureDf)
  featureDf = melt(featureDf, id.vars="line", variable.name="feature")

  pAdjustedDf <- data.frame(pAdjustedSelect)
  pAdjustedDf$line <- rownames(pAdjustedDf)
  pAdjustedDf <- melt(pAdjustedDf,
                      id.vars="line",
                      variable.name="feature",
                      value.name="pAdjusted")

  directionDf <- data.frame(directionSelect)
  directionDf$line <- rownames(directionDf)
  directionDf <- melt(directionDf,
                      id.vars="line",
                      variable.name="feature",
                      value.name="direction")

  directionDf$direction =
      c("negative", "positive")[ifelse(directionDf$direction < 0, 1, 2)]

  featureDf <- merge(featureDf, pAdjustedDf, sort=FALSE)
  featureDf <- merge(featureDf, directionDf, sort=FALSE)

  pAdjustedThresh = 0.01

  ## order features and cell lines
  featureDf$feature = factor(featureDf$feature,
                             levels=ftrLevels,
                             ordered=TRUE)
  featureDf$line <- factor(featureDf$line, levels=mutationOrder)

  theme_new = theme_bw()
```

```
  #theme_new$text$size = 3
  #theme_new$axis.text$size = rel(0.2)
  theme_new$axis.text.x = element_blank()

  maxInt = max(featureDf$value)

  colors = colorRampPalette(c("black", "grey90"))(length(unique(featureDf$line)))
  if(i==1) colors[grep("AKT1/2", featureDf$line)[1]] = "red"
  if(i==2) colors[grep("MEK1", featureDf$line)[1]] = "red"

  if(i > 2) {
    colors = c("red", "black")
    featureDf <- subset(featureDf, line %in% c("HCT116 P1", "CTNNB1 wt"))
    featureDf$line = gsub("HCT116 P1", "CTNNB1 mut", featureDf$line)
  }

  ## remove grid
  theme_new$panel.grid.major$colour="white"

  starplot <- ggplot(featureDf, aes(fill=direction)) +
    facet_wrap(~line, ncol=3) +
    geom_bar(aes(feature, maxInt*1.2),
             fill=backgroundColors,
             stat="identity",
             width = 1)  +
    geom_bar(aes(feature, maxInt),
             fill="white",
             stat="identity",
             width = 1)  +
    geom_bar(aes(feature, value),
             stat="identity") +
    coord_polar(start=-pi/nlevels(featureDf$feature)) +
    ylim(c(0,maxInt*1.2)) +
    geom_point(aes(feature, maxInt*1.1),
               data = featureDf[featureDf$pAdjusted < pAdjustedThresh,],
               pch=8,
               col=2) +
    theme_new + labs(title = paste0("Interactions of ", drugPheno$name[i]))
  print(starplot)

}
```

Here we plot the profiles for all compounds with significant interactions using absolute values and color coding the direction of the interactions. This was provided as Appendix Figure 7 and the code is not executed for the generation of this vignette.

```
#### plot interactions for 1 drug as radar plot of all celllines
#### including ftrs as segments...

featureDf <- do.call(rbind,
                     lapply(seq_len(dim(int)[1]),
                            function(i){
                              tmp=data.frame(int[i,,])
                              tmp$GeneID = dimnames(int)[[1]][i]
                              tmp$line <- rownames(tmp)
                              tmp
                            }))
featureDf = melt(featureDf, id.vars=c("GeneID", "line"),
                 variable.name="feature")
```

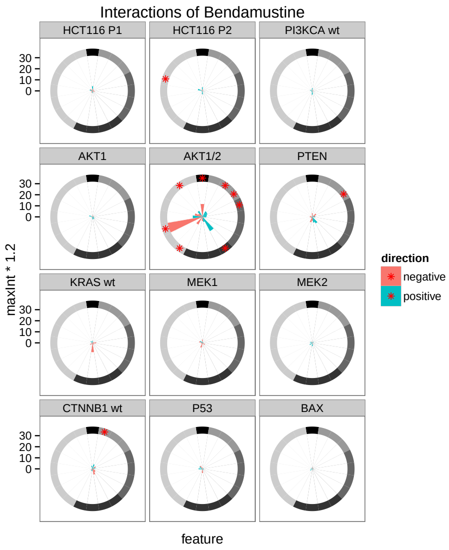

(a) Interaction profile of Bendamustine. This figure is the basis for Appendix Figure S7A in the paper.

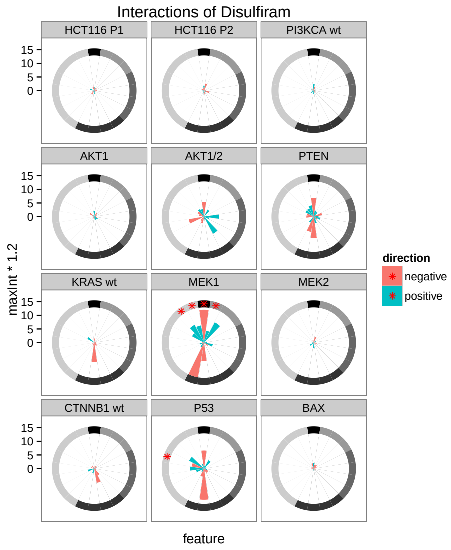

(b) Interaction profile of Disulfiram. This figure is the basis for Appendix Figure S7B in the paper.

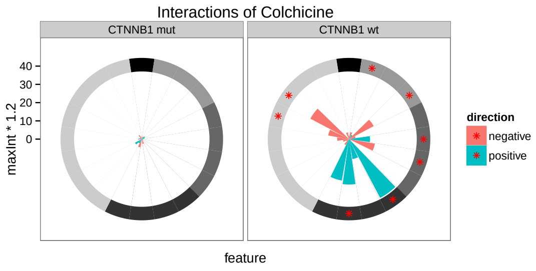

(c) Interaction profile of Colchicine in the parental cell line and the CTNNB1 WT background. This figure is the basis for Appendix Figure S3 in the paper.

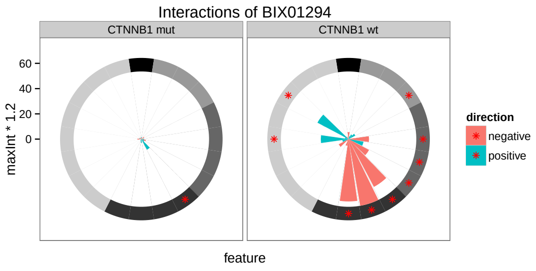

(d) Interaction profile of BIX01294 in the parental cell line and the CTNNB1 WT background. This figure is the basis for Appendix Figure S3 in the paper.

Figure 8: Interaction profiles

```
pAdjustedDf <- do.call(rbind,
                      lapply(seq_len(dim(pAdjusted)[1]),
                             function(i){
                               tmp=data.frame(pAdjusted[i,,])
                               tmp$GeneID = dimnames(pAdjusted)[[1]][i]
                               tmp$line <- rownames(tmp)
                               tmp
                             }))
pAdjustedDf <- melt(pAdjustedDf,
                    id.vars=c("GeneID", "line"),
                    variable.name="feature",
                    value.name="pAdjusted")
```

```
directionDf <- do.call(rbind,
                     lapply(seq_len(dim(pAdjusted)[1]),
                            function(i){
                              tmp=data.frame(direction[i,,])
                              tmp$GeneID = dimnames(direction)[[1]][i]
                              tmp$line <- rownames(tmp)
                              tmp
                            }))
directionDf <- melt(directionDf, id.vars=c("GeneID", "line"), variable.name="feature", value.name="dire
directionDf$direction = c("negative", "positive")[ifelse(directionDf$direction < 0, 1, 2)]

featureDf <- merge(featureDf, pAdjustedDf, sort=FALSE)
featureDf <- merge(featureDf, directionDf, sort=FALSE)

## remove controls
featureDf <- subset(featureDf, !grepl("ctr", GeneID))
featureDf$Name <- with(interactions$anno$drug,
                       Name[match(featureDf$GeneID, GeneID)])


pAdjustedThresh = 0.01
## just keep drugs with interactions
featureDf <- subset(featureDf,
                    GeneID %in% unique(GeneID[pAdjusted < pAdjustedThresh]))

## order features and cell lines
featureDf$feature = factor(featureDf$feature,
                           levels=ftrLevels,
                           ordered=TRUE)
featureDf$line <- factor(featureDf$line, levels=mutationOrder)

maxValue=10

colors = colorRampPalette(c("black", "grey90"))(length(unique(featureDf$line)))


theme_new = theme_bw(base_size=5) +
  theme(panel.grid.major = element_blank(),
        panel.grid.minor = element_blank(),
        legend.key.size = unit(.3, "cm"),
        axis.text.y = element_text(size=3))
#theme_new£text£size = 3
#theme_new£axis.text£size = rel(0.2)
theme_new$axis.text.x = element_blank()


barColor = "lightblue"

allplots <- lapply(unique(featureDf$GeneID[order(featureDf$Name)]),
                   function(id){
  subset=subset(featureDf, GeneID %in% id)
  subset$maxInt = ifelse(max(subset$value) < maxValue,
                         maxValue,
                         max(subset$value))

  starplot <- ggplot(subset, aes(fill=direction)) +
    facet_grid(GeneID ~ line) +
```

```
    geom_bar(aes(feature, maxInt*1.2),
             fill=backgroundColors,
             stat="identity",
             width = 1)  +
    geom_bar(aes(feature, maxInt),
             fill="white",
             stat="identity",
             width = 1)  +
    geom_bar(aes(feature, value),
             stat="identity") +
    coord_polar(start=-pi/nlevels(featureDf$feature)) +
    #ylim(c(0,maxInt*1.2)) +
    scale_y_continuous() +
    geom_point(aes(feature, maxInt*1.1),
               data = subset[subset$pAdjusted < pAdjustedThresh,],
               pch=8,
               col=2,
               size=0.3,
               show_guide=FALSE) +
    labs(y="Interaction score", x="", title=unique(subset$Name)) +
    theme_new
  invisible(starplot)
})

library(gridExtra)
ggsave(file.path("result", "all_plots_unscaled.pdf"),
       do.call(marrangeGrob,
               c(allplots, list(nrow=8, ncol=1, top=NULL))),
       width=8.27, height=11.7)
```

6 Clustering of cell lines

The cell lines are clustered based on the raw feature values and the calculated interactions. We use the mean values of the replicates and scale by the MAD. The correlation between cell line profiles is transformed into a distance for this.

6.1 Clustering cell lines based on features

Clustering of cell lines on their raw features.

```
if(!exists("interactions"))
  data("interactions", package="PGPC")

drugAnno = interactions$anno$drug

filterFDR = function(d, pAdjusted, pAdjustedThresh = 0.1){
  select = pAdjusted <= pAdjustedThresh
  select[is.na(select)] = FALSE
  selectedRows = apply(select, 1, any)
  d[selectedRows,,]
}

D = interactions$D
D2 = D
dim(D2) = c(prod(dim(D2)[1:2]),dim(D2)[3],dim(D2)[4])
```

```
SD = apply(D2, 3, function(m) apply(m, 2, mad, na.rm=TRUE))
MSD = apply(SD, 2, function(x) { median(x,na.rm=TRUE) } )

D = apply(D, c(1, 2, 4), mean)
for (i in 1:dim(D)[3]) {
  D[,,i] = (D[,,i] - median(D[,,i])) / MSD[i]
}

dimnames(D) = list(template = paste(interactions$anno$drug$GeneID),
                   query = interactions$anno$line$mutation,
                   phenotype = interactions$anno$ftr)


## filter FDR
pAdjustedThresh = 0.01
pAdjusted = interactions$pVal[,,,2]
Dfilter = filterFDR(D, pAdjusted, pAdjustedThresh)

## combine controls
Dfilter = apply(Dfilter, c(2,3),
                function(x) tapply(x, dimnames(Dfilter)$template, mean))
ctrlToKeep = c("ctrl Paclitaxel", "ctrl U0126", "ctrl Vinblastin")
Dfilter = Dfilter[!grepl("ctr", dimnames(Dfilter)[[1]]) |
                              dimnames(Dfilter)[[1]] %in% ctrlToKeep,,]

celllineCorrelation = PGPC:::getCorr(aperm(Dfilter, c(2, 1, 3)),
                                     drugAnno)
celllineDist = PGPC:::trsf(celllineCorrelation)

hc <- hclust(as.dist(celllineDist))

heatmap.2(celllineDist,
          Colv = as.dendrogram(hc),
          Rowv = as.dendrogram(hc),
          trace="none",
          col=colorRampPalette(c("darkblue", "white"))(64),
          breaks = c(seq(0,0.5999,length.out=64),0.6),
          margin=c(9,9))

tmp = par(mar=c(5, 4, 4, 10) + 0.1)
plot(as.dendrogram(hc), horiz=TRUE)
par(tmp)

save(celllineDist, file=file.path("result", "celllineDist.rda"))
```

6.2 Clustering cell lines based on interaction terms

Here we do the same analysis as above, just the interaction scores are used this time.

```
PI = interactions$res
PI2 = PI  ##aperm(PI, c(1,3,2,4,5))
dim(PI2) = c(prod(dim(PI2)[1:2]),dim(PI2)[3],dim(PI2)[4])

SD = apply(PI2, 3, function(m) apply(m, 2, mad, na.rm=TRUE))
MSD = apply(SD, 2, function(x) { median(x,na.rm=TRUE) } )

## normalize by mean SD
```

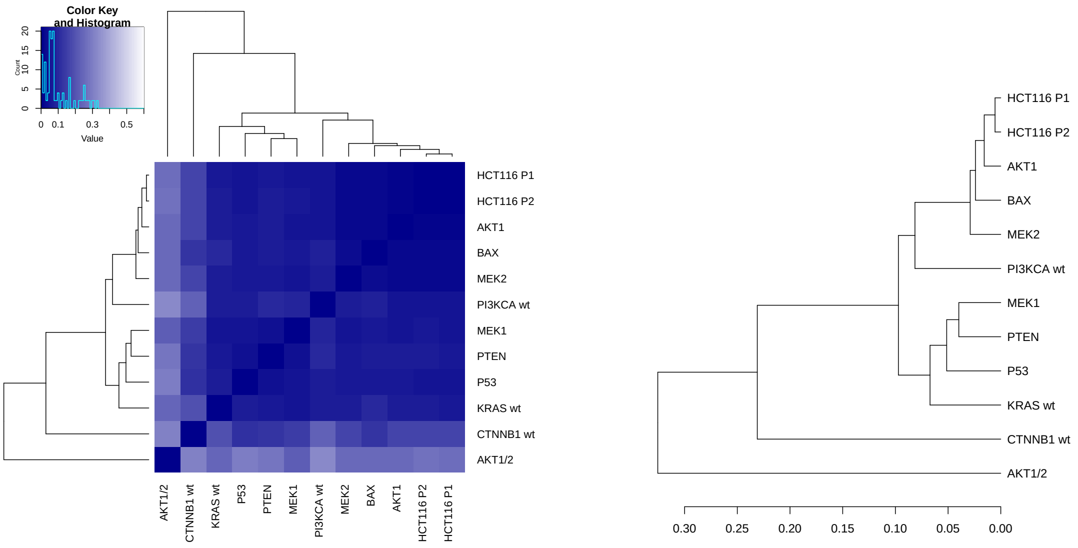

Figure 9: Clustering of cell lines based on the raw values of the selected features. This figure is the basis for Appendix Figure S5B in the paper.

```
PI = apply(interactions$res, c(1, 2, 4), mean)
for (i in 1:dim(PI)[3]) {
  PI[,,i] = PI[,,i] / MSD[i]
}

dimnames(PI) = list(template = interactions$anno$drug$GeneID,
                   query = interactions$anno$line$mutation,
                   phenotype = interactions$anno$ftr)

PIfilter = filterFDR(PI, pAdjusted, pAdjustedThresh)

## combine controls
PIfilter = apply(PIfilter, c(2,3),
                 function(x) tapply(x, dimnames(PIfilter)$template, mean))
PIfilter = PIfilter[!grepl("ctr", dimnames(PIfilter)[[1]]) |
                              dimnames(PIfilter)[[1]] %in% ctrlToKeep,,]


celllineCorrelation = PGPC:::getCorr(aperm(PIfilter, c(2, 1, 3)),
                                     drugAnno)
celllineDist = PGPC:::trsf(celllineCorrelation)

hccelllineDist <- as.dendrogram(hclust(as.dist(celllineDist)))


## reorder HCT116 P1 to top
lines = rownames(celllineDist)
wts = rep(0, length(lines))
wts[match("HCT116 P1", lines)] = 10
hccelllineDist= reorder(hccelllineDist, wts)


heatmap.2(celllineDist,
          Colv = hccelllineDist,
          Rowv = hccelllineDist,
```

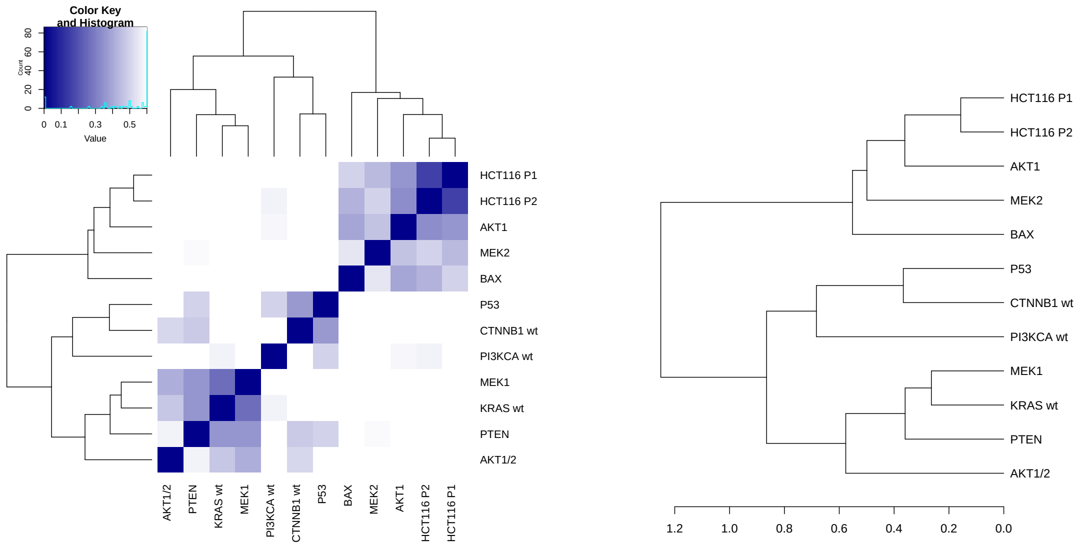

Figure 10: Clustering of cell lines based on the interaction profiles of the selected features. This figure is the basis for Appendix Figure S5C in the paper.

```
          trace="none",
          col=colorRampPalette(c("darkblue", "white"))(64),
          breaks = c(seq(0,0.5999,length.out=64),0.6),
          margin=c(9,9))

tmp = par(mar=c(5, 4, 4, 10) + 0.1)
plot(hccelllineDist, horiz=TRUE)
par(tmp)
```

7 Compound - cell line interaction network

7.1 Extract significant interactions for visualization.

In this section we calculate some statistics from the obtained interaction data and generate a table of drug-cell line interactions for visualizing them graph network in Cytoscype [8].

We start by extracting all interactions with an adjusted p-value ¡ 0.01.

```
library(PGPC)
data(interactions, package="PGPC")
drugAnno = interactions$anno$drug

d = interactions

pAdjusted = interactions$pVal[,,,2]
dimnames(pAdjusted) = list(template = paste(interactions$anno$drug$GeneID),
                          query = interactions$anno$line$mutation,
                          phenotype = interactions$anno$ftr)
pAdjustedThresh = 0.01

result = NULL
for (ftr in seq_along(interactions$anno$ftr)){
  pAdjusted = d$pVal[,,ftr,2]
```

```
  top = pAdjusted <= pAdjustedThresh

  r1 = d$res[,,1,ftr][top]
  r2 = d$res[,,2,ftr][top]

  rMean = apply(d$res[,,,ftr], c(1,2), mean)[top]

  unames <- data.frame(well = rep(d$anno$drug$Well,
                                  length(d$anno$line$startPlate)),
                       plate= rep(as.numeric(gsub("P",
                                                  "",
                                                  d$anno$drug$PlateName)),
                                  length(d$anno$line$startPlate)),
                       startPlate = rep(d$anno$line$startPlate,
                                        each=nrow(d$anno$drug)))
  unames$uname = paste(sprintf("%03d", unames$plate + unames$startPlate - 1),
                       unames$well,
                       sep="-")

  unames = as.matrix(unames$uname)
  dim(unames) = dim(top)

  uname = unames[which(top)]

  ## add annotation
  selTreatment = which(top) %% dim(top)[1]
  selTreatment[selTreatment == 0] = dim(top)[1]
  drug = d$anno$drug[selTreatment,]
  line = d$anno$line[which(top) %/% dim(top)[1]+1,]

  topHits = data.frame(ftr = d$anno$ftr[ftr],
                       uname = gsub("-", "-01-", uname),
                       GeneID = drug$GeneID,
                       r1,
                       r2,
                       rMean,
                       pAdjusted = pAdjusted[which(top)],
                       stringsAsFactors=FALSE)

  topHits = cbind(topHits, line)
  topHits = cbind(topHits,
                  drugAnno[match(topHits$GeneID, drugAnno$GeneID),
                           -match("GeneID",names(drugAnno))])
  topHits = topHits[order(topHits$pAdjusted),]
  rownames(topHits) = 1:nrow(topHits)
  result=rbind(result, topHits)
}

## add controls to names
result$Name[grep("ctr", result$GeneID)] <-
  result$GeneID[grep("ctr", result$GeneID)]

cat("No. of all interactions (including controls):", nrow(result))

## No. of all interactions (including controls): 3067
```

7.1.1 Removing controls from the interactions

We remove the controls from the interactions. The total number of interactions per cell line and total number of drugs showing an interaction for a cell line is calculated and plotted.

```
#########################
## filter interactions
#########################
## 1. remove all controls
result <- subset(result, !grepl("ctr", GeneID))

cat("No. of interactions (controls removed):", nrow(result))

## No. of interactions (controls removed): 2359

cat("No. of drug-cell line interactions (ftrs combined):",
    nrow(unique(result[,c("GeneID", "mutation")])))

## No. of drug-cell line interactions (ftrs combined): 770

## genereate summary plots
## number of interactions per line vs. no of drugs / no of interactions per drugs
mutationOrder = c("HCT116 P1", "HCT116 P2", "PI3KCA wt",
                  "AKT1", "AKT1/2", "PTEN", "KRAS wt",
                  "MEK1", "MEK2", "CTNNB1 wt", "P53", "BAX")

## number of total interactions per cell line
noIntPerLine = table(result$mutation)
noIntPerLine = noIntPerLine[mutationOrder]

tmp = par(mar=c(6, 4, 4, 2) + 0.1)
mp <- barplot(noIntPerLine,
              ylab="total interactions per cell line",
              names.arg="")
text(mp[,1],
     par("usr")[3],
     labels = names(noIntPerLine),
     srt = 45,
     adj = c(1.1,1.1),
     xpd = TRUE,
     cex=.9)
```

```
## number of interaction drugs per line
noDrugsPerLine = sapply(names(noIntPerLine),
                        function(line){
                          length(unique(result$GeneID[result$mutation==line]))
                        })
noDrugsPerLine

## HCT116 P1 HCT116 P2 PI3KCA wt      AKT1    AKT1/2      PTEN   KRAS wt      MEK1      MEK2
##        21        31        80        39       106        73        96        60        35
## CTNNB1 wt       P53       BAX
##       122        64        43

mp <- barplot(noDrugsPerLine,
              ylab="interacting drugs per cell line",
              names.arg="")
text(mp[,1],
     par("usr")[3],
     labels = names(noDrugsPerLine),
     srt = 45,
     adj = c(1.1,1.1),
```

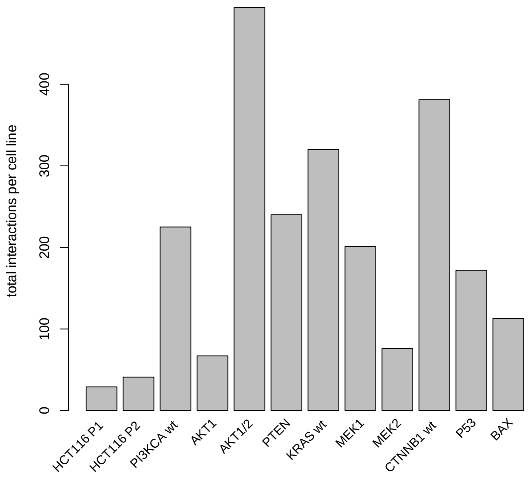

Figure 11: Total number of interactions per cell line. This figure is the basis for Figure 2E in the paper.

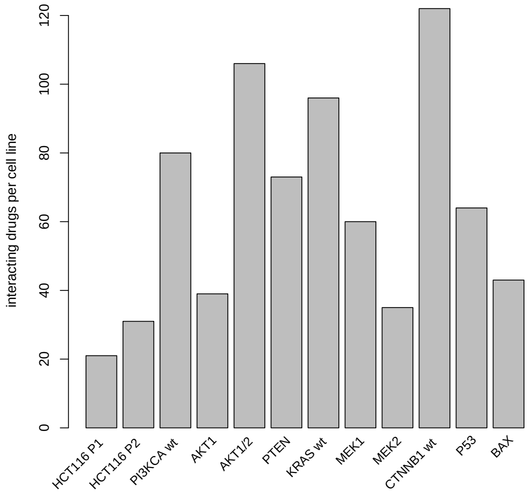

Figure 12: Number of drugs with at least one specific interaction per cell line.

```
     xpd = TRUE,
     cex=.9)
```

```
par(tmp)

## percentage of all possible interactions (removing controls)
nrow(result) /
  ((dim(interactions$res)[1] -
        sum(grepl("ctr", interactions$anno$drug$GeneID))) *
     prod(dim(interactions$res)[c(2,4)]))

## [1] 0.007703109

## percentage of drugs showing an interactions (removing controls)
length(unique(result$GeneID)) /
    (dim(interactions$res)[1] -
         sum(grepl("ctr", interactions$anno$drug$GeneID)))
```

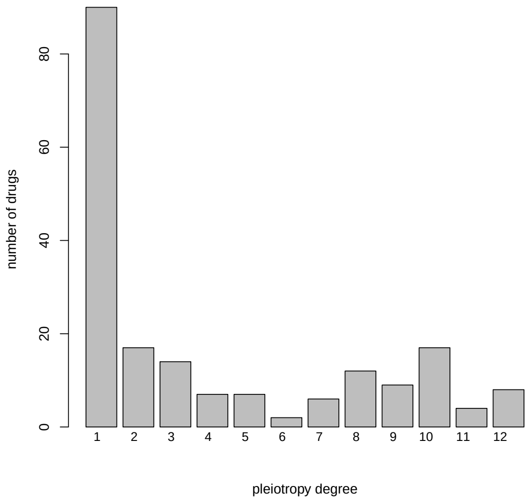

Figure 13: Pleiotropic degree per cell line. This figure is the basis for Figure 2D in the paper.

```
## [1] 0.1512539
```

7.1.2 Pleiotropic degree

Here we define and calculate the pleiotropic degree. It is the number of cell lines that interact with a given drug.

```
## pleiotropy degree
pleiotropicDegree = sapply(unique(result$GeneID),
                          function(drug)
                            length(unique(subset(result, GeneID==drug)$name)))

mp <- barplot(table(pleiotropicDegree),
              ylab="number of drugs",
              names.arg="",
              xlab="pleiotropy degree")
text(mp[,1],
     par("usr")[3],
     labels = 1:12,
     adj = c(1.1,1.1),
     xpd = TRUE,
     cex=.9)
```

```
table(pleiotropicDegree)

## pleiotropicDegree
##  1  2  3  4  5  6  7  8  9 10 11 12
## 90 17 14  7  7  2  6 12  9 17  4  8
```

For some features the pleiotropic degree shows a correlatin with the drug main effect. This is especially the case for cell number.

```
## pleiotropic degree vs. drug effect
drugEffect = apply(interactions$effect$drug, c(1,3), mean)
dimnames(drugEffect) = list(interactions$anno$drug$GeneID,
                            interactions$anno$ftr)

for(ftr in colnames(drugEffect)){
  plot(pleiotropicDegree,
```

```
        drugEffect[match(names(pleiotropicDegree), rownames(drugEffect)), ftr],
        main=ftr,
        ylab ="drug effect")
}
```

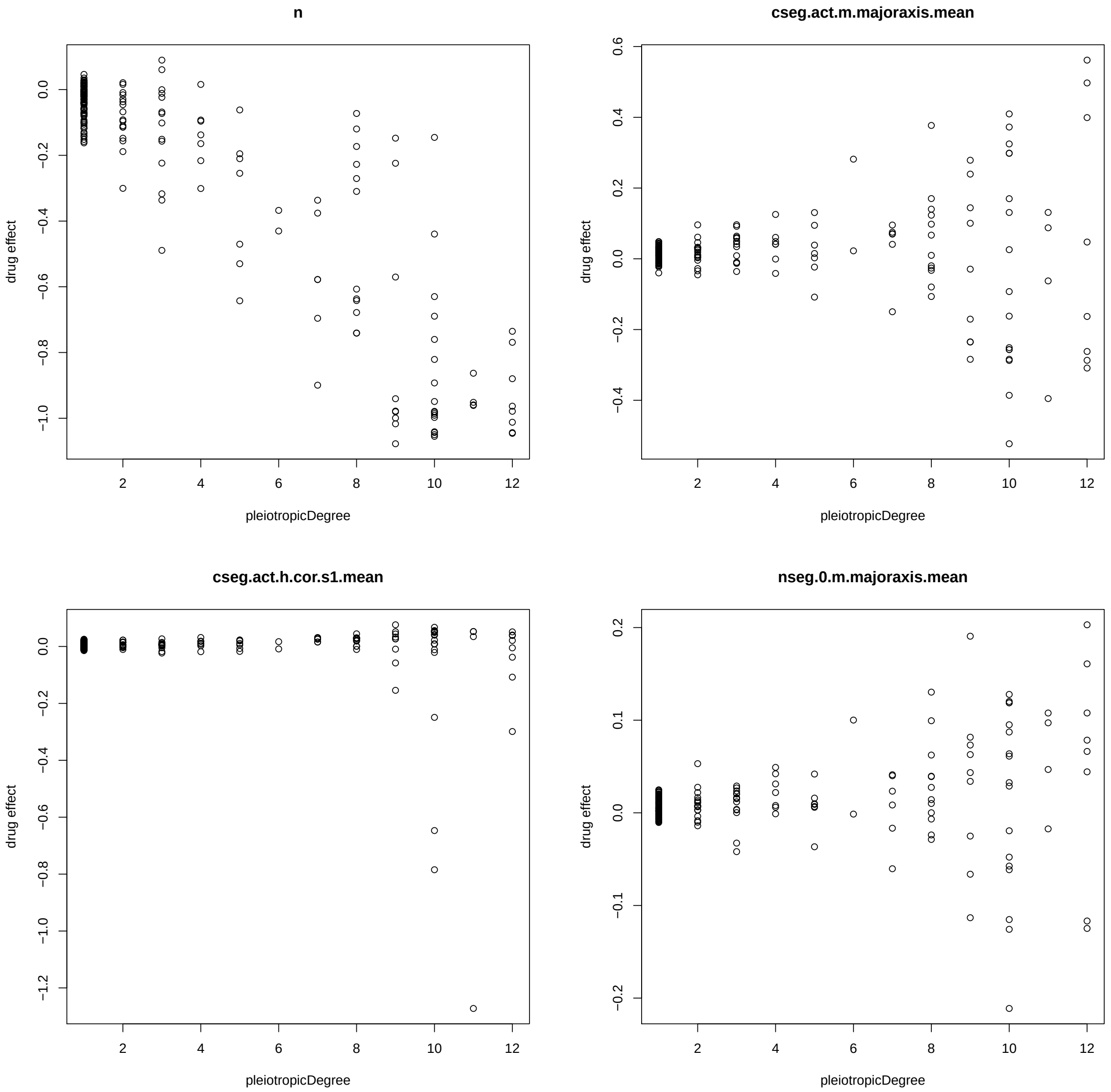

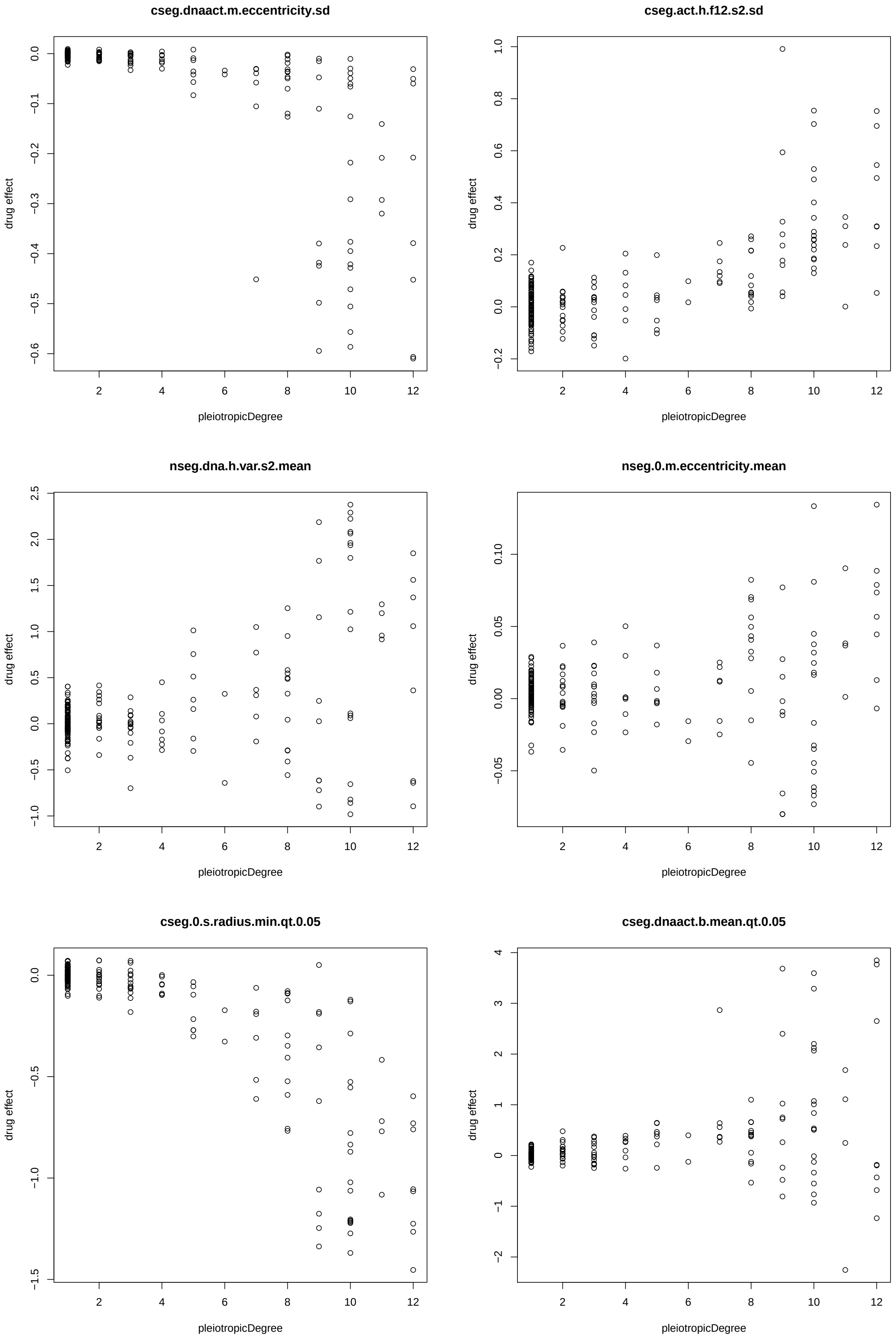
cseg.dnaact.m.eccentricity.sd
cseg.act.h.f12.s2.sd
nseg.dna.h.var.s2.mean
nseg.0.m.eccentricity.mean
cseg.0.s.radius.min.qt.0.05
cseg.dnaact.b.mean.qt.0.05
drug effect
pleiotropicDegree

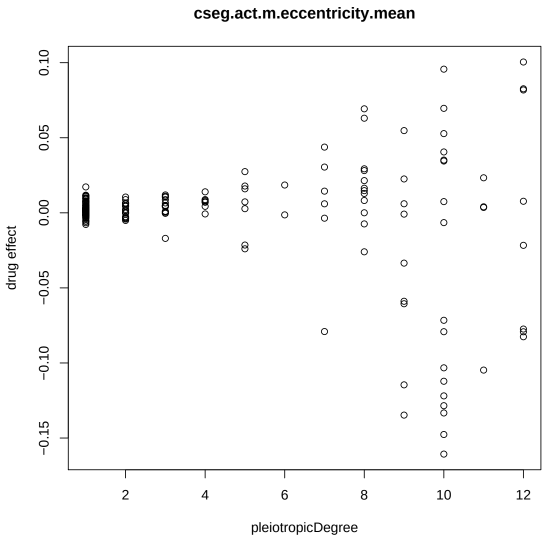
cseg.act.m.eccentricity.mean
drug effect
pleiotropicDegree
0.10
0.05
0.00
−0.05
−0.10
−0.15
2
4
6
8
10
12

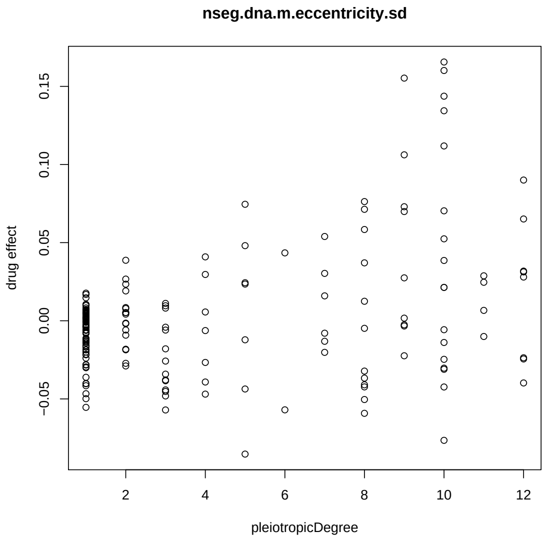
nseg.dna.m.eccentricity.sd
drug effect
pleiotropicDegree
0.15
0.10
0.05
0.00
−0.05
2
4
6
8
10
12

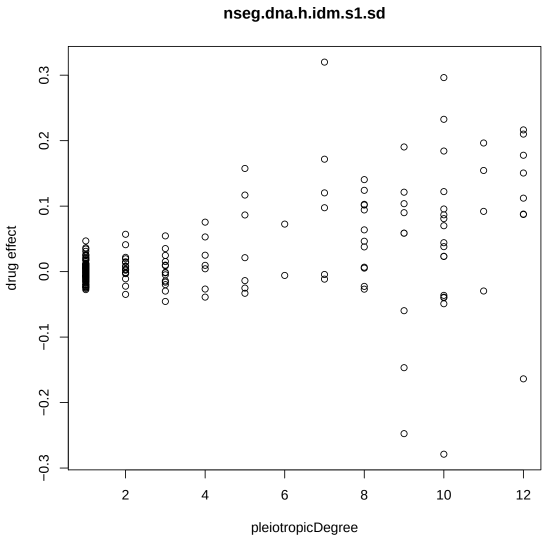
nseg.dna.h.idm.s1.sd
drug effect
pleiotropicDegree
0.3
0.2
0.1
0.0
−0.1
−0.2
−0.3
2
4
6
8
10
12

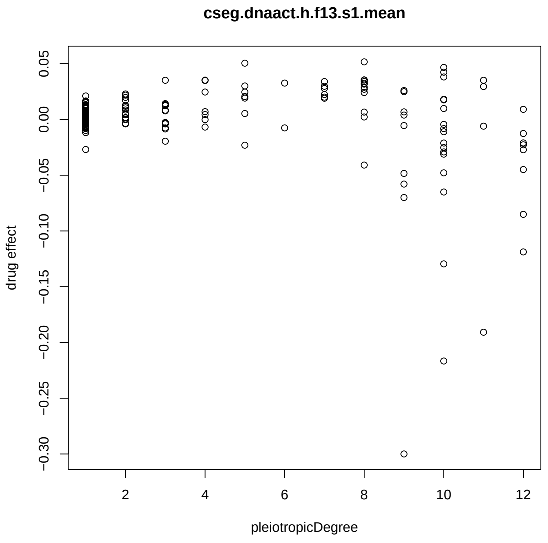
cseg.dnaact.h.f13.s1.mean
drug effect
pleiotropicDegree
0.05
0.00
−0.05
−0.10
−0.15
−0.20
−0.25
−0.30
2
4
6
8
10
12

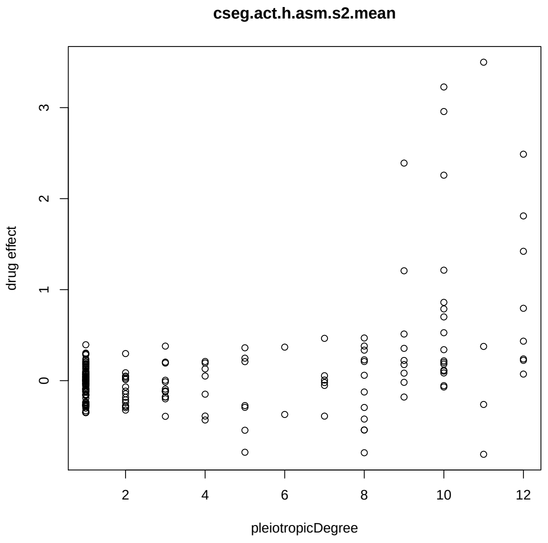
cseg.act.h.asm.s2.mean
drug effect
pleiotropicDegree
3
2
1
0
2
4
6
8
10
12

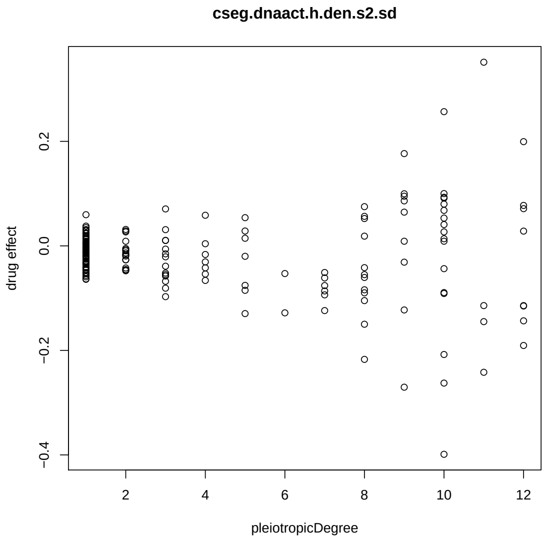
cseg.dnaact.h.den.s2.sd
drug effect
pleiotropicDegree
0.2
0.0
−0.2
−0.4
2
4
6
8
10
12

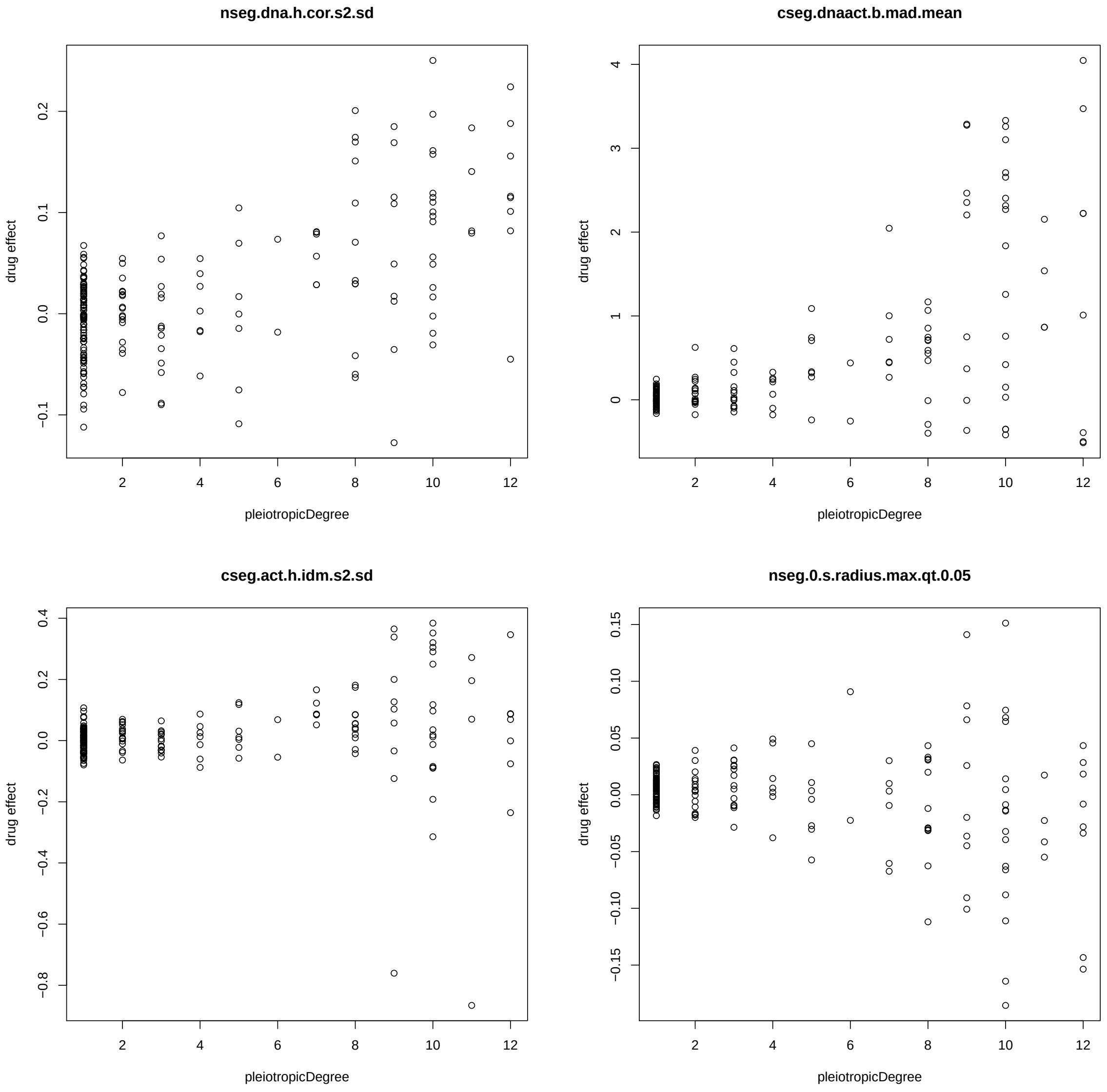

7.1.3 Grouping of features into feature classes.

First the number of interactions for each feature is calculated. Then the features are grouped into feature classes and the number of interactions for each class is calculated. Finally the overlap between feature classes is computed and represented as a venn diagram and barplot.

```
## no of interactions per feature
noIntPerFtr = table(result$ftr)[interactions$anno$ftr]

tmp = par(mar=c(9, 4, 4, 2) + 0.1)
mp <- barplot(noIntPerFtr,
              ylab="total interactions per feature",
              names.arg="")
text(mp[,1],
     par("usr")[3],
     labels = names(noIntPerFtr),
     srt = 45,
     adj = c(1.1,1.1),
     xpd = TRUE,
     cex=.9)
```

```
par(tmp)


result$ftrClass = PGPC:::hrClass(result$ftr)
significant = lapply(unique(result$ftrClass),
                     function(selectedFtr)
                       unique(subset(result, ftrClass==selectedFtr)$uname))
names(significant) = unique(result$ftrClass)

## merging the interactions for each category
plot(venn(significant))


overlap = sapply(names(significant),
                 function(class1){
                   sapply(names(significant), function(class2){
                     length(intersect(significant[[class1]],
                                      significant[[class2]]))
                   })
                 })
barplot(overlap,
        beside=TRUE,
        legend=names(significant),
        args.legend=list(x="topleft"))
```

7.1.4 Interaction map export for Cytoscape

To obtain a interaction map for display in Cytoscape we remove all drugs that only show an interaction for one feature in a given cell line. Also ambigous drugs showing interactions with 3 or more cell lines are removed.

```
## remove low confidence interactions
result <- do.call(rbind,
                  lapply(unique(result$name),
                         function(line) {
                           tmp = subset(result, line == name)
                           noFtrPerDrug = table(tmp$GeneID)
                           selectedDrug = names(noFtrPerDrug[noFtrPerDrug > 1])
                           subset(tmp, GeneID %in% selectedDrug)
                         }))

## remove ambiguous interactions
noLinesPerDrug = sapply(unique(result$GeneID),
                        function(drug)
                          length(unique(subset(result, GeneID==drug)$name)))
unambigDrugs = names(noLinesPerDrug[noLinesPerDrug <= 3])
result = subset(result, GeneID %in% unambigDrugs)
```

Some additional statistics that are not presented in the paper are calculated in this section.

```
## number of interactions per line vs. no of drugs / no of interactions per drugs
noIntPerLine = table(result$name)
barplot(noIntPerLine)

noDrugsPerLine = sapply(names(noIntPerLine),
                        function(line)
                            length(unique(result$GeneID[result$name==line])))

plot(as.vector(noIntPerLine), noDrugsPerLine)
```

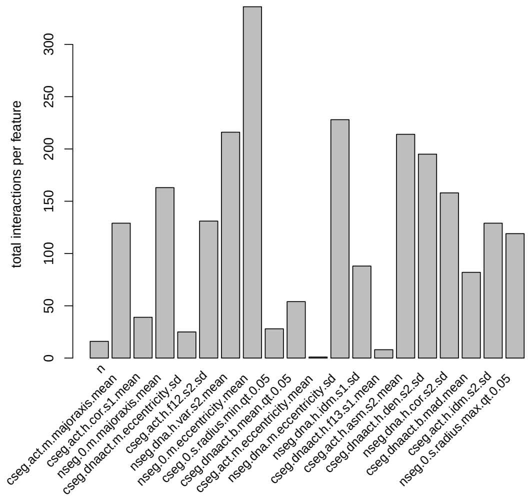

(a) Total interactions per feature.

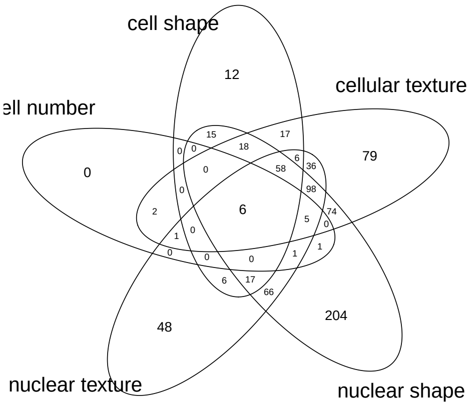

(b) Overlap of interactions in the 5 phenotypic classes. This figure is the basis for Figure 2C in the paper.

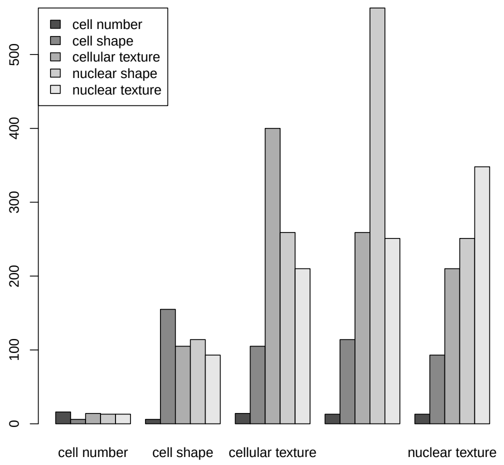

(c) Overlap of interactions in the 5 phenotypic classes shown as bar plots.

Figure 14: Distributions and overlap of interactions per feature

```
text(as.vector(noIntPerLine), noDrugsPerLine, names(noIntPerLine))

noIntPerDrug = table(result$GeneID)
barplot(noIntPerDrug)

for(line in unique(result$name)){
  barplot(table(result$GeneID[result$name==line]), main=line)
}

## features showing interactions per drug
noFtrsPerDrug = sapply(names(noIntPerDrug),
                       function(drug)
                         length(unique(result$ftr[result$GeneID==drug])))
```

```
plot(as.vector(noIntPerDrug), noFtrsPerDrug)
text(as.vector(noIntPerDrug), noFtrsPerDrug, names(noIntPerDrug))

hist(noFtrsPerDrug, breaks=20)

for(i in 1:5) print(sum(noFtrsPerDrug>i))
```

The results are saved and used as input to Cytoscape for visualization. The interactions for different features are combined into Phenogroups and also merged completely. In the latter case number of feature classes is calculated. We used the results with phenogroups as input to Cytoscape.

```
write.table(result, file=file.path("result", "cytoscapeExportFiltered.txt"),
            sep="\t",
            row.names=FALSE)

############################
## transform to pheno groups
############################
result$ftr = PGPC:::hrClass(result$ftr)
columnsToRemove = c("r1", "r2", "rMean", "pAdjusted")
result = unique(result[,-match(columnsToRemove, names(result))])

write.table(result,
            file=file.path("result", "cytoscapeExportFilteredPhenoGroups.txt"),
            sep="\t",
            row.names=FALSE)

#########################
## combine features
#########################
result = do.call(rbind,
                 lapply(unique(result$uname),
                        function(u){
                          tmp = subset(result, uname==u)
                          ftr = tmp$ftr
                          tmp = unique(tmp[,-match(c("ftr", "ftrClass"),
                                                   names(tmp))])
                          tmp$cellnumber =
                              ifelse("cell number" %in% ftr, 1, 0)
                          tmp$cellshape =
                              ifelse("cell shape" %in% ftr, 1, 0)
                          tmp$celltexture =
                              ifelse("cellular texture" %in% ftr, 1, 0)
                          tmp$nuclearshape =
                              ifelse("nuclear shape" %in% ftr, 1, 0)
                          tmp$nucleartexture =
                              ifelse("nuclear texture" %in% ftr, 1, 0)
                          tmp$noFtrClasses = length(ftr)
                          tmp
                        }))

write.table(result, file=file.path("result",
                                   "cytoscapeExportFilteredFtrsCombined.txt"),
            sep="\t",
            row.names=FALSE)
```

8 Heat maps of interaction profiles

In this section we display the interaction profiles as heatmaps. In order to visualize the interactions of all features in one plot the interaction terms are scaled by the median and median absolute deviation for each feature.

```
if(!exists("interactions")){
  data("interactions", package="PGPC")
}
PI = interactions$res
PI2 = PI  ##aperm(PI, c(1,3,2,4,5))
dim(PI2) = c(prod(dim(PI2)[1:2]),dim(PI2)[3],dim(PI2)[4])

SD = apply(PI2, 3, function(m) apply(m, 2, mad, na.rm=TRUE))
MSD = apply(SD, 2, function(x) { median(x,na.rm=TRUE) } )

## normalize by mean SD
PI = apply(interactions$res, c(1, 2, 4), mean)
for (i in 1:dim(PI)[3]) {
  PI[,,i] = PI[,,i] / MSD[i]
}

dimnames(PI) = list(template = interactions$anno$drug$GeneID,
                   query = interactions$anno$line$mutation,
                   phenotype = interactions$anno$ftr)

myColors = c(`Blue`="cornflowerblue",
  `Black`="#000000",
  `Yellow`="yellow")
colBY = colorRampPalette(myColors)(513)

cuts = c(-Inf,
         seq(-6, -2, length.out=(length(colBY)-3)/2),
         0.0,
         seq(2, 6, length.out=(length(colBY)-3)/2),
         +Inf)

ppiw = .25
ppih = 1.4
fac = 2.2
d = dim(PI)

ordTempl = PGPC:::orderDim(PI, 1)
ordQuery = PGPC:::orderDim(PI, 2)
ordFeat  = PGPC:::orderDim(PI, 3)

PGPC:::myHeatmap(PI[ordTempl, ordQuery, ordFeat],
                 cuts=cuts,
                 fontsize=10,
                 col=colBY)
```

Next we focus on the interactions with a FDR below 0.01. Also all controls are removed, except Paclitaxel, U0126 and Vinblastin. The mean values accross the control wells are calculated for the selected controls.

```
filterFDR = function(d, pAdjusted, pAdjustedThresh = 0.1){
  select = pAdjusted <= pAdjustedThresh
  select[is.na(select)] = FALSE
  selectedRows = apply(select, 1, any)
  d[selectedRows,,]
}
```

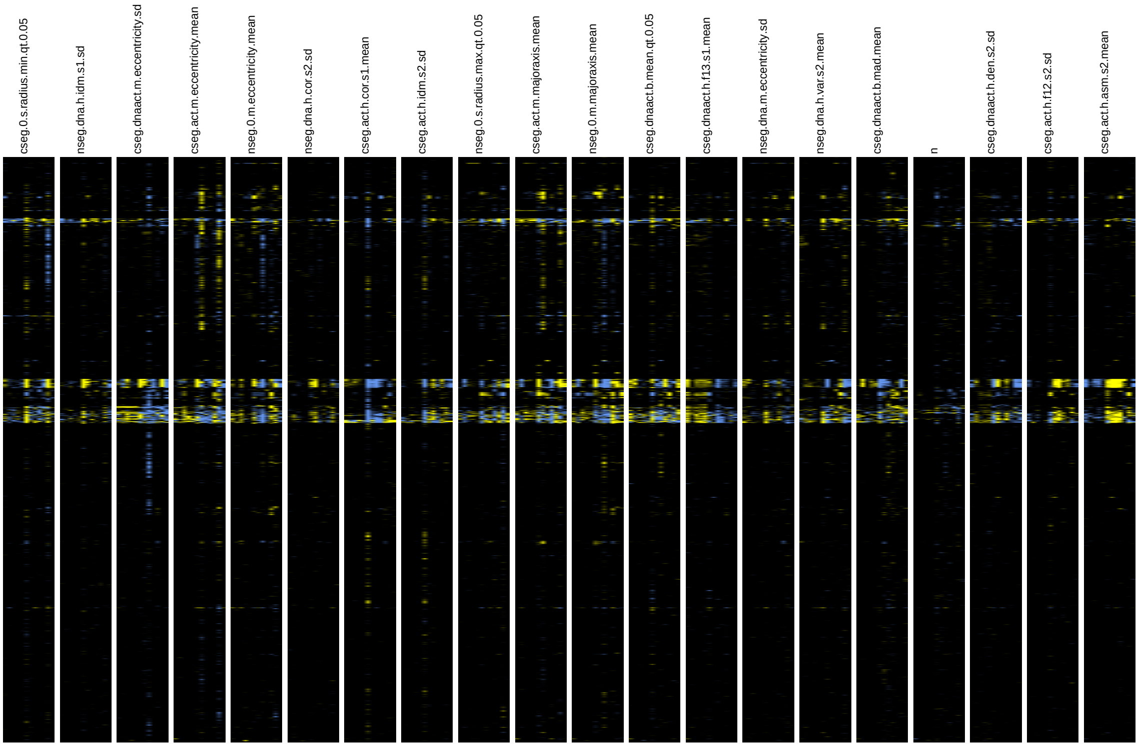

Figure 15: Heatmap of interaction profiles for all drugs

```
pAdjustedThreshold = 0.01
pAdjusted = interactions$pVal[,,,2]
PIfilter = filterFDR(PI,
                     pAdjusted,
                     pAdjustedThresh = pAdjustedThreshold)

PIfilter = apply(PIfilter, c(2,3),
                 function(x) tapply(x, dimnames(PIfilter)$template, mean))

ctrlToKeep = c("ctrl Paclitaxel", "ctrl U0126", "ctrl Vinblastin")
### some other contrls:
# ctrlToKeep = c("ctrl Paclitaxel", "ctrl U0126", "ctrl Vinblastin", "ctrl IWP", "ctrl DAPT")
PIfilter = PIfilter[!grepl("ctr", dimnames(PIfilter)[[1]]) |
                              dimnames(PIfilter)[[1]] %in% ctrlToKeep,,]

ordTempl  = PGPC:::orderDim(PIfilter, 1)
ordQuery  = PGPC:::orderDim(PIfilter, 2)
ordFeat   = PGPC:::orderDim(PIfilter, 3)

PGPC:::myHeatmap(PIfilter[ordTempl,ordQuery,ordFeat],
                 cuts=cuts,
                 fontsize=10,
                 col=colBY)
```

```
drugAnno = interactions$anno$drug
subset = drugAnno[drugAnno$compoundID %in% dimnames(PIfilter)[[1]] &
                    !grepl("ctr", drugAnno$GeneID),]
write.table(subset[, c("Name", "GeneID", "Selectivity", "Selectivity_updated")],
            file=file.path("result", "annotation_selected_compounds.txt"),
            sep="\t",
            quote=FALSE,
```

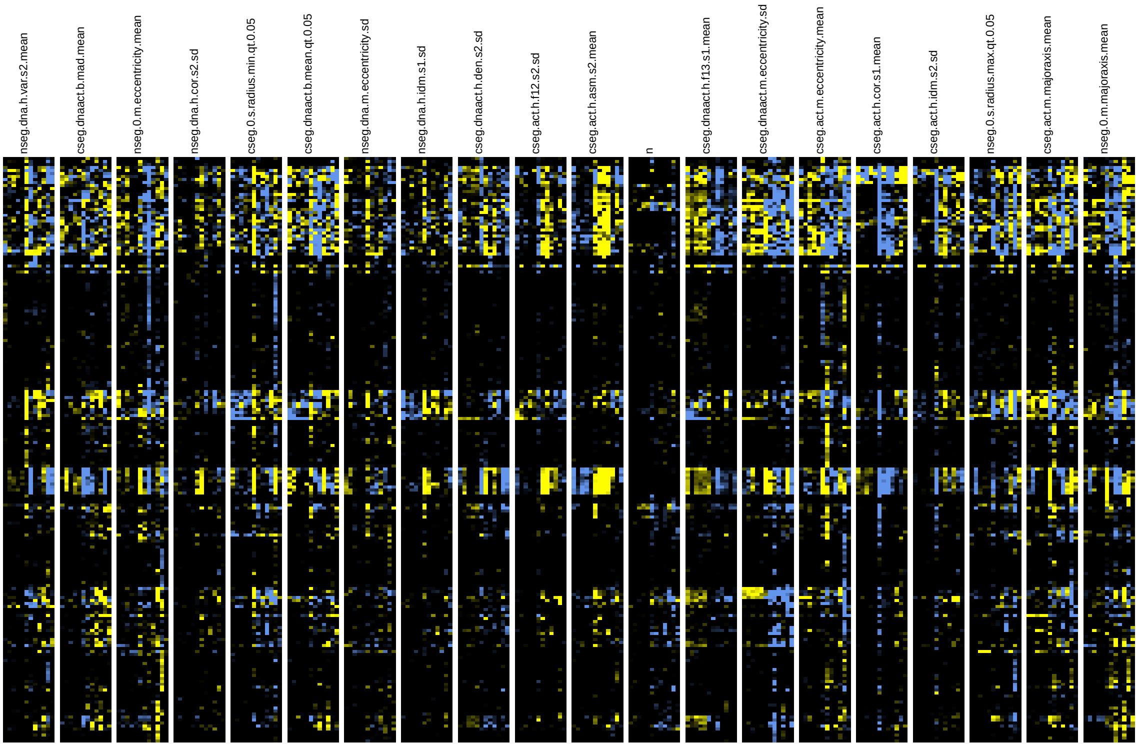

Figure 16: Heatmap of interaction profiles for drugs that show at least one specific interaction

```
            row.names=FALSE)
```

9 Clustering of interaction profiles

9.1 Clustering of interaction profiles using the filtered data

To investigate drug similarity and cluster drugs with similar function or targets, we use the interactions profiles to calculate a distance between the drugs. The metric that we use is 1-cor(x,y), where x and y represent the interaction profiles for two drugs.

```
PIdist = PGPC:::getDist(PIfilter, drugAnno=drugAnno)

hcInt <- as.dendrogram(hclust(as.dist(PIdist)))

heatmap.2(PIdist,
          Colv = hcInt,
          Rowv = hcInt,
          trace="none",
          col=colorRampPalette(c("darkblue", "white"))(64),
          breaks = c(seq(0,0.5999,length.out=64),0.6),
          cexRow=.15,
          cexCol=.15)
```

9.1.1 Reordered dendrogram

Due to the ambiguity of the cluster tree and for visualization purposes we rearange two clusters. Clusters are colored by cutting the cluster tree at a height of 0.6. For visability we only color clusters that contain at least 3 drugs.

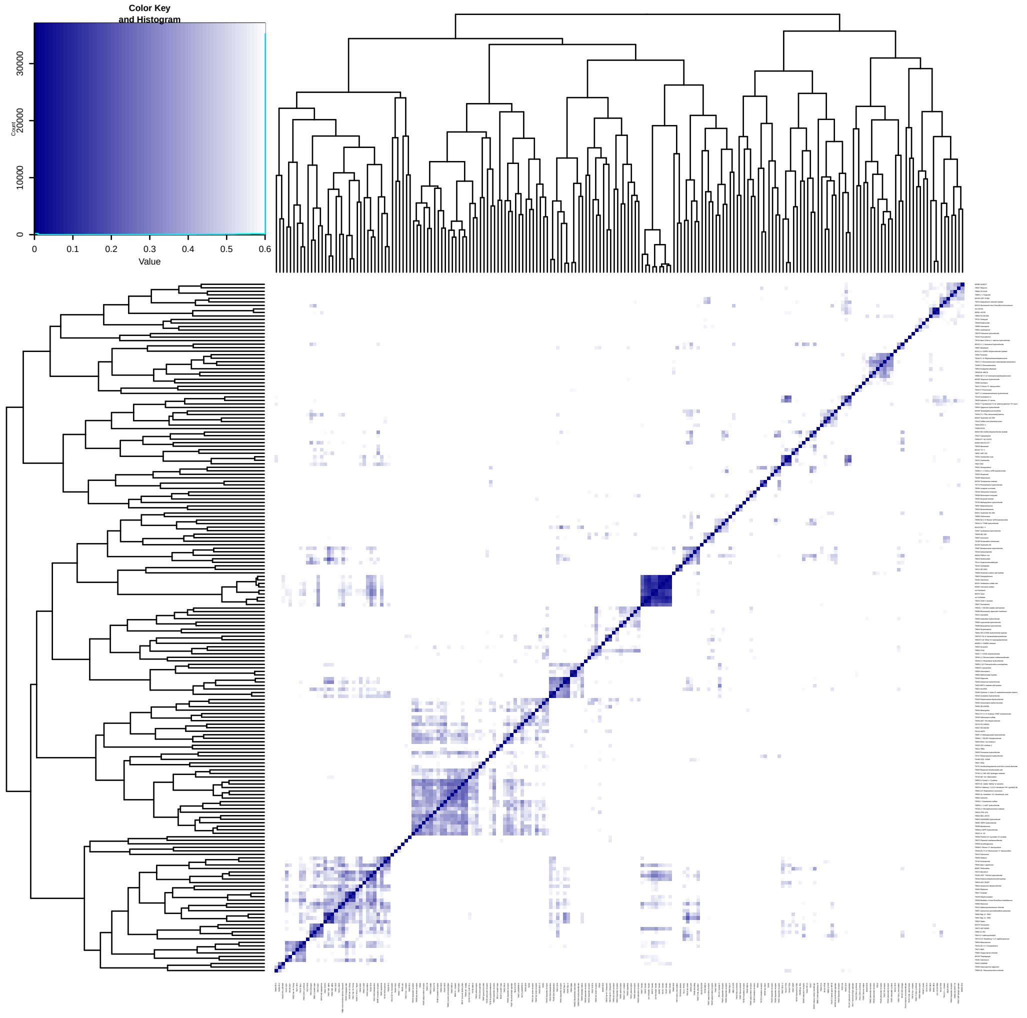

Figure 17: Clustering of interaction profiles for drugs that show at least one interaction.

```
## reorder dendrogram
wts = rep(0, dim(PIdist)[1])

## reorder bio cluster
inbetween = c(146, 187,  66, 170,  73, 121, 180)
wts[inbetween] = 1000

drugIds = sapply(strsplit(rownames(PIdist), " "), "[", 1)

## reorder Etoposide cluster
wts[match("79462", drugIds)] = 10

## reorder calcimycin cluster
wts[match("79471", drugIds)] = 5
wts[match("79982", drugIds)] = 10

hcInt = reorder(hcInt, wts)

cluster = cutree(as.hclust(hcInt), h=0.6)

## make color table
inCl <- table(cluster)
```

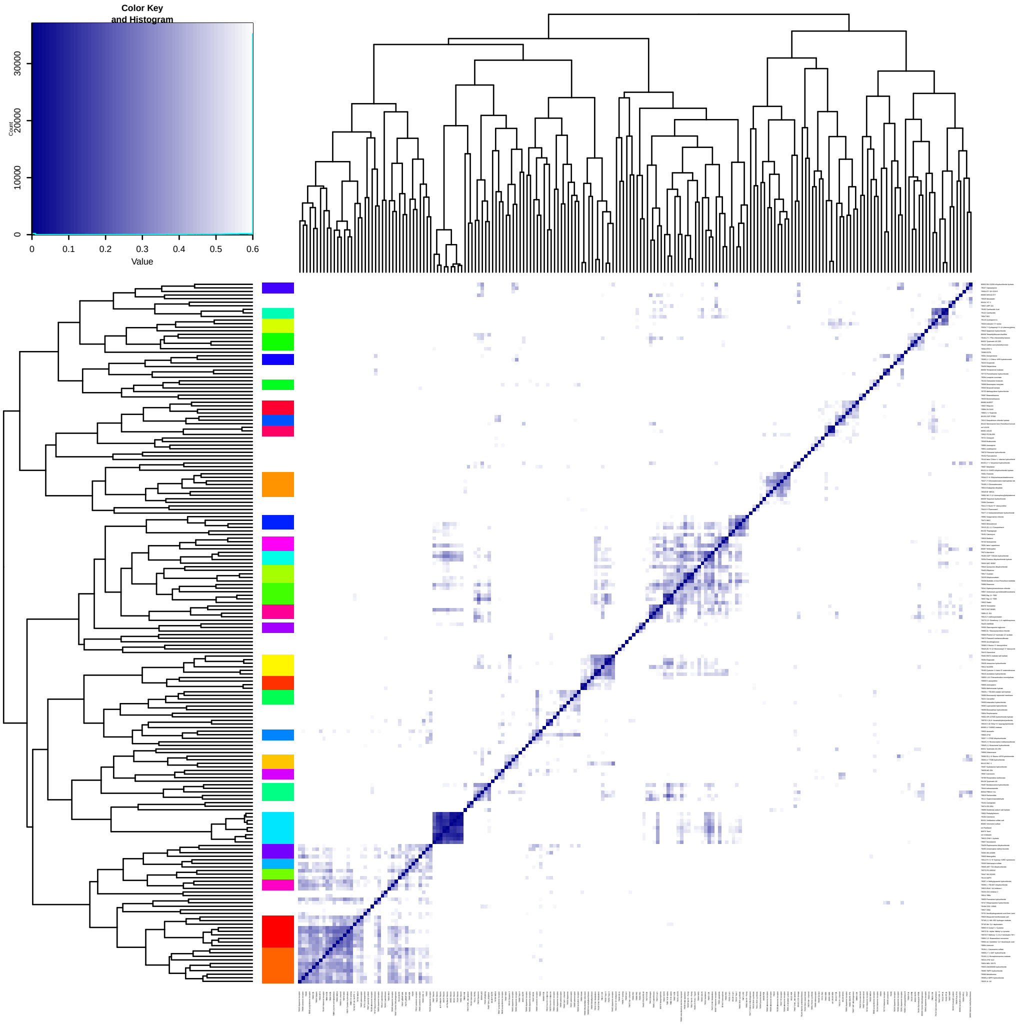

Figure 18: Clustering of interaction profiles for drugs that show at least one interaction. Clusters of size larger than 2 are colored. The dendrogram and coloring of clusters in this figure are the basis for Figure 5A and Appendix Figure S8 in the paper.

```
cl2col <- data.frame(cl=names(inCl), col="white", stringsAsFactors=FALSE)
largerCluster <- names(inCl[inCl>2])
cl2col$col[cl2col$cl %in% largerCluster] <- rainbow(length(largerCluster))

col = cl2col$col[match(cluster, cl2col$cl)]

heatmap.2(PIdist,
          Colv = hcInt,
          Rowv = hcInt,
          trace="none",
          col=colorRampPalette(c("darkblue", "white"))(64),
          breaks = c(seq(0,0.5999,length.out=64),0.6),
          cexRow=.15,
          cexCol=.15,
          RowSideColors=col)
```

9.2 Structural similarity of compounds

Here we investigate the chemical similarity of compounds based on their 2D structure using the *ChemmineR* package. As distance measure the Tanimoto Coefficient is used.

```
## read structure file
sdfset <- read.SDFset(system.file("extdata",
                                  "conf",
                                  "LOPAC-regexpr-formated.sdf",
                                  package = "PGPC"))

## map GeneIDs and library names to the compound names in the sdf file
drugs <-  data.frame(GeneID = dimnames(PIfilter)[[1]], stringsAsFactors=FALSE)
drugs$Name = interactions$anno$drug$Name[match(drugs$GeneID,
                                               interactions$anno$drug$GeneID)]

## add SDF compound name to controls
controls <- data.frame(GeneID = c("ctrl DAPT", "ctrl IWP", "ctrl LY",
                                  "ctrl Paclitaxel", "ctrl PD",  "ctrl RAPA",
                                  "ctrl U0126",   "ctrl Vinblastin",
                                  "ctrl Wortmannin", "ctrl Y27",
                                  "neg ctr DMSO"),
                       Name = c("DAPT", "IWP-2", "LY-294,002 hydrochloride",
                                "Taxol", "PD 98,059", "Sirolimus", "U0126",
                                "Vinblastine sulfate salt",
                                "Wortmannin from Penicillium funiculosum",
                                "Y-27632 dihydrochloride", "neg ctr DMSO"),
                       stringsAsFactors=FALSE)

findNames <- match(controls$GeneID, drugs$GeneID)
drugs$Name[findNames[!is.na(findNames)]] <- controls$Name[!is.na(findNames)]

## get drug names in sdf file and adjust format
drugsInSDF <- datablocktag(sdfset, 5)
drugsInSDF[grep(" $", drugsInSDF)] <-
    gsub(" *$","", drugsInSDF[grep(" $", drugsInSDF)])

## keep only selected drugs and add selected controls twice to sdf file
ctrlToKeep = c("ctrl Paclitaxel", "ctrl U0126", "ctrl Vinblastin")
selectedControls <- match(controls$Name[match(ctrlToKeep, controls$GeneID)],
                          datablocktag(sdfset, 5))
sdfsetControls <- sdfset[selectedControls]
cid(sdfsetControls) <- ctrlToKeep

sdfsetMerged <- c(sdfset[unique(match(drugs$Name, drugsInSDF))] ,
                  sdfsetControls)
selectedDrugsInSDF <- drugsInSDF[unique(match(drugs$Name, drugsInSDF))]

## Structure similarity searching and clustering using atom pairs
## use unique(inSDFfile) because controls could appear twice
## Generate atom pair descriptor database for searching
apset <- sdf2ap(sdfsetMerged)
dummy <- cmp.cluster(db=apset,
                     cutoff=0,
                     save.distances=file.path("result", "distmat.rda"))
load(file.path("result", "distmat.rda"))

## annotate distance matrix with GeneIDs and drugnames
dimnames(distmat) <- list(c(drugs$GeneID[match(selectedDrugsInSDF, drugs$Name)],
```

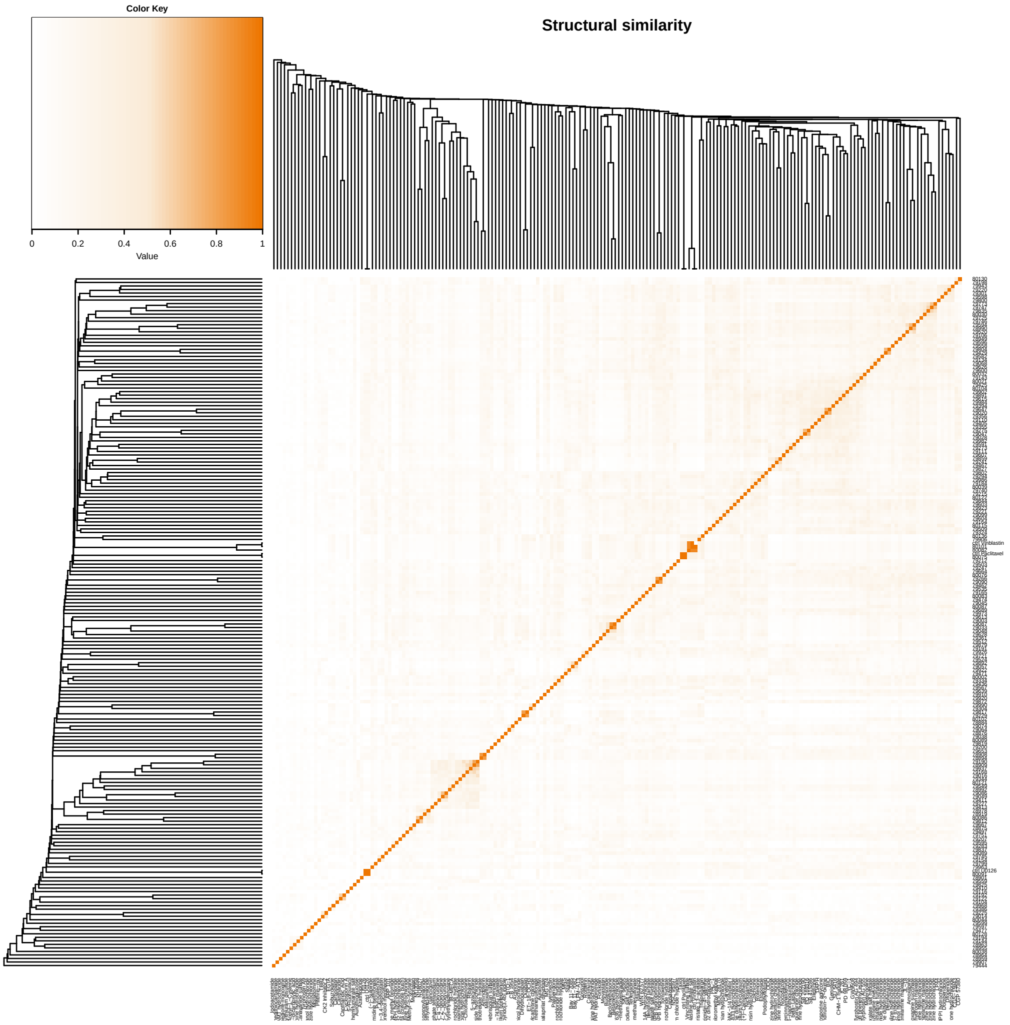

Figure 19: Clustering of drug similarity defined by Tanimoto distance.

```
                                ctrlToKeep),
                              c(selectedDrugsInSDF, ctrlToKeep))

hc <- as.dendrogram(hclust(as.dist(distmat), method="single"))

heatmap.2(1-distmat,
          Rowv=hc,
          Colv=hc,
          col=colorRampPalette(c( "white","antiquewhite", "darkorange2"))(64),
          density.info="none",
          trace="none",
          main="Structural similarity")
```

9.2.1 Chemical similarity heat map ordered by interaction profile similarity

The chemical similarity is plotted ordered by interaction profile similarity.

```
## check correct ordering
drugIds = sapply(strsplit(rownames(PIdist), " "), "[", 1)
drugIds[grep("ctrl", drugIds)] = gsub(" $", "",
                                      rownames(PIdist)[grep("ctrl", drugIds)])
stopifnot(!any(diff(match(drugIds, rownames(distmat))) != 1))
```

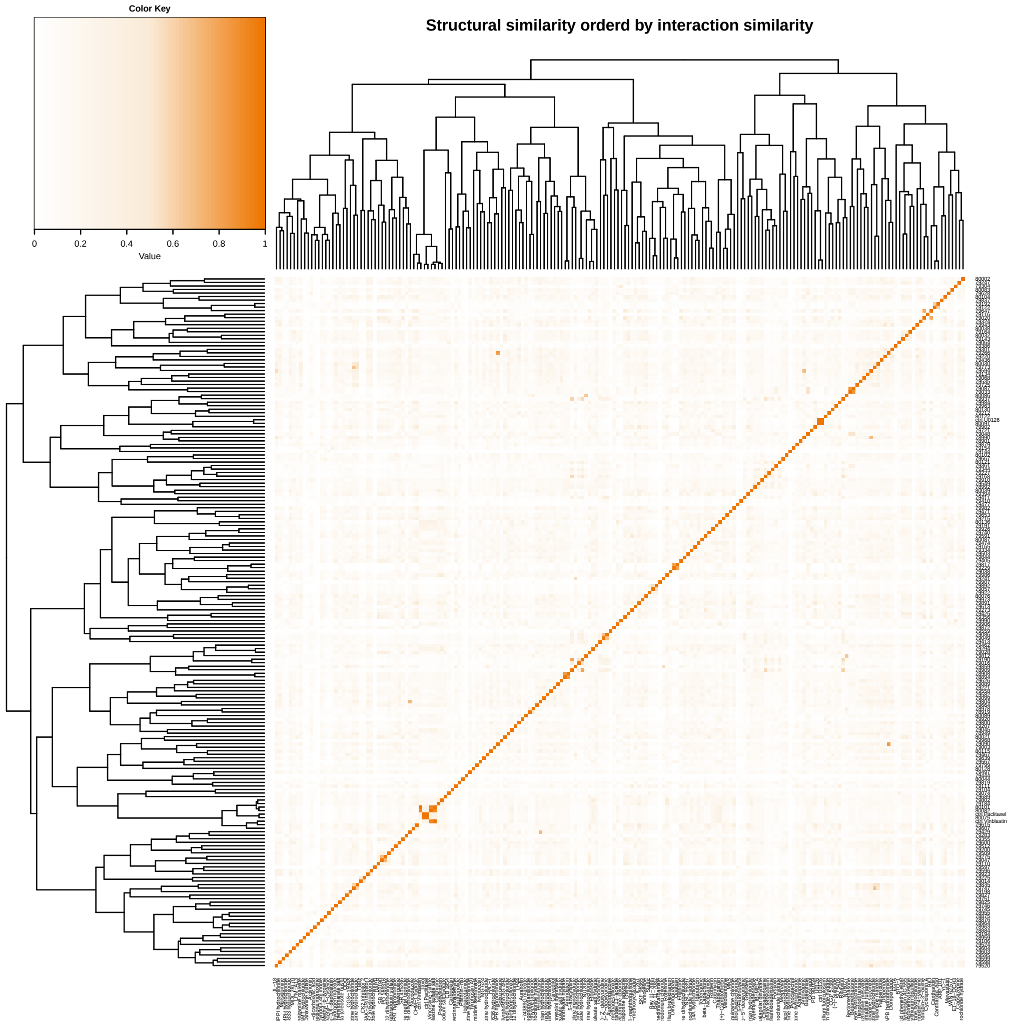

Figure 20: Heatmap of drug similarity defined by Tanimoto distance ordered according to interaction profile similarity.

```
heatmap.2(1-distmat,
          Rowv=hcInt,
          Colv=hcInt,
          col=colorRampPalette(c( "white","antiquewhite", "darkorange2"))(64),
          density.info="none",
          trace="none",
          main="Structural similarity orderd by interaction similarity")
```

9.2.2 Combined cluster heat map

The interaction profile similarity and chemical similarity are combined in one heat map which is ordered by interaction profile similarity.

A list of identified clusters is plotted seperately in the folder result/Figures/ in the working directory.

```
## plot combined cluster heatmap
chemDistOrdered = distmat[order.dendrogram(rev(hcInt)), order.dendrogram(hcInt)]

ordered  = PIdist[order.dendrogram(rev(hcInt)), order.dendrogram(hcInt)]
ordered[nrow(ordered) - row(ordered) + 2 < col(ordered) + 1] =
    -1-chemDistOrdered[nrow(ordered) - row(ordered) + 2 < col(ordered) + 1]
```

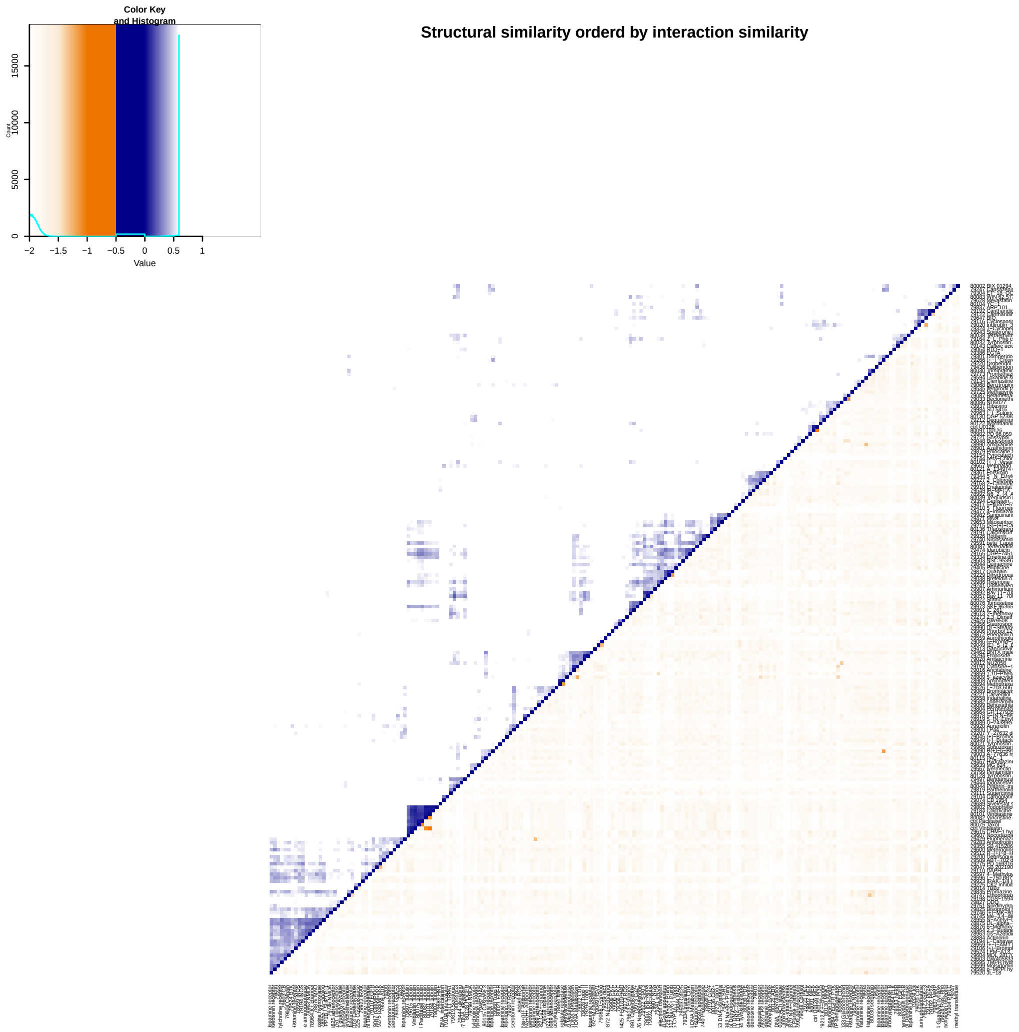

Figure 21: Clustering of drug interaction profiles combined with chemical similarity defined by Tanimoto distance. This figure is the basis of Figure 5A in the paper.

```
heatmap.2(ordered,
          Rowv=FALSE,
          Colv=FALSE,
          dendrogram="none",
          col=c(colorRampPalette(c( "white","antiquewhite", "darkorange2"))(64),
                colorRampPalette(c("darkblue", "white"))(64)),
          breaks = c(seq(-2,-1,length.out=64), -0.5, seq(0,0.6,length.out=64)),
          trace="none",
          main="Structural similarity orderd by interaction similarity")
```

```
cluster = list(C1 = c(79802, 79184, 80101, 80082, "ctrl Paclitaxel", 80075,
                      "ctrl Vinblastin", 79615, 79607),
               C2 = c("ctrl U0126", 80091, 79902),
               C3 = c(79225, 79014),
               C4=c(79275, 79047),
               C5=c(79087, 79033),
               C6=c(78978, 78919),
               C7=c(79411, 79410),
               C8=c(79294, 79028, 79812, 79190, 79016),
               C9=c(79653, 79215, 80136, 79191),
               C10=c(79104, 79074),
```

```
               C11=c(79497, 79444, 80044, 79819, 79111),
               C12=c(79926, 79740),
               C13=c(79164, 80032, 79143),
               C13B=c(80038, 79164, 80032, 79143, 79064),
               C14=c(79817, 79229, 79038),
               C15=c(79165, 79334, 79503),
               C15B=c(79474, 79165, 79334, 79503),
               C16=c(79892, 79057, 79922),
               C17=c(80104, 79837),
               C18=c(79192, 79122, 79647),
               C18B=c(79192, 79122, 79647, 79116, 79020),
               C19=c(78909, 78908, 78894),
               C19B=c(79859, 78909, 78908, 78894))

## get reordered drug ids
drugIds = sapply(strsplit(rownames(ordered), " "), "[", 1)
drugIds[grep("ctrl", drugIds)] = gsub(" $", "",
                                      rownames(ordered)[grep("ctrl", drugIds)])

for(clName in names(cluster)){
  cl = cluster[[clName]]
  pdf(file=file.path("result", "Figures", paste0(clName, ".pdf")),
      width=8,
      height=8)
  heatmap.2(ordered[match(cl, drugIds), rev(match(cl, rev(drugIds)))],
            Rowv=FALSE,
            Colv=FALSE,
            dendrogram="none",
            col=c(colorRampPalette(c("white",
                                     "antiquewhite",
                                     "darkorange2"))(64),
                  colorRampPalette(c("darkblue", "white"))(64)),
            breaks = c(seq(-2,-1,length.out=64),
                       -0.5,
                       seq(0,0.6,length.out=64)),
            trace="none",
            key=FALSE,
            main=clName)
  dev.off()
}
```

9.3 Clustering of interaction profiles using the filtered data of a single parental cell line

To estimate the information obtained from a high-content screen in a single cell line, we looked at drug similarities, using just one parental cell line.

```
## remove controls
singleCellLine = PIfilter[, match("HCT116 P2", dimnames(PIfilter)[[2]]),]

singleDist = PGPC:::getDist(singleCellLine, drugAnno=drugAnno)

hcsingleDist <- as.dendrogram(hclust(as.dist(singleDist)))

heatmap.2(singleDist,
          Colv = hcsingleDist,
          Rowv = hcsingleDist,
          trace="none",
```

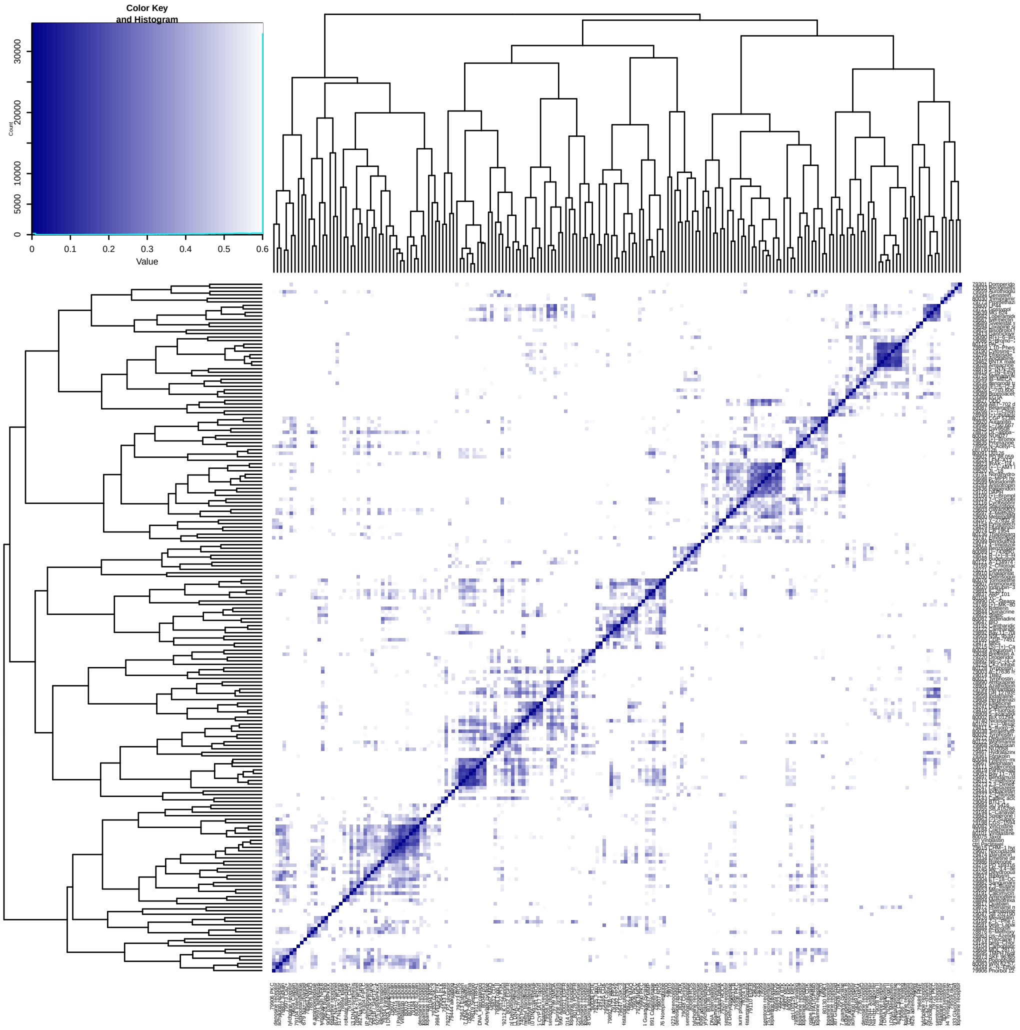

Figure 22: Clustering of drug interaction profiles using data from just one parental cell line. This figure is the basis of Appendix Figure S10 in the paper.

```
          col=colorRampPalette(c("darkblue", "white"))(64),
          breaks = c(seq(0,0.5999,length.out=64),0.6))
```

Next the heatmap for single cell line is ordered by the dendrogram obtained by using all data.

```
## combine controls
heatmap.2(singleDist,
          Colv = hcInt,
          Rowv = hcInt,
          trace="none",
          col=colorRampPalette(c("darkblue", "white"))(64),
          breaks = c(seq(0,0.5999,length.out=64),0.6))
```

9.4 Clustering of interaction profiles using the filtered cell number as feature.

To estimate the information obtained from a pharmaco-genetic screen using only cellular fitness as a readout, we looked at drug similarities, using just the cell number feature of all cell lines.

```
## combine controls
justN = PIfilter[,,match("n", dimnames(PIfilter)[[3]])]
```

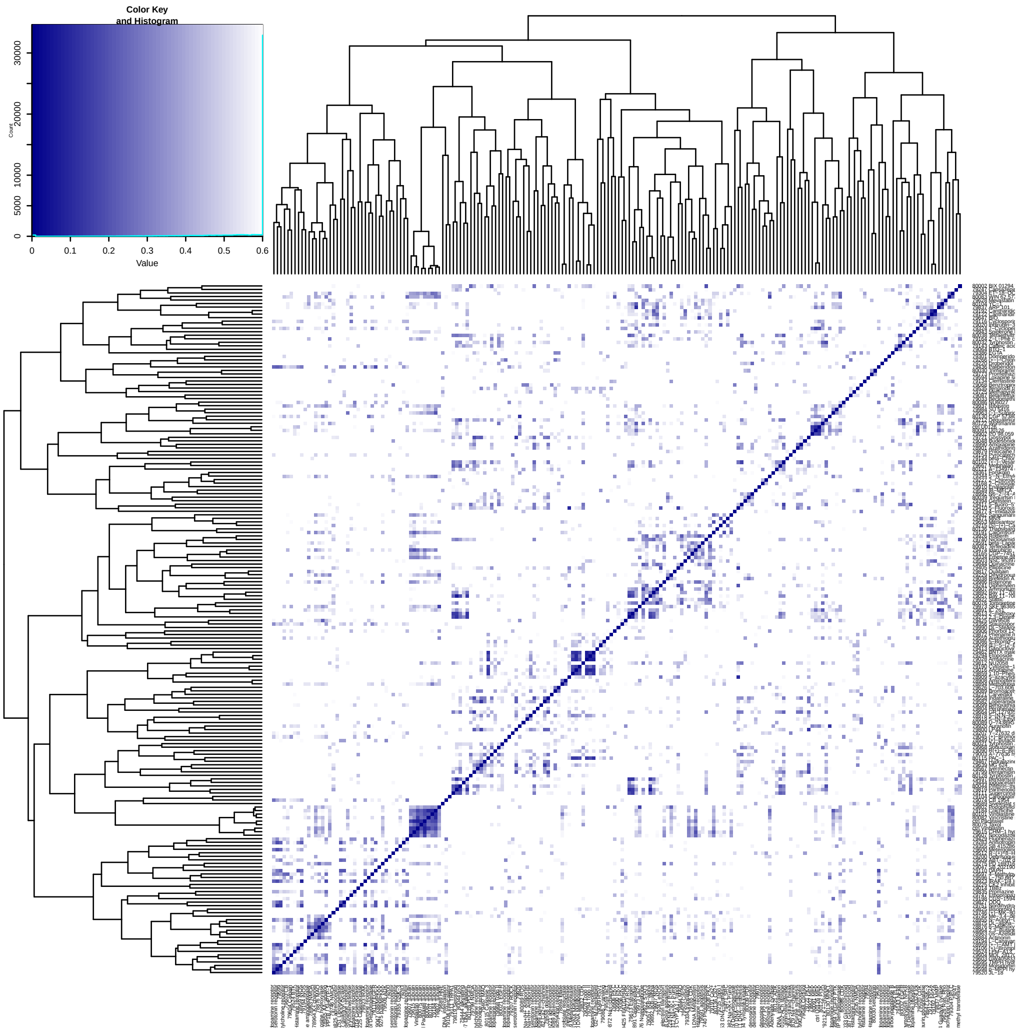

Figure 23: Clustering of drug interaction profiles using data from just one parental cell line ordered by the clustering of the whole data set.

```
justNDist = PGPC:::getDist(justN, drugAnno=drugAnno)

hcjustNDist <- as.dendrogram(hclust(as.dist(justNDist)))

heatmap.2(justNDist,
          Colv = hcjustNDist,
          Rowv = hcjustNDist,
          trace="none",
          col=colorRampPalette(c("darkblue", "white"))(64),
          breaks = c(seq(0,0.5999,length.out=64),0.6))
```

Next the heatmap for only cell number is ordered by the dendrogram obtained by using all data.

```
## combine controls
heatmap.2(justNDist,
          Colv = hcInt,
          Rowv = hcInt,
          trace="none",
          col=colorRampPalette(c("darkblue", "white"))(64),
          breaks = c(seq(0,0.5999,length.out=64),0.6))
```

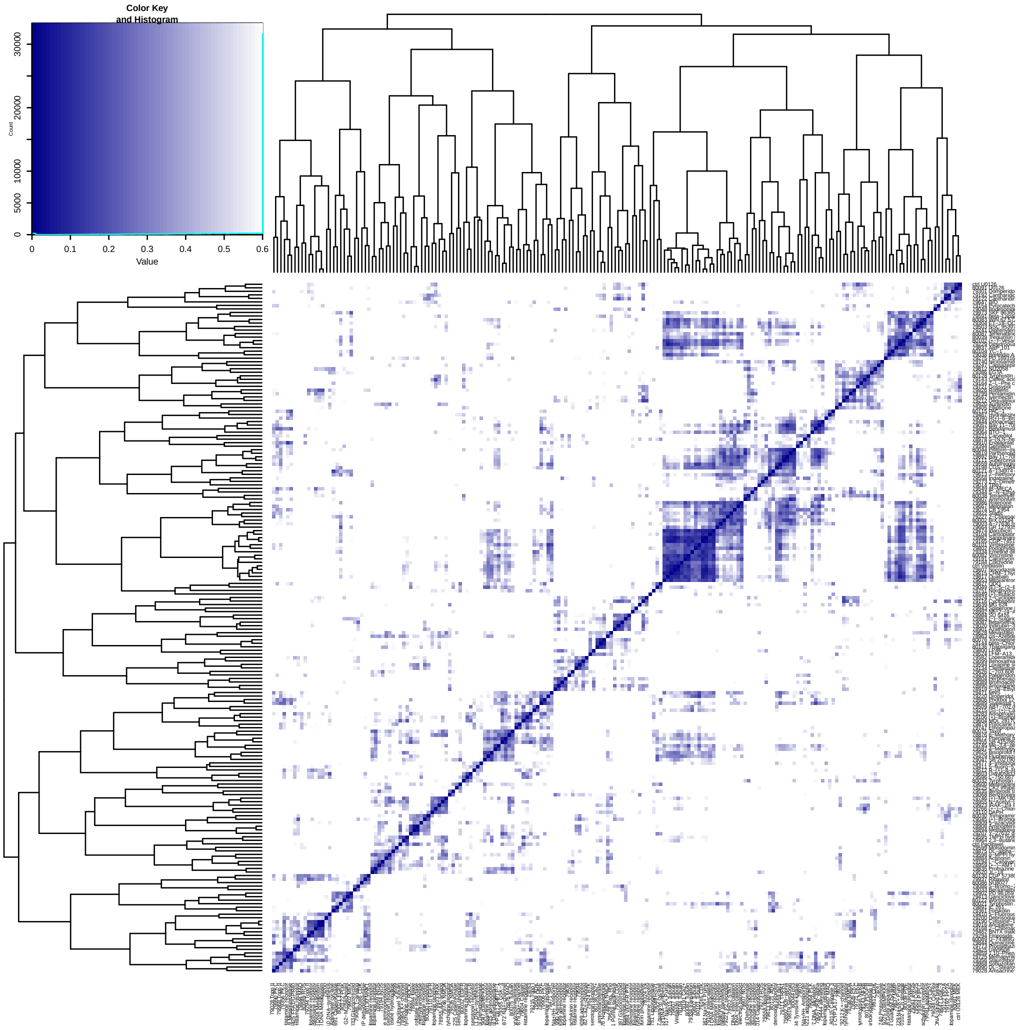

Figure 24: Clustering of drug interaction profiles using just the cell number feature of all cell lines. This figure is the basis of Appendix Figure 13 in the paper.

```
###### check tanimoto for specifc compounds..
grepSDFset("BAY 11-7082", sdfset, field="datablock", mode="subset")
grepSDFset("BAY 11-7085", sdfset, field="datablock", mode="subset")
grepSDFset("Stattic", sdfset, field="datablock", mode="subset")
grepSDFset("Ouabain", sdfset, field="datablock", mode="subset")
grepSDFset("Dihydroouabain", sdfset, field="datablock", mode="subset")
grepSDFset("Brefeldin A", sdfset, field="datablock", mode="subset")

# bay7082 is CMP1018
# bay7085 is CMP183
# stattic is 1048
cmp.similarity(apset["CMP1018"], apset["CMP183"])
cmp.similarity(apset["CMP1018"], apset["CMP1048"])
cmp.similarity(apset["CMP183"], apset["CMP1048"])

plot(sdfset[1018], print=FALSE)
plot(sdfset[183], print=FALSE)
plot(sdfset[1048], print=FALSE)
#plot(sdfset[c("1018","183", "1048", "943")], print=FALSE)
# ouabain is CMP943?
#79229
```

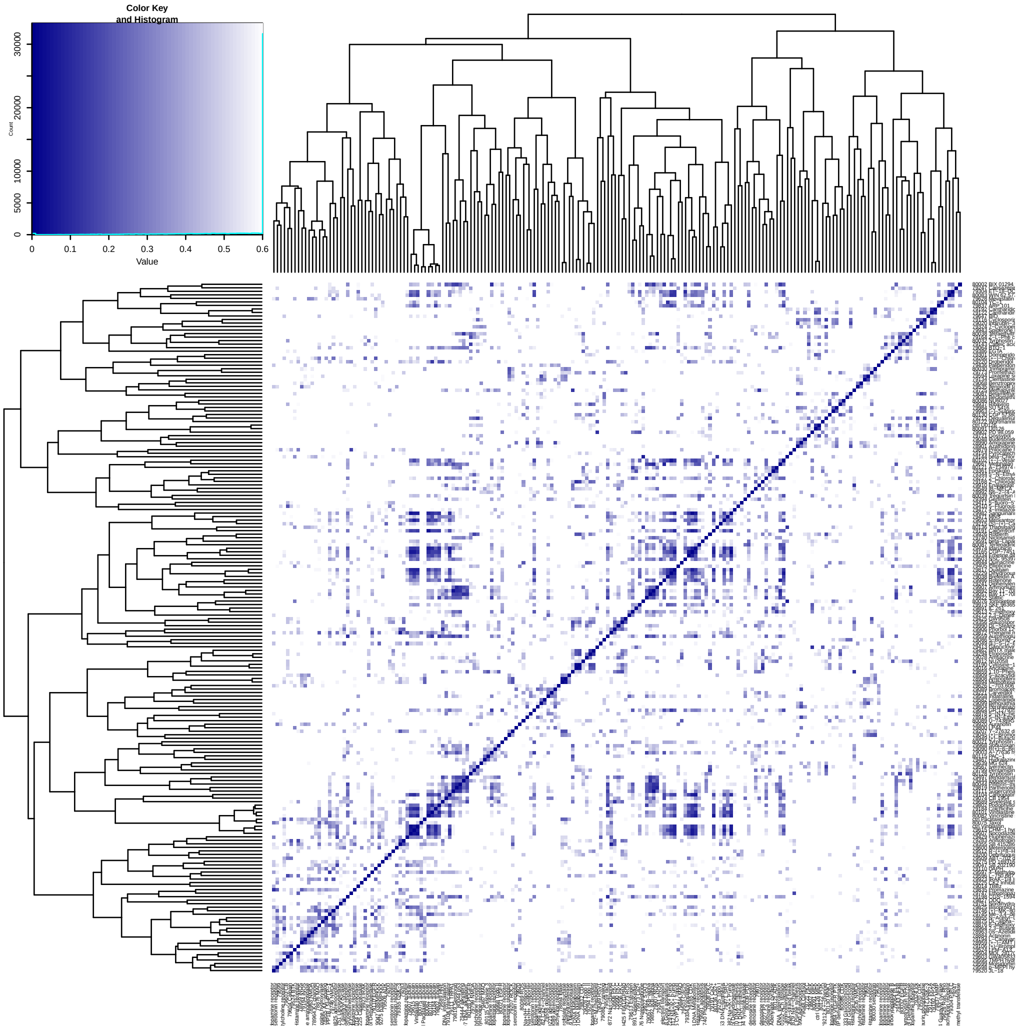

Figure 25: Clustering of drug interaction profiles using just the cell number feature of all cell lines ordered by the clustering of the whole data set.

```
#79817
# Dihydroouabain is CMP355
# brefeldin is 164

cmp.similarity(apset["CMP355"], apset["CMP943"])
cmp.similarity(apset["CMP355"], apset["CMP164"])
cmp.similarity(apset["CMP943"], apset["CMP164"])
```

10 Correlation between interaction profiles of shared drug targets

10.1 Using the target selectivity for grouping

Here we investigate how the correlation of drugs with shared drug targets is distributed. This is done using the interaction profiles of all cell lines and features, all cell lines and only the cell number feature as well as only the parental cell line and all features.

The two groups of drugs with "shared targets" and "non-shared targets" are compared by looking at the difference in the integral of the emperical distribution function of correlation coefficients.

```
## get mean distance for drugs with shared targets
drugAnno = interactions$anno$drug
Selectivity <- drugAnno$Selectivity_updated[match(dimnames(PIfilter)[[1]],
                                                  drugAnno$GeneID)]

annoForDensity = drugAnno[match(dimnames(PIfilter)[[1]],
                                 drugAnno$GeneID),]

plotDensities <- function(d, anno, ...){
  M = PGPC:::getCorr(d, drugAnno=anno)
  sharedClass <- array(FALSE, dim=dim(M))
  for(target in unique(Selectivity)){
    if(is.na(target)) next
    targetdrugs = anno$GeneID[anno$Selectivity_updated %in% target]
    if(length(targetdrugs)>1){
      sel = dimnames(M)[[1]] %in% targetdrugs
      sharedClass[sel, sel] = TRUE
    }
  }
  diag(sharedClass) = TRUE
  notSharedDist = M[!sharedClass]

  diag(sharedClass) = FALSE
  sharedDist = M[sharedClass]

  classDist = c(sharedDist, notSharedDist)
  class = c(rep("shared selectivity", length(sharedDist)),
            rep("no shared selectivity", length(notSharedDist)))

  multidensity(classDist ~ class,
               xlab="Correlation between drug profiles",
               ylim=c(0,2.5),
               col=c("blue", "red"),
               ...)
  tmp <- multiecdf(classDist ~ class,
                   xlab="Correlation between drug profiles",
                   subsample=FALSE,
                   col=c("blue", "red"),
                   ...)

  integrals <- sapply(lapply(tmp,
                             integrate,
                             lower=min(classDist),
                             upper=max(classDist),
                             subdivisions=length(table(classDist))),
                      function(x) x$value)

  ## add class numbers
  integrals = c(integrals, N=table(class))

  corOrder = order(classDist, decreasing=TRUE)
  classDist = classDist[corOrder]
  class = class[corOrder]

  ratio = (cumsum(class=="shared selectivity"))/
    (cumsum(class=="shared selectivity") +
       cumsum(class=="no shared selectivity"))
```

```
  plot(classDist, ratio,
       xlab="correlation coefficient",
       ylab="shared target fraction",
       ylim=c(0,1),
       type="l",
       ...)
  abline(h = c(.1, .2, .3), col="grey", lty=2)
  text(0, 0.3, sum(ratio >= 0.3))
  text(0, 0.2, sum(ratio >= 0.2))
  text(0, 0.1, sum(ratio >= 0.1))

  integrals
}
```

```
intAll <- plotDensities(PIfilter,
                        annoForDensity, lwd=5,
                        xlim=c(-1,1),
                        main=paste("ctrl combined all data,",
                                   "all isogenics and all features"))
```

```
intN <- plotDensities(justN,
                      annoForDensity, lwd=5,
                      xlim=c(-1,1),
                      main="all isogenics, only cell number")
```

```
intSingle <- plotDensities(singleCellLine,
                           annoForDensity, lwd=5,
                           xlim=c(-1,1),
                           main="all features, only parental cell line")
```

```
integrals <- as.data.frame(rbind(intAll, intN, intSingle))
integrals$type = c("all data", "only cell number,\nall cell lines",
                   "only parental cell line,\nall feature")

theme_new = theme_bw()
theme_new$text$size = 20
theme_new$axis.text$size = rel(1.2)

theme_new = theme_new +
  theme(axis.text.x = element_text(angle = 45, hjust = 1))
p = ggplot(data=melt(integrals,
                     measure.vars=c("no shared selectivity",
                                    "shared selectivity")),
           aes(x=type,
               y= value,
               fill=variable))
dodge <- position_dodge(.5)

p = p + geom_bar(stat="identity", position=dodge, width=.5) +
  theme_new +
  scale_fill_manual(values = c("blue", "red"))
print(p)

## difference
integrals$difference = integrals$"no shared selectivity" -
    integrals$"shared selectivity"
integrals$similarity = "Target selectivity"

p = ggplot(data=integrals,
```

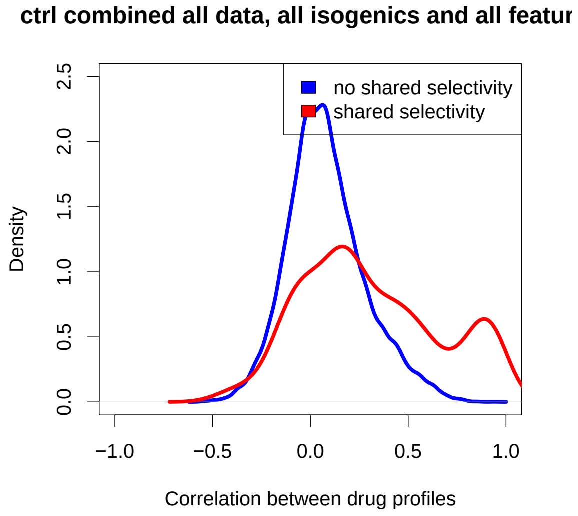

(a) Density distribution of correlation coefficients per class

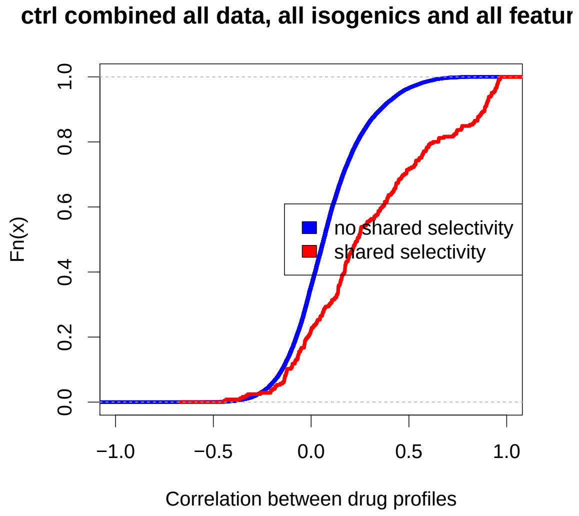

(b) Cumulative distribution of correlation coefficients per class.

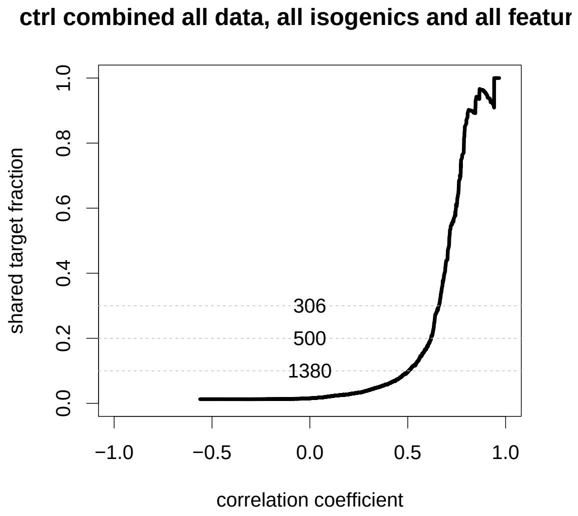

(c) Fraction of drugs with shared targets plotted against the correlation coefficients.

Figure 26: Distribution of correlation coefficients of the interaction profiles for the whole data set. Pannel (b) is the basis of Figure 5B in the paper.

```
           aes(x=type,
               y=difference))
p = p + geom_bar(stat="identity", width=.5) +
  theme_new +
  labs(title = "Target selectivity") +
  scale_y_continuous(limits = c(0,.3))
print(p)
```

10.2 Using the chemical similarity for grouping

Here we use the structure similarity to look whether the interaction profiles of drugs with similar structure show an increased correlation. We use a threshold of 0.6 on the Tanimoto distances between compounds calculated with the

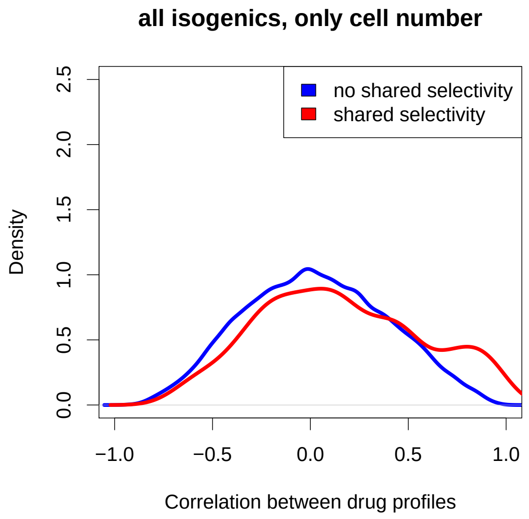

(a) Density distribution of correlation coefficients per class

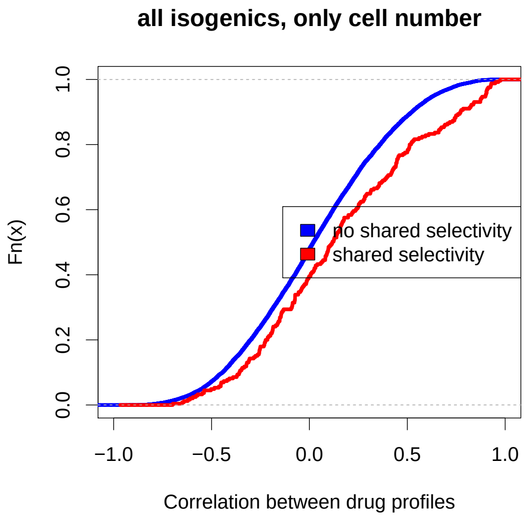

(b) Cumulative distribution of correlation coefficients per class.

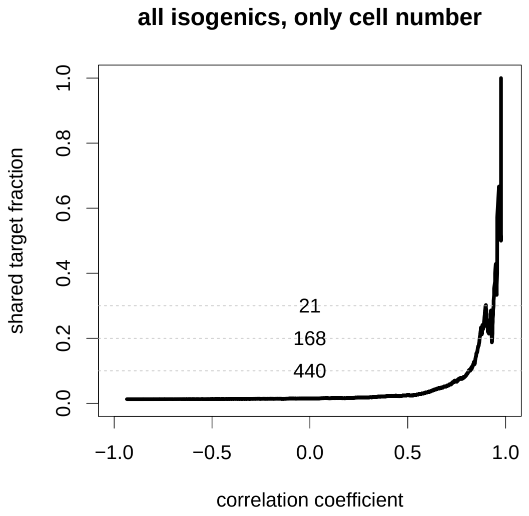

(c) Fraction of drugs with shared targets plotted against the correlation coefficients.

Figure 27: Distribution of correlation coefficients of the interaction profiles using only cell number for all cell lines. Pannel (b) is the basis of Figure 5B in the paper.

ChemmineR package.

The analysis of the previous section is repeated for the two groups "similar structure" and "different structure".

```
## get mean distance for drugs with shared targets
plotDensityTanimotoThresh <- function(profile,
                                      distmat,
                                      tanimotoThresh = 0.6,
                                      ...){

  similarStructure = distmat < tanimotoThresh
  M = PGPC:::getCorr(profile, drugAnno=drugAnno)

  diag(similarStructure) = TRUE
```

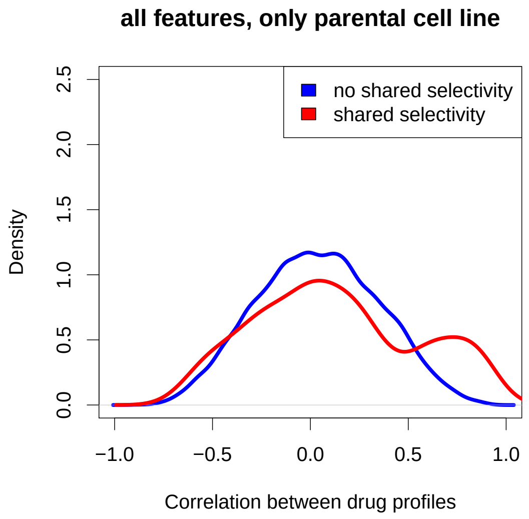

(a) Density distribution of correlation coefficients per class

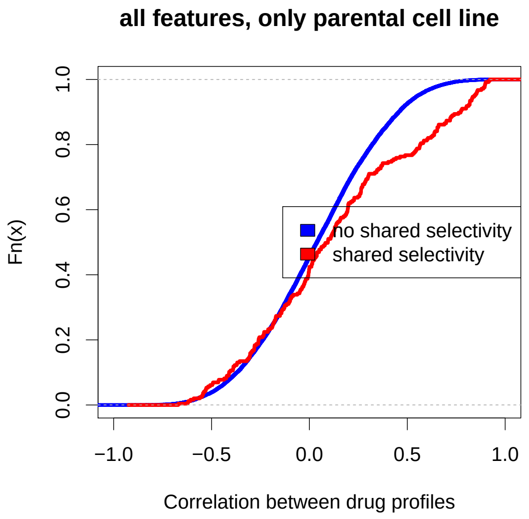

(b) Cumulative distribution of correlation coefficients per class.

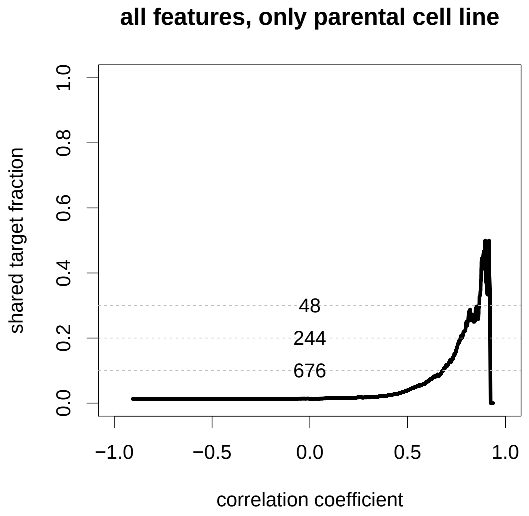

(c) Fraction of drugs with shared targets plotted against the correlation coefficients.

Figure 28: Distribution of correlation coefficients of the interaction profiles using all features of only one parental cell line. Pannel (b) is the basis of Figure 5B in the paper.

```
notSharedDist = M[!similarStructure]

diag(similarStructure) = FALSE
sharedDist = M[similarStructure]

classDist = c(sharedDist, notSharedDist)
class = c(rep("similar structure", length(sharedDist)),
          rep("different structure", length(notSharedDist)))

multidensity(classDist ~ class,
             xlab="Correlation between drug profiles",
             ylim=c(0,2.5),
             col=c("blue", "red"),
```

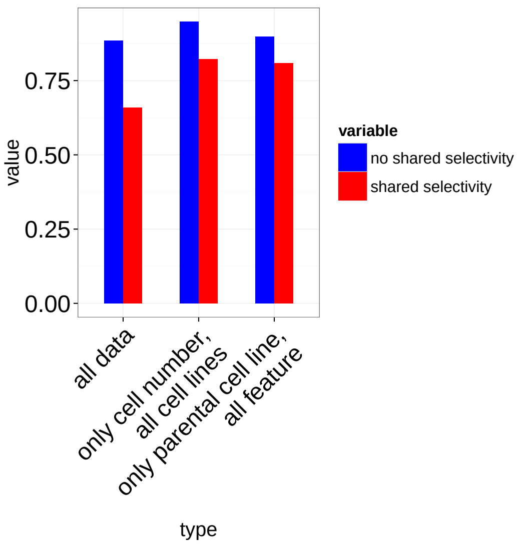

(a) Direct comparison of the AUC for each class and data set.

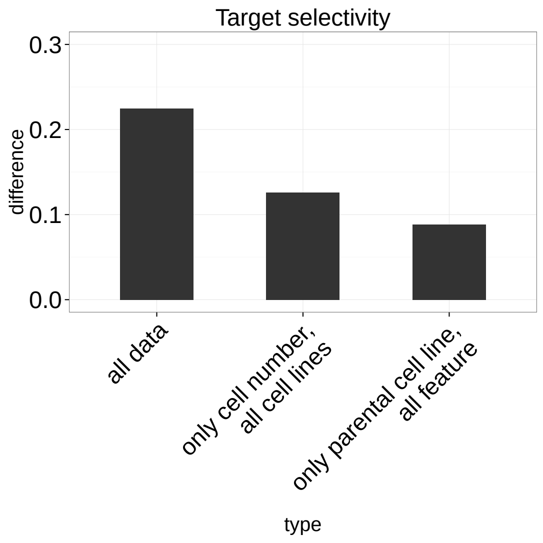

(b) Comparison of the differece in AUC between classes for the different data sets. This figure ist the basis for Figure 5C in the paper.

Figure 29: Comparison of the area under the curve (AUC) of the cummulative distribution function from the different data sets and classes defined by the target selectivity.

```
              ...)
tmp <- multiecdf(classDist ~ class,
                 xlab="Correlation between drug profiles",
                 subsample=FALSE,
                 col=c("blue", "red"),
                 ...)

integrals <- sapply(lapply(tmp,
                           integrate,
                           lower=min(classDist),
                           upper=max(classDist),
                           subdivisions=length(table(classDist))),
                    function(x) x$value)

## add class numbers
integrals = c(integrals, N=table(class))

corOrder = order(classDist, decreasing=TRUE)
classDist = classDist[corOrder]
class = class[corOrder]

ratio = (cumsum(class=="similar structure"))/
  (cumsum(class=="similar structure") + cumsum(class=="different structure"))

plot(classDist, ratio,
     xlab="correlation coefficient",
     ylab="shared target fraction",
     ylim=c(0,1),
     type="l",
     ...)
abline(h = c(.1, .2, .3), col="grey", lty=2)
text(0, 0.3, sum(ratio >= 0.3))
```

```
  text(0, 0.2, sum(ratio >= 0.2))
  text(0, 0.1, sum(ratio >= 0.1))

  integrals
}

## reorder according to drug order in ctrlCombined
stopifnot(!any(diff(match(dimnames(PIfilter)[[1]], rownames(distmat))) != 1))

tanimotoThresh = 0.6
```

```
intAllTanimoto <- plotDensityTanimotoThresh(PIfilter, distmat,
                                            tanimotoThres=tanimotoThresh, lwd=5,
                                            xlim=c(-1,1),
                                            main=paste0("all data,",
                                                        " Tanimoto dist < ",
                                                        tanimotoThresh))
```

```
intNTanimoto <- plotDensityTanimotoThresh(justN, distmat,
                                          tanimotoThres=tanimotoThresh,lwd=5,
                                          xlim=c(-1,1),
                                          main=paste("all isogenics,",
                                                     "cellnumber only,",
                                                     "Tanimoto dist < ",
                                                     tanimotoThresh))
```

```
intSingleTanimoto <- plotDensityTanimotoThresh(singleCellLine, distmat,
                                               tanimotoThres=tanimotoThresh,
                                               lwd=5,
                                               xlim=c(-1,1),
                                               main=paste("single cell line,",
                                                          "Tanimoto dist < ",
                                                          tanimotoThresh))
```

```
integralsTanimoto <- as.data.frame(rbind(intAllTanimoto,
                                         intNTanimoto,
                                         intSingleTanimoto))
integralsTanimoto$type = c("all data",
                           "only cell number,\nall cell lines",
                           "only parental cell line,\nall feature")

theme_new = theme_bw()
theme_new$text$size = 20
theme_new$axis.text$size = rel(1.2)

theme_new = theme_new +
  theme(axis.text.x = element_text(angle = 45, hjust = 1))
p = ggplot(data=melt(integralsTanimoto,
                     measure.vars=c("different structure",
                                    "similar structure")),
           aes(x=type,
               y= value,
               fill=variable))
dodge <- position_dodge(.5)

p = p + geom_bar(stat="identity", position=dodge, width=.5) +
  theme_new +
  scale_fill_manual(values = c("blue", "red"))
print(p)
```

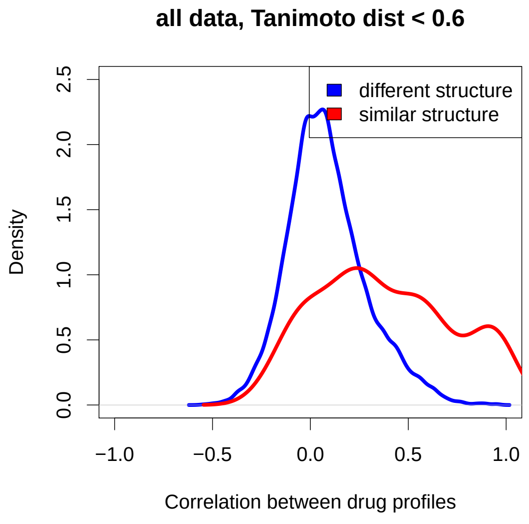

(a) Density distribution of correlation coefficients per class

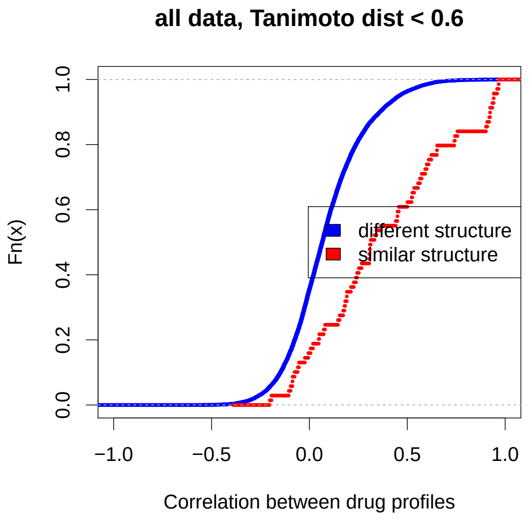

(b) Cumulative distribution of correlation coefficients per class.

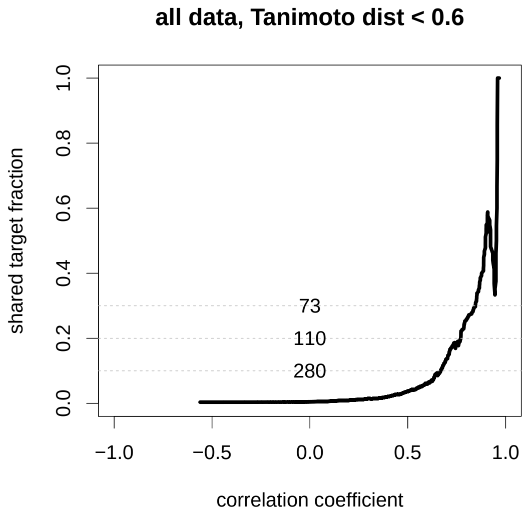

(c) Fraction of drugs with shared targets plotted against the correlation coefficients.

Figure 30: Distribution of correlation coefficients of the interaction profiles for the whole data set. Pannel (b) is the basis of Appendix Figure S11A in the paper.

```
## difference
integralsTanimoto$difference = integralsTanimoto$"different structure" -
    integralsTanimoto$"similar structure"
integralsTanimoto$similarity = "Chemical similarity"

p = ggplot(data=integralsTanimoto,
           aes(x=type,
               y=difference))
p = p + geom_bar(stat="identity", width=.5) +
  theme_new +
  labs(title = "Chemical similarity") +
```

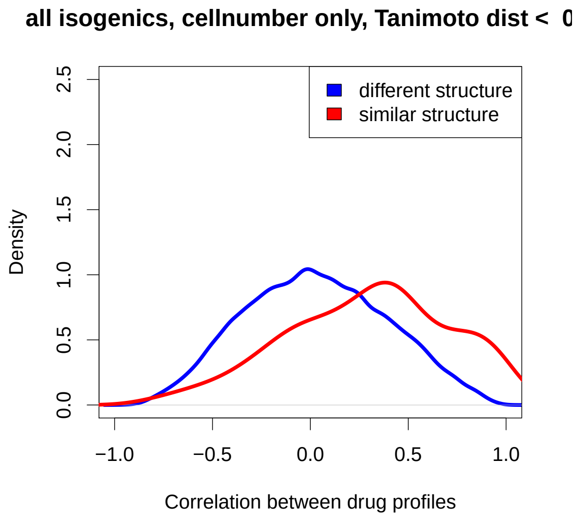

(a) Density distribution of correlation coefficients per class

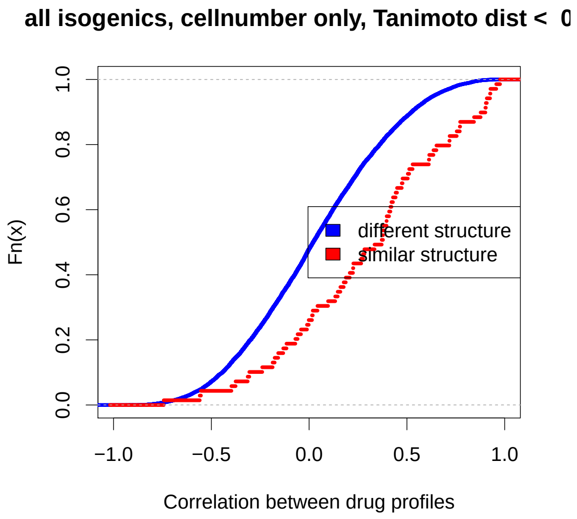

(b) Cumulative distribution of correlation coefficients per class.

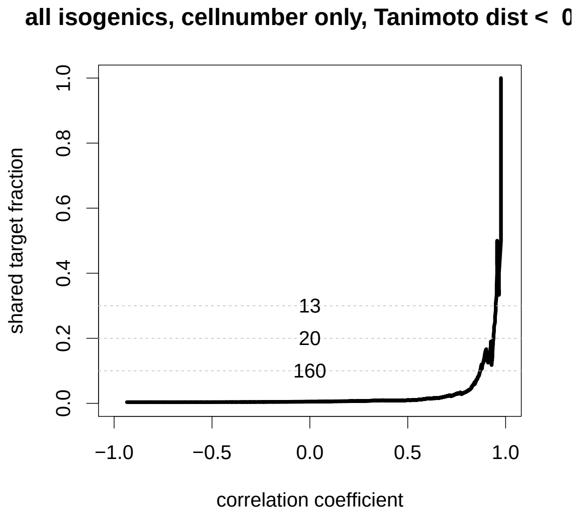

(c) Fraction of drugs with shared targets plotted against the correlation coefficients.

Figure 31: Distribution of correlation coefficients of the interaction profiles using only cell number for all cell lines. Pannel (b) is the basis of Appendix Figure S11A in the paper.

```
  scale_y_continuous(limits = c(0,.3))
print(p)

cols = c("type", "difference", "similarity")
p = ggplot(data=rbind(integrals[,cols], integralsTanimoto[,cols]),
           aes(x=type,
               y= difference,
               fill=similarity))
dodge <- position_dodge(.5)

p = p + geom_bar(stat="identity", position=dodge, width=.5) +
  theme_new +
  scale_fill_manual(values = c("grey", "black"))
```

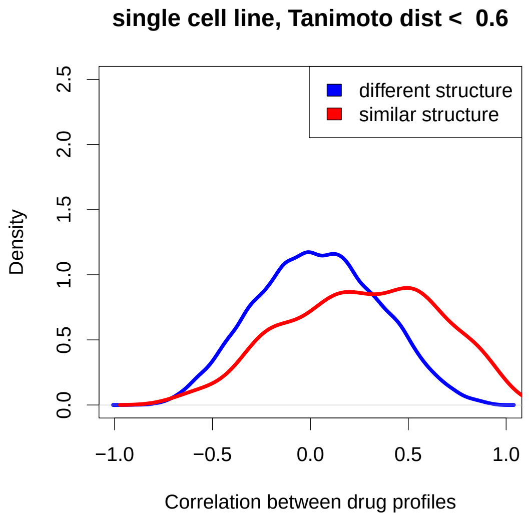

(a) Density distribution of correlation coefficients per class

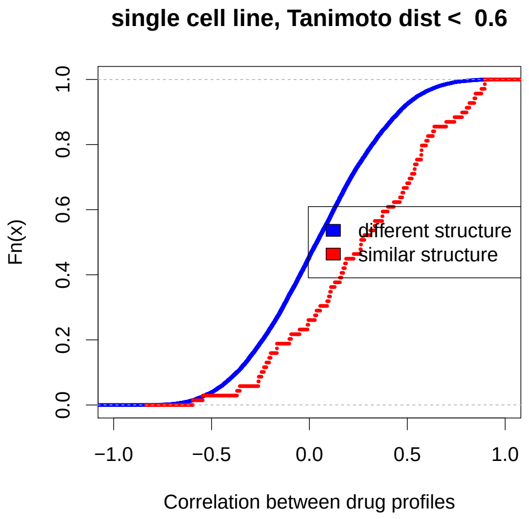

(b) Cumulative distribution of correlation coefficients per class.

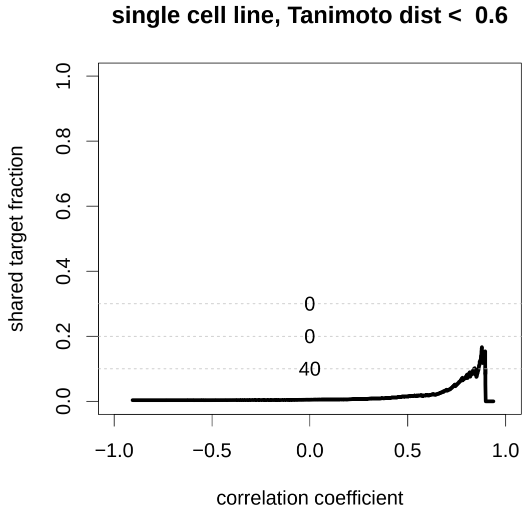

(c) Fraction of drugs with shared targets plotted against the correlation coefficients.

Figure 32: Distribution of correlation coefficients of the interaction profiles using all features of only one parental cell line. Pannel (b) is the basis of Appendix Figure S11A in the paper.

```
print(p)
```

11 Follow-up: Drug combinations

In this follow-up experiment we tested synergistic drug combinations that we predicted based on the chemical-genetic interaction data.

First we use *cellHTS2* to normalize the data. The data is log transformed and normalized using the NPI method of the *cellHTS2*.

```
savePath="result"
dataPath=system.file("extdata", "drug_combinations",
```

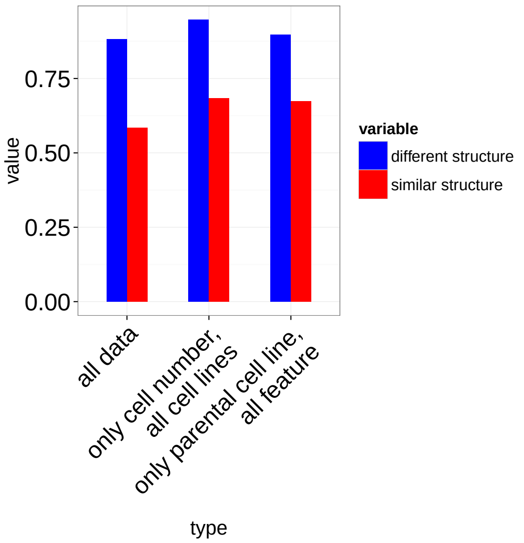

(a) Direct comparison of the AUC for each class and data set.

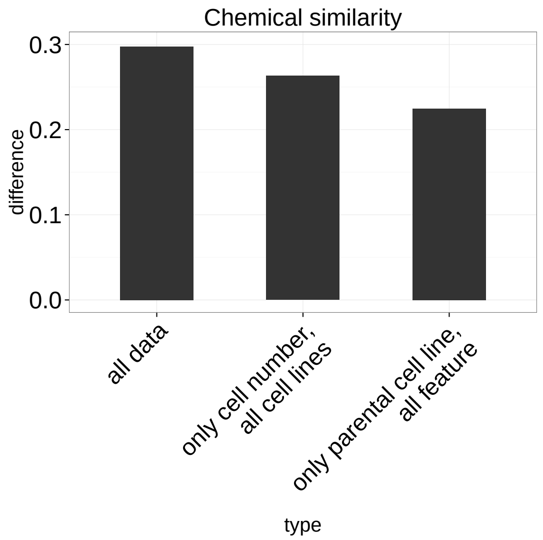

(b) Comparison of the differece in AUC between classes for the different data sets. This figure ist the basis for Appendix Figure S11B in the paper.

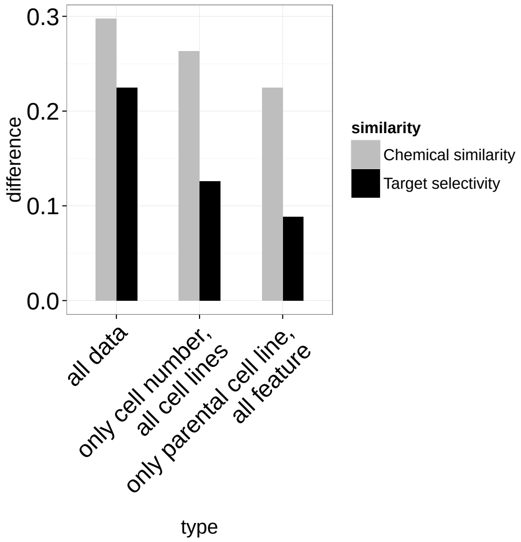

(c) Comparison of the differece in AUC between classes for the different data sets and classes defined by target selectivity or structural similarity.

Figure 33: Comparison of the area under the curve (AUC) of the cummulative distribution function from the different data sets and classes defined by the structural similarity.

```
                      package="PGPC")
Name="drug_combis"
LogTransform=TRUE
Annotation="2013-10-22drugcombiAnno_2plates.txt"
Plateconf="2013-10-22plateconfig_all.txt"
Description="Description.txt"
NormalizationMethod="NPI"
NormalizationScaling="multiplicative"
VarianceAdjust="none"
SummaryMethod="mean"
Score="zscore"
```

```
PlateList=c("2013-10-22platelist_HCT_72_2layout_8plates_mod.txt",
            "2013-10-22platelist_DLD_72_2layout_8plates.txt")

## include intensities
mySettings = getSettings()
mySettings$plateList$intensities$include = TRUE
setSettings(mySettings)
```

```
for(plate in PlateList){
  Outdir = file.path(savePath,
                     paste(gsub("_2layout_8plates.txt|_2layout_8plates_mod.txt",
                                "", plate),
                           NormalizationMethod,
                           VarianceAdjust, sep = "_"))
  x = readPlateList(plate,path=dataPath, name=Name)
  x = configure(x, descripFile=Description,
                confFile=Plateconf,,
                path=dataPath)
  xp = normalizePlates(x,
                       log=LogTransform,
                       scale=NormalizationScaling,
                       method=NormalizationMethod,
                       varianceAdjust=VarianceAdjust)
  xp@state["normalized"] = TRUE
  xsc = scoreReplicates(xp, sign = "-",
                        method = Score)
  xsc = summarizeReplicates(xsc,
                            summary = SummaryMethod)
  xsc = cellHTS2::annotate(xsc,
                           geneIDFile = file.path(dataPath,
                                                  Annotation))
  out = writeReport(raw = x,
                    normalized = xp,
                    scored = xsc,
                    outdir=Outdir,
                    force=TRUE)
}
## Warning:  The R script which produced this cellHTS2 report has not been provided via the 'mainScript
argument.
## We recommend storing this script for future reference along with the report.
## Warning:  The R script which produced this cellHTS2 report has not been provided via the 'mainScript
argument.
## We recommend storing this script for future reference along with the report.
```

11.1 Quality control

To check the quality of the data we create scatter plots of replicates and overview plots of raw and normalized values vs. concentration for each drug combination. From this analysis we found that replicate 4 of the HCT screen did not correlate with the other plates. It was therefore removed from the analysis.

```
experiment = "2013-10-22platelist_HCT_72_NPI_none"

## get the dataframe and check the data in an explorative manner
df = read.delim(file.path("result", experiment, "in", "topTable.txt"),
                stringsAsFactors=FALSE)
```

```
df$identifier = paste(df$drug1, df$drug2, sep="_")
#  head(df)

# plot correlations
p = ggplot(data=df, aes(x=raw_r1_ch1, y= raw_r2_ch1))
p = p + geom_point() +
  labs(title = gsub("_", " ", gsub("2013-10-22platelist_", "", experiment)))
print(p)

p = ggplot(data=df, aes(x=raw_r2_ch1, y= raw_r3_ch1))
p = p + geom_point() +
  labs(title = gsub("_", " ", gsub("2013-10-22platelist_", "", experiment)))
print(p)

p = ggplot(data=df, aes(x=raw_r1_ch1, y= raw_r3_ch1))
p = p + geom_point() +
  labs(title = gsub("_", " ", gsub("2013-10-22platelist_", "", experiment)))
print(p)

p = ggplot(data=df, aes(x=raw_r4_ch1, y= raw_r5_ch1))
p = p + geom_point() +
  labs(title = gsub("_", " ", gsub("2013-10-22platelist_", "", experiment)))
print(p)

## Warning:  Removed 384 rows containing missing values (geom_point).

p = ggplot(data=df, aes(x=raw_r1_ch1, y= raw_r5_ch1))
p = p + geom_point(na.rm=TRUE) +
  labs(title = gsub("_", " ", gsub("2013-10-22platelist_", "", experiment)))
print(p)

# plot all
p = ggplot(data=melt(df, measure.vars=names(df)[grep("raw_r", names(df))]),
           aes(x=concdrug1.micro_M., y=value, color=variable))
p = p + facet_wrap(~identifier) +
  scale_x_log10() +
  geom_point(na.rm=TRUE) +
  theme_bw() +
  labs(title = gsub("_", " ", gsub("2013-10-22platelist_", "", experiment)))
print(p)

p = ggplot(data=melt(df,
                     measure.vars=names(df)[grep("normalized_r", names(df))]),
           aes(x=concdrug1.micro_M., y=value, color=variable))
p = p + facet_wrap(~identifier) +
  scale_x_log10() +
  geom_point(na.rm=TRUE) +
  theme_bw() +
  labs(title = gsub("_", " ", gsub("2013-10-22platelist_", "", experiment)))
print(p)
```

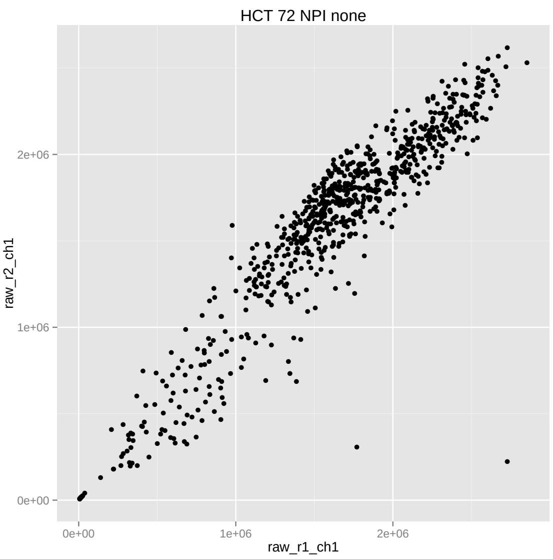
HCT 72 NPI none
raw_r2_ch1
raw_r1_ch1
0e+00
1e+06
2e+06

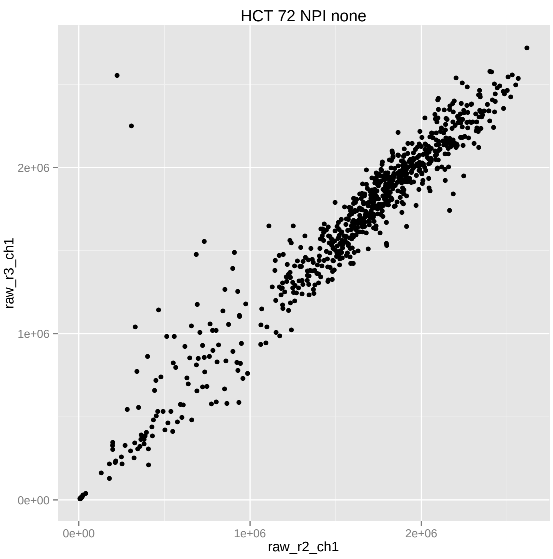
HCT 72 NPI none
raw_r3_ch1
raw_r2_ch1
0e+00
1e+06
2e+06

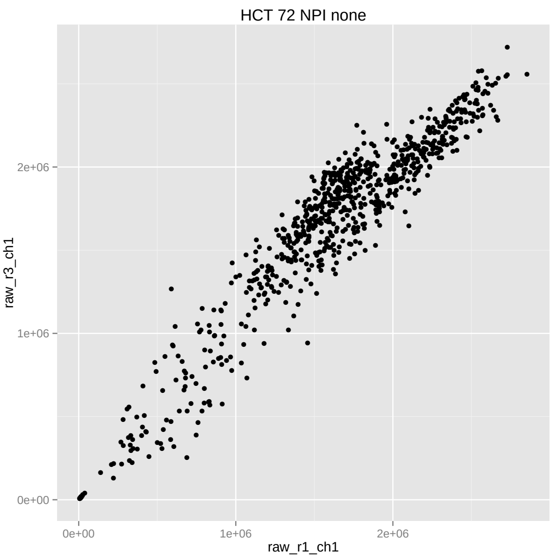
HCT 72 NPI none
raw_r3_ch1
raw_r1_ch1
0e+00
1e+06
2e+06

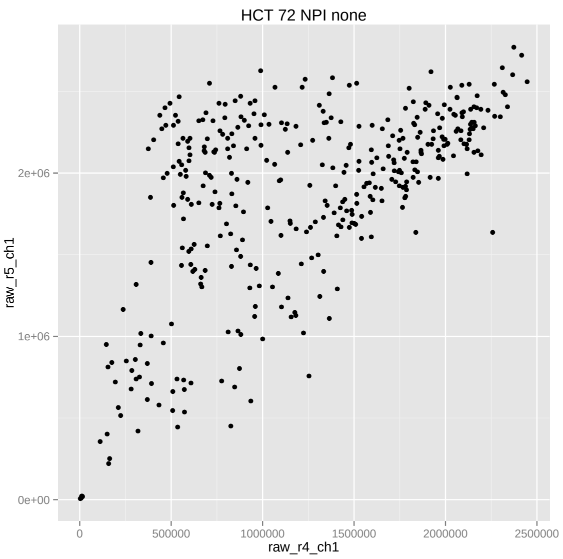
HCT 72 NPI none
raw_r5_ch1
raw_r4_ch1
0e+00
1e+06
2e+06
0
500000
1000000
1500000
2000000
2500000

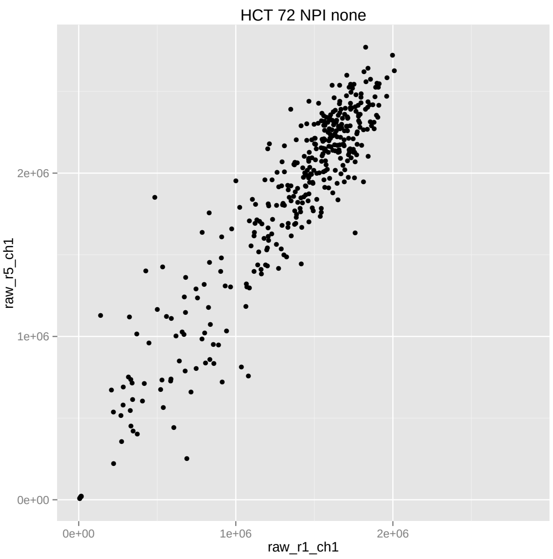
HCT 72 NPI none
raw_r5_ch1
raw_r1_ch1
0e+00
1e+06
2e+06

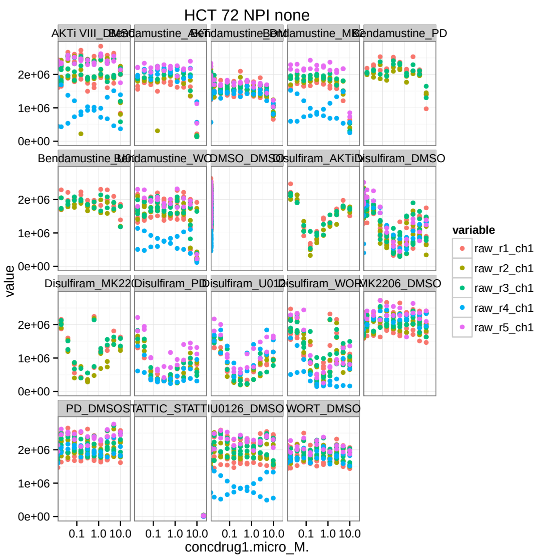
HCT 72 NPI none
value
concdrug1.micro_M.
0.1 1.0 10.0
0e+00
1e+06
2e+06
variable
raw_r1_ch1
raw_r2_ch1
raw_r3_ch1
raw_r4_ch1
raw_r5_ch1
Disulfiram_DMSO
MK2206_DMSO
PD_DMSO
U0126_DMSO
WORT_DMSO

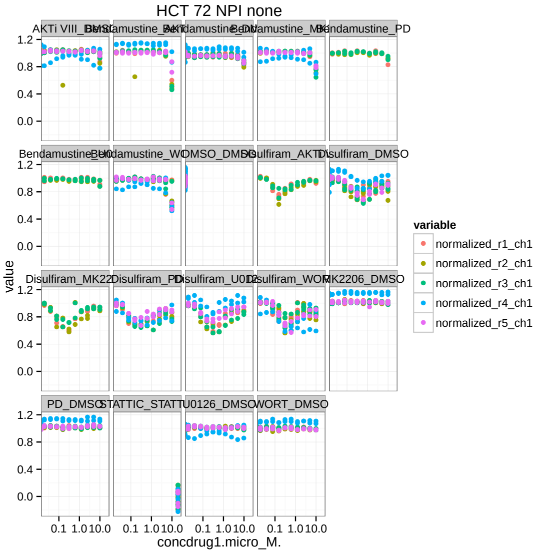

11.2 Interactions / Synergies

11.2.1 Analysis functions

We first define the functions to calculated interactions and plot the results for a given drug combination.

For each drug combination we look for synergies at all used concentrations. The values for the drug combinations are compared with the expected values using either a multiplicative model (additive on log scale) or the HSA (highest single agent) model [9, 10].

In terms of the multiplicative model the expected value is the sum of the individual inhibitions calculated on log transformed values.

$$I_{combination} = I_{drug\ A} + I_{drug\ B} \tag{2}$$

From the NPI values the inhibition is calculated by 1 - NPI

$$I_{drug\ x} = 1 - NPI_{drug\ x} \tag{3}$$

The function `getExpectedValue` calucaltes these values from the data for the provided drug combination and type of interacton model.

```
getExpectedValue <- function(df,
                             position1,
                             position2,
                             drugNames,
                             type=c("nonInteraction", "HSA")){
  normCols = c("normalized_r1_ch1",
               "normalized_r2_ch1" ,
               "normalized_r3_ch1",
               "normalized_r4_ch1" ,
               "normalized_r5_ch1")
  normCols <- normCols[normCols %in% names(df)]

  if(type=="nonInteraction"){
    tmp <- df[position1,]
    tmp[,normCols] <- tmp[,normCols] + df[position2,normCols] - 1
    tmp$identifier = "expected"
    tmp$GeneID = gsub(drugNames[1], "expected", tmp$GeneID)
    tmp$drug1 = gsub(drugNames[1], "expected", tmp$GeneID)
    tmp$color = "white"
```

```
  } else if(type=="HSA"){
    ## test which single compound has the stronges mean effect and use this as expected
    mean1 <- tapply(unlist(df[position1,normCols]),
                    rep(df$concdrug1.micro_M.[position1], length(normCols)),
                    mean,
                    na.rm=TRUE)
    mean2 <- tapply(unlist(df[position2,normCols]),
                    rep(df$concdrug1.micro_M.[position2], length(normCols)),
                    mean,
                    na.rm=TRUE)

    ## take values of drug1 and exchange if drug2 shows a stronger effect for a conc
    tmp <- df[position1,]

    concToChange <- names(mean1[mean1 > mean2])

    for(conc in concToChange){
      concPos <- df$concdrug1.micro_M.[position2] == as.numeric(conc)
      tmp[concPos,normCols] <- df[position2[concPos],normCols]
    }

    tmp$identifier = "expected"
    tmp$GeneID = gsub(drugNames[1], "expected", tmp$GeneID)
    tmp$drug1 = gsub(drugNames[1], "expected", tmp$GeneID)
    tmp$color = "white"
  } else {
    stop("type must be 'nonInteraction' or 'HSA'.")
  }

  columnsToKeep <- c("plate",
                     normCols,
                     "drug1",
                     "concdrug1.micro_M.",
                     "drug2",
                     "concdrug2.micro_M.",
                     "GeneID",
                     "identifier",
                     "color")

  invisible(melt(rbind(df, tmp)[,columnsToKeep],
            measure.vars=normCols))
}
```

To test for drug synergy we use a one-sided t-test to test if the inhibition of the drug combination is stronger than the expected inhibition. This is done independently for each concentration of a drug combination.

```
testSynergy <- function(df, drugNames, summarizeWells=TRUE){

  if(summarizeWells){
    ## summarize wells on the same plate
    meanValue <- tapply(df$value, list(df$GeneID, df$variable, df$plate),
                        mean, na.rm=TRUE)
    for(i in dimnames(meanValue)[[1]]){
      for(j in dimnames(meanValue)[[2]]){
        for(k in dimnames(meanValue)[[3]]){
          selection <- df$GeneID == i &
            df$variable == j & df$plate == k
          if(sum(selection) > 0)
```

```
          df$meanValue[selection] = meanValue[i, j, k]
        }
      }
    }
    df$value = NULL
    df = unique(df)
    colnames(df) <- gsub("meanValue", "value", colnames(df))
  }

  ## test synergy for each drug concentration
  for(conc in unique(df$concdrug1.micro_M.)){
    if(conc==0) next
    dfConc = df[df$concdrug1.micro_M. == conc, ]
    test = t.test(dfConc$value[dfConc$identifier == paste(drugNames[1],
                                                          drugNames[2],
                                                          sep="_")],
                  dfConc$value[dfConc$identifier == "expected"],
                  alternative="less")
    df$pvalue[df$concdrug1.micro_M. == conc] = test$p.value
    df$combi_mean[df$concdrug1.micro_M. == conc] = test$estimate[1]
    df$expected_mean[df$concdrug1.micro_M. == conc] = test$estimate[2]
  }
  ## set pvalue to 1 for 0 drug concentrations
  df$pvalue[is.na(df$pvalue)] = 1
  invisible(df)
}
```

The values of individual replicates are summarized using the mean value and the standard error of the mean. This data is used to visualize the results.

```
getSummary <- function(df){
  ## calculate mean values and sem
  ids = paste(df$concdrug1.micro_M., df$identifier)

  sem <- function(x, ...) sd(x, ...)/sqrt(sum(!is.na((x))))

  summary = data.frame(concdrug1.micro_M. =
                         as.vector(tapply(df$concdrug1.micro_M.,
                                          ids,
                                          unique)),
                       identifier = as.vector(tapply(df$identifier,
                                                     ids,
                                                     unique)),
                       color = as.vector(tapply(df$color,
                                                ids,
                                                unique)),
                       pvalue = as.vector(tapply(df$pvalue,
                                                 ids,
                                                 unique)),
                       mean = as.vector(tapply(df$value,
                                               ids,
                                               mean,
                                               na.rm=TRUE)),
                       sem = as.vector(tapply(df$value,
                                              ids,
                                              sem,
                                              na.rm=TRUE)),
                       n = as.vector(tapply(!is.na(df$value),
```

```
                                                ids,
                                                sum)),
                        stringsAsFactors=FALSE)

  summary$identifier = as.factor(summary$identifier)
  levels(summary$identifier) = levels(df$identifier)
  summary$color = as.factor(summary$color)
  levels(summary$color) = levels(df$color)
  invisible(summary)
}
```

The function `analyseDrugCombi` calculates the expected values and sumarizes the results across replicates the selected drug combination.

```
analyseDrugCombi <- function(df, combi, plotSingle=TRUE, type="nonInteraction"){

  drugNames = unlist(strsplit(combi, "_"))

  ## select all wells beloning to the combination
  df_sub = subset(df,
                 grepl(paste(c(paste(drugNames, collapse="_"),
                               paste0(drugNames, "_DMSO")),
                             collapse="|"),
                       df$identifier))

  ## head(df_sub)
  df_sub <- df_sub[order(df_sub$GeneID),]

  position1 <- which(df_sub$identifier == paste0(drugNames[1], "_DMSO") &
                      !grepl("DMSO_0$|^DMSO_0", df_sub$GeneID))
  position2 <- which(df_sub$identifier == paste0(drugNames[2], "_DMSO") &
                      !grepl("DMSO_0$|^DMSO_0", df_sub$GeneID))

  if(!identical(gsub(drugNames[1], "", df_sub$GeneID[position1]),
                gsub(drugNames[2], "", df_sub$GeneID[position2])))
    stop("single drug values do not match")

  ## add color information for plots
  df_sub$color = "#5CBA48"
  df_sub$color[position1] = "gray60"
  df_sub$color[position2] = "gray30"

  tmp <- getExpectedValue(df_sub,
                          position1,
                          position2,
                          drugNames,
                          type=type)

  ## manually set colors and order for plots
  labelOrder = c(paste0(drugNames, "_DMSO"),
                 "expected",
                 paste(drugNames, collapse="_"))
  tmp$identifier = factor(tmp$identifier, levels = labelOrder)
  colorForPlot = c("gray60", "gray30", "white", "#5CBA48")
  tmp$color = factor(tmp$color, levels= colorForPlot)

  tmp <- testSynergy(tmp, drugNames)
  if(plotSingle) PGPC:::plotSingleValues(tmp, pthresh=0.05)
```

```
  invisible(getSummary(tmp))
}
```

11.2.2 Investigating drug synergies

The data for the HCT cell lines and the drug combinations presented in the paper is shown in this section. For simplicity we only show the results for one drug combination. The other combinations can be visualized by uncommenting the other drug combinations.

We first load the data from the cellHTS2 report.

```
experiment = "2013-10-22platelist_HCT_72_NPI_none"

## get the dataframe and check the data in an explorative manner
df = read.delim(file.path("result", experiment, "in", "topTable.txt"),
                stringsAsFactors=FALSE)
df$identifier = paste(df$drug1, df$drug2, sep="_")
df$concdrug1.micro_M. = round(df$concdrug1.micro_M., 4)

## remove problematic replicate 4
df <- df[, -grep("r4", names(df))]


theme_new = theme_bw()
theme_new$text$size = 20
theme_new$axis.text$size = rel(1.2)
```

First we look at the drug interactions for Bendamustin and Akt inhibitors using the non-interacting model.

We plot the distribution of the NPI values for single measurements, a line plot and barplot of mean and sem values, and finally the mean inhibition and its sem as bar plot.

```
drugCombis = c("Bendamustine_AKTi VIII")
##drugCombis = c("Bendamustine_AKTi VIII", "Bendamustine_PD", "Bendamustine_U0126",
##                "Bendamustine_MK2206")

## for better overview we plot only a selection of concentrations
concentrations = c("1.25", "2.5", "5", "10")
breaks = c(1,3,10)
limits = c(1, 11)

for (combi in drugCombis){
  summary <- analyseDrugCombi(df, combi, plotSingle=TRUE)

  PGPC:::plotSummary(summary,
                          concentration=concentrations,
                          pthresh=0.05,
                          breaks=breaks,
                          limits=limits)

  PGPC:::plotSummaryBarplot(summary,
                                 concentration=concentrations,
                                 pthresh=0.05)

  PGPC:::plotSummaryBarplotInhibition(summary,
                                           concentration=concentrations,
                                           pthresh=0.05)
}
```

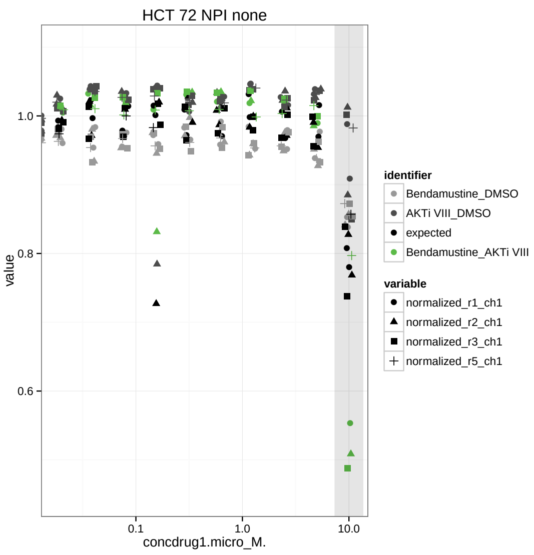

(a) Normalized viability values for all experiments.

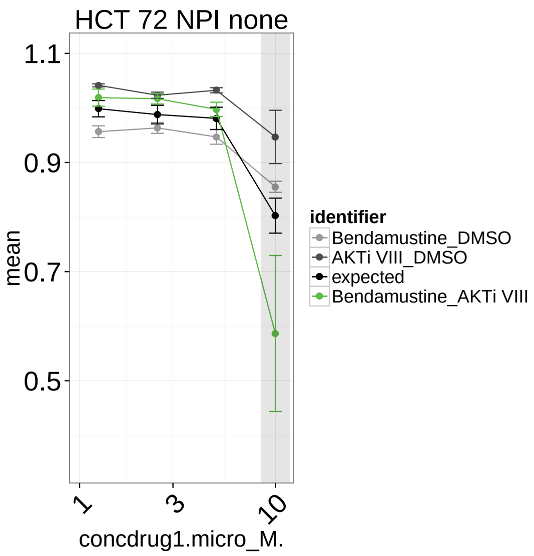

(b) Line plot of sumarized viability values for selected concentrations.

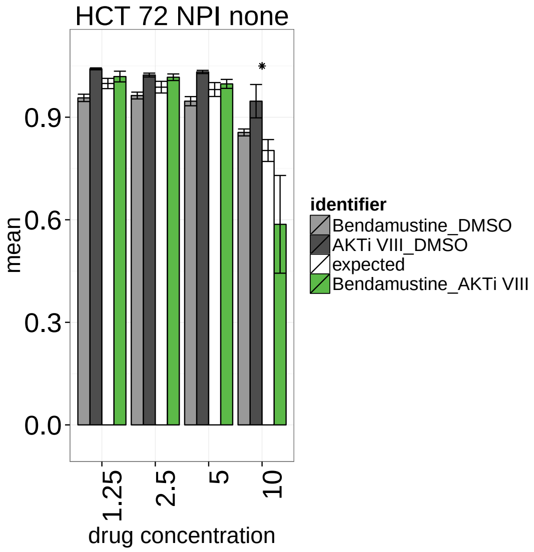

(c) Barplot of sumarized viability values for selected concentrations.

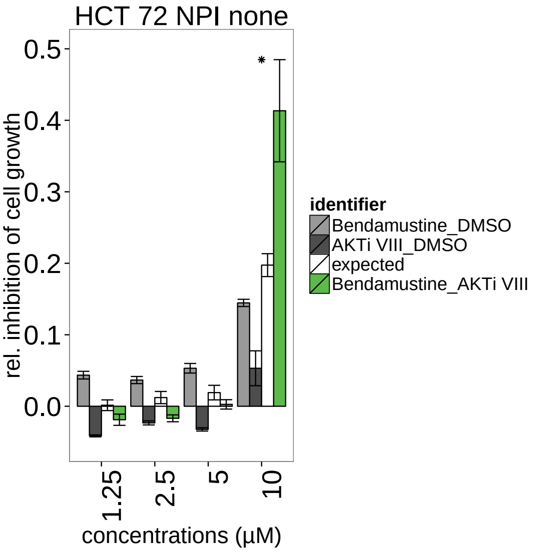

(d) Barplot of drug inhibition for selected concentrations. This figure is the basis of Figure 4B in the paper.

Figure 34: Drug synergies for Bendamustine and AKT inhibitors calculated using an non-interacting model in the HCT116 parental cell line. Error bars, means +- s.e.m

Here we show the results using the HSA model for the same drug combination.

```
for (combi in drugCombis){
  summary <- analyseDrugCombi(df, combi, plotSingle=TRUE, type="HSA")

  PGPC:::plotSummary(summary,
                       concentration=concentrations,
                       pthresh=0.05,
                       breaks=breaks,
                       limits=limits)

  PGPC:::plotSummaryBarplot(summary,
                              concentration=concentrations,
                              pthresh=0.05)
```

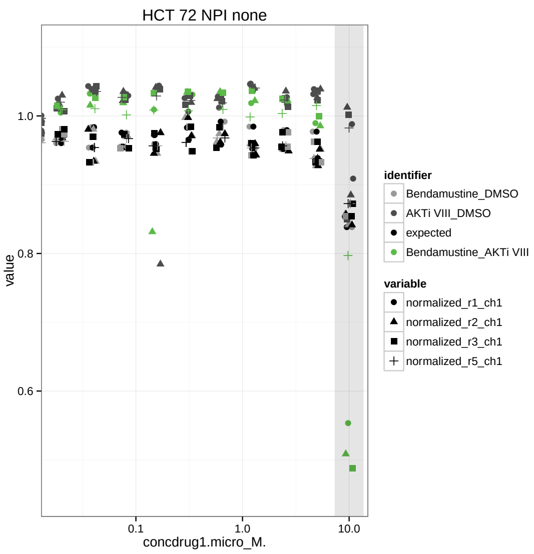

(a) Normalized viability values for all experiments.

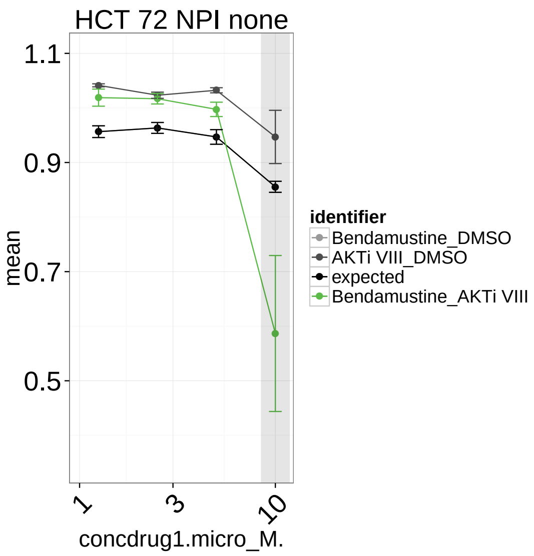

(b) Line plot of sumarized viability values for selected concentrations.

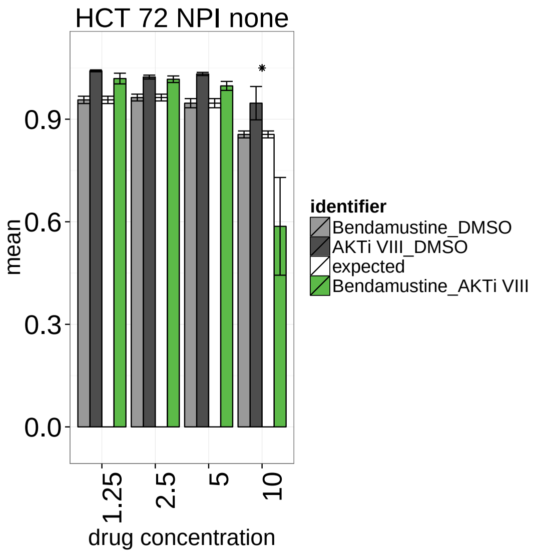

(c) Barplot of sumarized viability values for selected concentrations.

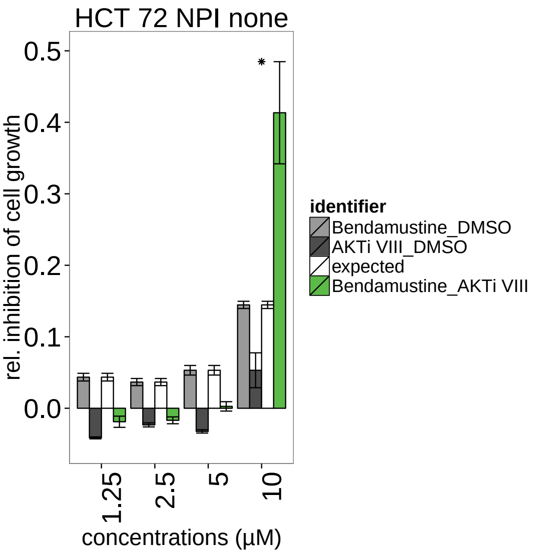

(d) Barplot of drug inhibition for selected concentrations.

Figure 35: Drug synergies for Bendamustine and AKT inhibitors calculated using the HSA model in the HCT116 parental cell line. Error bars, means +- s.e.m

```
  PGPC:::plotSummaryBarplotInhibition(summary,
                                       concentration=concentrations,
                                       pthresh=0.05)
}
```

Next we look at the drug interactions for Disulfiram and MEK inhibitors. First, we use the non-interacting model for estimating drug synergy.

```
drugCombis = c("Disulfiram_PD")
#drugCombis = c("Disulfiram_PD", "Disulfiram_U0126")

## for better overview plot only a selection of concentrations
concentrations = c("0.0195", "0.0391","0.0781","0.1562")
```

```
breaks = c(0.01, 0.03, 0.1, 0.3)
limits=c (0.01, 0.5)

for (combi in drugCombis){
  summary <- analyseDrugCombi(df, combi, plotSingle=TRUE)

  PGPC:::plotSummary(summary,
                             concentration=concentrations,
                             pthresh=0.05,
                             breaks=breaks,
                             limits=limits)

  PGPC:::plotSummaryBarplot(summary,
                                    concentration=concentrations,
                                    pthresh=0.05)

  PGPC:::plotSummaryBarplotInhibition(summary,
                                              concentration=concentrations,
                                              pthresh=0.05)
}
```

And second, we use the HSA model for estimating drug synergy.

```
for (combi in drugCombis){
  summary <- analyseDrugCombi(df, combi, plotSingle=TRUE, type="HSA")

  PGPC:::plotSummary(summary,
                             concentration=concentrations,
                             pthresh=0.05,
                             breaks=breaks,
                             limits=limits)

  PGPC:::plotSummaryBarplot(summary,
                                    concentration=concentrations,
                                    pthresh=0.05)

  PGPC:::plotSummaryBarplotInhibition(summary,
                                              concentration=concentrations,
                                              pthresh=0.05)
}
```

11.2.3 Investigating drug synergies, DLD cell line

The data for the DLD cell lines and the drug combinations presented in supplemental Figures is shown in this section. It is analogous to the analysis of the HCT116 celline in the previous section.

We first load the data from the cellHTS2 report.

```
experiment = "2013-10-22platelist_DLD_72_NPI_none"

## get the dataframe and check the data in an explorative manner
df = read.delim(file.path("result", experiment, "in", "topTable.txt"),
                stringsAsFactors=FALSE)
df$identifier = paste(df$drug1, df$drug2, sep="_")
df$concdrug1.micro_M. = round(df$concdrug1.micro_M., 4)

theme_new = theme_bw()
theme_new$text$size = 20
```

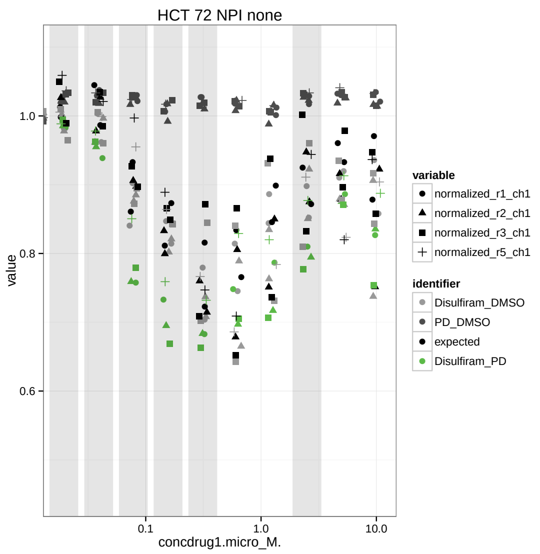

(a) Normalized viability values for all experiments.

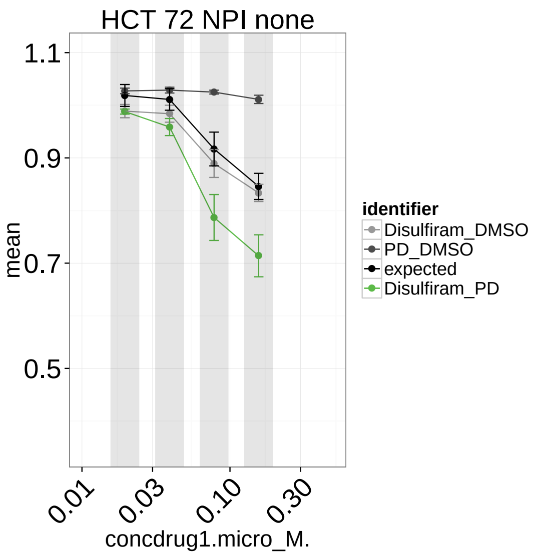

(b) Line plot of sumarized viability values for selected concentrations.

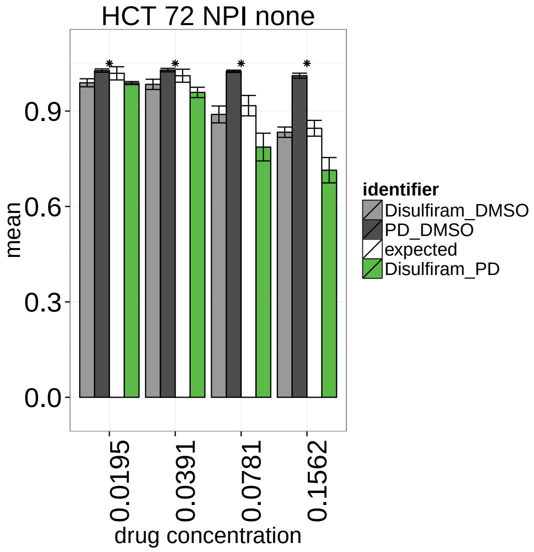

(c) Barplot of sumarized viability values for selected concentrations.

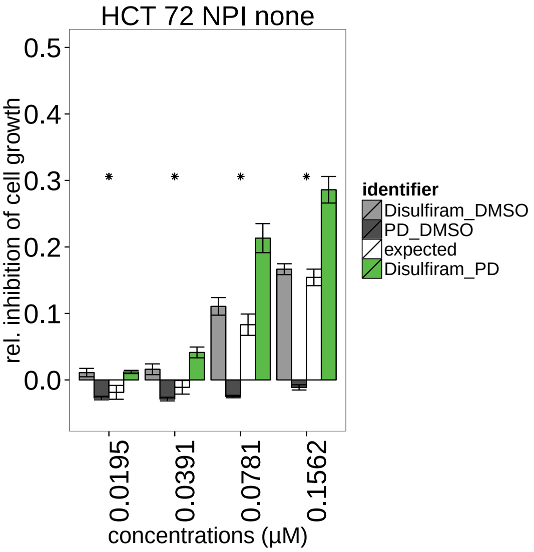

(d) Barplot of drug inhibition for selected concentrations. This figure is the basis of Figure 4D in the paper.

Figure 36: Drug synergies for Disulfiram and MEK inhibitors calculated using an non-interacting model in the HCT116 parental cell line. Error bars, means +- s.e.m

```
theme_new$axis.text$size = rel(1.2)
```

First we look at the drug interactions for Bendamustin and Akt inhibitors using the non-interacting model.

```
drugCombis = c("Bendamustine_AKTi VIII")
## drugCombis = c("Bendamustine_AKTi VIII", "Bendamustine_PD", "Bendamustine_U0126",
##                "Bendamustine_MK2206")


## for better overview plot only a selection of concentrations
concentrations = c("1.25", "2.5", "5", "10")
breaks = c(1,3,10)
limits = c(1, 11)
```

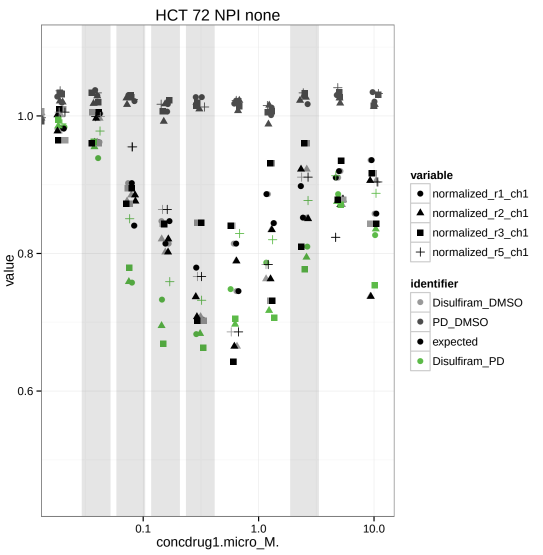

(a) Normalized viability values for all experiments.

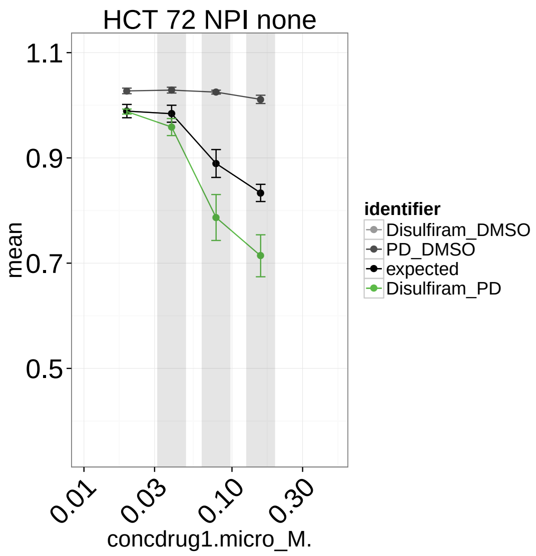

(b) Line plot of sumarized viability values for selected concentrations.

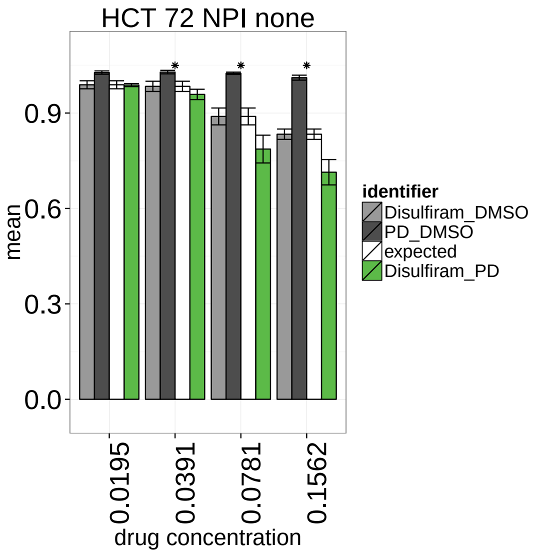

(c) Barplot of sumarized viability values for selected concentrations.

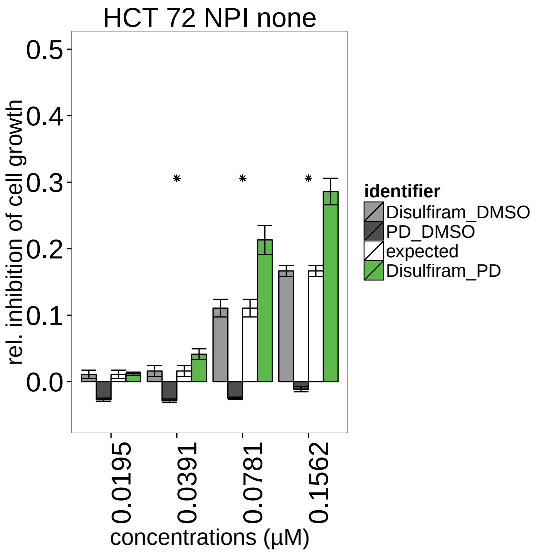

(d) Barplot of drug inhibition for selected concentrations.

Figure 37: Drug synergies for Disulfiram and MEK inhibitors calculated using an non-interacting model in the HCT116 parental cell line. Error bars, means +- s.e.m

```
for (combi in drugCombis){
  summary <- analyseDrugCombi(df, combi, plotSingle=TRUE)

  PGPC:::plotSummary(summary,
                     concentration=concentrations,
                     pthresh=0.05,
                     breaks=breaks,
                     limits=limits)

  PGPC:::plotSummaryBarplot(summary,
                            concentration=concentrations,
                            pthresh=0.05)
```

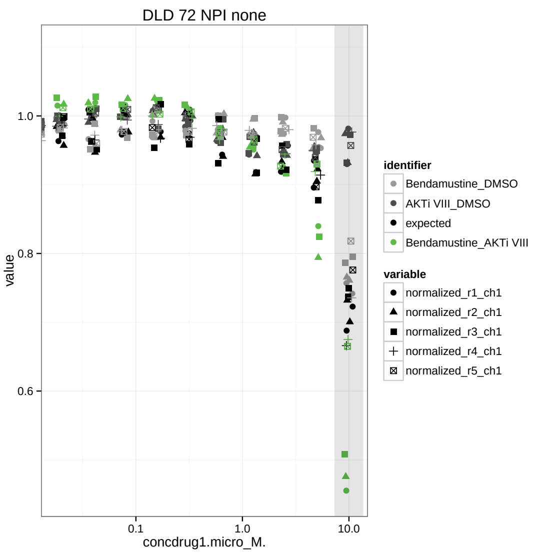

(a) Normalized viability values for all experiments.

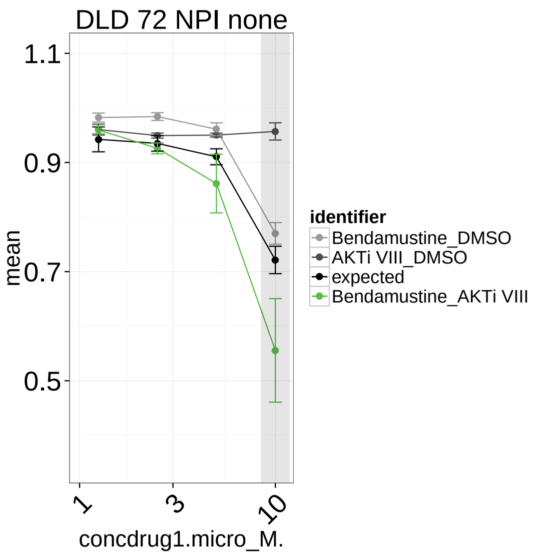

(b) Line plot of sumarized viability values for selected concentrations.

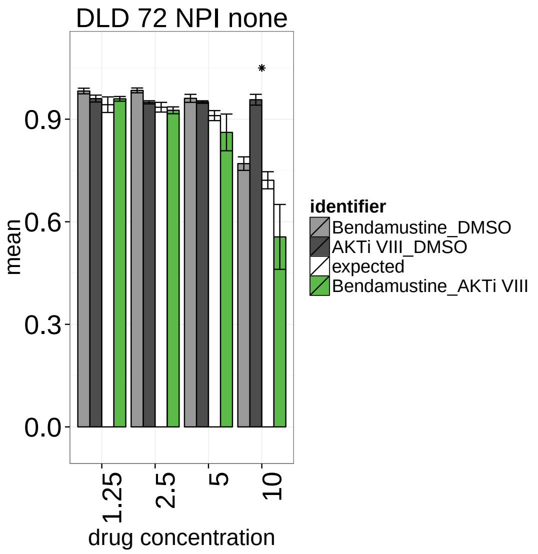

(c) Barplot of sumarized viability values for selected concentrations.

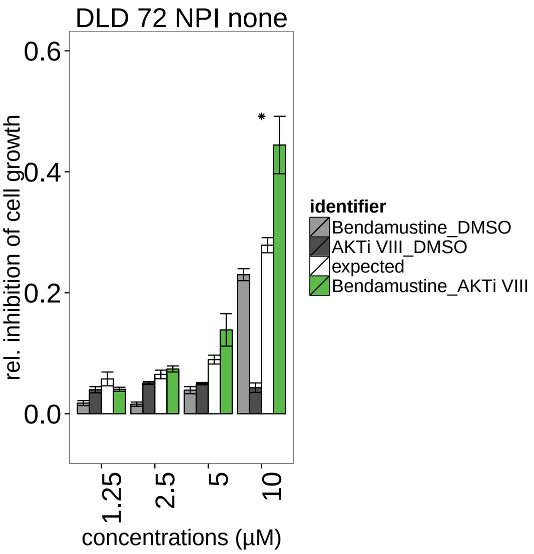

(d) Barplot of drug inhibition for selected concentrations. This figure is the basis of Expanded view Figure EV3C in the paper.

Figure 38: Drug synergies for Bendamustine and AKT inhibitors calculated using an non-interacting model in the DLD-1 cell line. Error bars, means +- s.e.m

```
  PGPC:::plotSummaryBarplotInhibition(summary,
                                      concentration=concentrations,
                                      pthresh=0.05,
                                      ylimits=c(-0.05,.6))
}
```

And next using the HSA model for Bendamustin and the Akt inhibitors.

```
for (combi in drugCombis){
  summary <- analyseDrugCombi(df, combi, plotSingle=TRUE, type="HSA")

  PGPC:::plotSummary(summary,
```

```
                            concentration=concentrations,
                            pthresh=0.05,
                            breaks=breaks,
                            limits=limits)

  PGPC:::plotSummaryBarplot(summary,
                                   concentration=concentrations,
                                   pthresh=0.05)

  PGPC:::plotSummaryBarplotInhibition(summary,
                                             concentration=concentrations,
                                             pthresh=0.05,
                                             ylimits=c(-0.05,.6))
}
```

Next we look at the drug interactions for Disulfiram and MEK inhibitors using the non-interacting model.

```
drugCombis = c("Disulfiram_PD")
##drugCombis = c("Disulfiram_PD", "Disulfiram_U0126")



## for better overview plot only a selection of concentrations
concentrations = c("0.0195", "0.0391","0.0781","0.1562")
breaks = c(0.01, 0.03, 0.1, 0.3)
limits=c (0.01, 0.5)

for (combi in drugCombis){
  summary <- analyseDrugCombi(df, combi, plotSingle=TRUE)

  PGPC:::plotSummary(summary,
                            concentration=concentrations,
                            pthresh=0.05,
                            breaks=breaks,
                            limits=limits)

  PGPC:::plotSummaryBarplot(summary,
                                   concentration=concentrations,
                                   pthresh=0.05)

  PGPC:::plotSummaryBarplotInhibition(summary,
                                             concentration=concentrations,
                                             pthresh=0.05)
}
```

And finally we use the HSA model for Disulfiram and the MEK inhibitors.

```
for (combi in drugCombis){
  summary <- analyseDrugCombi(df, combi, plotSingle=TRUE, type="HSA")

  PGPC:::plotSummary(summary,
                            concentration=concentrations,
                            pthresh=0.05,
                            breaks=breaks,
                            limits=limits)

  PGPC:::plotSummaryBarplot(summary,
                                   concentration=concentrations,
                                   pthresh=0.05)
```

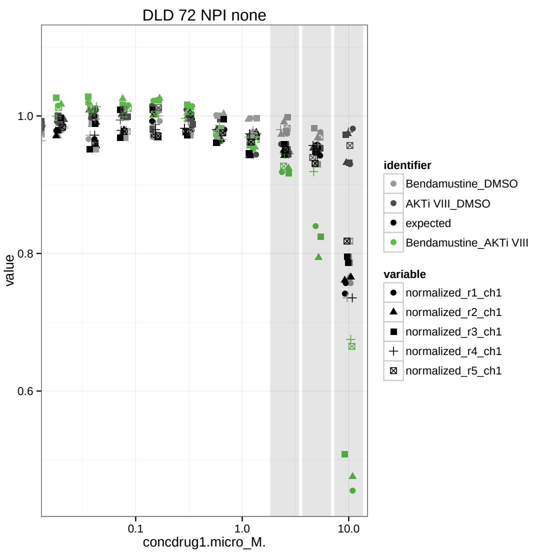

(a) Normalized viability values for all experiments.

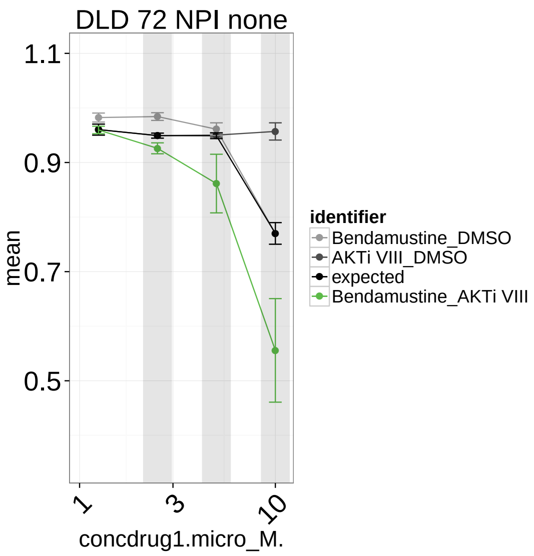

(b) Line plot of sumarized viability values for selected concentrations.

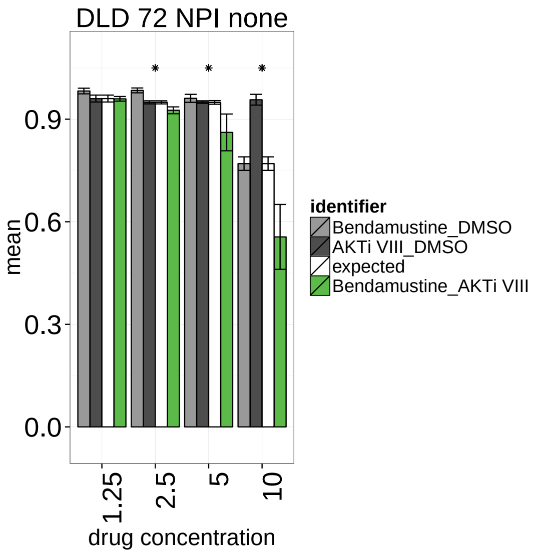

(c) Barplot of sumarized viability values for selected concentrations.

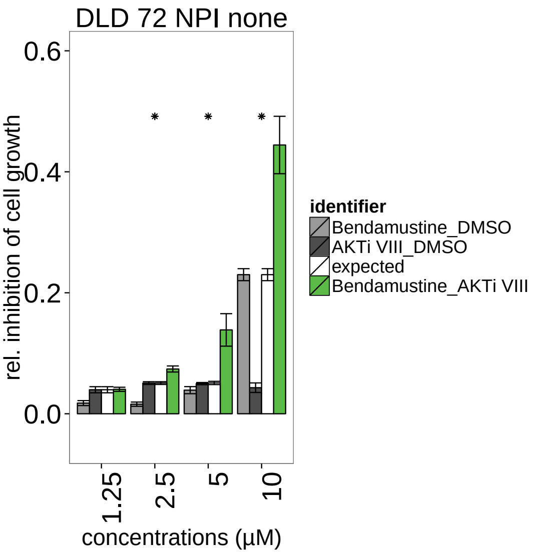

(d) Barplot of drug inhibition for selected concentrations.

Figure 39: Drug synergies for Bendamustine and AKT inhibitors calculated using the HSA model in the DLD-1 cell line. Error bars, means +- s.e.m

```
  PGPC:::plotSummaryBarplotInhibition(summary,
                                      concentration=concentrations,
                                      pthresh=0.05)
}
```

12 Follow-up: Proteasome inhibition assay

Here we analyse the effect of drugs on the proteasome activity. The CellTiter-Glo assay is used to normalize for the viability effects of the drugs. DMSO is used as control for 100 % proteasome activity.

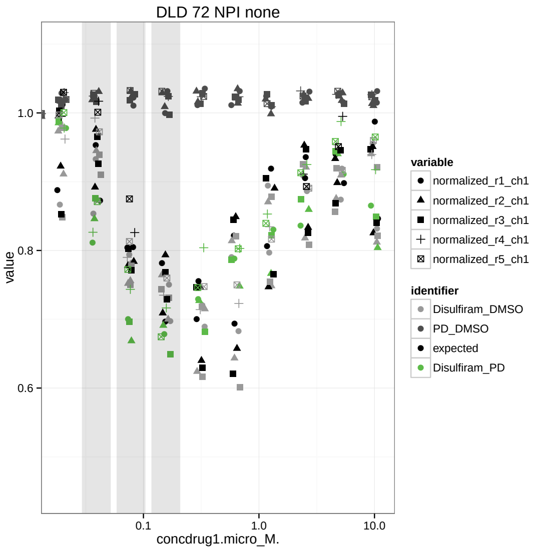

(a) Normalized viability values for all experiments.

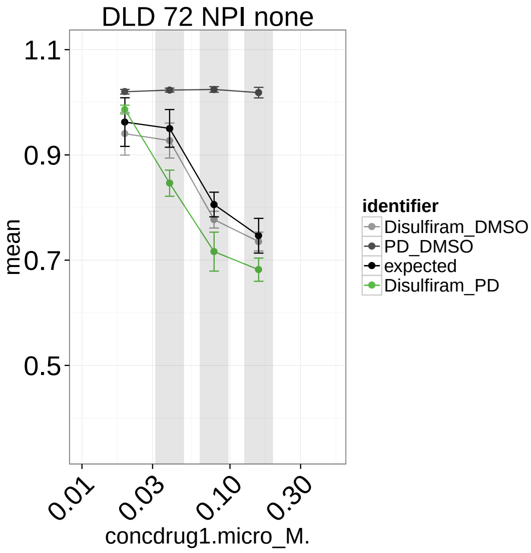

(b) Line plot of sumarized viability values for selected concentrations.

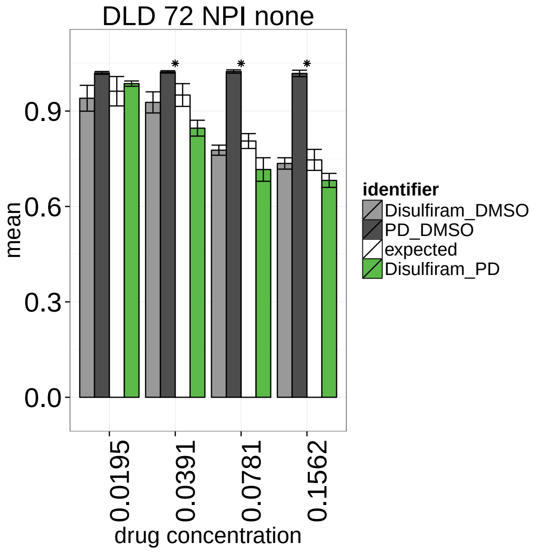

(c) Barplot of sumarized viability values for selected concentrations.

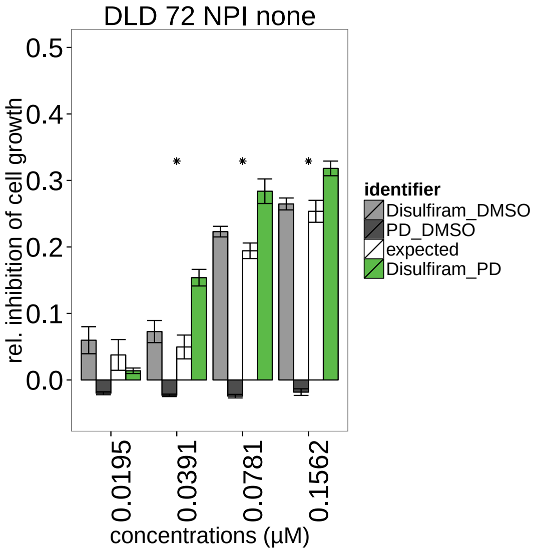

(d) Barplot of drug inhibition for selected concentrations. This figure is the basis of Expanded View Figure EV3H in the paper.

Figure 40: Drug synergies for Disulfiram and MEK inhibitors calculated using an non-interacting model in the DLD-1 cell line. Error bars, means +- s.e.m

12.0.4 Data processing and quality control

First we read the data from the different plates.

```
readData <- function(path, files, log10Transform=TRUE){
  do.call(rbind, lapply(files, function(file){
    tmp <- read.delim(file.path(path, file))
    tmp$plate = match(file, files)
    tmp$plateName = file
    if(log10Transform) tmp$log10Value = log10(tmp$value)
    tmp
  }))
}
```

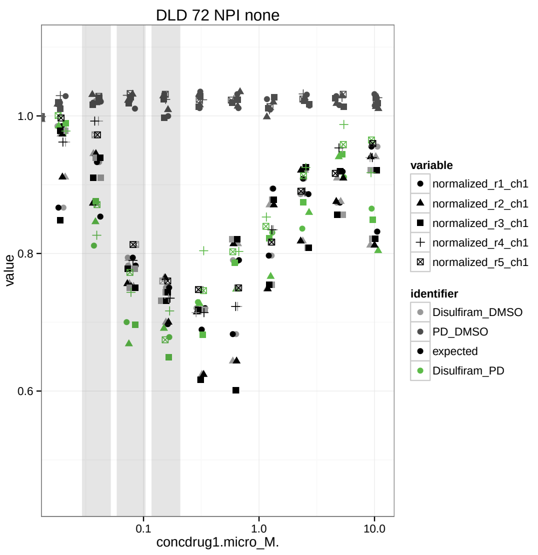

(a) Normalized viability values for all experiments.

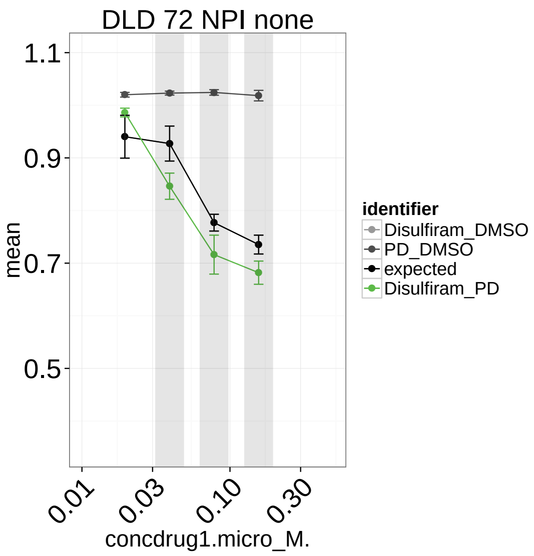

(b) Line plot of sumarized viability values for selected concentrations.

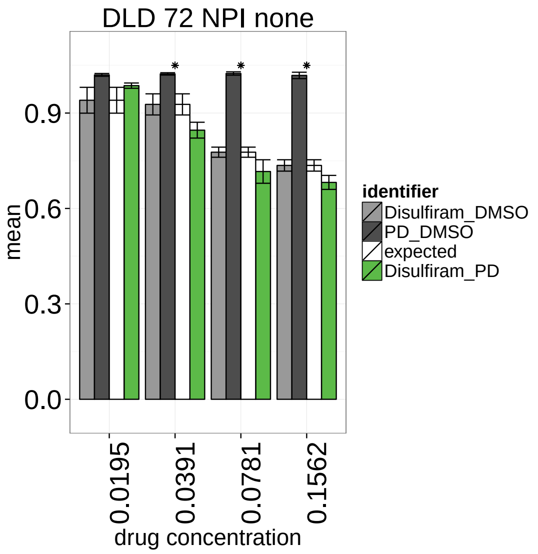

(c) Barplot of sumarized viability values for selected concentrations.

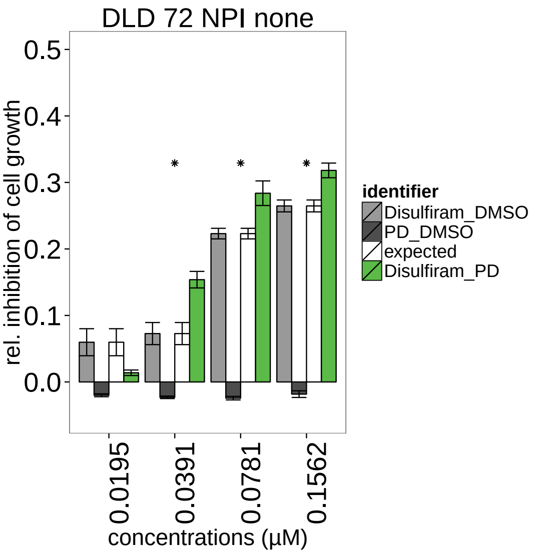

(d) Barplot of drug inhibition for selected concentrations.

Figure 41: Drug synergies for Disulfiram and MEK inhibitors calculated using an non-interacting model in the DLD-1 cell line. Error bars, means +- s.e.m

```
path=system.file("extdata", "Proteasome_assays_follow-up",
                 package="PGPC")
files = list.files(path)

data = readData(path,files)
head(data)

##    value       drug assay plate                         plateName log10Value
## 1 198550       DMSO  CASP     1 2014-01-30New_protGlo_24h_v01.txt   5.297870
## 2 186220       DMSO  CASP     1 2014-01-30New_protGlo_24h_v01.txt   5.270026
## 3 203560 Disulfiram  CASP     1 2014-01-30New_protGlo_24h_v01.txt   5.308692
## 4 186180 Disulfiram  CASP     1 2014-01-30New_protGlo_24h_v01.txt   5.269933
## 5 199990       ZPCK  CASP     1 2014-01-30New_protGlo_24h_v01.txt   5.301008
```

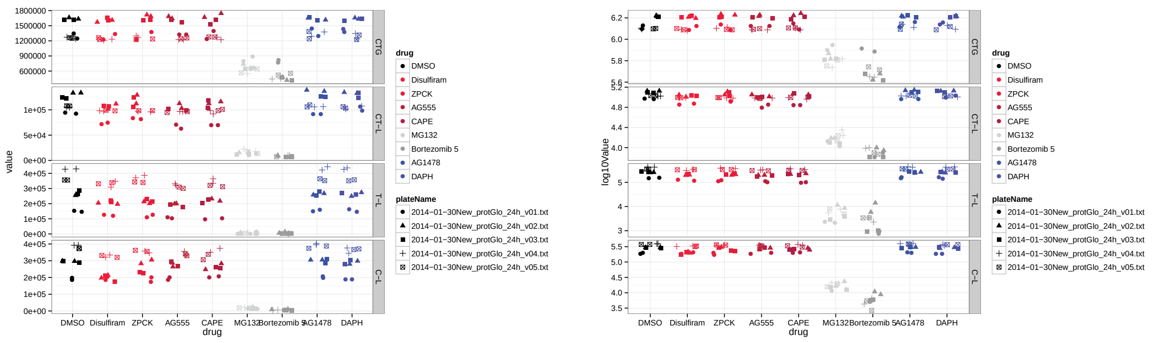

(a) Untransformed values.

(b) log10 transformed values.

Figure 42: Raw plate reader values for the different drugs and assays. CellTiterGlo (CTG), chymotrypsin-like (CT-L), trypsin-like (T-L), and caspase-like (C-L) activity assay.

```
## 6 174820        ZPCK   CASP     1 2014-01-30New_protGlo_24h_v01.txt   5.242591

## remove BTO-1 drug
data <- subset(data, drug != "BTO-1")

data$assay = factor(data$assay,
                    levels=c("CTG", "Chym","Tryp", "CASP"),
                    ordered=TRUE)
levels(data$assay) = c("CTG", "CT-L","T-L", "C-L")
data$drug = factor(data$drug,
                   levels=c("DMSO", "Disulfiram", "ZPCK", "AG555", "CAPE",
                            "MG132","Bortezomib 5", "AG1478", "DAPH"),
                   ordered=TRUE)

cbpalette = c("black", "#ee1e39", "#e51d3a", "#bb1f3b",
              "#b21e42", "#d1d2d2", "#9b9b9b", "#3c53a4", "#4a52a3")

ggplot(data, aes(x=drug, y=value, color=drug, shape=plateName)) +
  geom_jitter(position = position_jitter(width = .3), size=2.5) +
  theme_bw() +
  scale_color_manual(values=cbpalette) +
  facet_grid(assay ~ ., scales="free_y")


ggplot(data, aes(x=drug, y=log10Value, color=drug, shape=plateName)) +
  geom_jitter(position = position_jitter(width = .3), size=2.5) +
  theme_bw() +
  scale_color_manual(values=cbpalette) +
  facet_grid(assay ~ ., scales="free_y")
```

We alculate the mean values per plate for the two wells of each drug assay combination.

```
mergeWells <- function(data){
  for(plate in unique(data$plate)){
    pp = data$plate == plate
    for(drug in unique(data$drug)){
      ii = data$drug == drug
      for (assay in unique(data$assay)){
        jj = data$assay == assay
        data$mean[pp & ii & jj] = mean(data$value[pp & ii & jj])
      }
    }
```

```
  }
  data$log10Value = log10(data$mean)
  data$value=NULL
  names(data) <- gsub("mean", "value", names(data))
  unique(data)
}

data = mergeWells(data)
```

12.0.5 Viability normalization

The data is normalized to the viability control "CTG" assay wells on each plate.

```
normalizeViability <- function(data){
  for(plate in unique(data$plate)){
    pp = data$plate == plate
    for(drug in unique(data$drug)){
      ii = data$drug == drug
      jj = data$assay == "CTG"

      data$normalized[ii & pp] = data$value[ii & pp] / data$value[ii & jj & pp]

      data$logNormalized[ii & pp] =
        data$log10Value[ii & pp] - data$log10Value[ii & jj & pp]
    }
  }
  data
}

data = normalizeViability(data)
head(data)

##              drug assay plate                         plateName log10Value  value
## 1            DMSO   C-L     1 2014-01-30New_protGlo_24h_v01.txt   5.284171 192385
## 3      Disulfiram   C-L     1 2014-01-30New_protGlo_24h_v01.txt   5.289745 194870
## 5            ZPCK   C-L     1 2014-01-30New_protGlo_24h_v01.txt   5.272781 187405
## 7           AG555   C-L     1 2014-01-30New_protGlo_24h_v01.txt   5.286131 193255
## 9            CAPE   C-L     1 2014-01-30New_protGlo_24h_v01.txt   5.307838 203160
## 11 Bortezomib 5   C-L     1 2014-01-30New_protGlo_24h_v01.txt   3.710540   5135
##      normalized logNormalized
## 1  0.148551242    -0.8281237
## 3  0.152451809    -0.8168674
## 5  0.144055191    -0.8414711
## 7  0.145595703    -0.8368514
## 9  0.153899764    -0.8127620
## 11 0.006470922    -2.1890338

# Error bars represent standard error of the mean
ggplot(data, aes(x=drug, y=normalized, color=drug, shape=plateName)) +
  geom_point(position=position_jitter(height=0), size=2.5) +
  theme_bw() +
  scale_color_manual(values=cbpalette) +
  facet_grid(assay ~ ., scales="free_y")


ggplot(data, aes(x=drug, y=logNormalized, color=drug, shape=plateName)) +
  geom_point(position=position_jitter(height=0), size=2.5) +
  theme_bw() +
```

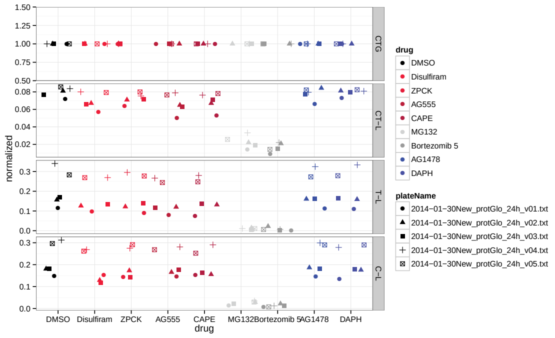

(a) Untransformed values.

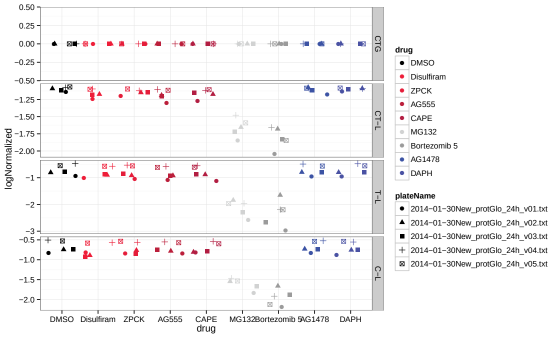

(b) log10 transformed values.

Figure 43: Viability normalized values for the different drugs and assays. CellTiterGlo (CTG), chymotrypsin-like (CT-L), trypsin-like (T-L), and caspase-like (C-L) activity assay.

```
  scale_color_manual(values=cbpalette) +
  facet_grid(assay ~ ., scales="free_y")
```

12.0.6 Proteasome activity normalization

The proteasome activity is measured compared to the DMSO controls of the corresponding wells on each plate. For the untransformed values DMSO is set to 1 and for the log10 transformed values DMSO is set to 0 for each assay.

```
normalizeAssay <- function(data){
  for(plate in unique(data$plate)){
    pp = data$plate == plate
    for(assay in unique(data$assay)){
      ii = data$assay == assay
      jj = data$drug == "DMSO"

      data$normalizedActivity[ii & pp] =
        data$normalized[ii & pp] / data$normalized[ii & jj & pp]

      data$logNormalizedActivity[ii & pp] =
        data$logNormalized[ii & pp] - data$logNormalized[ii & jj & pp]
    }
  }
  data
}

data = normalizeAssay(data)
head(data)

##              drug assay plate                         plateName log10Value  value
## 1            DMSO   C-L     1 2014-01-30New_protGlo_24h_v01.txt   5.284171 192385
## 3      Disulfiram   C-L     1 2014-01-30New_protGlo_24h_v01.txt   5.289745 194870
## 5            ZPCK   C-L     1 2014-01-30New_protGlo_24h_v01.txt   5.272781 187405
## 7           AG555   C-L     1 2014-01-30New_protGlo_24h_v01.txt   5.286131 193255
## 9            CAPE   C-L     1 2014-01-30New_protGlo_24h_v01.txt   5.307838 203160
## 11 Bortezomib 5   C-L     1 2014-01-30New_protGlo_24h_v01.txt   3.710540   5135
##     normalized logNormalized normalizedActivity logNormalizedActivity
## 1  0.148551242    -0.8281237          1.0000000           0.000000000
## 3  0.152451809    -0.8168674          1.0262574           0.011256294
## 5  0.144055191    -0.8414711          0.9697340          -0.013347374
## 7  0.145595703    -0.8368514          0.9801042          -0.008727731
## 9  0.153899764    -0.8127620          1.0360046           0.015361665
```

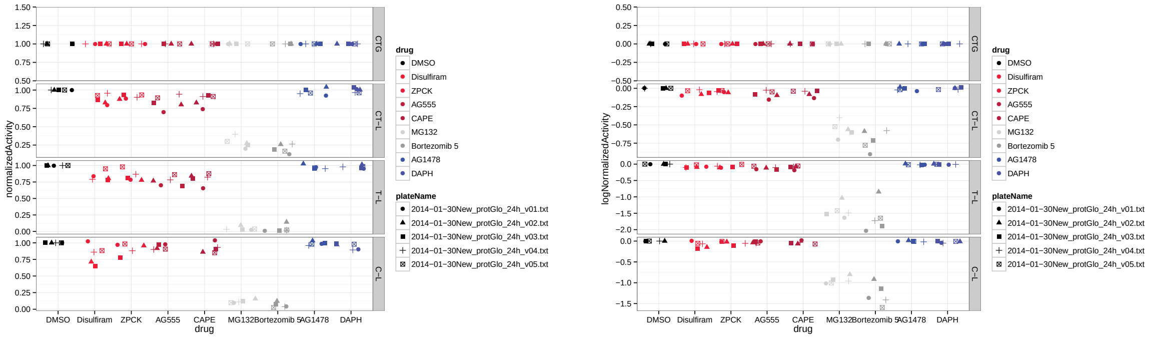

(a) Untransformed values. (b) log10 transformed values.

Figure 44: Viability and proteasome activity normalized values for the different drugs and assays. CellTiterGlo (CTG), chymotrypsin-like (CT-L), trypsin-like (T-L), and caspase-like (C-L) activity assay.

```
## 11 0.006470922    -2.1890338          0.0435602          -1.360910136

# Error bars represent standard error of the mean
ggplot(data, aes(x=drug, y=normalizedActivity, color=drug, shape=plateName)) +
  geom_point(position=position_jitter(height=0), size=2.5) +
  theme_bw() +
  scale_color_manual(values=cbpalette) +
  facet_grid(assay ~ ., scales="free_y")


ggplot(data, aes(x=drug,
                 y=logNormalizedActivity,
                 color=drug,
                 shape=plateName)) +
  geom_point(position=position_jitter(height=0), size=2.5) +
  theme_bw() +
  scale_color_manual(values=cbpalette) +
  facet_grid(assay ~ ., scales="free_y")
```

12.0.7 Result summary

The values are summarized using the values of all plate. Mean +- sem are plotted for each drug/assay combination. A two sided t-test is used to test whether the drugs have an effect on the proteasome compared to the fixed value of the DMSO control.

```
summarySE <- function(data=NULL, measurevar, groupvars=NULL, na.rm=FALSE,
                      conf.interval=.95, .drop=TRUE) {

  #  handle NA's: if na.rm==TRUE, don't count them
  length2 <- function (x, na.rm=FALSE) {
    if (na.rm) sum(!is.na(x))
    else       length(x)
  }

  #  summary. For each group's data frame, return a vector with
  # N, mean, and sd
  datac <- ddply(data, groupvars, .drop=.drop,
                 .fun = function(xx, col) {
                   tTest = try(t.test(xx[[col]] -
                                        ifelse(grepl("log", measurevar),
                                               0,
```

```
                                            1)),
                              silent=TRUE)
                   c(N    = length2(xx[[col]], na.rm=na.rm),
                     mean = mean   (xx[[col]], na.rm=na.rm),
                     sd   = sd     (xx[[col]], na.rm=na.rm),
                     p.value = ifelse(class(tTest) != "try-error",
                                      tTest$p.value,
                                      1)
                   )
                 },
                 measurevar
  )

  # Rename the "mean" column
  datac <- rename(datac, c("mean" = measurevar))

  datac$se <- datac$sd / sqrt(datac$N)  # Calculate standard error of the mean

  # Confidence interval multiplier for standard error
  # Calculate t-statistic for confidence interval:
  # e.g., if conf.interval is .95, use .975 (above/below), and use df=N-1
  ciMult <- qt(conf.interval/2 + .5, datac$N-1)
  datac$ci <- datac$se * ciMult

  return(datac)
}

summary = summarySE(data,
                    measurevar="normalizedActivity",
                    groupvars=c("drug","assay"))

## remove ctg assay
summary <- subset(summary, assay!="CTG")

## remove DMSO control
summary <- subset(summary, drug!="DMSO")

pthresh = 0.05

p <- ggplot(summary,
            aes(x=assay,
                y=100*(1-normalizedActivity),
                ymin=100*(1-normalizedActivity-se),
                ymax=100*(1-normalizedActivity+se),
                fill=drug)) +
  geom_bar(position=position_dodge(), stat="identity", color="black") +
  geom_errorbar(width=.2,
                position=position_dodge(.9)) +
  theme_bw() + theme(panel.grid = element_blank()) +
  scale_fill_manual(values=cbpalette[-1]) +
  ylab("normalized proteasome inhibition (%)")
if(any(summary$p.value < pthresh))
  p = p + geom_point(aes(y=ifelse(p.value < pthresh,
                                  100*(1-normalizedActivity + se + 0.05),
                                  NA)),
                     position=position_dodge(.9),
                     pch=8,
                     col=1,
```

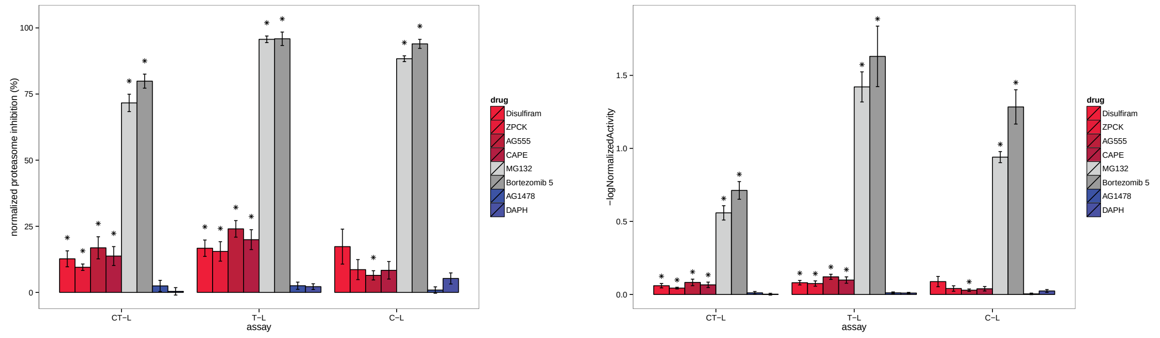

(a) Untransformed values. This figure is the basis of Figure 6B in the paper.

(b) log10 transformed values.

Figure 45: Summary of viability and proteasome activity normalized values for the different drugs and assays. CellTiterGlo (CTG), chymotrypsin-like (CT-L), trypsin-like (T-L), and caspase-like (C-L) activity assay. Error bars, means +- s.e.m.

```
                    show_guide = FALSE,
                    na.rm=TRUE)
print(p)


logSummary = summarySE(data,
                      measurevar="logNormalizedActivity",
                      groupvars=c("drug","assay"))

## remove ctg assay
logSummary <- subset(logSummary, assay!="CTG")

## remove DMSO control
logSummary <- subset(logSummary, drug!="DMSO")

p <- ggplot(logSummary,
            aes(x=assay, y=-logNormalizedActivity,
                ymin=-logNormalizedActivity-se,
                ymax=-logNormalizedActivity+se,
                fill=drug)) +
  geom_bar(position=position_dodge(), stat="identity", color="black") +
  geom_errorbar(width=.2,
                position=position_dodge(.9)) +
  theme_bw() + theme(panel.grid = element_blank()) +
  scale_fill_manual(values=cbpalette[-1])
if(any(summary$p.value < pthresh))
  p = p + geom_point(aes(y=ifelse(p.value < pthresh,
                                  -logNormalizedActivity+se+0.05,
                                  NA)),
                     position=position_dodge(width=.9),
                     pch=8,
                     col=1,
                     show_guide = FALSE,
                     na.rm=TRUE)
print(p)
```

13 Session Info

```
## R Under development (unstable) (2015-11-06 r69607)
## Platform: x86_64-pc-linux-gnu (64-bit)
## Running under: Ubuntu 14.04.3 LTS
##
## locale:
##  [1] LC_CTYPE=en_US.UTF-8       LC_NUMERIC=C               LC_TIME=en_US.UTF-8
##  [4] LC_COLLATE=C               LC_MONETARY=en_US.UTF-8    LC_MESSAGES=en_US.UTF-8
##  [7] LC_PAPER=en_US.UTF-8       LC_NAME=C                  LC_ADDRESS=C
## [10] LC_TELEPHONE=C             LC_MEASUREMENT=en_US.UTF-8 LC_IDENTIFICATION=C
##
## attached base packages:
##  [1] stats4    grid      parallel  stats     graphics  grDevices utils     datasets
##  [9] methods   base
##
## other attached packages:
##  [1] PGPC_0.99.5          plyr_1.8.3           reshape2_1.4.1       ChemmineR_2.23.0
##  [5] geneplotter_1.49.0   annotate_1.49.0      XML_3.98-1.3         AnnotationDbi_1.33.1
##  [9] IRanges_2.5.5        S4Vectors_0.9.7      lattice_0.20-33      ggplot2_1.0.1
## [13] gplots_2.17.0        limma_3.27.3         SearchTrees_0.5.2    imageHTS_1.21.0
## [17] cellHTS2_2.35.0      locfit_1.5-9.1       hwriter_1.3.2        vsn_3.39.0
## [21] splots_1.37.0        genefilter_1.53.0    Biobase_2.31.0       BiocGenerics_0.17.0
## [25] RColorBrewer_1.1-2   EBImage_4.13.0
##
## loaded via a namespace (and not attached):
##  [1] splines_3.3.0         gtools_3.5.0          affy_1.49.0
##  [4] highr_0.5.1           RBGL_1.47.0           tiff_0.1-5
##  [7] Category_2.37.0       robustbase_0.92-5     RSQLite_1.0.0
## [10] digest_0.6.8          colorspace_1.2-6      preprocessCore_1.33.0
## [13] Matrix_1.2-2          GSEABase_1.33.0       pcaPP_1.9-60
## [16] zlibbioc_1.17.0       xtable_1.8-0          fftwtools_0.9-7
## [19] mvtnorm_1.0-3         scales_0.3.0          gdata_2.17.0
## [22] jpeg_0.1-8            affyio_1.41.0         proto_0.3-10
## [25] survival_2.38-3       magrittr_1.5          evaluate_0.8
## [28] MASS_7.3-44           class_7.3-14          graph_1.49.1
## [31] BiocInstaller_1.21.1  tools_3.3.0           BiocStyle_1.9.2
## [34] formatR_1.2.1         stringr_1.0.0         munsell_0.4.2
## [37] cluster_2.0.3         e1071_1.6-7           caTools_1.17.1
## [40] RCurl_1.95-4.7        rjson_0.2.15          labeling_0.3
## [43] bitops_1.0-6          gtable_0.1.2          codetools_0.2-14
## [46] abind_1.4-3           DBI_0.3.1             rrcov_1.3-8
## [49] knitr_1.11            KernSmooth_2.23-15    prada_1.41.0
## [52] stringi_1.0-1         Rcpp_0.12.1           png_0.1-7
## [55] DEoptimR_1.0-4
```

References

[1] Grégoire Pau, Florian Fuchs, Oleg Sklyar, Michael Boutros, and Wolfgang Huber. EBImage — an R package for image processing with applications to cellular phenotypes. *Bioinformatics*, 26(7):979–981, 2010. doi:10.1093/bioinformatics/btq046.

[2] Anne E Carpenter, Thouis R Jones, Michael R Lamprecht, Colin Clarke, In Han Kang, Ola Friman, David a Guertin, Joo Han Chang, Robert a Lindquist, Jason Moffat, Polina Golland, and David M Sabatini. CellProfiler: image analysis software for identifying and quantifying cell phenotypes. *Genome Biology*, 7(10):R100,

January 2006. URL: http://www.pubmedcentral.nih.gov/articlerender.fcgi?artid=1794559&tool=pmcentrez&rendertype=abstract, doi:10.1186/gb-2006-7-10-r100.

[3] Thomas Horn, Thomas Sandmann, Bernd Fischer, Elin Axelsson, Wolfgang Huber, and Michael Boutros. Mapping of signaling networks through synthetic genetic interaction analysis by RNAi. *Nature Methods*, 8(4):341–6, May 2011. URL: http://www.ncbi.nlm.nih.gov/pubmed/21378980, doi:10.1038/nmeth.1581.

[4] Christina Laufer, Bernd Fischer, Maximilian Billmann, Wolfgang Huber, and Michael Boutros. Mapping genetic interactions in human cancer cells with RNAi and multiparametric phenotyping. *Nature Methods*, 10(5):427–31, May 2013. URL: http://www.ncbi.nlm.nih.gov/pubmed/23563794, doi:10.1038/nmeth.2436.

[5] Wolfgang Huber, Anja von Heydebreck, Holger Sültmann, Annemarie Poustka, and Martin Vingron. Variance stabilization applied to microarray data calibration and to the quantification of differential expression. *Bioinformatics*, 18 (Suppl.(1997), 2002.

[6] Ramamurthy Mani, Robert P St Onge, John L Hartman, Guri Giaever, and Frederick P Roth. Defining genetic interaction. *Proceedings of the National Academy of Sciences of the United States of America*, 105(9):3461–6, March 2008. URL: http://www.pubmedcentral.nih.gov/articlerender.fcgi?artid=2265146&tool=pmcentrez&rendertype=abstract, doi:10.1073/pnas.0712255105.

[7] Michael Costanzo, Anastasia Baryshnikova, Jeremy Bellay, Yungil Kim, Eric D Spear, Carolyn S Sevier, Huiming Ding, Judice L Y Koh, Kiana Toufighi, Sara Mostafavi, Jeany Prinz, Robert P St Onge, Benjamin VanderSluis, Taras Makhnevych, Franco J Vizeacoumar, Solmaz Alizadeh, Sondra Bahr, Renee L Brost, Yiqun Chen, Murat Cokol, Raamesh Deshpande, Zhijian Li, Zhen-Yuan Lin, Wendy Liang, Michaela Marback, Jadine Paw, Bryan-Joseph San Luis, Ermira Shuteriqi, Amy Hin Yan Tong, Nydia van Dyk, Iain M Wallace, Joseph a Whitney, Matthew T Weirauch, Guoqing Zhong, Hongwei Zhu, Walid a Houry, Michael Brudno, Sasan Ragibizadeh, Balázs Papp, Csaba Pál, Frederick P Roth, Guri Giaever, Corey Nislow, Olga G Troyanskaya, Howard Bussey, Gary D Bader, Anne-Claude Gingras, Quaid D Morris, Philip M Kim, Chris a Kaiser, Chad L Myers, Brenda J Andrews, and Charles Boone. The genetic landscape of a cell. *Science (New York, N.Y.)*, 327(5964):425–31, January 2010. URL: http://www.ncbi.nlm.nih.gov/pubmed/20093466, doi:10.1126/science.1180823.

[8] Paul Shannon, Andrew Markiel, Owen Ozier, Nitin S Baliga, Jonathan T Wang, Daniel Ramage, Nada Amin, Benno Schwikowski, and Trey Ideker. Cytoscape: a software environment for integrated models of biomolecular interaction networks. *Genome Research*, 13(11):2498–504, November 2003. URL: http://www.pubmedcentral.nih.gov/articlerender.fcgi?artid=403769&tool=pmcentrez&rendertype=abstract, doi:10.1101/gr.1239303.

[9] Xu Tan, Long Hu, Lovelace J Luquette, Geng Gao, Yifang Liu, Hongjing Qu, Ruibin Xi, Zhi John Lu, Peter J Park, and Stephen J Elledge. Systematic identification of synergistic drug pairs targeting HIV. *Nature Biotechnology*, 30(11):1125–30, November 2012. URL: http://www.pubmedcentral.nih.gov/articlerender.fcgi?artid=3494743&tool=pmcentrez&rendertype=abstract, doi:10.1038/nbt.2391.

[10] Alexis a Borisy, Peter J Elliott, Nicole W Hurst, Margaret S Lee, Joseph Lehar, E Roydon Price, George Serbedzija, Grant R Zimmermann, Michael a Foley, Brent R Stockwell, and Curtis T Keith. Systematic discovery of multicomponent therapeutics. *Proceedings of the National Academy of Sciences of the United States of America*, 100(13):7977–82, June 2003. URL: http://www.pubmedcentral.nih.gov/articlerender.fcgi?artid=164698&tool=pmcentrez&rendertype=abstract, doi:10.1073/pnas.1337088100.
